# α-Vinylation of Ester Equivalents via Main Group Catalysis for the Construction of Quaternary Centers

**DOI:** 10.1021/acs.orglett.3c00535

**Published:** 2023-05-16

**Authors:** Chloe G. Williams, Sepand K. Nistanaki, Conner W. Wells, Hosea M. Nelson

**Affiliations:** Department of Chemistry and Chemical Engineering, California Institute of Technology, Pasadena, California 91125, United States

## Abstract

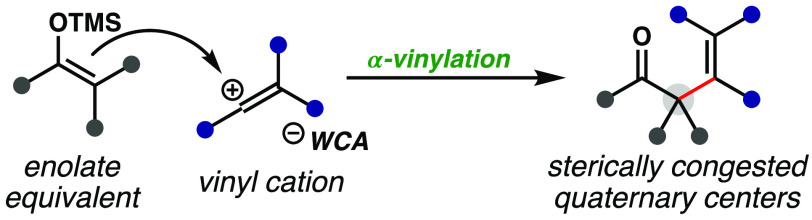

A methodology for the construction of sterically congested quaternary centers via the trapping of vinyl carbocations with silyl ketene acetals is disclosed. This main group-catalyzed α-vinylation reaction is advantageous as methods to access these congested motifs are limited. Moreover, β,γ-unsaturated carbonyl moieties and tetrasubstituted alkenes are present in various bioactive natural products and pharmaceuticals, and this catalytic platform offers a means of accessing them using simple and inexpensive materials.

All-carbon quaternary centers are critical structural motifs found in various natural products and pharmaceutical drug molecules.^1^ Along with increasing the structural complexity of molecules, these moieties have been shown to enhance the potency, selectivity, and metabolic stability of bioactive compounds targeted in drug discovery campaigns (Figure 1A, top).^1e^ For example, Mould and co-workers showed in the development of reversible inhibitors for lysine specific demethylase 1 (LSD1), a histone that plays a role in cancers such as leukemia, that the introduction of a quaternary center improved the molecule’s potency toward its target by more than double, increased its half-life in mouse microsomes, and improved hERG inhibition liability.^2^ Despite these clear advantageous effects, the construction of quaternary centers is still a challenging synthetic problem due to the high steric environment they contain.^3^ While enolate alkylation is a known strategy toward accessing quaternary centers, this approach is typically limited to the construction of C(sp^3^)–C(sp^3^) bonds, with few examples of Michael-type additions of 1,3-dicarbonyl compounds into activated alkynes reported.^4^ Moreover, various transition metal-catalyzed methodologies have been developed to form C(sp^3^)–C(sp^2^) bonds through cross-coupling of enolate equivalents with aryl and alkenyl electrophiles.^5^ Many of these include α-vinylation or α-arylation of ketone enolates or their derivatives using transition metals, such as palladium, nickel, copper, or ruthenium (Figure 1B).^6^ In particular, α-vinylation of enolate equivalents is a powerful approach toward accessing β,γ-unsaturated carbonyl motifs, which are prevalent in bioactive natural products and medicines (Figure 1A, bottom).^7^ While useful, two drawbacks of current catalytic α-vinylation methods exist: (1) the requirement of transition metal catalysts that can encompass laborious ligand syntheses and (2) limitations in constructing sterically congested motifs via the use of fully substituted alkenyl electrophiles.^5a,6a,6d^ Zaid and co-workers reported a transition metal-free method using stoichiometric base for accessing α-vinylated carbonyl compounds, though the scope of this reaction was limited to minimally substituted styrenes.^8^ Despite the existence of the above-mentioned transition metal methodologies, reports of forming quaternary centers via α-vinylation of carbonyl compounds are limited and often rely on the use of less substituted vinyl electrophiles.^6a,6c,6d,6f^ Therefore, there is a clear need for new methods to access α-vinylated quaternary centers bearing fully substituted vinyl electrophiles. To note, tetrasubstituted olefins are attractive functional groups in the pharmaceutical industry as they are present in bioactive molecules, such as the anticancer agents tamoxifen and etacstil.^9^

**Figure 1:**
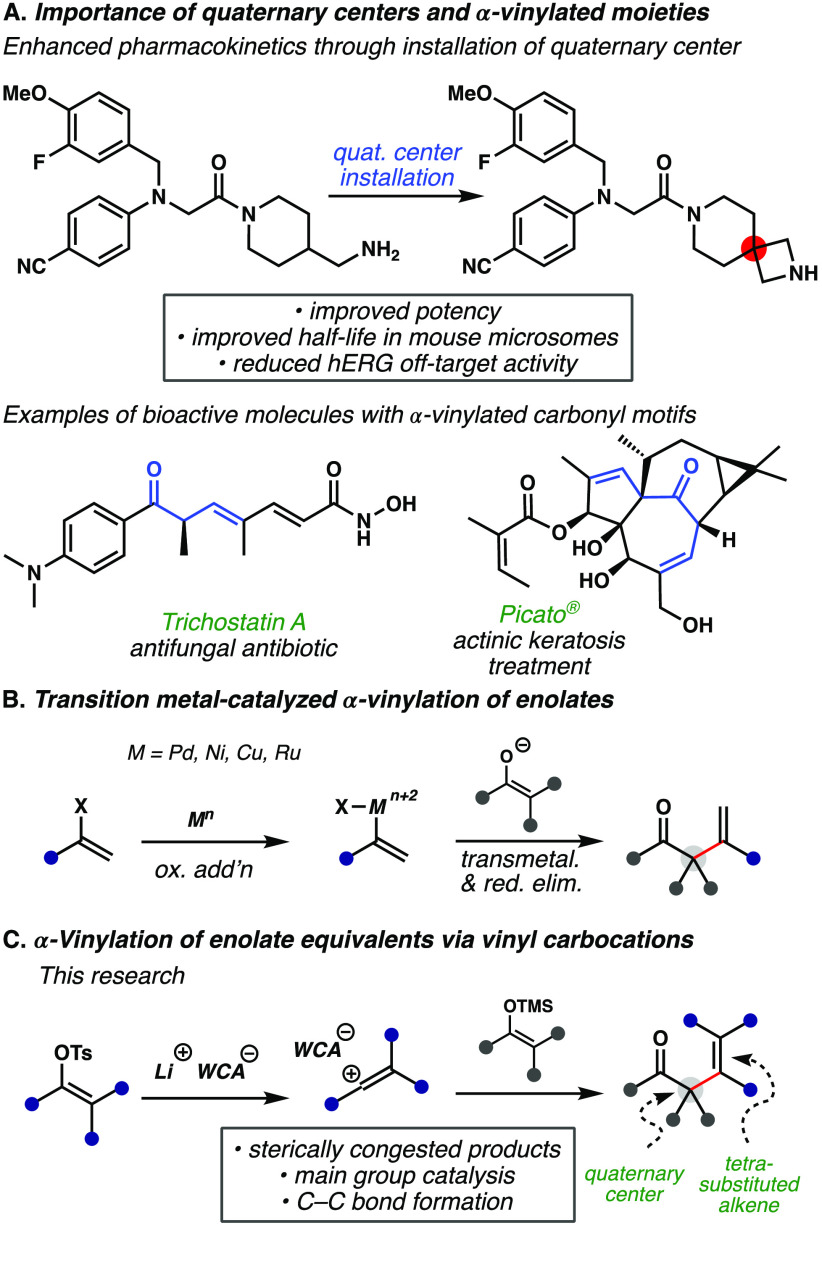
Motivation and strategies for accessing sterically congested all-carbon quaternary centers. (A) Relevance of quaternary centers and α-vinylated carbonyl moieties in bioactive molecules. (B) α-Vinylation of carbonyls via transition metal catalysis. (C) Lewis acid-catalyzed α-vinylation of carbonyl equivalents to access sterically congested structures.

In analogy to well-established enolate alkylation chemistry and precedent from stoichiometric flash photolysis studies performed by Mayr,^10^ it was hypothesized that catalytically generated electrophilic vinyl carbocations could be directly trapped by enolate equivalents to form α-vinylated carbonyl compounds (Figure 1C). Mild catalytic methods for the generation of vinyl cations from vinyl sulfonates have been reported recently using lithium/weakly coordinating anion (WCA) salts as the catalyst,^11^ which are significantly less expensive than transition metals used in previous α-vinylation methods.^12^ Moreover, given that increased substitution of vinyl sulfonates enables more facile ionization, it was hypothesized that this approach would allow for the generation of fully substituted vinyl carbocations that could directly engage in a nucleophilic attack by enolate equivalents.^13,14^ Herein we report a main group-catalyzed α-vinylation reaction to construct highly congested quaternary centers fused to tetrasubstituted olefins. During the late-stage preparation of this manuscript, Chen and co-workers disclosed trapping vinyl cations with silyl enol ethers to access difluoromethylene-skipped enones utilizing squareamide additives; however, though complementary to this report, this method does not appear to enable access to the sterically congested motifs of interest to this study.^15^

With reaction conditions inspired by previous work,^11^ initial studies commenced with exploring methyl ester silyl ketene acetal **1** and vinyl tosylate **2**, and gratifyingly, the desired product (**3**) was observed using 10 mol % [Li]^+^[B(C_6_F_5_)_4_]^−^ with 1.5 equiv of LiHMDS in *o*-DFB solvent with a 45% yield (Table 1, entry 1). We elected to utilize vinyl tosylates as the vinyl cation precursor because they are bench-stable crystalline solids that could tolerate full substitution on the olefin and electron-rich aromatic moieties, thereby expanding the scope of substrates that could be employed.^11,14,16^ The reaction in other solvents, such as *o*-DCB and cyclohexane, was not as efficient (entries 2–3), but when the reaction was performed in PhCF_3_, a 45% yield was also obtained (entry 4). Product was not observed when the reaction was performed without [Li]^+^[B(C_6_F_5_)_4_]^−^ catalyst (entry 5), but an increase in yield was observed by omitting the use of base (entry 6). Interestingly, in previous studies from our group, a stoichiometric lithium base was required for catalyst turnover.^11,14^ The yield was further improved by increasing the equivalents of silyl ketene acetal (entry 7). Other alkoxy groups on the silyl ketene acetal were also briefly surveyed, and it was found that using ethyl ester derived silyl ketene acetals resulted in an improvement in yield (entry 8). However, by implementing a bulkier isopropyl variant, a significant drop in yield was observed, likely due to Lewis acid-mediated dealkylation of the silyl ketene acetal and product, supported by mass spectrometry experiments (entry 9).

**Table 1:**
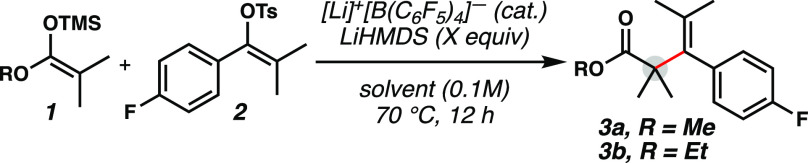

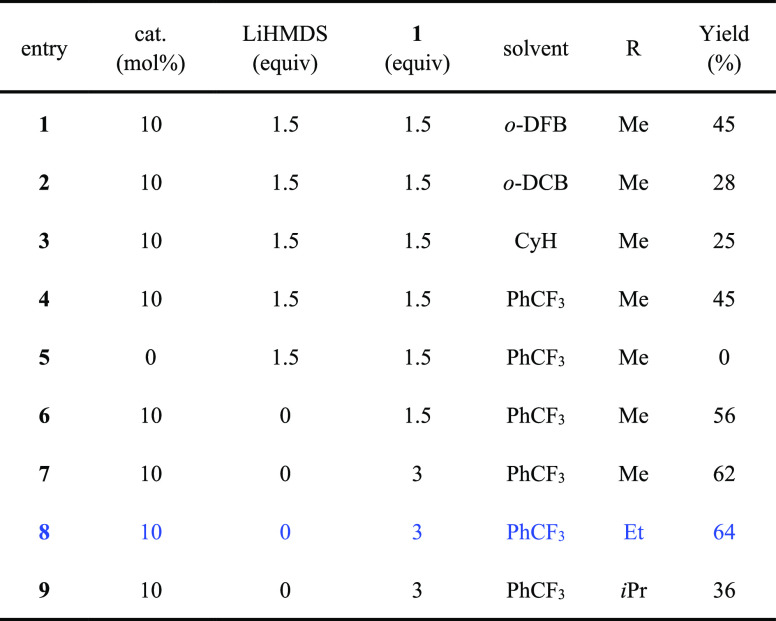
Optimization of α-Vinylation of Silyl Ketene Acetals with Main Group Catalyst^a^

a Yields determined by ^19^F NMR using C_6_F_6_ as an internal standard. OTs, *para-*toluenesulfonate; *o*-DFB, 1,2-difluorobenzene; *o*-DCB, 1,2-dichlorobenzene; CyH, cyclohexane.

With the optimized reaction conditions in hand, various substrates were studied (Figure 2). Both with ethoxy and methoxy silyl ketene acetals, sterically congested products **4** and **5** were isolated in good yields of 80% and 82% yield, respectively. Notably, tetrasubstituted olefins could be constructed, and this methodology tolerates substitution at the vinyl tosylate olefin to access various structures (**5**, **6**). More electron-rich vinyl tosylates were also tolerated, furnishing products **7** and **8** in good yields. Brominated and iodinated substrates were also compatible with the reaction conditions, delivering vinylation products **10** and **11**, albeit in slightly diminished yields. Current organometallic methods to access α-vinylated carbonyl compounds often rely on the use of palladium and nickel, which are typically incompatible with aryl halides. Upon exploration of the vinyl cation precursor, the silyl ketene acetal coupling component was also investigated. It was found that an unsymmetrical silyl ketene acetal can afford product **12** in good yield. Additionally, silyl ketene acetals bearing cyclic functional groups are also competent in the reaction (entry **13**, Figure 2). It was found that bulky benzyl groups on the silyl ketene acetal are also tolerated, furnishing sterically congested product **14** in 69% yield. These types of sterically encumbered scaffolds (tetrasubstituted olefin fused quaternary centers) are challenging to construct in a concise and catalytic manner, making this a useful method for the construction of α-vinylated quaternary centers. In addition to fully substituted silyl ketene acetals, trisubstituted variants also proved successful in this reaction, leading to products **15** and **16**, notably without olefin isomerization to the corresponding α,β-unsaturated ester. A phenyl substituent on the silyl ketene acetal delivered **17**, albeit in diminished yield.

**Figure 2:**
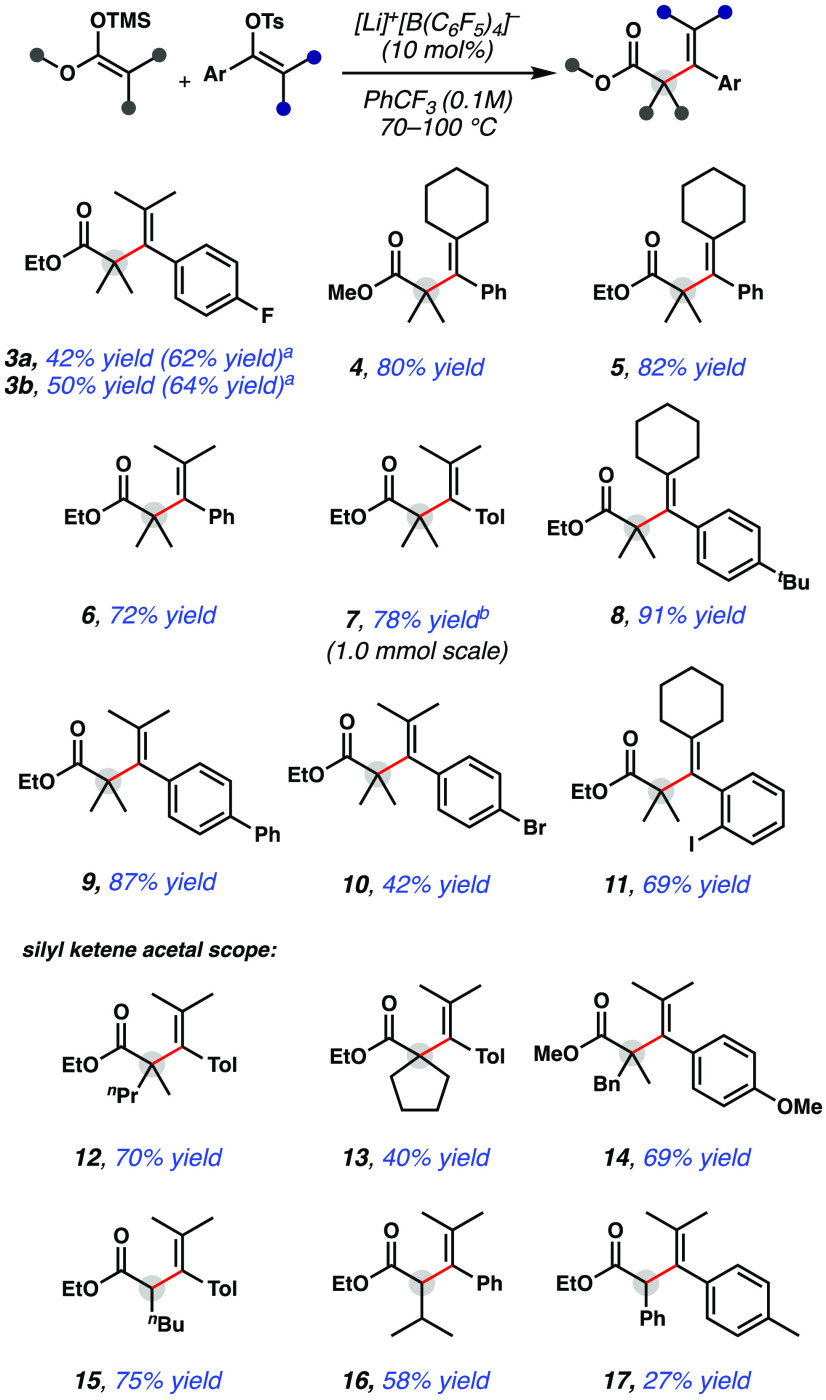
Lewis acid-catalyzed α-vinylation reactions of silyl ketene acetals. Isolated yield after column chromatography on 0.20 mmol scale with 3 equiv of silyl ketene acetal unless otherwise noted. ^a^Yield determined by ^19^F NMR using C_6_F_6_ as an internal standard. ^b^Reaction performed on 1.0 mmol scale. ^*t*^Bu, *tert*-butyl; ^*n*^Bu, *n*-butyl; ^*n*^Pr, *n*-propyl; Tol, *p*-tolyl; Bn, benzyl.

A proposed catalytic cycle is shown in Figure 3A. A Lewis acidic Li-based initiator (Li-WCA) undergoes initial ionization of the vinyl tosylate, forming vinyl cation **18**.^11^ Then, nucleophilic attack by the silyl ketene acetal occurs, forming oxocarbenium **19**, forging a new C–C bond and quaternary center. Since a stoichiometric lithium base is not required to obtain the desired product by turning over the catalytic cycle (Table 1), an in situ generated silyl Lewis acid is postulated to subsequently ionize vinyl tosylates to propagate the catalytic cycle. To probe whether the reaction could be catalyzed by silylium (instead of lithium), silyl ketene acetal **1** and vinyl tosylate **2** were subjected to silylium/WCA by mixing catalytic [Ph_3_C]^+^[B(C_6_F_5_)_4_]^−^ with silane, leading to in situ silylium generation via the Bartlett-Condon-Schneider hydride transfer reaction.^17^ This delivered the vinylated product **3a**, albeit in a lower yield (Figure 3B). This suggests that lithium is not needed for catalysis and supports that in situ silylium generation is a likely operative pathway for this reaction. Attempts to replace the silyl ketene acetal with its corresponding ester were unsuccessful, as the desired product was not observed in the presence or absence of a base. Finally, replacing the vinyl tosylate with the corresponding isobutyrophenone did not lead to the desired product, and only starting material was recovered, further supporting the proposed mechanism.

**Figure 3:**
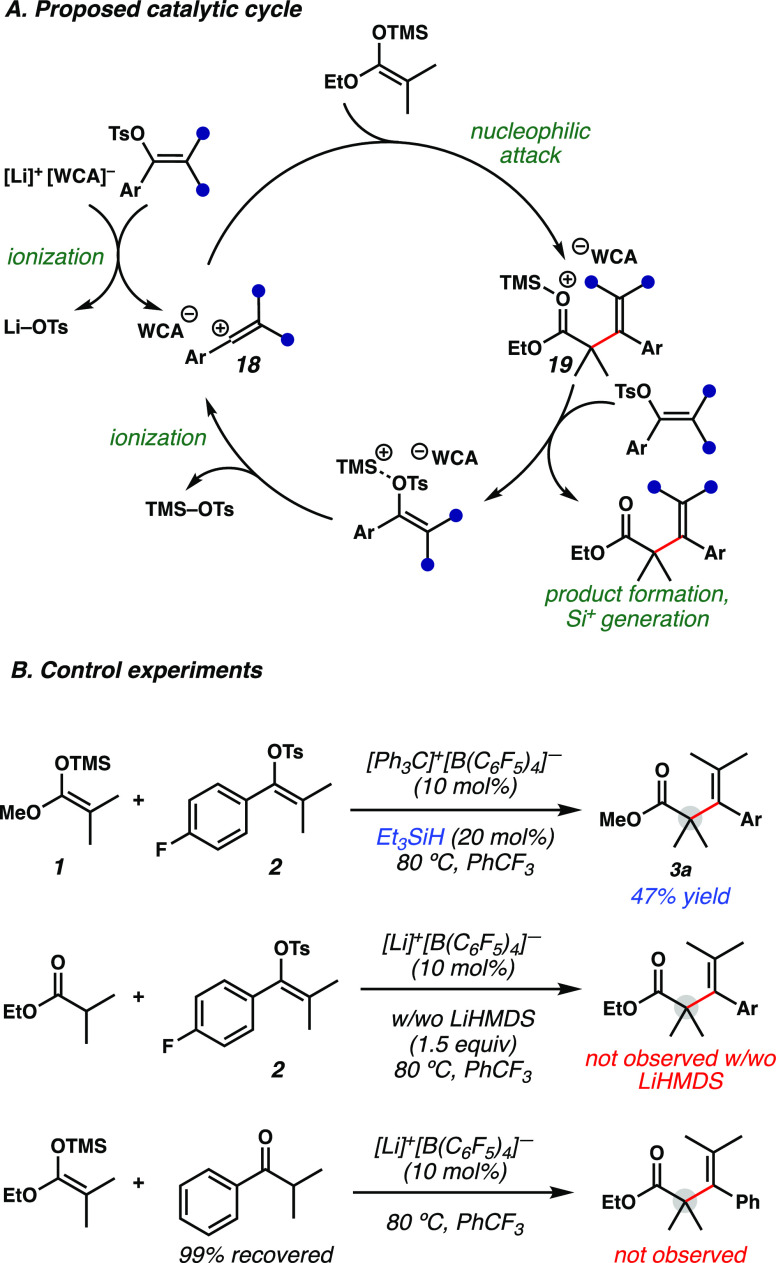
Mechanistic studies. (A) Proposed catalytic cycle. (B) Control experiments.

In efforts to study other methods for the generation of vinyl cations that could directly engage in intermolecular α-vinylation, the electrophilic alkylation of alkynes was explored, which has been a reported strategy to access vinyl carbocation intermediates.^18^ It was hypothesized that a tethered alkyl chain bearing an appropriate leaving group could be cyclized onto an alkyne through Lewis acid activation of the leaving group, and the resulting vinyl cation could then be trapped by silyl ketene acetals in an intermolecular fashion as discussed above (Figure 4). This alkyne difunctionalization cascade enables substitution at distal positions of the product to generate further complexity. To this end, it was discovered that alkyne substrates with an appended tosylate group can engage in alkyne alkylation/intermolecular nucleophilic trapping cascades to deliver tetrasubstituted olefin products **20**–**22**. In the case where a secondary tosylate is employed, which results in an unsymmetrical cyclopentane ring upon cyclization, a single olefin isomer was isolated in good yield, highlighting a selective addition step to forge tetrasubstituted *E*-olefins. These results highlight an alternative, alkyne difunctionalization approach for accessing sterically congested carbonyl compounds, which complement the vinyl tosylate ionization approach outlined in this study.

**Figure 4:**
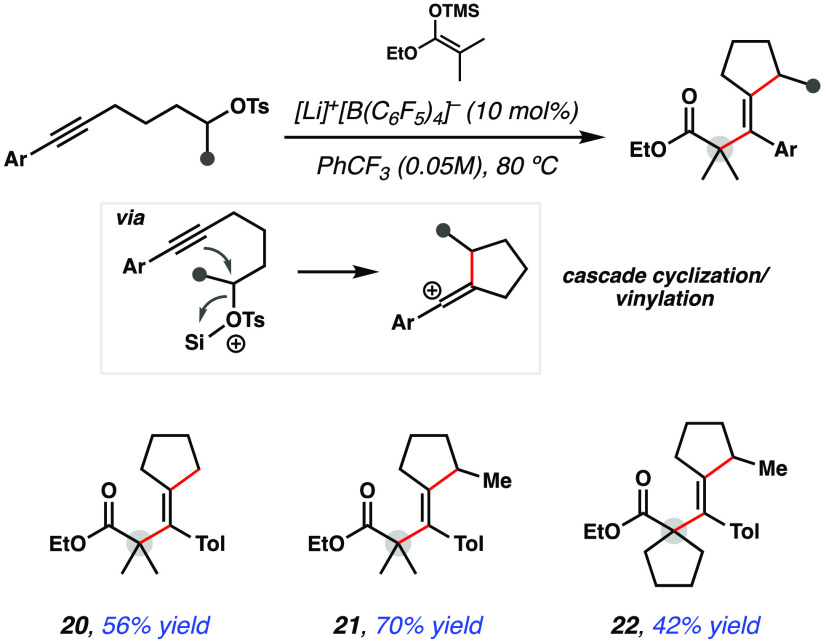
α-Vinylation of esters from alkyne starting materials. Isolated yield after column chromatography on 0.20 mmol scale with 3 equiv of silyl ketene acetal.

In conclusion, two main group-catalyzed approaches toward accessing sterically congested α-vinylated ester products through the trapping of vinyl cations with silyl ketene acetals are disclosed. Many of the catalytic approaches toward accessing α-vinylated ester products rely on transition metal catalysis, while here a simple main group salt is utilized in this transformation. Additionally, methods to construct α-vinylated carbonyl products bearing a tetrasubstituted alkene adjacent to a quaternary center are limited. This study opens the door toward further application of catalytically generated vinyl cation intermediates in synthesis, as well as the possibility to access these products in an asymmetric fashion. Overall, vinyl cations are underutilized reactive intermediates in catalysis,^19^ and this work highlights their ability to form sterically congested motifs.

## Data Availability

The data underlying this study are available in the published article and its Supporting Information.

## Supporting Information Available

Experimental procedures, characterization data, and NMR spectra (PDF)
