# Supplementary material for: α-Vinylation of Ester Equivalents via Main Group Catalysis for the Construction of Quaternary Centers
Source: Org Lett. 2023 May 16;25(20):3591–5. doi: 10.1021/acs.orglett.3c00535 (PMC10226172; doi:10.1021/acs.orglett.3c00535)
Supplement: Supplementary file 1 — ol3c00535_si_001.pdf [file ol3c00535_si_001.pdf]

Supporting Information for

α-Vinylation of Ester Equivalents *via* Main Group Catalysis for the Construction of Quaternary Centers

Chloe G. Williams, Sepand K. Nistanaki, Conner W. Wells and Hosea M. Nelson*

Department of Chemistry and Chemical Engineering, California Institute of Technology, Pasadena CA 91125, United States

*Corresponding author. Email: hosea@caltech.edu (H.M.N.)

Table of Contents

1. Materials and Methods....2

2. Preparation of Vinyl Tosylate....3–12

3. Preparation of Silyl Ketene Acetals....13–15

4. Preparation of Alkynyl Substrates....15–18

5. α-Vinylation of Esters with Vinyl Cations....18–34

5.1 General procedure for reactions with vinyl tosylates....18–31

5.2 Discussion of failed substrate class....31

5.3 General procedure for reactions with alkynes....32–34

6. Mechanistic Studies....35–36

7. References....36–37

8. NMR Spectra....38–124

1- Materials & Methods

Unless otherwise stated, all reactions were performed in an MBraun or VAC glovebox under nitrogen atmosphere with ≤ 0.5 ppm O_2 levels. All glassware and stir-bars were dried in a 160 °C oven for at least 12 hours and cycled directly into the glovebox for use. Solid substrates were dried on high vacuum over P_2O_5 overnight. All solvents were rigorously dried before use. 1,2-Dichloroethane, benzene, and trifluorotoluene were degassed and dried in a JC Meyer solvent system and stored inside a glovebox. Cyclohexane was distilled over potassium. *o*-Difluorobenzene was distilled over CaH_2. All other solvents used for substrate synthesis were dried in a JC Meyer solvent system. Diisopropylamine was distilled over CaH_2 prior to use. $[Li]^+[B(C_6F_5)_4]^-$ salt was synthesized according to literature procedure.[1] Preparatory thin layer chromatography (TLC) was performed using Millipore silica gel 60 F_{254} pre-coated plates (0.25 mm) and visualized by UV fluorescence quenching. SiliaFlash P60 silica gel (230-400 mesh) was used for flash chromatography. NMR spectra were recorded on a Bruker 400 MHz with Prodigy cryoprobe (^1H, ^{13}C, ^{31}P, ^{11}B), a Bruker 400 MHz (^1H, ^{13}C, ^{19}F), a Varian 300 MHz (^1H, ^{19}F), and a Bruker AV-500 (^1H, ^{13}C). ^1H NMR spectra are reported relative to $CDCl_3$ (7.26 ppm) unless noted otherwise. Data for ^1H NMR spectra are as follows: chemical shift (ppm), multiplicity, coupling constant (Hz), integration. Multiplicities are as follows: s = singlet, d = doublet, t = triplet, dd = doublet of doublet, dt = doublet of triplet, ddd = doublet of doublet of doublet, td = triplet of doublet, qd = quartet of doublets, m = multiplet. Structural assignments were made with additional information from gCOSY, gHSQC, and gHMBC experiments. ^{13}C NMR spectra are reported relative to $CDCl_3$ (77.1 ppm) unless noted otherwise. IR Spectra were record on a Thermo Scientific Nicolet iS50 FT-IR and are reported in terms of frequency absorption (cm^{-1}). High resolution mass spectra (HR-MS) were recorded on an Agilent 6230 time-of-flight LC/MS (LC/TOF) using electrospray ionization (ESI) or acquired by the Caltech Mass Spectral Facility by Field Ionization/Field Desorption mass spectrometry using a JEOL AccuTOF GC-Alpha (JMS-T2000GC) mass spectrometer interfaced with an Agilent 8890 GC system. Ions were detected as M+. (Radical cations). All commercial chemicals and reagents were used as received, unless otherwise noted. Lithium hexamethyldisilazide was purchased from Sigma Aldrich as a solid and brought in the glovebox as received.

2 – Preparation of Vinyl Tosylate Substrates

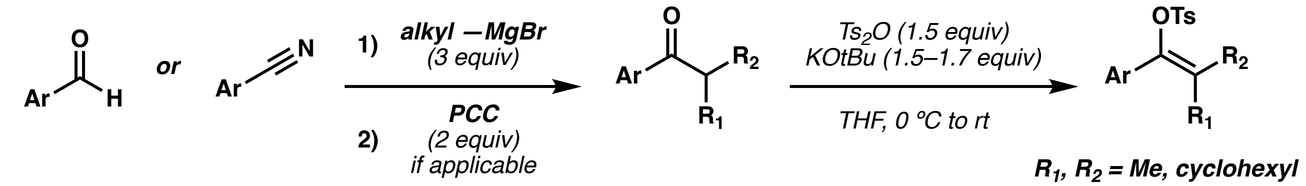

The procedure outlined above was used to prepare vinyl tosylate substrates from the corresponding ketone, which was either commercially available or synthesized from reported literature procedures through Grignard-addition to the aldehyde or benzonitrile.

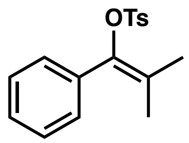

2-methyl-1-phenylprop-1-en-1-yl 4-methylbenzenesulfonate (SI-1)

To a flame-dried flask was added commercially available 2-methyl-1-phenylpropan-1-one (2.96 g, 1.0 equiv, 20.0 mmol) and THF (65.0 mL). This solution was cooled to 0 ºC, and then a solution of KOtBu (3.81 g, 1.7 equiv, 34.0 mmol) in THF (34.0 mL) was added dropwise. The resulting solution was then stirred at 0 ºC for 2 hours. Next, a solution of Ts_2O (9.79 g, 1.5 equiv, 30.0 mmol) in THF (50.0 mL) was added to the enolate solution with vigorous stirring, and then the solution was allowed to warm to room temperature and stirred for 1.5 hours (solution turns thick). The reaction was diluted with ethyl acetate (50 mL) and water (50 mL). The organic layer was separated, and the aqueous layer was extracted with ethyl acetate (3 x 20 mL), dried over Na_2SO_4, filtered, concentrated *in vacuo*, and purified by silica flash column chromatography (10% diethyl ether in hexanes) to give vinyl tosylate **SI-1** (3.5 g, 58% yield).

^{1}H NMR (400 MHz, $CDCl_3$) δ 7.44 (d, J = 8.4 Hz, 2H), 7.20 – 7.11 (m, 5H), 7.10 – 7.03 (m, 2H), 2.33 (s, 3H), 1.89 (s, 3H), 1.75 (s, 3H).

^{13}C NMR (101 MHz, $CDCl_3$) δ 144.3, 141.3, 134.4, 134.0, 129.6, 129.3, 128.0, 127.9, 127.8, 126.5, 21.6, 20.1, 19.2.

FT-IR (neat film NaCl): 3057, 3031, 2995, 2918, 2860, 2860, 1598, 1492, 1444, 1363, 1306, 1272, 1189, 1175, 1085, 1071, 1033, 992, 890, 820, 804, 789, 709, 698, 671, 582, 557, 544 cm^{-1}.

HR-MS (ESI) m/z: [M+H]+ Calculated for $C_{17}H_{19}O_3S$ 303.1049; Found 303.1050.

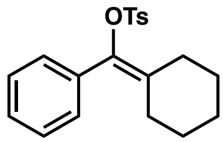

cyclohexylidene(phenyl)methyl 4-methylbenzenesulfonate (SI-2)

To a flame-dried flask was added commercially available cyclohexyl(phenyl)methanone (2.00 g, 1.0 equiv, 10.6 mmol) and THF (32.0 mL). This solution was cooled to 0 ºC, and then a solution of KOtBu (1.78 g, 1.5 equiv, 15.9 mmol) in THF (15.9 mL) was added dropwise. The resulting solution was then stirred at 0 ºC for 2 hours. Next, solid Ts_2O (5.19 g, 1.5 equiv, 15.9 mmol) was added to the enolate solution with vigorous stirring, and then the solution was allowed to warm to room temperature and stirred for 1.5 hours (solution turns thick). The reaction was diluted with ethyl acetate (50 mL) and water (50 mL). The organic layer was separated, and the aqueous layer was extracted with ethyl acetate (3 x 20 mL), dried over Na_2SO_4, filtered, concentrated *in vacuo*, and purified by silica flash column chromatography (5% ethyl acetate in hexanes) to give vinyl tosylate **SI-2** (1.01 g, 28% yield).

^{1}H NMR (400 MHz, $CDCl_3$) δ 7.48 – 7.40 (m, 2H), 7.22 – 7.10 (m, 5H), 7.09 – 7.01 (m, 2H), 2.40 (t, *J* = 5.8 Hz, 2H), 2.34 (s, 3H), 2.17 (t, *J* = 5.7 Hz, 2H), 1.65 – 1.46 (m, 6H).

^{13}C NMR (101 MHz, $CDCl_3$) δ 144.2, 138.8, 134.5, 133.8, 133.6, 129.7, 129.2, 128.04, 128.02, 127.8, 30.0, 28.9, 27.8, 27.2, 26.3, 21.6.

FT-IR (neat film NaCl): 3057, 2929, 2854, 1599, 1446, 1368, 1187, 1176, 1002, 786, 700, 555 cm^{-1}.

HR-MS (ESI) m/z: [M+K]+ Calculated for $C_{20}H_{22}O_3S$ 342.1290; Found 342.1294.

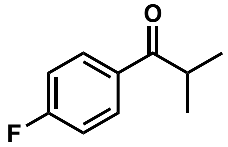

1-(4-fluorophenyl)-2-methylpropan-1-one (SI-3) was prepared according to literature procedures and matched the NMR data in the literature.[2]

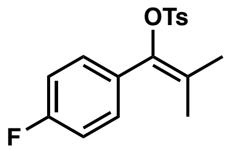

1-(4-fluorophenyl)-2-methylprop-1-en-1-yl 4-methylbenzenesulfonate (SI-4)
To a flame-dried flask was added **SI-3** (3.00 g, 1.0 equiv, 18.05 mmol) and THF (60.0 mL). This solution was cooled to 0 ºC, and then a solution of KOtBu (3.05 g, 1.5 equiv, 27.1 mmol) in THF (27.0 mL) was added dropwise. The resulting solution was then stirred at 0 ºC for 2 hours. Next, solid Ts_2O (8.84 g, 1.5 equiv, 27.1 mmol) was added to the enolate solution with vigorous stirring, and then the solution was allowed to warm to room temperature and stirred for 1.5 hours (solution turns thick). The reaction was diluted with ethyl acetate (30 mL) and water (30 mL). The organic layer was separated, and the aqueous layer was extracted with ethyl acetate (3 x 20 mL), dried over Na_2SO_4, filtered, concentrated *in vacuo*, and purified by silica flash column chromatography (7% diethyl ether in hexanes) to give vinyl tosylate **SI-4** (3.8 g, 66% yield).

^{1}H NMR (400 MHz, $CDCl_3$) δ 7.46 (d, *J* = 8.3 Hz, 2H), 7.17 – 7.06 (m, 4H), 6.83 (t, *J* = 8.7 Hz, 2H), 2.36 (s, 3H), 1.88 (s, 3H), 1.73 (s, 3H).
^{13}C NMR (101 MHz, $CDCl_3$) δ 162.2 (d, *J* = 248.1 Hz), 144.4, 140.1, 134.3, 131.3 (d, *J* = 8.3 Hz), 130.0 (d, *J* = 3.2 Hz), 129.2, 127.8, 126.7, 114.7 (d, *J* = 21.6 Hz), 21.5, 19.9, 19.0.
^{19}F NMR (282 MHz, $CDCl_3$) δ -112.9.
FT-IR (neat film NaCl): 3069, 2994, 2920, 2861, 1601, 1508, 1366, 1189, 1177, 1082, 995, 844, 784, 669 cm^{-1}.
HR-MS (ESI) m/z: [M+K]+ Calculated for $C_{17}H_{17}FO_3S$ 320.0883; Found 320.0883.

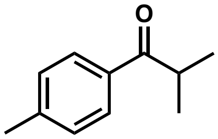

2-methyl-1-(*p*-tolyl)propan-1-one (SI-5) was prepared according to literature procedures and matched the NMR data in the literature.[2]

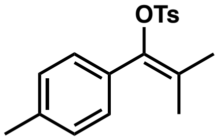

2-methyl-1-(*p*-tolyl)prop-1-en-1-yl 4-methylbenzenesulfonate (SI-6)

To a flame-dried flask was added **SI-5** (1.64 g, 1.0 equiv, 10.1 mmol) and THF (33.0 mL). This solution was cooled to 0 ºC, and then a solution of KOtBu (1.93 g, 1.7 equiv, 17.2 mmol) in THF (17.2 mL) was added dropwise. The resulting solution was then stirred at 0 ºC for 2 hours. Next, a solution of Ts_2O (4.96 g, 1.5 equiv, 15.2 mmol) in THF (25.3 mL) was added to the enolate solution with vigorous stirring, and then the solution was allowed to warm to room temperature and stirred for 1.5 hours (solution turns thick). The reaction was diluted with ethyl acetate (30 mL) and water (30 mL). The organic layer was separated, and the aqueous layer was extracted with ethyl acetate (3 x 20 mL), dried over Na_2SO_4, filtered, concentrated *in vacuo*, and purified by silica flash column chromatography (7% diethyl ether in hexanes) to give vinyl tosylate **SI-6** (1.2 g, 38% yield).

^{1}H NMR (400 MHz, $CDCl_3$)) δ 7.45 – 7.34 (m, 2H), 7.03 – 6.98 (m, 2H), 6.98 – 6.93 (m, 2H), 6.91 – 6.84 (m, 2H), 2.28 (s, 3H), 2.21 (s, 3H), 1.78 (s, 3H), 1.67 (s, 3H).
^{13}C NMR (101 MHz, $CDCl_3$) δ 144.2, 141.4, 137.9, 134.6, 131.2, 129.5, 129.2, 128.5, 128.1, 125.9, 21.7, 21.4, 20.2, 19.2.
FT-IR (neat film NaCl): 3029, 2994, 2919, 2861, 1598, 1511, 1449, 1365, 1307, 1189, 1176, 1082, 993, 830, 812, 784, 670, 560 cm^{-1}.
HR-MS (ESI) m/z: [M+Na]+ Calculated for $C_{18}H_{20}NaO_3S$ 339.1025; Found 339.1026.

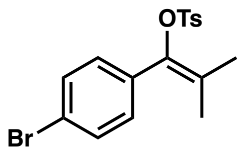

1-(4-bromophenyl)-2-methylprop-1-en-1-yl 4-methylbenzenesulfonate (SI-7)

To a flame-dried flask was added commercially available **1-(4-bromophenyl)-2-methylpropan-1-one** (1.00 g, 1.0 equiv, 4.40 mmol) and THF (14.7 mL). This solution was cooled to 0 ºC, and then a solution of KOtBu (741 mg, 1.5 equiv, 6.61 mmol) in THF (6.6 mL) was added dropwise. The resulting solution was then stirred at 0 ºC for 2 hours. Next, solid Ts_2O (2.16 g, 1.5 equiv, 6.61 mmol) was added to the enolate solution with vigorous stirring, and then the solution was allowed to warm to room temperature and stirred for 1.5 hours (solution turns thick). The reaction was diluted with ethyl acetate (30 mL) and water (30 mL). The organic layer was separated, and the aqueous layer was extracted with ethyl acetate (3 x 20 mL), dried over

Na_2SO_4, filtered, concentrated *in vacuo*, and purified by silica flash column chromatography (15% diethyl ether in hexanes) to give vinyl tosylate **SI-7** (1.2 g, 71% yield).

^{1}H NMR (400 MHz, $CDCl_3$) δ 7.45 (d, *J* = 8.3 Hz, 2H), 7.26 – 7.22 (m, 2H), 7.12 – 7.08 (m, 2H), 7.01 – 6.96 (m, 2H), 2.38 (s, 3H), 1.88 (s, 3H), 1.74 (s, 3H).

^{13}C NMR (101 MHz, $CDCl_3$) δ 144.7, 140.2, 134.3, 132.9, 131.2, 131.0, 129.4, 128.0, 127.4, 122.2, 21.7, 20.1, 19.2.

FT-IR (neat film NaCl): 3066, 2991, 2920, 2858, 1597, 1589, 1485, 1448, 1367, 1190, 1175, 1083, 993, 835, 812, 785, 734, 664, 589, 559 cm^{-1}.

HR-MS (FD) m/z: [M•]+ Calculated for $C_{17}H_{17}BrO_3S$ 380.0076; Found 380.0082.

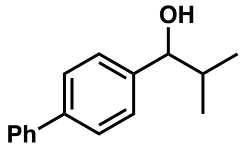

1-([1,1'-biphenyl]-4-yl)-2-methylpropan-1-ol (SI-8)

Procedure adapted from the reported literature.[3] To a flame-dried flask was added commercially available [1,1'-biphenyl]-4-carbaldehyde (3.00 g, 1.0 equiv, 16.46 mmol) and THF (16 mL), and this flask was cooled to 0 ºC. Then, 2M isopropylmagnesium chloride (8.2 mL, 1 equiv, 16.46 mmol) was added dropwise and the reaction was allowed to stir at 0 ºC. Upon full consumption of starting material, saturated NH_4Cl was added, and the crude reaction was extracted with ethyl acetate (3x 20 mL). The combined organic layers were washed with water, followed by brine, and then dried with Na_2SO_4 and concentrated *in-vacuo.* Pure material was obtained by silica flash column chromatography (15% ether in hexanes) to afford white solid **SI-8** (1.1 g, 29% yield).

^{1}H NMR (500 MHz, $CDCl_3$) δ 7.66 – 7.62 (m, 4H), 7.50 (dd, *J* = 8.4, 6.9 Hz, 2H), 7.46 – 7.44 (m, 2H), 7.42 – 7.37 (m, 1H), 4.49 (d, *J* = 6.8 Hz, 1H), 2.07 (h, *J* = 6.7 Hz, 1H), 1.10 (d, *J* = 6.6 Hz, 3H), 0.91 (d, *J* = 6.8 Hz, 3H).

^{13}C NMR (101 MHz, $CDCl_3$) δ 142.8, 141.0, 140.4, 128.9, 127.4, 127.2, 127.1, 127.0, 79.9, 35.4, 19.2, 18.4.

FT-IR (neat film NaCl): 3390, 3056, 3028, 2957, 2870, 1600, 1486, 1468, 1405, 1384, 1365, 1175, 1029, 1016, 1007, 836, 761, 737, 696, 574, 507 cm^{-1}.

HR-MS (ESI) m/z: [M–H_2O]+ Calculated for $C_{16}H_{17}$ 209.1325; Found 209.1329.

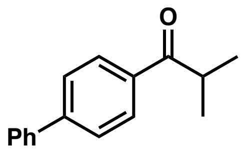

1-([1,1'-biphenyl]-4-yl)-2-methylpropan-1-one (SI-9)

To a flame-dried flask was added PCC (2.03 g, 2.0 equiv, 9.43 mmol) and DCM (19 mL). **SI-8** was then added dropwise. The resulting solution was stirred until the starting material was fully consumed, as monitored by TLC. Upon completion, the reaction was plugged through a short silica plug with DCM and then concentrated to afford **SI-9,** which was used without further purification (0.953 g, 90% yield). NMR data matched those reported in the literature.[4]

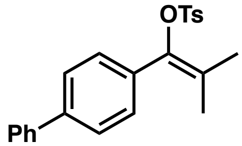

1-([1,1'-biphenyl]-4-yl)-2-methylprop-1-en-1-yl 4-methylbenzenesulfonate (SI-10)

To a flame-dried flask was added **SI-9** (0.953 g, 1.0 equiv, 4.25 mmol) and THF (14.2 mL). This solution was cooled to 0 ºC, and then a solution of KOtBu (715 mg, 1.5 equiv, 6.37 mmol) in THF (6.4 mL) was added dropwise. The resulting solution was then stirred at 0 ºC for 2 hours. Next, solid Ts_2O (2.08 g, 1.5 equiv, 6.37 mmol) was added to the enolate solution with vigorous stirring, and then the solution was allowed to warm to room temperature and stirred for 1.5 hours (solution turns thick). The reaction was diluted with ethyl acetate (30 mL) and water (30 mL). The organic layer was separated, and the aqueous layer was extracted with ethyl acetate (3 x 20 mL), dried over Na_2SO_4, filtered, concentrated *in vacuo*, and purified by silica flash column chromatography (15% diethyl ether in hexanes) to give vinyl tosylate **SI-10** (838 mg, 52% yield).

^{1}H NMR (400 MHz, $CDCl_3$) δ 7.53 – 7.49 (m, 2H), 7.49 – 7.42 (m, 4H), 7.38 – 7.32 (m, 3H), 7.21 – 7.16 (m, 2H), 7.08 – 7.01 (m, 2H), 2.29 (s, 3H), 1.93 (s, 3H), 1.82 (s, 3H).

^{13}C NMR (101 MHz, $CDCl_3$) δ 144.7, 140.2, 134.3, 132.9, 131.2, 131.0, 129.4, 128.1, 127.4, 122.2, 21.7, 20.1, 19.2.

FT-IR (neat film NaCl): 3031, 2993, 2918, 2858, 1598, 1486, 1366, 1189, 1176, 1082, 992, 848, 808, 789, 755, 735, 698, 670, 586, 571, 552 cm^{-1}.

HR-MS (ESI) m/z: [M+Na]+ Calculated for $C_{23}H_{22}NaO_3S$ 401.1182; Found 401.1184.

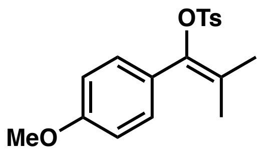

1-(4-methoxyphenyl)-2-methylprop-1-en-1-yl 4-methylbenzenesulfonate (SI-11)

To a flame-dried flask was added commercially available 1-(4-methoxyphenyl)-2-methylpropan-1-one **(**4.80 g, 1.0 equiv, 26.9 mmol) and THF (87 mL). This solution was cooled to 0 ºC, and then a solution of KOtBu (5.14, 1.7 equiv, 45.8 mmol) in THF (46 mL) was added dropwise. The resulting solution was then stirred at 0 ºC for 2 hours. Next, a solution of Ts_2O (13.2 g, 1.5 equiv, 40.4 mmol) in THF (67 mL) was added to the enolate solution with vigorous stirring, and then the solution was allowed to warm to room temperature and stirred for 1.5 hours (solution turns thick). The reaction was diluted with ethyl acetate (30 mL) and water (30 mL). The organic layer was separated, and the aqueous layer was extracted with ethyl acetate (3 x 20 mL), dried over Na_2SO_4, filtered, concentrated *in vacuo*, and purified by silica flash column chromatography (15% diethyl ether in hexanes) to give vinyl tosylate **SI-11** (5.4 g, 60% yield). The purified material matched the NMR data in the literature.[5]

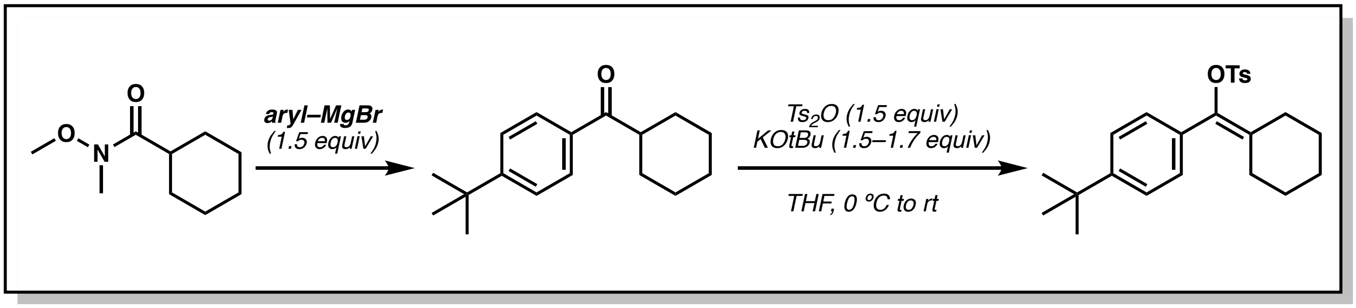

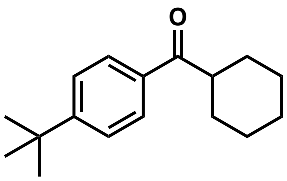

(4-(*tert*-butyl)phenyl)(cyclohexyl)methanone (SI-12)

SI-12 was synthesized by following a reported procedure.[6] To a flask was added magnesium turnings (1.96 g, 1.5 equiv, 80.6 mmol) and the flask was flame-dried 3x under vacuum. THF (81 mL) was then added with a spec of iodine. 1-bromo-4-(tert-butyl)benzene (17.7 mL, 1.9 equiv, 102 mmol) was added, and then the reaction flask was gently heated with a heat gun until the reaction initiated, as indicated by dissipation of iodine color. The reaction was then stirred until

all magnesium turnings had been consumed. Upon consumption of magnesium, the reaction was cooled to 0 ºC, and a solution of N-methoxy-N-methylcyclohexanecarboxamide (9.20 g, 1.0 equiv, 53.4 mmol) in THF (179 mL) was added dropwise. Upon consumption of the starting material in about 10 minutes (TLC 60% ethyl acetate in hexanes), saturated NH_4Cl was added to quench the reaction. The reaction was then extracted with ethyl acetate (3x), and the combined organics were washed with water, then brine, dried with Na_2SO_4, and concentrated *in vacuo.* The crude material was flashed via silica flash column chromatography (20% ethyl acetate in hexanes) to afford colorless oil **SI-12** (4.0 g, 30% yield) which matched reported literature spectra.[7]

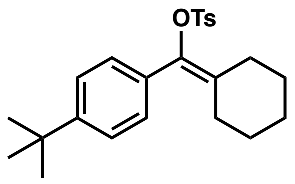

(4-(*tert*-butyl)phenyl)(cyclohexylidene)methyl 4-methylbenzenesulfonate (SI-13)
To a flame-dried flask was added **SI-12** (1.00 g, 1.0 equiv, 4.09 mmol) and THF (13.3 mL). This solution was cooled to 0 ºC, and then a solution of KOtBu (689 mg, 1.5 equiv, 6.14 mmol) in THF (6.14 mL) was added dropwise. The resulting solution was then stirred at 0 ºC for 2 hours. Next, solid Ts_2O (2.00 g, 1.5 equiv, 6.14 mmol) was added to the enolate solution with vigorous stirring, and then the solution was allowed to warm to room temperature and stirred for 1.5 hours (solution turns thick). The reaction was diluted with ethyl acetate (30 mL) and water (30 mL). The organic layer was separated, and the aqueous layer was extracted with ethyl acetate (3 x 20 mL), dried over Na_2SO_4, filtered, concentrated *in vacuo*, and purified by silica flash column chromatography (7% diethyl ether in hexanes) to give vinyl tosylate **SI-13** (340 mg, 21% yield).

^{1}H NMR (400 MHz, $CDCl_3$) δ 7.41 – 7.33 (m, 2H), 7.10 – 7.06 (m, 2H), 7.02 – 6.96 (m, 4H), 2.48 – 2.43 (m, 2H), 2.30 (s, 3H), 2.17 (s, 2H), 1.65 – 1.50 (m, 6H), 1.25 (s, 9H).
^{13}C NMR (101 MHz, $CDCl_3$) δ 151.0, 143.7, 139.0, 134.8, 133.2, 130.5, 129.4, 129.1, 128.0, 124.6, 34.6, 31.4, 30.1, 29.0, 27.8, 27.3, 26.4, 21.6.
FT-IR (neat film NaCl): 2962, 2929, 2854, 1598, 1449, 1366, 1187, 1175, 1106, 1094, 1021, 1003, 981, 903, 844, 825, 812, 780, 730, 667, 579, 569, 556 cm^{-1}.
HR-MS (ESI) m/z: [M+Na]+ Calculated for $C_{24}H_{30}NaO_3S$ 421.1808; Found 421.1809.

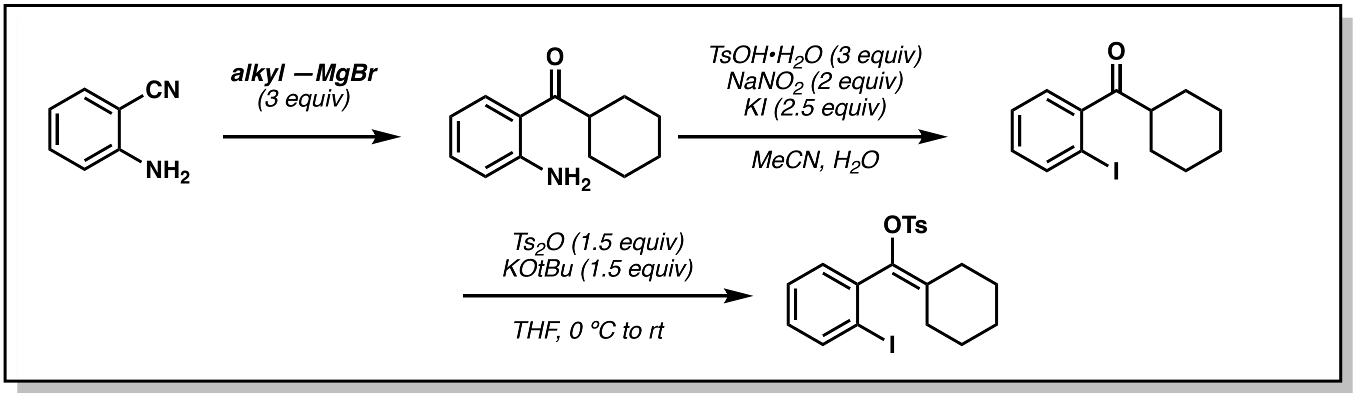

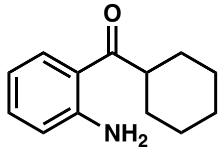

(2-aminophenyl)(cyclohexyl)methanone (SI-14)

Following a reported procedure[8], to a flame-dried flask, 2-aminobenzonitrile (12.0 g, 1.0 equiv, 101.6 mmol) was suspended in THF (101 mL) and the flask was cooled to 0 ºC. Then, 1M cyclohexylmagnesium bromide (290 mL, 3.0 equiv, 305 mmoL) was added dropwise. After addition was complete, the reaction was warmed to room temperature. Starting material was consumed after about 4 hours (monitored by TLC). The reaction was then cooled to 0 ºC, and water was slowly added, followed by conc. HCl. The reaction was then extracted with diethyl ether 3x, and the combined organics were dried with $MgSO_4$ and concentrated. The crude reaction mixture was purified *via* silica flash column chromatography (20% ether/hexanes) to afford **SI-14** (14.9 g, 72% yield). NMR spectra matched those reported in the literature.[8]

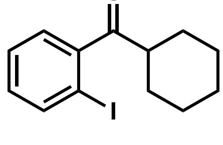

cyclohexyl(2-iodophenyl)methanone (SI-15)

Following a reported procedure[9], *p*-toluenesulfonic acid monohydrate (11.2 g, 3.00 equiv, 59.0 mmol) was added to a flask with MeCN (80 mL), followed by **SI-14** (4.00 g, 1.0 equiv, 19.7 mmol). The solution was cooled to 0 ºC, and a solution of $NaNO_2$ (2.71 g, 2.0 equiv, 39.4 mmol) in water (6 mL) was added dropwise over 5 minutes. Then, a solution of KI (8.17 g, 2.50 equiv, 49.2 mmol) in water (8 mL) was added slowly. The reaction was allowed to stir at 0 ºC for 10 additional minutes, then was warmed to room temperature and stirred for 3 hours. Water was

then added and then the reaction was basified to pH 9 with saturated $NaHCO_3$. Saturated $Na_2S_2O_3$ was added next. The reaction was then extracted with EtOAc (3x 250 mL), and the combined organic layers were washed with brine and dried with Na_2SO_4, and then concentrated. Pure product **SI-15** was obtained via silica flash column chromatography (2% -->6% diethyl ether in hexanes and matched the reported spectra (5.10 g, 83% yield).[10]

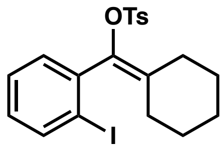

cyclohexylidene(2-iodophenyl)methyl 4-methylbenzenesulfonate (SI-16)

To a flame-dried flask was added **SI-15** (2.50 g, 1.0 equiv, 7.96 mmol) and THF (30 mL). This solution was cooled to 0 ºC, and then a solution of KOtBu (1.34 g, 1.5 equiv, 11.9 mmol) in THF (45 mL) was added dropwise. The resulting solution was then stirred at 0 ºC for 2 hours. Next, solid Ts_2O (3.90 g, 1.5 equiv, 11.9 mmol) was added to the enolate solution with vigorous stirring, and then the solution was allowed to warm to room temperature and stirred for 1.5 hours (solution turns thick). The reaction was diluted with ethyl acetate (30 mL) and water (30 mL). The organic layer was separated, and the aqueous layer was extracted with ethyl acetate (3 x 20 mL), dried over Na_2SO_4, filtered, concentrated *in vacuo*, and purified by silica flash column chromatography (15% diethyl ether in hexanes) to give vinyl tosylate **SI-16** (3.05 g, 82% yield).

^{1}H NMR (500 MHz, $CDCl_3$) δ 7.63 (dd, *J* = 7.9, 1.2 Hz, 1H), 7.52 – 7.47 (m, 2H), 7.23 (dtd, *J* = 15.9, 7.9, 6.3 Hz, 2H), 7.07 (d, *J* = 8.0 Hz, 2H), 6.87 (td, *J* = 7.5, 1.9 Hz, 1H), 2.51 (ddd, *J* = 13.6, 6.9, 4.8 Hz, 1H), 2.37 (ddd, *J* = 13.0, 7.6, 4.8 Hz, 1H), 2.33 (s, 3H), 1.93 (t, *J* = 5.8 Hz, 2H), 1.65 (h, *J* = 5.5 Hz, 2H), 1.60 – 1.48 (m, 4H).

^{13}C NMR (126 MHz, $CDCl_3$) δ 144.1, 139.3, 139.1, 138.7, 135.2, 134.5, 132.9, 129.8, 129.2, 127.8, 127.5, 100.1, 30.3, 28.4, 27.6, 27.1, 26.4, 21.7.

FT-IR (neat film NaCl): 3064, 2927, 2853, 1598, 1460, 1448, 1431, 1364, 1307, 1257, 1232, 1209, 1188, 1175, 1117, 1095, 1051, 1018, 1002, 979, 827 cm^{-1}.

HR-MS (ESI) m/z: [M+K]+ Calculated for 491.0154 Observed: 491.0143.

3 – Preparation of Silyl Ketene Acetals

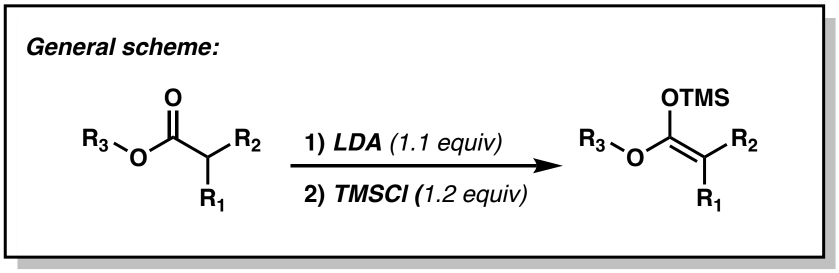

The general reaction scheme outlined above was used to prepare silyl ketene acetals from commercially available esters and was adapted from the literature.[11]

To a flame-dried flask was added diisopropylamine (1.1 equiv) and THF (0.66 M) and cooled to 0 ºC. Then, a solution of 2.5 M *n*-Butyllithium (1.1 equiv) was added dropwise, and the solution was allowed to warm to room temperature and stirred for 30 minutes. The reaction was then cooled to -78 ºC and the appropriate ester was added dropwise (1.0 equiv), and the resulting solution was stirred for 1 hour at -78 ºC. TMS-Cl (1.2 equiv) was subsequently added dropwise at -78 ºC, and the reaction was allowed to slowly warm up to room temperature overnight. The reaction was then concentrated *in-vacuo,* and then pentanes was then added. The suspension was filtered through a pad of celite, concentrated once more, and then distilled for purification to afford colorless oils.

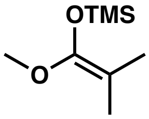

((1-methoxy-2-methylprop-1-en-1-yl)oxy)trimethylsilane was purchased and used as received.

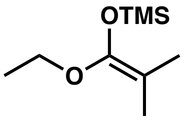

((1-ethoxy-2-methylprop-1-en-1-yl)oxy)trimethylsilane (SI-17) was prepared according to the described procedure on 30.00 mmol scale (50% yield, 3g) and matches reported spectra.[12]

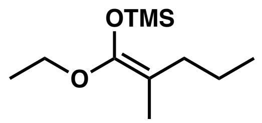

((1-ethoxy-2-methylpent-1-en-1-yl)oxy)trimethylsilane (SI-18) (mixture of E/Z isomers) was prepared according to the described procedure on 30.0 mmol scale (60% yield, 4 g) and obtained as a mixture of E/Z isomers (E/Z ratio 60:40). The compound matches reported spectra.[13]

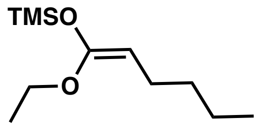

((1-ethoxyhex-1-en-1-yl)oxy)trimethylsilane (SI-19) (mixture of E/Z isomers) was prepared according to the described procedure on 30.2 mmol scale (60% yield, 4 g) and was obtained as a mixture of E/Z isomers (E/Z ratio = 94:6). Product was assigned as E olefin isomer by comparing to similar silyl ketene acetals.[14]

^{1}H NMR (400 MHz, $CDCl_3$) δ 3.82 (q, *J* = 7.1 Hz, 2H), 3.72 (t, *J* = 7.3 Hz, 1H), 1.99 – 1.93 (m, 2H), 1.31 – 1.25 (m, 4H), 1.22 (t, *J* = 7.1 Hz, 3H), 0.89 (m, 3H), 0.21 (s, 9H).
^{13}C NMR (101 MHz, $CDCl_3$) δ 152.8, 87.2, 63.3, 33.2, 24.5, 22.5, 15.2, 14.2, 0.02.
FT-IR (neat film NaCl): 2958, 2932, 2873, 2861, 1737, 1466, 1373, 1251, 1178, 1110, 1038, 845, 729, 677 cm^{-1}.
HR-MS (FI) m/z: [M•]+ Calculated for $C_{11}H_{24}O_2Si$ 216.1540; Found 216.1545.

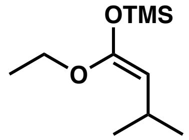

(*E*)-((1-ethoxy-3-methylbut-1-en-1-yl)oxy)trimethylsilane (SI-20) (mixture of E/Z isomers) was prepared according to the described procedure on 30.0 mmol scale (70% yield) with E/Z ratio = 98:2. The compound matches the reported literature.[15]

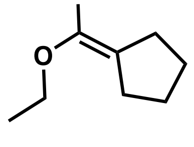

(cyclopentylidene(ethoxy)methoxy)trimethylsilane (SI-21) was prepared according to the described procedure on 37.3 mmol scale (50% yield, 4 g).

^{1}H NMR (400 MHz, $CDCl_3$) δ 3.85 – 3.72 (m, 2H), 2.24 – 2.16 (m, 2H), 2.12 (dddd, *J* = 8.3, 4.6, 2.4, 1.0 Hz, 2H), 1.61 – 1.56 (m, 4H), 1.22 (t, *J* = 7.1 Hz, 3H), 0.20 (s, 9H).

^{13}C NMR (101 MHz, $CDCl_3$) δ 145.9, 102.2, 64.1, 28.4, 27.8, 27.2, 27.0, 15.2, 0.3.

FT-IR (neat film NaCl): 2955, 2898, 2867, 2845, 2357, 1713, 1443, 1389, 1315, 1252, 1232, 1215, 1178, 1150, 1081, 1028, 1005, 949, 875, 845, 756, 697 cm^{-1}.

HR-MS (FI) m/z: [M•]+ Calculated for $C_{11}H_{22}O_2Si$ 214.1402; Found 214.1389.

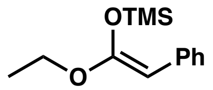

((1-ethoxy-2-phenylvinyl)oxy)trimethylsilane (mixture of E/Z isomers) (SI-22) was prepared according to the described procedure on 30.0 mmol scale (40% yield) with E/Z ratio = 1:21. The compound matches the reported literature.[16]

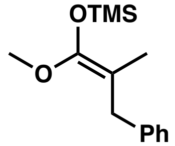

((1-methoxy-2-methyl-3-phenylprop-1-en-1-yl)oxy)trimethylsilane (mixture of E/Z isomers) (SI-23) was prepared according to the described procedure on 12.9 mmol scale (60% yield) with E/Z ratio = 6.4:1. The compound matches the reported literature.[17]

4 – Preparation of Alkyne Cyclization Substrates

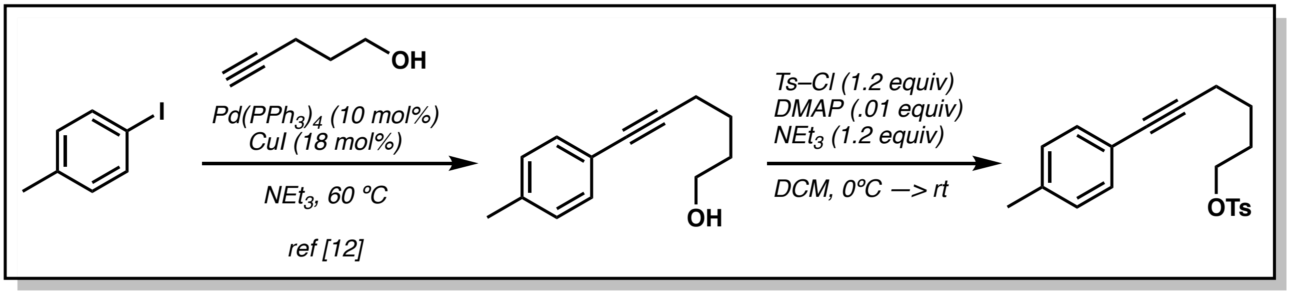

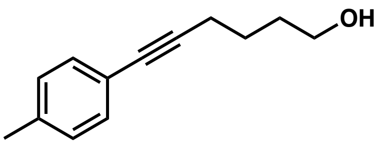

6-(*p*-tolyl)hex-5-yn-1-ol (SI-24)

This compound was prepared according to a reported procedure[18] and all spectra match reported.[19]

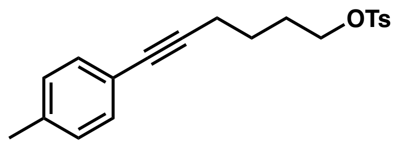

6-(*p*-tolyl)hex-5-yn-1-yl 4-methylbenzenesulfonate (SI-25)

Alcohol **SI-24** (800 mg, 1.0 equiv, 4.25 mmol) was dissolved in dry DCM (30 mL) in a flame-dried flask. The solution was cooled to 0 ºC, then 4-dimethylaminopyridine (DMAP) (5.2 mg, 0.01 equiv, 0.04 mmol) was added, followed by 4-toluenesulfonyl chloride (Ts-Cl) (972 mg, 1.2 equiv, 5.10 mmol) add as solids in one portion. Then, dry (distilled over CaH_2) triethylamine (0.71 mL, 1.2 equiv, 5.10 mmol) was added dropwise. The mixture was allowed to warm to room temperature slowly overnight. The next morning, the reaction was quenched with 1M HCl (aq.), and extracted with DCM three times. The combines organics were dried over Na_2SO_4, filtered, concentrated, then purified *via* silica column flash chromatography (10% ethyl acetate in hexanes) to afford pure tosylate **SI-25** (1.0 g, 69% yield) as a thick colorless oil which solidifies upon cooling.

^{1}H NMR (400MHz, $CDCl_3$) δ 7.84 – 7.76 (m, 2H), 7.38 – 7.29 (m, 2H), 7.29 – 7.21 (m, 2H), 7.12 – 7.05 (m, 2H), 4.09 (t, *J* = 6.3 Hz, 2H), 2.44 (s, 3H), 2.37 (t, *J* = 6.9 Hz, 2H), 2.33 (s, 3H), 1.83 (tt, *J* = 8.1, 6.0 Hz, 2H), 1.68 – 1.57 (m, 2H).

^{13}C NMR (101 MHz, $CDCl_3$) δ 144.8, 137.8, 133.2, 131.5, 129.9, 129.1, 128.0, 120.7, 88.2, 81.4, 70.2, 28.1, 24.7, 21.7, 21.5, 18.8.

FT-IR (neat film NaCl): 2951, 2922, 1509, 1355, 1188, 1172, 1097, 1019, 813, 689, 661, 552, 525 cm^{-1}.

HR-MS (ESI) m/z: [M+H]+ Calculated for $C_{20}H_{23}O_3S^+$: 343.1368; Found 343.1366.

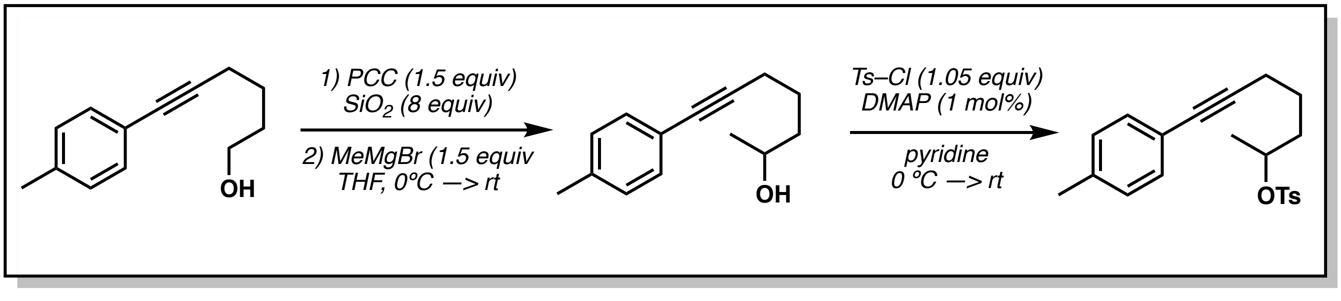

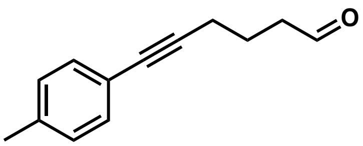

6-(*p*-tolyl)hex-5-ynal (SI-26)

Following a reported procedure.[20] To a flame-dried flask containing silica gel (2.0 g) and PCC (1.37g, 1.5 equiv, 6.37 mmol) was added dry DCM (50 mL). Then, a solution of alcohol **SI-24** (800 mg, 1.0 equiv, 4.25 mmol) dissolved in 10 mL dry DCM was added dropwise. The reaction flask was sealed and heated to 35 ºC overnight. The next morning, the reaction mixture was filtered through a pad of silica and washed through with DCM. The filtrate was concentrated, affording analytically pure (by NMR) material (**SI-26**) that matched reported literature[20] and was taken forward as is.

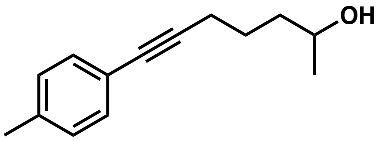

7-(*p*-tolyl)hept-6-yn-2-ol (SI-27)

Aldehyde **SI-26** (600 mg, 1.0 equiv, 3.22 mmol) was dissolved in 10 mL dry THF in a flame-dried Schlenk flask then cooled to 0 ºC. A solution of methylmagnesium bromide (1.6 mL, 1.5 equiv, 4.8 mmol) in THF (3 M solution) was added dropwise. After warming to room temperature for 30 minutes, the reaction was complete by TLC analysis and was quenched with saturated ammonium chloride. The mixture was extracted with diethyl ether three times, and the combined organics were dried over Na_2SO_4, filtered, and concentrated. Purification via silica gel flash chromatography (20% ethyl acetate in hexanes) afforded pure alcohol (**SI-27**) as a colorless oil (460 mg, 71% yield).

^{1}H NMR (400MHz, $CDCl_3$) δ 7.32 – 7.24 (m, 2H), 7.09 (ddt, *J* = 7.2, 1.5, 0.8 Hz, 2H), 3.88 (h, *J* = 6.1 Hz, 1H), 2.44 (t, *J* = 6.7 Hz, 2H), 2.33 (s, 3H), 1.82 – 1.55 (m, 4H), 1.23 (dd, *J* = 6.1, 0.7 Hz, 3H).

^{13}C NMR (101 MHz, $CDCl_3$) δ 137.5, 131.4, 128.9, 89.1, 80.9, 67.7, 38.4, 25.0, 23.6, 21.4, 19.4.

FT-IR (neat film NaCl): 3351, 2964, 2924, 2886, 1509, 1455, 1373, 1176, 1105, 1085, 979, 942, 816, 525 cm^{-1}.

HR-MS (ESI) m/z: [M+H]+ Calculated for $C_{14}H_{19}O^+$: 203.1430: Found 203.1439.

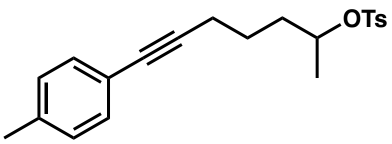

7-(*p*-tolyl)hept-6-yn-2-yl 4-methylbenzenesulfonate (SI-28)

Alcohol **SI-27** (385 mg, 1.0 equiv, 1.9 mmol) was dissolved in 1.2 mL dry pyridine (distilled over CaH_2) in an oven-dried dram vial equipped with a stir bar. The vial was cooled to 0 ºC, then DMAP (0.2 mg, .1 mol%, 0.002 mmol) was added followed by tosyl chloride (381 mg, 1.05 equiv, 2.0 mmol) as a solid. The mixture was stirred for 1 hour at 0 ºC then allowed to warm to room temperature overnight. The next morning, the mixture was filtered and diluted with cold diethyl ether and cold 4M HCl (aq). After vigorously shaking this mixture, the organic layer was removed and the aqueous layer was extracted with cold diethyl ether twice more. The combined organics were washed with cold 4M HCl twice more, then washed with water twice, then washed with brine. The organic layer was dried over Na_2SO_4, filtered, and concentrated. Purification *via* silica gel flash chromatography (10% ethyl acetate in hexanes) afforded tosylate **SI-28** as a colorless oil (435 mg, 64% yield).

^{1}H NMR (400MHz, $CDCl_3$) δ 7.74 – 7.67 (m, 2H), 7.21 (d, *J* = 8.0 Hz, 2H), 7.17 (m, 2H), 7.02 – 6.95 (m, 2H), 4.64 – 4.51 (m, 1H), 2.31 (s, 3H), 2.25 – 2.18 (m, 5H), 1.71 – 1.54 (m, 2H), 1.53 – 1.33 (m, 2H), 1.19 (d, *J* = 6.3 Hz, 3H).

^{13}C NMR (101 MHz, $CDCl_3$) δ 144.6, 137.7, 134.5, 131.5, 129.9, 129.1, 127.8, 120.8, 88.4, 81.3, 80.1, 35.7, 24.2, 21.7, 21.5, 21.0, 19.0.

FT-IR (neat film NaCl): 2935, 2868, 1598, 1509, 1453, 1354, 1188, 1174, 1098, 1043, 893, 816, 663, 577, 556 cm^{-1}.

HR-MS (ESI) m/z: [M+H]+ Calculated for $C_{21}H_{25}O_3S^+$: 357.1524 ; Found 357.1519.

5.1 – α-Vinylation of Silyl Ketene Acetals

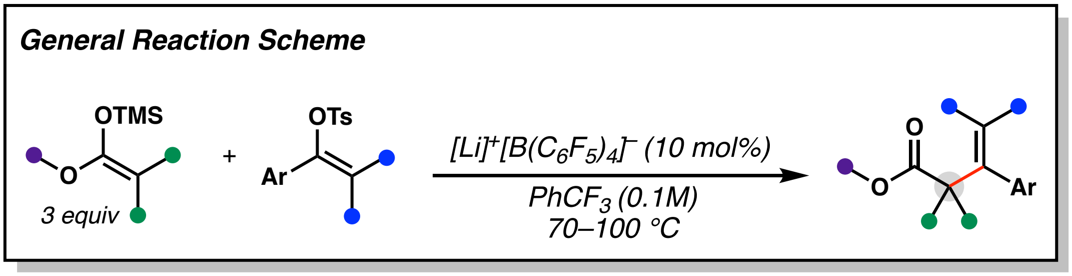

***General Procedure 1**:* All reactions were conducted in a well-maintained glove box (O_2, H_2O <0.5 ppm) on 0.2 mmol scale unless otherwise noted. To an oven dried dram vial with a magnetic stir bar was added $[Li]^+[B(C_6F_5)_4]^-$ (13.7 mg, 0.02 mmol, 0.1 equiv). To this was added trifluorotoluene (2 mL), and the corresponding silyl ketene acetal (3 equiv). Substrate (0.2 mmol, 1.0 equiv) was added and the reaction was allowed to stir at 80 °C in a metal heating block placed on an IKA hot plate for 12 hours (unless otherwise noted). The reactions were monitored by TLC, typically using 10% diethyl ether in hexanes for the mobile phase and stained with $KMnO_4$ (α-vinylation products are typically higher in R_f than the starting tosylate and are very distinguishable when stained with $KMnO_4$). Upon completion of reaction, the reaction mixture was removed from the glovebox and diluted with ether containing a drop of triethylamine. This was pushed through a plug of triethylamine treated silica gel in a pipette. The reaction mixture was concentrated in vacuo to give the crude material. The crude material was purified by silica flash chromatography on triethylamine treated silica gel (typically 100% hexanes with 0.1%TEA → 1% diethyl ether in hexanes with 0.1% TEA → 2% diethyl ether in hexanes with 0.1% TEA) and then dried on high vacuum to obtain material that is pure by ^1H NMR.

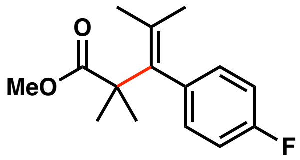

methyl 3-(4-fluorophenyl)-2,2,4-trimethylpent-3-enoate (3a)

Following General Procedure 1: To an oven dried dram vial with a magnetic stir bar was added $[Li]^+[B(C_6F_5)_4]^-$ (13.7 mg, 0.02 mmol, 0.1 equiv). To this was added trifluorotoluene (2 mL), and ((1-methoxy-2-methylprop-1-en-1-yl)oxy)trimethylsilane (105 mg, 0.60 mmol, 3 equiv). Vinyl tosylate **SI-4** (64.1 mg, 0.2 mmol, 1.0 equiv) was added and the reaction was allowed to stir at 80 °C in a metal heating block placed on an IKA hot plate for 12 hours. The reaction mixture was removed from the glovebox and diluted with ether containing a drop of triethylamine. This was pushed through a plug of triethylamine treated silica gel in a pipette and concentrated *in vacuo* to give the crude material. The crude material was purified by silica flash chromatography on triethylamine treated silica gel (2% diethyl ether in hexanes with 0.1% TEA) to give a colorless oil **3a** (21.0 mg, 42% yield).

^{1}H NMR (300 MHz, $CDCl_3$) δ 7.04 – 6.96 (m, 4H), 3.73 (s, 3H), 1.65 (s, 3H), 1.36 (s, 3H), 1.15 (s, 6H).

^{13}C NMR (126 MHz, $CDCl_3$) δ 179.6, 161.5 (d, *J* = 244.2 Hz), 138.3 (d, *J* = 3.4 Hz), 137.6, 131.1, 131.0 (d, *J* = 7.6 Hz), 114.9 (d, *J* = 21.0 Hz), 52.3, 45.9, 27.6, 23.9, 20.7.
^{19}F NMR (282 MHz, $CDCl_3$) δ -116.94.
FT-IR (neat film NaCl): 2976, 2948, 2873, 1731, 1601, 1506, 1251, 1220, 1138, 844, 584, 337.
HR-MS (ESI) m/z: [M+K]+ Calculated for $C_{15}H_{20}FO_2$ 251.1447; Found 251.1445.

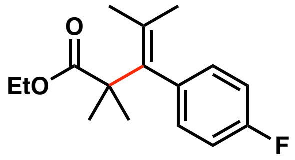

ethyl 3-(4-fluorophenyl)-2,2,4-trimethylpent-3-enoate (3b)
Following General Procedure 1: To an oven dried dram vial with a magnetic stir bar was added $[Li]^+[B(C_6F_5)_4]^-$ (13.7 mg, 0.02 mmol, 0.1 equiv). To this was added trifluorotoluene (2 mL), and the silyl ketene acetal **SI-17** (113 mg, 0.60 mmol, 3 equiv). Vinyl tosylate **SI-4** (64.1 mg, 0.2 mmol, 1.0 equiv) was added and the reaction was allowed to stir at 80 °C in metal heating block placed on an IKA hot plate for 12 hours. The reaction mixture was removed from the glovebox and diluted with ether containing a drop of triethylamine. This was pushed through a plug of triethylamine treated silica gel in a pipette and concentrated *in vacuo* to give the crude material. The crude material was purified by silica flash chromatography on triethylamine treated silica gel (2% diethyl ether in hexanes with 0.1% TEA) to give a colorless oil **3b** (26.5 mg, 50% yield).

^{1}H NMR (400 MHz, $CDCl_3$) δ 7.08 – 6.92 (m, 4H), 4.18 (q, *J* = 7.1 Hz, 2H), 1.66 (s, 3H), 1.35 (s, 3H), 1.29 (t, *J* = 7.1 Hz, 3H), 1.14 (s, 6H).
^{13}C NMR (101 MHz, $CDCl_3$) δ 179.0, 161.5 (d, *J* = 244.2 Hz), 138.5 (d, *J* = 3.6 Hz), 137.7, 131.0, 130.9 (d, *J* = 7.7 Hz), 114.8 (d, *J* = 21.0 Hz), 60.8, 45.9, 27.6, 23.9, 20.9, 14.4.
^{19}F NMR (282 MHz, $CDCl_3$) δ -117.1.
FT-IR (neat film NaCl): 2977, 2934, 2873, 1726, 1600, 1506, 1469, 1383, 1249, 1219, 1172, 1155, 1136, 1090, 1028, 857, 830, 810, 774, 733, 584, 538 cm^{-1}.
HR-MS (ESI) m/z: [M+H]+ $C_{16}H_{22}FO_2$ 265.1598; Found 265.1603.

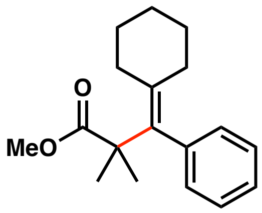

methyl 3-cyclohexylidene-2,2-dimethyl-3-phenylpropanoate (4)

Following General Procedure 1: To an oven dried dram vial with a magnetic stir bar was added $[Li]^+[B(C_6F_5)_4]^-$ (13.7 mg, 0.02 mmol, 0.1 equiv). To this was added trifluorotoluene (2 mL), and ((1-methoxy-2-methylprop-1-en-1-yl)oxy)trimethylsilane (105 mg, 0.60 mmol, 3 equiv). Vinyl tosylate **SI-2** (68.5 mg, 0.2 mmol, 1.0 equiv) was added and the reaction was allowed to stir at 80 °C in metal heating block placed on an IKA hot plate for 12 hours. The reaction mixture was removed from the glovebox and diluted with ether containing a drop of triethylamine. This was pushed through a plug of triethylamine treated silica gel in a pipette and concentrated *in vacuo* to give the crude material. The crude material was purified by silica flash chromatography on triethylamine treated silica gel (2% diethyl ether in hexanes with 0.1% TEA) to give a colorless oil **4** (43.5 mg, 80% yield).

^{1}H NMR (300 MHz, $CDCl_3$) δ 7.33 – 7.26 (m, 2H), 7.24 – 7.16 (m, 1H), 7.13 – 6.95 (m, 2H), 3.73 (s, 3H), 2.09 –2.07 (m, 2H), 1.74 – 1.68 (m, 2H), 1.57 – 1.44 (m, 4H), 1.40 – 1.32 (m, 2H), 1.15 (s, 6H).

^{13}C NMR (126 MHz, $CDCl_3$) δ 180.1, 142.1, 138.3, 135.6, 129.5, 127.9, 126.1, 52.2, 45.6, 33.8, 31.2, 28.4, 28.0, 27.6, 26.8.

FT-IR (neat film NaCl): 3054, 3018, 2974, 2924, 2852, 1728, 1457, 1443, 1249, 1137, 1129, 774, 761, 703 cm^{-1}.

HR-MS (ESI) m/z: [M+K]+ Calculated for $C_{18}H_{25}O_2$ 273.1855; Found 273.1846.

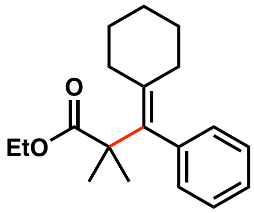

ethyl 3-cyclohexylidene-2,2-dimethyl-3-phenylpropanoate (5)

Following General Procedure 1: To an oven dried dram vial with a magnetic stir bar was added $[Li]^+[B(C_6F_5)_4]^-$ (13.7 mg, 0.02 mmol, 0.1 equiv). To this was added trifluorotoluene (2 mL), and the silyl ketene acetal **SI-17** (113 mg, 0.60 mmol, 3 equiv). Vinyl tosylate **SI-2** (68.5 mg, 0.2 mmol, 1.0 equiv) was added and the reaction was allowed to stir at 80 °C in metal heating block placed on an IKA hot plate for 12 hours. The reaction mixture was removed from the glovebox and diluted with ether containing a drop of triethylamine. This was pushed through a plug of triethylamine treated silica gel in a pipette and concentrated *in vacuo* to give the crude material.

The crude material was purified by silica flash chromatography on triethylamine treated silica gel (2% diethyl ether in hexanes with 0.1% TEA) to give a colorless oil **5** (47.0 mg, 82% yield).

^{1}H NMR (300 MHz, $CDCl_3$) δ 7.33 – 7.26 (m, 2H), 7.25 – 7.20 (m, 1H), 7.10 – 7.00 (m, 2H), 4.17 (q, J = 7.1 Hz, 2H), 2.11 (m, 2H), 1.78 – 1.66 (m, 2H), 1.58 – 1.45 (m, 4H), 1.42 – 1.34 (q, J = 6.1 Hz, 2H), 1.30 (t, J = 7.1 Hz, 3H), 1.15 (s, 6H).
^{13}C NMR (126 MHz, $CDCl_3$) δ 179.5, 142.3, 138.2, 135.8, 129.5, 127.9, 126.1, 60.7, 45.6, 33.8, 31.3, 28.4, 28.1, 27.6, 26.8, 14.4.
FT-IR (neat film NaCl): 3054, 2975, 2925, 2852, 1725, 1489, 1468, 1444, 1383, 1363, 1293, 1248, 1171, 1155, 1137, 1030, 853, 774, 760, 704, 531, 406 cm^{-1}.
HR-MS (ESI) m/z: [M+Na]+ Calculated for $C_{19}H_{26}NaO_2$ 309.1825; Found 309.1818.

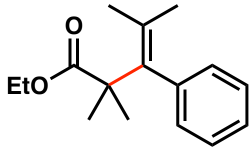

ethyl 2,2,4-trimethyl-3-phenylpent-3-enoate (6)
Following General Procedure 1: To an oven dried dram vial with a magnetic stir bar was added $[Li]^+[B(C_6F_5)_4]^-$ (13.7 mg, 0.02 mmol, 0.1 equiv). To this was added trifluorotoluene (2 mL), and the silyl ketene acetal **SI-17** (113 mg, 0.60 mmol, 3 equiv). Vinyl tosylate **SI-1** (60.5 mg, 0.2 mmol, 1.0 equiv) was added and the reaction was allowed to stir at 80 °C in metal heating block placed on an IKA hot plate for 12 hours. The reaction mixture was removed from the glovebox and diluted with ether containing a drop of triethylamine. This was pushed through a plug of triethylamine treated silica gel in a pipette and concentrated *in vacuo* to give the crude material. The crude material was purified by silica flash chromatography on triethylamine treated silica gel (2% diethyl ether in hexanes with 0.1% TEA) to give a colorless oil **6** (35.0 mg, 72% yield).

^{1}H NMR (300 MHz, $CDCl_3$) δ 7.33 – 7.27 (m, 2H), 7.25 – 7.20 (m, 1H), 7.08 – 7.01 (m, 2H), 4.19 (q, J = 7.1 Hz, 2H), 1.67 (s, 3H), 1.36 (s, 3H), 1.29 (t, J = 7.1 Hz, 3H), 1.16 (s, 6H).
^{13}C NMR (126 MHz, $CDCl_3$) δ 179.2, 142.7, 138.7, 130.1, 129.5, 128.0, 126.1, 60.7, 45.9, 27.6, 23.9, 20.9, 14.4.
FT-IR (neat film NaCl): 3055, 2977, 2933, 2872, 1727, 1490, 1469, 1443, 1383, 1362, 1249, 1137, 1085, 1029, 933, 862, 775, 764, 703, 632, 455 cm^{-1}.
HR-MS (ESI) m/z: [M+H]+ Calculated for $C_{16}H_{23}O_2$ 247.1693; Found 247.1701.

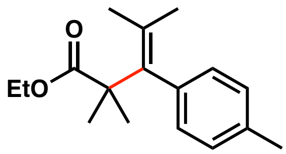

ethyl 2,2,4-trimethyl-3-(*p*-tolyl)pent-3-enoate (7)

Following General Procedure 1 with slight modifications; performed on 1.0 mmol scale: To a flame dried 50 mL Schlenk flask with a magnetic stirbar which was brough inside a glovebox, was added $[Li]^+[B(C_6F_5)_4]^-$ (68.6 mg, 0.10 mmol, 0.1 equiv). To this was added trifluorotoluene (10 mL), and the silyl ketene acetal **SI-17** (565 mg, 3.0 mmol, 3 equiv). Vinyl tosylate **SI-6** (316 mg, 1.0 mmol, 1.0 equiv) was added. The reaction was then sealed with a glass stopper and heated outside the glovebox at 80 ºC in an oil bath for 12 hours. The reaction mixture was then cooled to room temperature and diluted with ether containing 1% triethylamine. This was pushed through a small plug of triethylamine treated silica gel and concentrated *in vacuo* to give the crude material. The crude material was purified by silica flash chromatography on triethylamine treated silica gel (3% diethyl ether in hexanes with 0.1% TEA) to give a colorless oil **7** (200 mg, 78% yield).

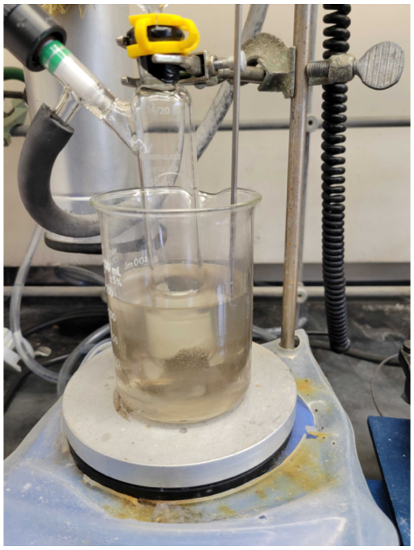

^{1}H NMR (400 MHz, $CDCl_3$) δ 7.14 – 7.08 (m, 2H), 6.96 – 6.88 (m, 2H), 4.18 (q, *J* = 7.1 Hz, 2H), 2.35 (s, 3H), 1.66 (s, 3H), 1.36 (s, 3H), 1.29 (t, *J* = 7.1 Hz, 3H), 1.15 (s, 6H).

^{13}C NMR (101 MHz, $CDCl_3$) δ 179.3, 139.6, 138.5, 135.5, 130.1, 129.4, 128.6, 60.7, 45.9, 27.6, 23.9, 21.3, 20.9, 14.4.

FT-IR (neat film NaCl): 2976, 2931, 2871, 1727, 1510, 1468, 1446, 1382, 1248, 1136, 1028, 932, 856, 815, 731, 529, 485 cm^{-1}.

HR-MS (ESI) m/z: [M+H]+ Calculated for $C_{17}H_{25}O_2$ 261.1849; Found 261.1862.

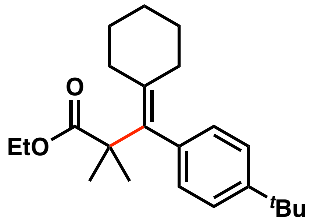

ethyl 3-(4-(*tert*-butyl)phenyl)-3-cyclohexylidene-2,2-dimethylpropanoate (8)

Following General Procedure 1: To an oven dried dram vial with a magnetic stir bar was added $[Li]^+[B(C_6F_5)_4]^-$ (13.7 mg, 0.02 mmol, 0.1 equiv). To this was added trifluorotoluene (2 mL), and the silyl ketene acetal **SI-17** (113 mg, 0.60 mmol, 3 equiv). Vinyl tosylate **SI-13** (79.7 mg, 0.2 mmol, 1.0 equiv) was added and the reaction was allowed to stir at 80 °C in a metal heating block placed on an IKA hot plate for 12 hours. The reaction mixture was removed from the glovebox and diluted with ether containing a drop of triethylamine. This was pushed through a plug of triethylamine treated silica gel in a pipette and concentrated *in vacuo* to give the crude material. The crude material was purified by silica flash chromatography on triethylamine treated silica gel (2% diethyl ether in hexanes with 0.1% TEA) to give a colorless oil **8** (62.0 mg, 91% yield).

^{1}H NMR (400 MHz, $CDCl_3$) δ 7.28 (d, *J* = 8.1 Hz, 3H), 6.95 (d, *J* = 7.9 Hz, 2H), 4.16 (q, *J* = 7.1 Hz, 2H), 2.09 (t, *J* = 5.6 Hz, 2H), 1.75 – 1.71 (m, 2H), 1.51 (dd, *J* = 8.4, 3.2 Hz, 3H), 1.37 (q, *J* = 5.9 Hz, 2H), 1.32 (s, 9H), 1.29 (t, *J* = 7.1 Hz, 4H), 1.14 (s, 6H).

^{13}C NMR (101 MHz, $CDCl_3$) δ 179.7, 148.7, 139.1, 138.0, 135.7, 129.0, 124.6, 60.7, 45.7, 34.5, 33.8, 31.6, 31.3, 28.5, 28.1, 27.6, 26.8, 14.4.

FT-IR (neat film NaCl): 3023, 2967, 2927, 2853, 1726, 1507, 1467, 1446, 1383, 1363, 1267, 1249, 1138, 1112, 1029, 853, 834, 805, 574, 409 cm^{-1}.

HR-MS (ESI) m/z: [M+H]+ Calculated for $C_{23}H_{35}O_2$ 343.2632; Found 343.2633.

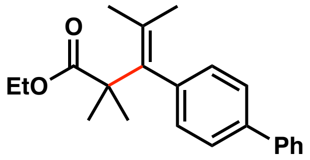

ethyl 3-([1,1'-biphenyl]-4-yl)-3-cyclohexylidene-2,2-dimethylpropanoate (9)

Following General Procedure 1: To an oven dried dram vial with a magnetic stir bar was added $[Li]^+[B(C_6F_5)_4]^-$ (13.7 mg, 0.02 mmol, 0.1 equiv). To this was added trifluorotoluene (2 mL), and the silyl ketene acetal **SI-17** (113 mg, 0.60 mmol, 3 equiv). Vinyl tosylate **SI-10** (75.7 mg, 0.2 mmol, 1.0 equiv) was added and the reaction was allowed to stir at 80 °C in a metal heating

block placed on an IKA hot plate for 12 hours. The reaction mixture was removed from the glovebox and diluted with ether containing a drop of triethylamine. This was pushed through a plug of triethylamine treated silica gel in a pipette and concentrated *in vacuo* to give the crude material. The crude material was purified by silica flash chromatography on triethylamine treated silica gel (2% diethyl ether in hexanes with 0.1% TEA) to give a colorless oil **9** (56.0 mg, 87% yield).

^{1}H NMR (400 MHz, $CDCl_3$) δ 7.64 – 7.61 (m, 2H), 7.58 – 7.54 (m, 2H), 7.44 (t, *J* = 7.7 Hz, 2H), 7.33 (td, *J* = 7.2, 1.3 Hz, 1H), 7.15 – 7.09 (m, 2H), 4.21 (q, *J* = 7.1 Hz, 2H), 1.70 (s, 3H), 1.42 (s, 3H), 1.31 (t, *J* = 7.1 Hz, 3H), 1.20 (s, 6H).
^{13}C NMR (101 MHz, $CDCl_3$) δ 179.2, 141.8, 141.1, 138.9, 138.3, 130.4, 130.0, 128.9, 127.2, 127.1, 126.6, 60.8, 45.9, 27.7, 24.0, 20.9, 14.6.
FT-IR (neat film NaCl): 3056, 3026, 2976, 2933, 2908, 2872, 1725, 1600, 1485, 1468, 1447, 1383, 1249, 1136, 1028, 1008, 933, 859, 767, 737, 697, 567, 435, 409 cm^{-1}.
HR-MS (ESI) m/z: [M+H]+ Calculated for $C_{22}H_{27}O_2$ 323.2006; Found 323.2017.

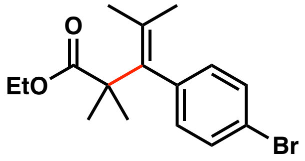

ethyl 3-(4-bromophenyl)-2,2,4-trimethylpent-3-enoate (10)
Following General Procedure 1: To an oven dried dram vial with a magnetic stir bar was added $[Li]^+[B(C_6F_5)_4]^-$ (13.7 mg, 0.02 mmol, 0.1 equiv). To this was added trifluorotoluene (2 mL), and the silyl ketene acetal **SI-17** (113 mg, 0.60 mmol, 3 equiv). Vinyl tosylate **SI-7** (76.3 mg, 0.2 mmol, 1.0 equiv) was added and the reaction was allowed to stir at 100 °C in a metal heating block placed on an IKA hot plate for 12 hours. The reaction mixture was removed from the glovebox and diluted with ether containing a drop of triethylamine. This was pushed through a plug of triethylamine treated silica gel in a pipette and concentrated *in vacuo* to give the crude material. The crude material was purified by silica flash chromatography on triethylamine treated silica gel (2% diethyl ether in hexanes with 0.1% TEA) to give a colorless oil **10** (27.0 mg, 42% yield).
^{1}H NMR (300 MHz, $CDCl_3$) δ 7.44 (dd, *J* = 8.4, 1.7 Hz, 2H), 6.93 (dd, *J* = 8.2, 1.8 Hz, 2H), 4.18 (qd, *J* = 7.1, 1.4 Hz, 2H), 1.66 (s, 3H), 1.35 (s, 3H), 1.29 (dd, *J* = 7.7, 6.1 Hz, 3H), 1.14 (d, *J* = 1.8 Hz, 6H).

^{13}C NMR (126 MHz, $CDCl_3$) δ 178.9, 141.6, 137.5, 131.3, 131.2, 131.0, 120.2, 60.8, 45.7, 27.6, 23.9, 20.9, 14.4.

FT-IR (neat film NaCl): 2976, 2932, 2872, 1726, 1483, 1469, 1383, 1248, 1136, 1028, 1012, 932, 819, 731, 689, 523, 419 cm^{-1}.

HR-MS (ESI) m/z: [M+H]+ Calculated for $C_{16}H_{22}BrO_2$ 325.0798; Found 325.0802.

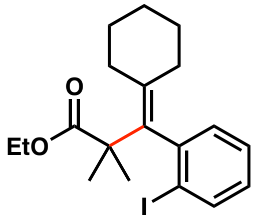

ethyl 3-cyclohexylidene-3-(2-iodophenyl)-2,2-dimethylpropanoate (11)

Following General Procedure 1: To an oven dried dram vial with a magnetic stir bar was added $[Li]^+[B(C_6F_5)_4]^-$ (13.7 mg, 0.02 mmol, 0.1 equiv). To this was added trifluorotoluene (2 mL), and the silyl ketene acetal **SI-17** (113 mg, 0.60 mmol, 3 equiv). Vinyl tosylate **SI-16** (93.7 mg, 0.2 mmol, 1.0 equiv) was added and the reaction was allowed to stir at 80 °C in a metal heating block placed on an IKA hot plate for 12 hours. The reaction mixture was removed from the glovebox and diluted with ether containing a drop of triethylamine. This was pushed through a plug of triethylamine treated silica gel in a pipette and concentrated *in vacuo* to give the crude material. The crude material was purified by silica flash chromatography on triethylamine treated silica gel (2% diethyl ether in hexanes with 0.1% TEA) to give a colorless oil **11** (56.7 mg, 69% yield).

^{1}H NMR (400 MHz, $CDCl_3$) δ 7.85 (dd, *J* = 8.0, 1.2 Hz, 1H), 7.29 (td, *J* = 7.4, 1.2 Hz, 1H), 7.22 (dd, *J* = 7.7, 1.9 Hz, 1H), 6.97 – 6.85 (m, 1H), 4.22 – 4.15 (m, 2H), 2.16 (dqt, *J* = 10.2, 6.9, 3.8 Hz, 2H), 1.77 – 1.57 (m, 5H), 1.51 (m 6H), 1.33 (d, *J* = 7.1 Hz, 3H), 1.06 (s, 3H). **note: one of the methyl peaks is buried with other peaks at 1.51 ppm.*

^{13}C NMR (101 MHz, $CDCl_3$) δ 179.2, 147.6, 139.8, 139.0, 136.6, 130.8, 127.9, 127.7, 102.0, 60.9, 45.8, 33.7, 31.4, 28.6, 27.70, 27.68, 27.2, 26.7, 14.5.

FT-IR (neat film NaCl): 3056, 2978, 2927, 2853, 1724, 1635, 1461, 1445, 1384, 1244, 1138, 1030, 1013, 856, 756, 733, cm^{-1}.

HR-MS (ESI) m/z: [M+H]+ Calculated for $C_{19}H_{26}IO_2$ 413.0972; Found 413.0986.

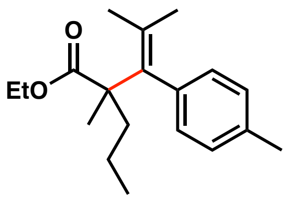

ethyl 2,4-dimethyl-2-propyl-3-(*p*-tolyl)pent-3-enoate (12)

Following General Procedure 1: To an oven dried dram vial with a magnetic stir bar was added $[Li]^+[B(C_6F_5)_4]^-$ (13.7 mg, 0.02 mmol, 0.1 equiv). To this was added trifluorotoluene (2 mL), and the silyl ketene acetal **SI-18** (130 mg, 0.60 mmol, 3 equiv). Vinyl tosylate **SI-6** (63.3 mg, 0.2 mmol, 1.0 equiv) was added and the reaction was allowed to stir at 80 °C in a metal heating block placed on an IKA hot plate for 12 hours. The reaction mixture was removed from the glovebox and diluted with ether containing a drop of triethylamine. This was pushed through a plug of triethylamine treated silica gel in a pipette and concentrated *in vacuo* to give the crude material. The crude material was purified by silica flash chromatography on triethylamine treated silica gel (3.5% diethyl ether in hexanes with 0.5% TEA) to give a colorless oil **12** (40.5 mg, 70% yield).

^{1}H NMR (400 MHz, $CDCl_3$) δ 7.09 (dddd, *J* = 6.9, 2.8, 1.9, 1.0 Hz, 2H), 6.96 – 6.89 (m, 2H), 4.17 (qd, *J* = 7.2, 0.7 Hz, 2H), 2.35 (s, 3H), 1.67 (s, 3H), 1.55 – 1.47 (m, 1H), 1.33 (s, 3H), 1.32 – 1.24 (m, 5H), 1.20 (s, 3H), 1.13 – 1.04 (m, 1H), 0.79 (t, *J* = 7.2 Hz, 3H).
^{13}C NMR (101 MHz, $CDCl_3$) δ 178.5, 139.8, 138.4, 135.4, 130.4, 129.8, 129.6, 128.6, 128.5, 60.5, 49.5, 42.3, 24.4, 24.2, 21.3, 22.0, 18.0, 14.9, 14.4.
FT-IR (neat film NaCl): 2961, 2933, 2872, 1726, 1510, 1456, 1374, 1303, 1216, 1138, 1039, 816 cm^{-1}.
HR-MS (ESI) m/z: [M+H]+ Calculated for $C_{19}H_{29}O_2$ 289.2162; Found 289.2170.

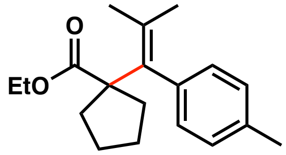

ethyl 1-(2-methyl-1-(*p*-tolyl)prop-1-en-1-yl)cyclopentane-1-carboxylate (13)

Following General Procedure 1: To an oven dried dram vial with a magnetic stir bar was added $[Li]^+[B(C_6F_5)_4]^-$ (13.7 mg, 0.02 mmol, 0.1 equiv). To this was added trifluorotoluene (2 mL), and the silyl ketene acetal **SI-21** (130 mg, 0.60 mmol, 3 equiv). Vinyl tosylate **SI-6** (63.3 mg, 0.2 mmol, 1.0 equiv) was added and the reaction was allowed to stir at 80 °C in a metal heating

block placed on an IKA hot plate for 12 hours. The reaction mixture was removed from the glovebox and diluted with ether containing a drop of triethylamine. This was pushed through a plug of triethylamine treated silica gel in a pipette and concentrated *in vacuo* to give the crude material. The crude material was purified by silica flash chromatography on triethylamine treated silica gel (3% diethyl ether in hexanes with 0.5% TEA) to give a colorless oil **13** (23.0 mg, 40% yield).

^{1}H NMR (400 MHz, $CDCl_3$) δ 7.14 – 7.06 (m, 2H), 6.98 (d, *J* = 8.0 Hz, 2H), 4.18 (q, *J* = 7.1 Hz, 2H), 2.35 (s, 3H), 2.22 – 2.14 (m, 2H), 1.72 (s, 3H), 1.53 – 1.48 (m, 4H), 1.37 (s, 3H), 1.29 (t, *J* = 7.1 Hz, 3H).

^{13}C NMR (101 MHz, $CDCl_3$) δ 178.1, 140.4, 138.9, 135.4, 131.1, 129.4, 128.6, 60.7, 57.9, 38.2, 24.5, 23.9, 21.4, 21.3, 14.4.

FT-IR (neat film NaCl): 2954, 2871, 1722, 1509, 1450, 1384, 1365, 1321, 1230, 1175, 1160, 1105, 1031, 860,. 814, 585, 533 cm^{-1}.

HR-MS (ESI) m/z: [M+H]+ Calculated for $C_{19}H_{27}O_2$ 287.2006; Found 287.2003.

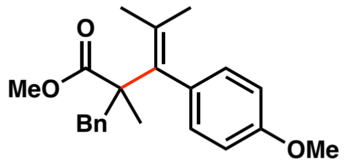

methyl 2-benzyl-3-(4-methoxyphenyl)-2,4-dimethylpent-3-enoate (14)

Following General Procedure 1: To an oven dried dram vial with a magnetic stir bar was added $[Li]^+[B(C_6F_5)_4]^-$ (13.7 mg, 0.02 mmol, 0.1 equiv). To this was added trifluorotoluene (2 mL), and the silyl ketene acetal **SI-23** (150 mg, 0.60 mmol, 3 equiv). Vinyl tosylate **SI-11** (66.5 mg, 0.2 mmol, 1.0 equiv) was added and the reaction was allowed to stir at 80 °C in a metal heating block placed on an IKA hot plate for 12 hours. The reaction mixture was removed from the glovebox and diluted with ether containing a drop of triethylamine. This was pushed through a plug of triethylamine treated silica gel in a pipette and concentrated *in vacuo* to give the crude material. The crude material was purified by silica flash chromatography on triethylamine treated silica gel (4% ethyl acetate in hexanes with 0.5% TEA) to give a colorless oil **14** (47.0 mg, 69% yield).

^{1}H NMR (400 MHz, $CDCl_3$) δ 7.25 – 7.19 (m, 3H), 7.06 – 7.02 (m, 2H), 6.97 (dd, *J* = 8.4, 2.2 Hz, 1H), 6.81 (dd, *J* = 8.4, 2.8 Hz, 1H), 6.66 (dd, *J* = 8.4, 2.8 Hz, 1H), 6.21 (dd, *J* = 8.4, 2.2 Hz, 1H), 3.76 (s, 3H), 3.71 (s, 3H), 2.92 (d, *J* = 13.1 Hz, 1H), 2.83 (d, *J* = 13.2 Hz, 1H), 1.71 (s, 3H), 1.38 (s, 3H), 1.14 (s, 3H).

^{13}C NMR (101 MHz, $CDCl_3$) δ 178.5, 157.7, 138.0, 136.7, 134.9, 131.8, 131.0, 130.8, 130.3, 127.8, 126.5, 113.3, 112.9, 55.2, 52.0, 50.8, 45.1, 24.9, 24.2, 21.0.
FT-IR (neat film NaCl): 3085, 3029, 2993, 2934, 2836, 1726, 1606, 1507, 1454, 1372, 1284, 1242, 1202, 1175, 1103, 1035, 909, 849, 829, 743, 701, 598, 548 cm^{-1}.
HR-MS (ESI) m/z: [M+H]+ Calculated for $C_{22}H_{27}O_3$ 339.1955; Found 339.1960.

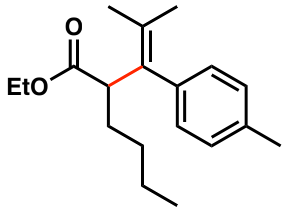

ethyl 2-(2-methyl-1-(*p*-tolyl)prop-1-en-1-yl)hexanoate (15)
Following General Procedure 1: To an oven dried dram vial with a magnetic stir bar was added $[Li]^+[B(C_6F_5)_4]^-$ (13.7 mg, 0.02 mmol, 0.1 equiv). To this was added trifluorotoluene (2 mL), and the silyl ketene acetal **SI-19** (130 mg, 0.60 mmol, 3 equiv). Vinyl tosylate **SI-6** (63.3 mg, 0.2 mmol, 1.0 equiv) was added and the reaction was allowed to stir at 80 °C in a metal heating block placed on an IKA hot plate for 12 hours. The reaction mixture was removed from the glovebox and diluted with ether containing a drop of triethylamine. This was pushed through a plug of triethylamine treated silica gel in a pipette and concentrated *in vacuo* to give the crude material. The crude material was purified by silica flash chromatography on triethylamine treated silica gel (1% ethyl acetate in hexanes with 0.5% TEA) to give a colorless oil **15** (43.0 mg, 75% yield).

^{1}H NMR (400 MHz, $CDCl_3$) δ 7.14 – 7.01 (m, 2H), 6.95 – 6.72 (m, 2H), 4.07 (qd, J = 7.1, 1.5 Hz, 2H), 3.65 (t, J = 7.4 Hz, 1H), 2.32 (s, 3H), 1.88 (s, 3H), 1.72 – 1.63 (m, 1H), 1.48 (s, 3H), 1.42 – 1.33 (m, 1H), 1.31 – 1.24 (m, 4H), 1.21 (t, J = 7.1 Hz, 3H), 0.89 – 0.83 (m, 3H).
^{13}C NMR (101 MHz, $CDCl_3$) δ 174.2, 137.8, 135.8, 132.8, 131.8, 129.5, 128.6, 60.3, 48.6, 30.0, 29.97, 22.9, 22.8, 21.3, 20.5, 14.4, 14.2.
FT-IR (neat film NaCl): 2956, 2927, 2860, 1732, 1510, 1446, 1367, 1217, 1175, 1128, 1112, 1032, 817, 728, 568 cm^{-1}.
HR-MS (ESI) m/z: [M+Na]+ Calculated for $C_{19}H_{28}NaO_2$ 311.1982; Found 311.1984.

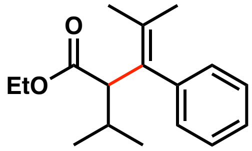

ethyl 2-isopropyl-4-methyl-3-phenylpent-3-enoate (16)

Following General Procedure 1: To an oven dried dram vial with a magnetic stir bar was added $[Li]^+[B(C_6F_5)_4]^-$ (13.7 mg, 0.02 mmol, 0.1 equiv). To this was added trifluorotoluene (2 mL), and the silyl ketene acetal **SI-20** (121 mg, 0.60 mmol, 3 equiv). Vinyl tosylate **SI-1** (60.5 mg, 0.2 mmol, 1.0 equiv) was added and the reaction was allowed to stir at 80 °C in a metal heating block placed on an IKA hot plate for 12 hours. The reaction mixture was removed from the glovebox and diluted with ether containing a drop of triethylamine. This was pushed through a plug of triethylamine treated silica gel in a pipette and concentrated *in vacuo* to give the crude material. The crude material was purified by silica flash chromatography on triethylamine treated silica gel (3% diethyl ether in hexanes with 0.5% TEA) to give a colorless oil **16** (30.0 mg, 58% yield).

^{1}H NMR (400 MHz, C_6D_6) δ 7.25 – 7.18 (m, 4H), 7.13 – 7.08 (m, 1H), 3.95 (q, *J* = 7.1 Hz, 2H), 3.51 (d, *J* = 11.0 Hz, 1H), 2.20 (dp, *J* = 10.9, 6.5 Hz, 1H), 1.83 (s, 3H), 1.47 (s, 3H), 1.01 (d, *J* = 6.4 Hz, 3H), 0.93 (t, *J* = 7.1 Hz, 3H), 0.91 (d, *J* = 6.7 Hz, 3H).
^{13}C NMR (101 MHz, $CDCl_3$) δ 173.5, 141.1, 133.5, 132.0, 129.8, 127.8, 126.4, 77.4, 60.1, 57.1, 29.9, 28.1, 23.2, 21.5, 20.9, 20.6, 14.3.
FT-IR (neat film NaCl): 2959, 2927, 2870, 1735, 1366, 1278, 1234, 1178, 1120, 1033, 702 cm^{-1}.
HR-MS (ESI) m/z: [M+H]+ Calculated for $C_{17}H_{25}O_2$ 261.1849; Found 261.1845.

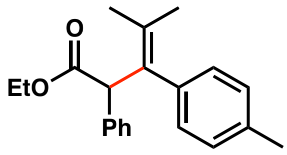

ethyl 4-methyl-2-phenyl-3-(*p*-tolyl)pent-3-enoate (17)

Following General Procedure 1: To an oven dried dram vial with a magnetic stir bar was added $[Li]^+[B(C_6F_5)_4]^-$ (13.7 mg, 0.02 mmol, 0.1 equiv). To this was added trifluorotoluene (2 mL), and the silyl ketene acetal **SI-22** (142 mg, 0.60 mmol, 3 equiv). Vinyl tosylate **SI-6** (63.3 mg, 0.2 mmol, 1.0 equiv) was added and the reaction was allowed to stir at 80 °C in a metal heating block placed on an IKA hot plate for 12 hours. The reaction mixture was removed from the glovebox and diluted with ether containing a drop of triethylamine. This was pushed through a

plug of triethylamine treated silica gel in a pipette and concentrated *in vacuo* to give the crude material. The crude material was purified by silica flash chromatography on triethylamine treated silica gel (3% diethyl ether in hexanes with 0.5% TEA) to give a colorless oil **17** (17.0 mg, 27% yield).

^{1}H NMR (400 MHz, $CDCl_3$) 7.25 – 7.17 (m, 3H), 7.17 – 7.11 (m, 2H), 7.01 – 6.96 (m, 2H), 6.84 – 6.71 (m, 2H), 5.05 (s, 1H), 4.08 (q, *J* = 7.1 Hz, 2H), 2.28 (s, 3H), 1.88 (s, 3H), 1.56 (s, 3H), 1.17 (t, *J* = 7.1 Hz, 3H).
^{13}C NMR (101 MHz, $CDCl_3$) δ 172.7, 138.2, 137.8, 135.7, 132.8, 132.3, 129.9, 129.4, 128.3, 128.0, 126.7, 60.8, 55.2, 23.0, 21.2, 20.9, 14.2.
FT-IR (neat film NaCl): 2959, 2927, 2870, 1735, 1366, 1278, 1234, 1178, 1120, 1033, 702 cm^{-1}.
HR-MS (ESI) m/z: [M+H]+ Calculated for $C_{21}H_{25}O_2$ 309.1849; Found 309.1855.

5.2 – Failed Substrate Class

The authors thought it would be beneficial to the reader to be aware of a type of vinyl tosylate substrate possessing two phenyl groups on the C–C double bond that resulted in an intractable product mixture. Although the reaction consumed the starting vinyl tosylate, the crude NMR was considerably more messy than other reactions discussed in this manuscript, and appears to possess oligomeric compounds as noted by the broad peaks throughout the spectra. The crude NMR is displayed on pg 124 in Section 8 of the Supplementary Materials.

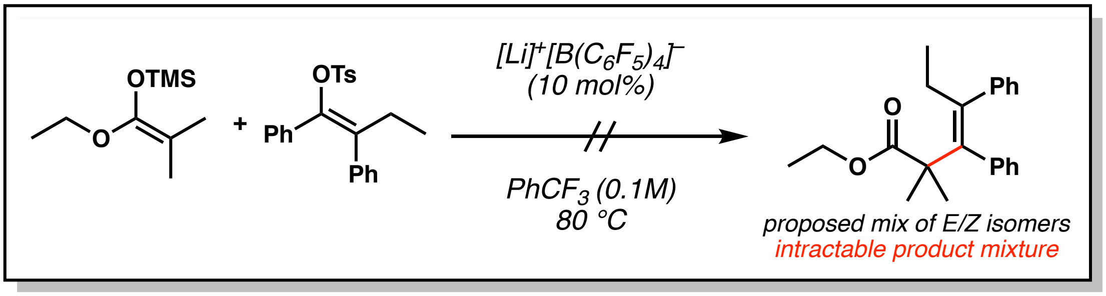

5.3 – Cyclization cascade reactions:

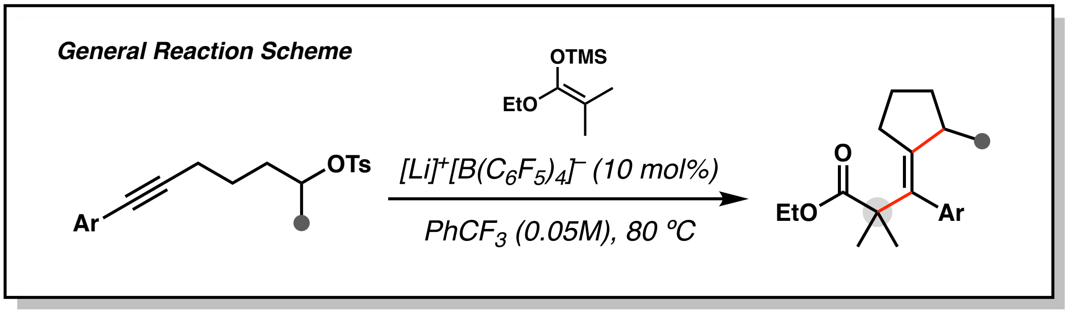

***General Procedure 2**:* All reactions were conducted in a well-maintained glove box (O_2, H_2O <0.5 ppm) on 0.2 mmol scale unless otherwise noted. To an oven dried dram vial with a magnetic stir bar was added $[Li]^+[B(C_6F_5)_4]^-$ (13.7 mg, 0.02 mmol, 0.1 equiv). To this was added trifluorotoluene (4 mL), and the corresponding silyl ketene acetal (3 equiv). Substrate (0.2 mmol, 1.0 equiv) was added and the reaction was allowed to stir at 80 °C in a metal heating block placed on an IKA hot plate for 24 hours. The reactions were monitored by TLC, typically using 10% diethyl ether in hexanes for the mobile phase (α-vinylation products are typically higher in R_f than the starting tosylate). Upon completion of reaction, the reaction mixture was removed from the glovebox and diluted with ether containing a drop of triethylamine. This was pushed through a plug of triethylamine treated silica gel in a pipette. The reaction mixture was concentrated *in vacuo* to give the crude material, which was purified by silica flash chromatography on triethylamine treated silica gel (typically 1–2% diethyl ether in hexanes with 0.1% triethylamine) and then dried on high vacuum to obtain material that is pure by ^{1}H NMR.

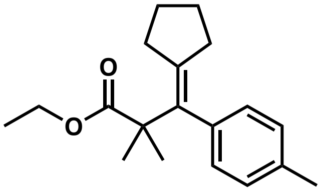

ethyl 3-cyclopentylidene-2,2-dimethyl-3-(*p*-tolyl)propanoate (20)

Following General Procedure 2: To an oven dried dram vial with a magnetic stir bar was added $[Li]^+[B(C_6F_5)_4]^-$ (13.7 mg, 0.02 mmol, 0.1 equiv). To this was added trifluorotoluene (4 mL), and the silyl ketene acetal **SI-17** (113.0 mg, 3 equiv, .6 mmol). Alkyl tosylate **SI-25** (68.5 mg, 1.0 equiv, 0.2 mmol) was added and the reaction was allowed to stir at 80 °C in a metal heating block placed on an IKA hot plate for 24 hours. The reaction mixture was removed from the glovebox and diluted with ether containing a drop of triethylamine. This was pushed through a plug of triethylamine treated silica gel in a pipette and concentrated *in vacuo* to give the crude material. The crude

material was purified by silica flash chromatography on triethylamine treated silica gel (2% diethyl ether in hexanes with 0.1% TEA) to give a colorless oil **20** (31.9 mg, 56% yield).

^{1}H NMR (400 MHz, $CDCl_3$) δ 7.15 – 7.08 (m, 2H), 7.00 – 6.92 (m, 2H), 4.17 (q, *J* = 7.1 Hz, 2H), 2.34 (s, 3H), 2.16 (tt, *J* = 7.1, 1.4 Hz, 2H), 1.85 (tt, *J* = 7.4, 1.2 Hz, 2H), 1.69 – 1.57 (m, 2H), 1.51 – 1.40 (m, 2H), 1.28 (t, *J* = 7.1 Hz, 3H), 1.18 (s, 6H).
^{13}C NMR (101 MHz, $CDCl_3$) δ 178.5, 141.8, 140.0, 135.6, 135.5, 129.0, 128.8, 60.6, 46.5, 34.0, 30.4, 27.5, 26.6, 25.8, 21.3, 14.3.
FT-IR (neat film NaCl): 2971, 2953, 2867, 1726, 1509, 1466, 1382, 1248, 1134, 1030, 806 cm^{-1}.
HR-MS (ESI) m/z: [M+H]+ Calculated for $C_{19}H_{27}O_2^+$: 287.2006 ; Found 287.2013.

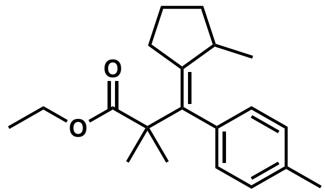

Ethyl 2,2-dimethyl-3-(2-methylcyclopentylidene)-3-(*p*-tolyl)propanoate (21)
Following General Procedure 2: To an oven dried dram vial with a magnetic stir bar was added $[Li]^+[B(C_6F_5)_4]^-$ (13.7 mg, 0.02 mmol, 0.1 equiv). To this was added trifluorotoluene (4 mL), and the silyl ketene acetal **SI-17** (113.0 mg, 3 equiv, .6 mmol). Alkyl tosylate **SI-28** (71.3 mg, 0.2 mmol, 1.0 equiv) was added and the reaction was allowed to stir at 80 °C in a metal heating block placed on an IKA hot plate for 24 hours. The reaction mixture was removed from the glovebox and diluted with ether containing a drop of triethylamine. This was pushed through a plug of triethylamine treated silica gel in a pipette and concentrated *in vacuo* to give the crude material. The crude material was purified by silica flash chromatography on triethylamine treated silica gel (1% → 2% diethyl ether in hexanes with 0.1% TEA) to give a colorless oil **21** (42.1 mg, 70% yield). *The olefin isomer (E) was assigned on the basis of NOESY NMR.

^{1}H NMR (400 MHz, $CDCl_3$) δ 7.14 – 7.06 (m, 2H), 6.98 (ddd, *J* = 11.9, 7.4, 1.5 Hz, 2H), 4.24 – 4.08 (m, 2H), 2.35 (s, 3H), 2.32 (s, 1H), 2.25 – 2.09 (m, 2H), 1.79 – 1.68 (m, 1H), 1.67 – 1.54 (m, 2H), 1.34 – 1.20 (m, 4H), 1.17 (s, 3H), 1.12 (s, 3H), 0.66 (d, *J* = 7.1 Hz, 3H).
^{13}C NMR (101 MHz, $CDCl_3$) δ 178.7, 146.6, 138.9, 135.8, 135.5, 130.2, 129.2, 128.8, 128.0, 60.5, 46.5, 38.4, 33.8, 29.0, 27.0, 26.3, 24.1, 21.3, 20.2, 14.3.
FT-IR (neat film NaCl): 2973, 2954, 2867, 1728, 1509, 1467, 1382, 1247, 1133, 1030 cm^{-1}.

HR-MS (ESI) m/z: [M+H]+ Calculated for $C_{20}H_{29}O_2^+$: 301.2162 ; Found 301.2170.

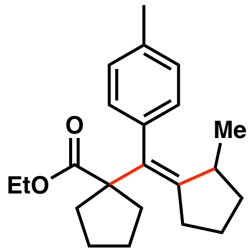

ethyl (*E*)-1-((2-methylcyclopentylidene)(*p*-tolyl)methyl)cyclopentane-1-carboxylate (22)

Following General Procedure 2: To an oven dried dram vial with a magnetic stir bar was added $[Li]^+[B(C_6F_5)_4]^-$ (13.7 mg, 0.02 mmol, 0.1 equiv). To this was added trifluorotoluene (4 mL), and the silyl ketene acetal **SI-21** (128.6 mg, 3 equiv, 0.6 mmol). Alkyl tosylate **SI-28** (71.3 mg, 1.0 equiv, 0.2 mmol) was added and the reaction was allowed to stir at 80 °C in a metal heating block placed on an IKA hot plate for 24 hours. The reaction mixture was removed from the glovebox and diluted with ether containing a drop of triethylamine. This was pushed through a plug of triethylamine treated silica gel in a pipette and concentrated *in vacuo* to give the crude material. The crude material was purified by silica flash chromatography on triethylamine treated silica gel (1% → 2% diethyl ether in hexanes with 0.1% TEA) to give a colorless oil **22** (27.1 mg, 42% yield). The olefin isomer (E) was assigned on the basis of NOESY NMR. Trace amounts of a second compound appear in NMR which may correspond to the Z isomer, though integration of its integral suggests <5%, and isolation of sufficient quantities of this minor product could not be achieved to definitively assign it as the Z isomer.

^{1}H NMR (400 MHz, $CDCl_3$) δ 7.13 – 7.03 (m, 3H), 7.02 – 6.95 (m, 1H), 4.17 (qq, *J* = 10.8, 7.1 Hz, 2H), 2.39 – 2.19 (m, 7H), 2.19 – 2.07 (m, 1H), 1.79 – 1.39 (m, 9H), 1.33 – 1.19 (m, 4H), 0.64 (d, *J* = 7.0 Hz, 3H).

^{13}C NMR (101 MHz, $CDCl_3$) δ 177.4, 147.8, 139.7, 135.7, 135.4, 130.0, 129.2, 128.7, 128.0, 60.0, 58.5, 38.3, 37.9, 36.5, 33.9, 29.8, 24.2, 23.9, 21.3, 19.9, 14.4.

FT-IR (neat film NaCl): 2953, 2869, 1723, 1508, 1450, 1229, 1175, 1157, 1106, 1031, 823 cm^{-1}.

HR-MS (ESI) m/z: [M+H]+ Calculated for $C_{22}H_{31}O_2^+$: 327.2319 ; Found 327.2330.

6 - Mechanistic Studies

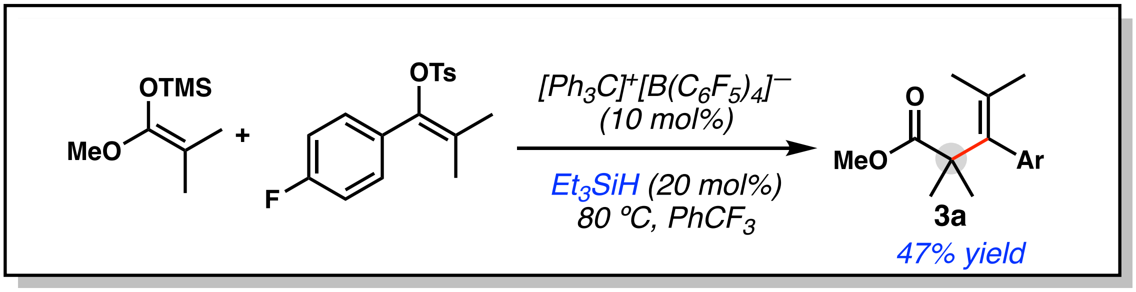

To an oven dried dram vial with a magnetic stir bar was added $[Ph_3C]^+[B(C_6F_5)_4]^-$ (4.6 mg, 0.005 mmol, 0.1 equiv). To this was added trifluorotoluene (0.5 mL), and then triethylsilane (1.60 µL, 0.01 mmol, 0.2 equiv). ((1-methoxy-2-methylprop-1-en-1-yl)oxy)trimethylsilane (26.1 mg, 0.150 mmol, 3 equiv) was added next, and then finally vinyl tosylate **SI-4** (16.0 mg, 0.05 mmol, 1.0 equiv) was added and the reaction was allowed to stir at 80 °C in a metal heating block placed on an IKA hot plate for 12 hours. The reaction mixture was removed from the glovebox and diluted with ether containing a drop of triethylamine. This was pushed through a plug of triethylamine treated silica gel in a pipette and concentrated *in vacuo* to give the crude material. The 47% yield of **3a** was determined by ^{19}F NMR using C_6F_6 as an internal standard.

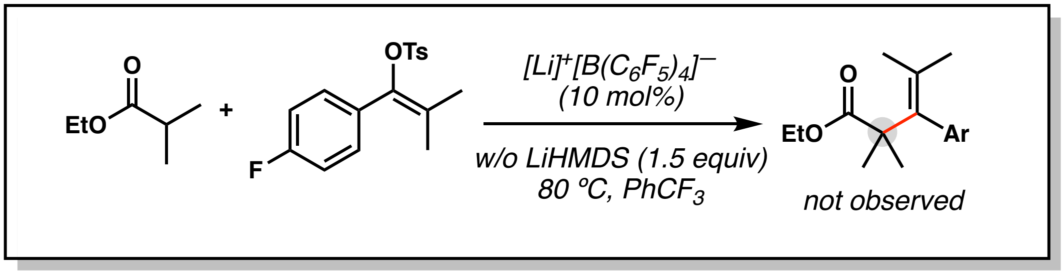

To an oven dried dram vial with a magnetic stir bar was added $[Li]^+[B(C_6F_5)_4]^-$ (3.14 mg, 0.005 mmol, 0.1 equiv) and LiHMDS (12.5 mg, 1.5 equiv, 0.075 mmol). To this was added trifluorotoluene (0.5 mL) and ethyl isobutyrate (20.1 µL, 3 equiv, 0.150 mmol). Vinyl tosylate **SI-4** (16.0 mg, 0.05 mmol, 1.0 equiv) was added and the reaction was allowed to stir at 80 °C in a metal heating block placed on an IKA hot plate for 12 hours. The reaction mixture was removed from the glovebox and diluted with ether containing a drop of triethylamine. This was pushed through a plug of triethylamine treated silica gel in a pipette and concentrated *in vacuo* to give the crude material, which was determined by TLC and ^{1}H NMR to give no conversion to the desired product.

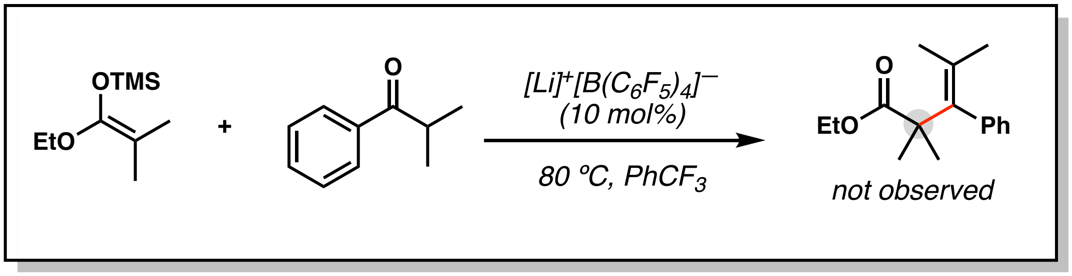

To an oven dried dram vial with a magnetic stir bar was added $[Li]^+[B(C_6F_5)_4]^-$ (3.14 mg, 0.005 mmol, 0.1 equiv). To this was added trifluorotoluene (0.5 mL) and silyl ketene acetal **SI-17** (28.3 mg, 3 equiv, 0.150 mmol). Isobutyrophenone (7.5 μL, 0.05 mmol, 1.0 equiv) was added and the reaction was allowed to stir at 80 °C in a metal heating block placed on an IKA hot plate for 12 hours. The reaction mixture was removed from the glovebox and diluted with ether containing a drop of triethylamine. This was pushed through a plug of triethylamine treated silica gel in a pipette and concentrated *in vacuo* to give the crude material, which was determined by TLC and ^{1}H NMR to give no conversion to the desired product.

7 – References

(1) Kuprat, M.; Lehmann, M.; Schulz, A.; Villinger, A. Synthesis of Pentafluorophenyl Silver by Means of Lewis Acid Catalysis: Structure of Silver Solvent Complexes. *Organometallics* **2010**, *29*, 1421–1427.
(2) Monfette, S.; Turner, Z. R.; Semproni, S. P.; Chirik, P. J. Enantiopure C_1-Symmetric Bis(Imino)Pyridine Cobalt Complexes for Asymmetric Alkene Hydrogenation. *J. Am. Chem. Soc.* **2012**, *134*, 4561–4564.
(3) Estopiñá-Durán, S.; Donnelly, L. J.; Mclean, E. B.; Hockin, B. M.; Slawin, A. M. Z.; Taylor, J. E. Aryl Boronic Acid Catalysed Dehydrative Substitution of Benzylic Alcohols for C−O Bond Formation. *Chem. – Eur. J.* **2019**, *25*, 3950–3956.
(4) Latham, D. E.; Polidano, K.; Williams, J. M. J.; Morrill, L. C. One-Pot Conversion of Allylic Alcohols to α-Methyl Ketones via Iron-Catalyzed Isomerization–Methylation. *Org. Lett.* **2019**, *21*, 7914–7918.
(5) Rappoport, Z.; Kaspi J. Vinylic cations from solvolysis. XVIII. Unusual solvent effects and external ion return in the solvolysis of several vinylic compounds in aqueous trifluoroethanol. *J. Am. Chem. Soc.* **1974**, *96*, 4518–4530.
(6) Dar, D. E.; Thiruvazhi, M.; Abraham, P.; Kitayama, S.; Kopajtic, T. A.; Gamliel, A.; Slusher, B. S.; Carroll, F. I.; Uhl, G. R. Structure–Activity Relationship of Trihexyphenidyl Analogs with Respect to the Dopamine Transporter in the on Going Search for a Cocaine Inhibitor. *Eur. J. Med. Chem.* **2005**, *40*, 1013–1021.
(7) Pandey, G.; Tiwari, S. K.; Singh, B.; Vanka, K.; Jain, S. *P*-Selective (Sp^2)-C–H Functionalization for an Acylation/Alkylation Reaction Using Organic Photoredox Catalysis. *Chem. Commun.* **2017**, *53*, 12337–12340.
(8) Boelke, A.; Caspers, L. D.; Nachtsheim, B. J. NH_2-Directed C–H Alkenylation of 2-Vinylanilines with Vinylbenziodoxolones. *Org. Lett.* **2017**, *19*, 5344–5347.
(9) Boelke, A.; Kuczmera, T. J.; Caspers, L. D.; Lork, E.; Nachtsheim, B. J. Iodolopyrazolium Salts: Synthesis, Derivatizations, and Applications. *Org. Lett.* **2020**, *22*, 7261–7266.
(10) Gutiérrez-Bonet, Á.; Flores-Gaspar, A.; Martin, R. Fe-Catalyzed Regiodivergent [1,2]-Shift of α-Aryl Aldehydes. *J. Am. Chem. Soc.* **2013**, *135*, 12576–12579.

(11) Chan, J. Z.; Chang, Y.; Wasa, M. B(C_6F_5)$_3$-Catalyzed C–H Alkylation of *N*-Alkylamines Using Silicon Enolates without External Oxidant. *Org. Lett.* **2019**, *21*, 984–988.
(12) Hatano, M.; Takagi, E.; Ishihara, K. Sodium Phenoxide−Phosphine Oxides as Extremely Active Lewis Base Catalysts for the Mukaiyama Aldol Reaction with Ketones. *Org. Lett.* **2007**, *9*, 4527–4530.
(13) Zikou, L.; Igglessi-Markopoulou, O. Modified Mukaiyama Reaction for the Synthesis of Quinoline Alkaloid Analogues: Total Synthesis of 3,3-Diisopentenyl- *N*-Methylquinoline-2,4-Dione. *Synthesis* **2008**, *2008*, 1861–1866.
(14) Gao, Y.; Qin, W.; Tian, M.; Zhao, X.; Hu, X. Defluorinative Alkylation of Trifluoromethyl Alkenes with Soft Carbon Nucleophiles Enabled by a Catalytic Amount of Base. *Adv. Synth. Catal.* **2022**, *364*, 2241–2247.
(15) Zhou, F.; Yamamoto, H. A Powerful Chiral Phosphoric Acid Catalyst for Enantioselective Mukaiyama-Mannich Reactions. *Angew. Chem. Int. Ed.* **2016**, *55*, 8970–8974.
(16) Kholod, I.; Vallat, O.; Buciumas, A.-M.; Neels, A.; Neier, R. Synthetic Strategies for the Synthesis and Transformation of Substituted Pyrrolinones as Advanced Intermediates for Rhazinilam Analogues. *Eur. J. Org. Chem.* **2014**, *2014*, 7865–7877.
(17) Schulz, Jr. W. J.; Speier, J. L. The Synthesis of *O*-Silyl Ketene Acetals from α-Haloesters. *Synthesis* **1989**, *3*, 163–166.
(18) Molinaro, C.; Jamison, T. F. Nickel-Catalyzed Reductive Coupling of Alkynes and Epoxides. *J. Am. Chem. Soc.* **2003**, *125*, 8076–8077.
(19) Liu, Z.; Pan, Y.; Zou, P.; Huang, H.; Chen, Y.; Chen, Y. Hypervalent Iodine Reagents Enable C–H Alkynylation with Iminophenylacetic Acids via Alkoxyl Radicals. *Org. Lett.* **2022**, *24*, 5951–5956.
(20) Cheng, L.-C.; Chen, W.-C.; Santhoshkumar, R.; Chao, T.-H.; Cheng, M.-J.; Cheng, C.-H. Synthesis of Quinolinium Salts from *N*-Substituted Anilines, Aldehydes, Alkynes, and Acids: Theoretical Understanding of the Mechanism and Regioselectivity: Synthesis of Quinolinium Salts from N-Substituted Anilines, Aldehydes, Alkynes, and Acids: Theoretical Understanding of the Mechanism and Regioselecti. *Eur. J. Org. Chem.* **2020**, *2020*, 2116–2129.

8 – ^{1}H, ^{13}C, ^{19}F NMR Spectral Data

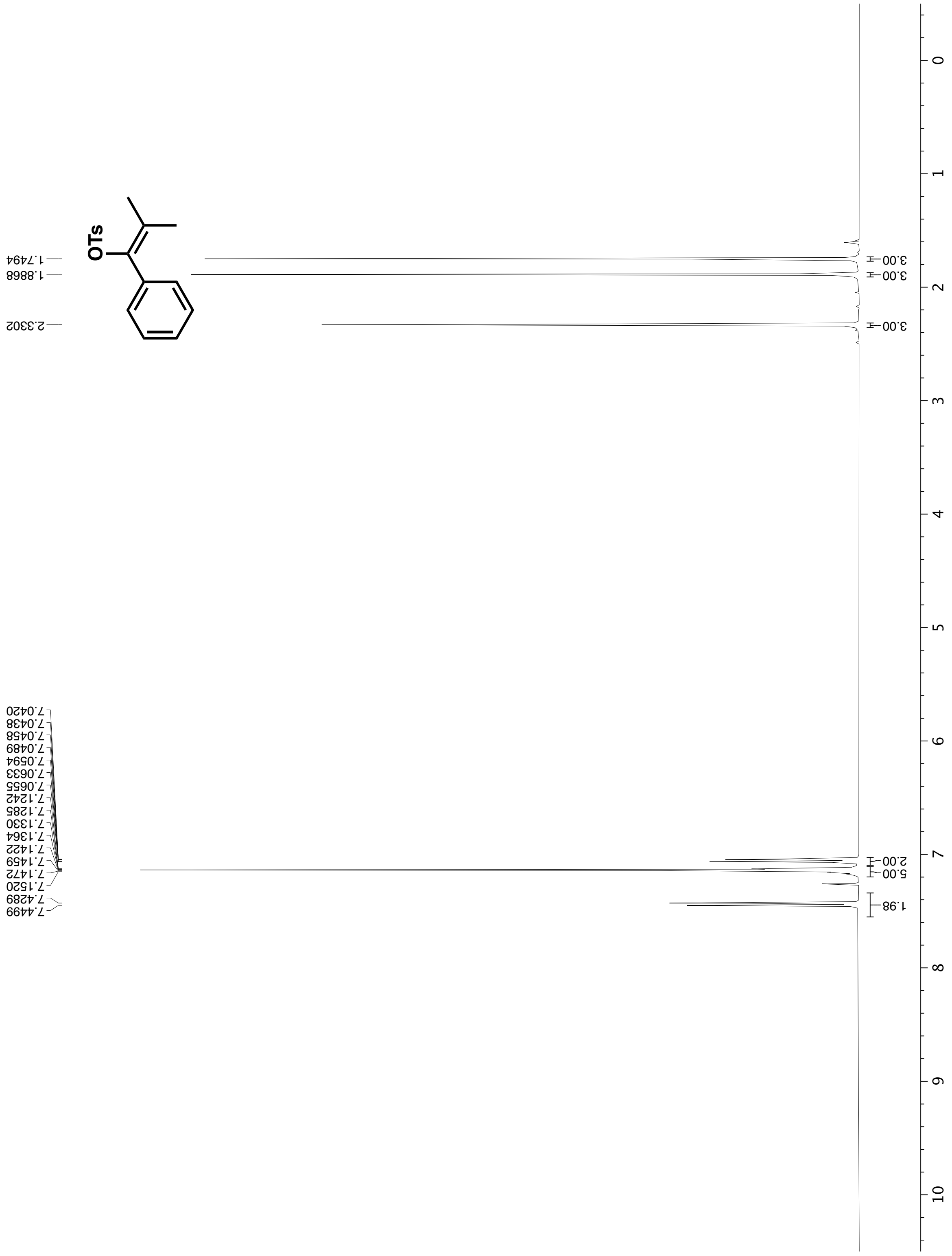

^{1}H NMR (400 MHz, $CDCl_3$) of compound **SI-1**.

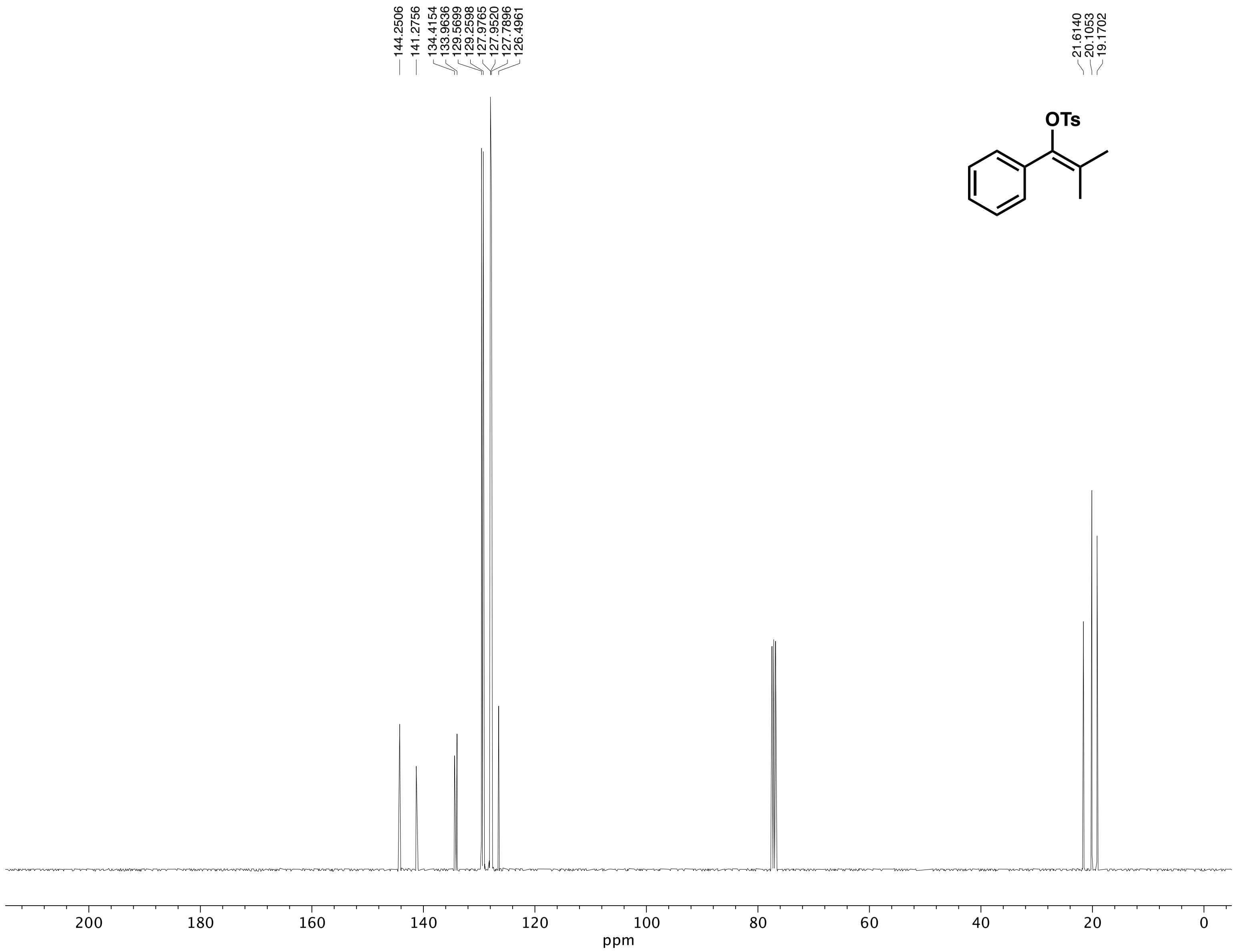

^{13}C NMR (101 MHz, $CDCl_3$) of compound **SI-1**.

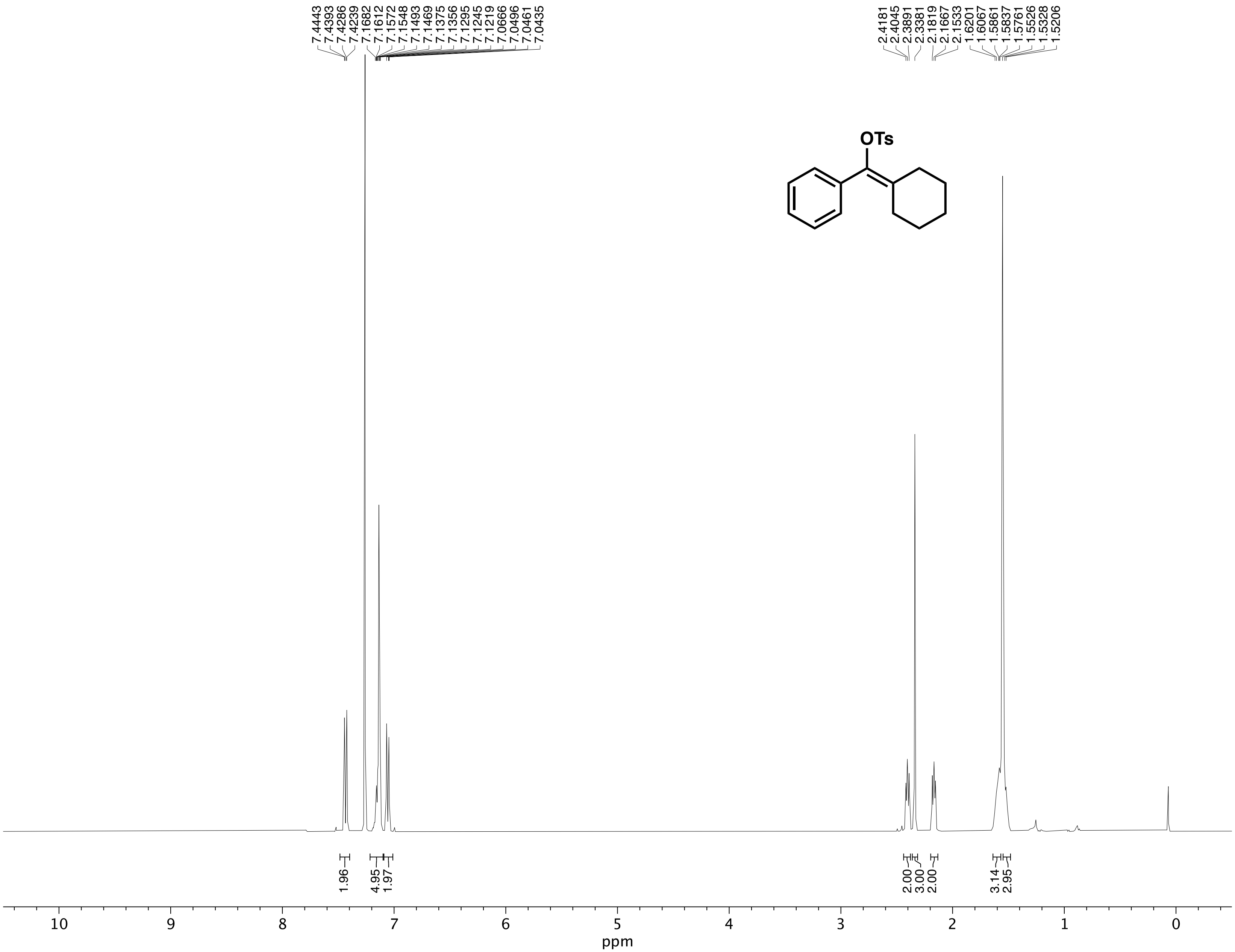

^{1}H NMR (400 MHz, $CDCl_3$) of compound **SI-2**.

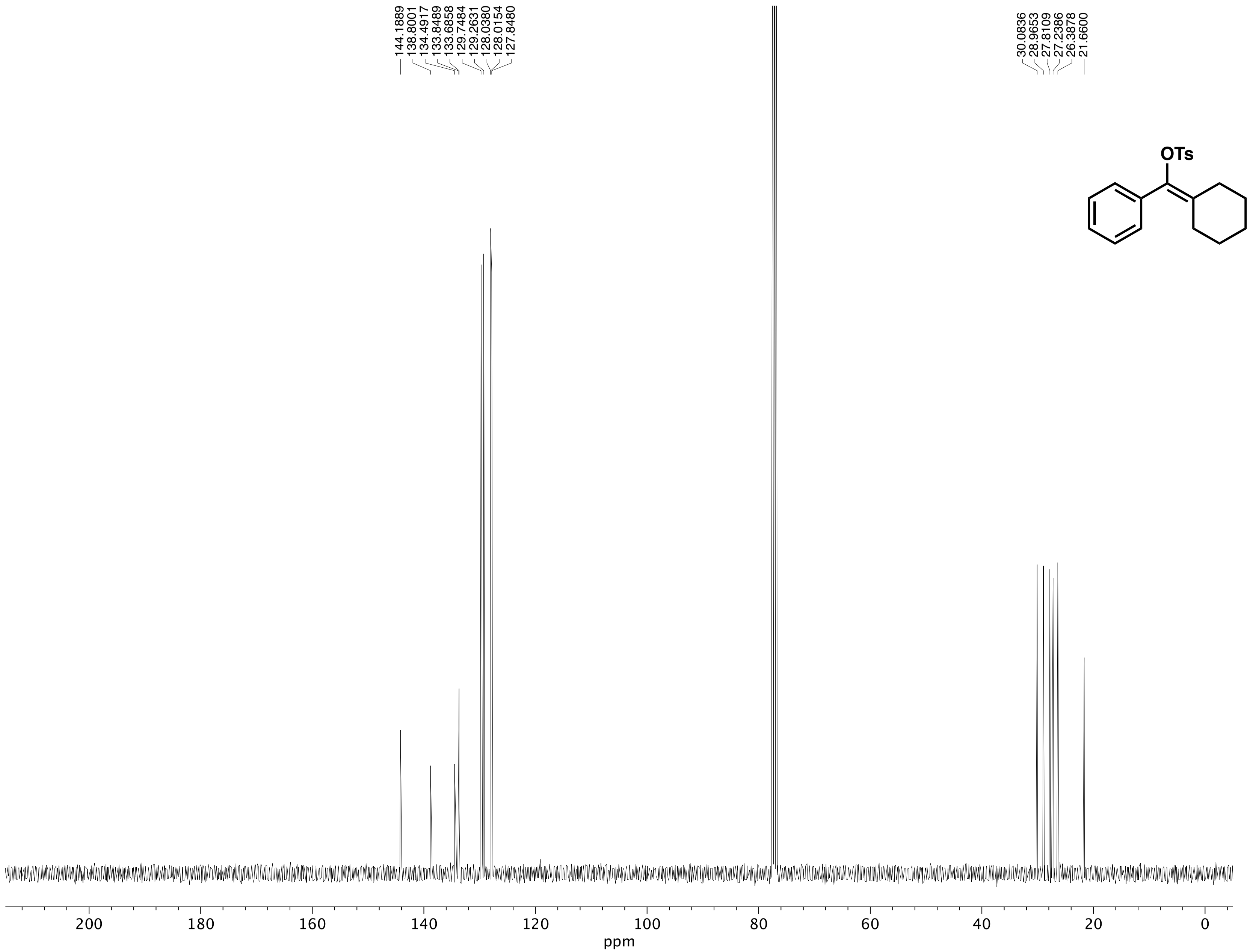

^{13}C NMR (101 MHz, $CDCl_3$) of compound **SI-2**.

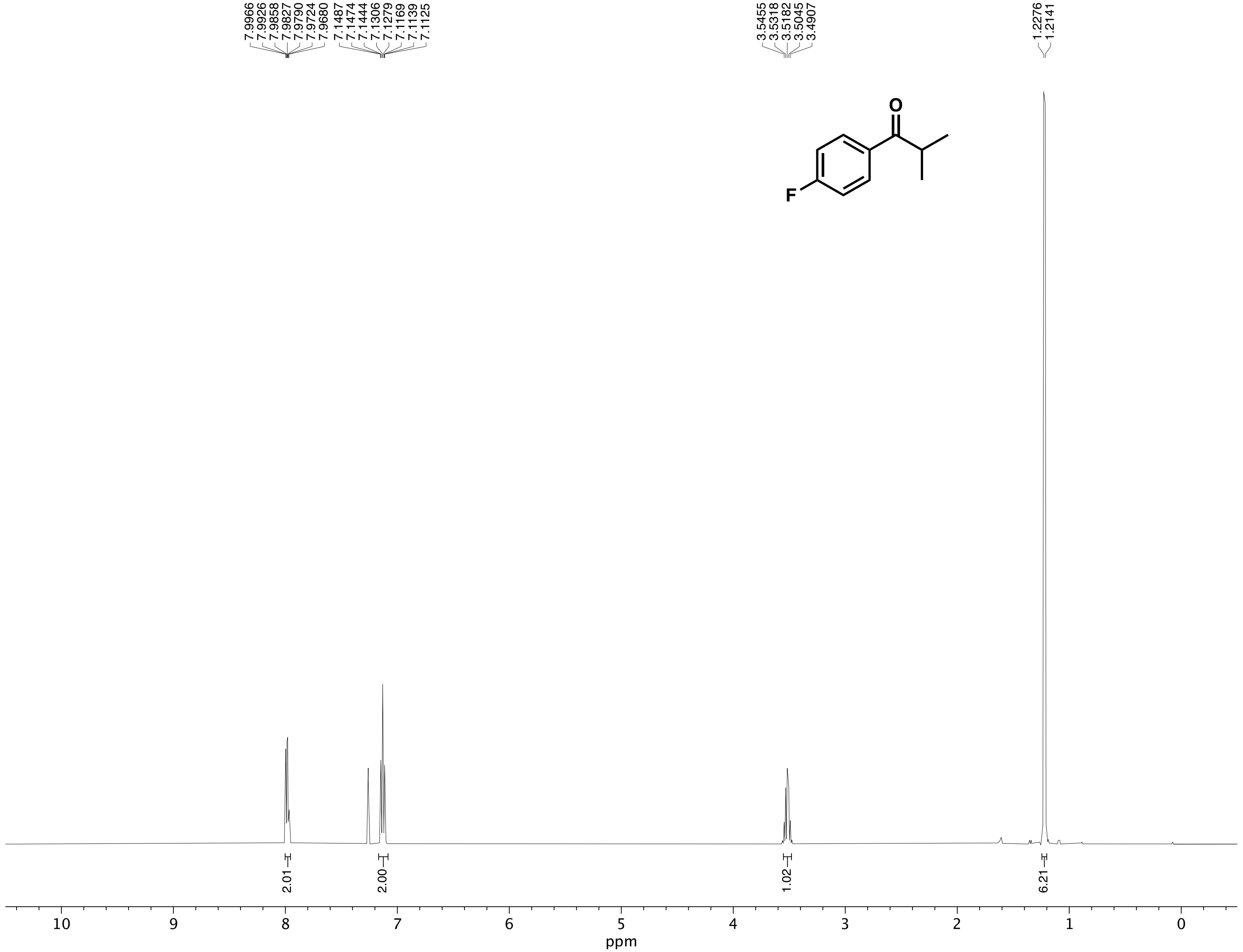

^{1}H NMR (500 MHz, $CDCl_3$) of compound **SI-3.**

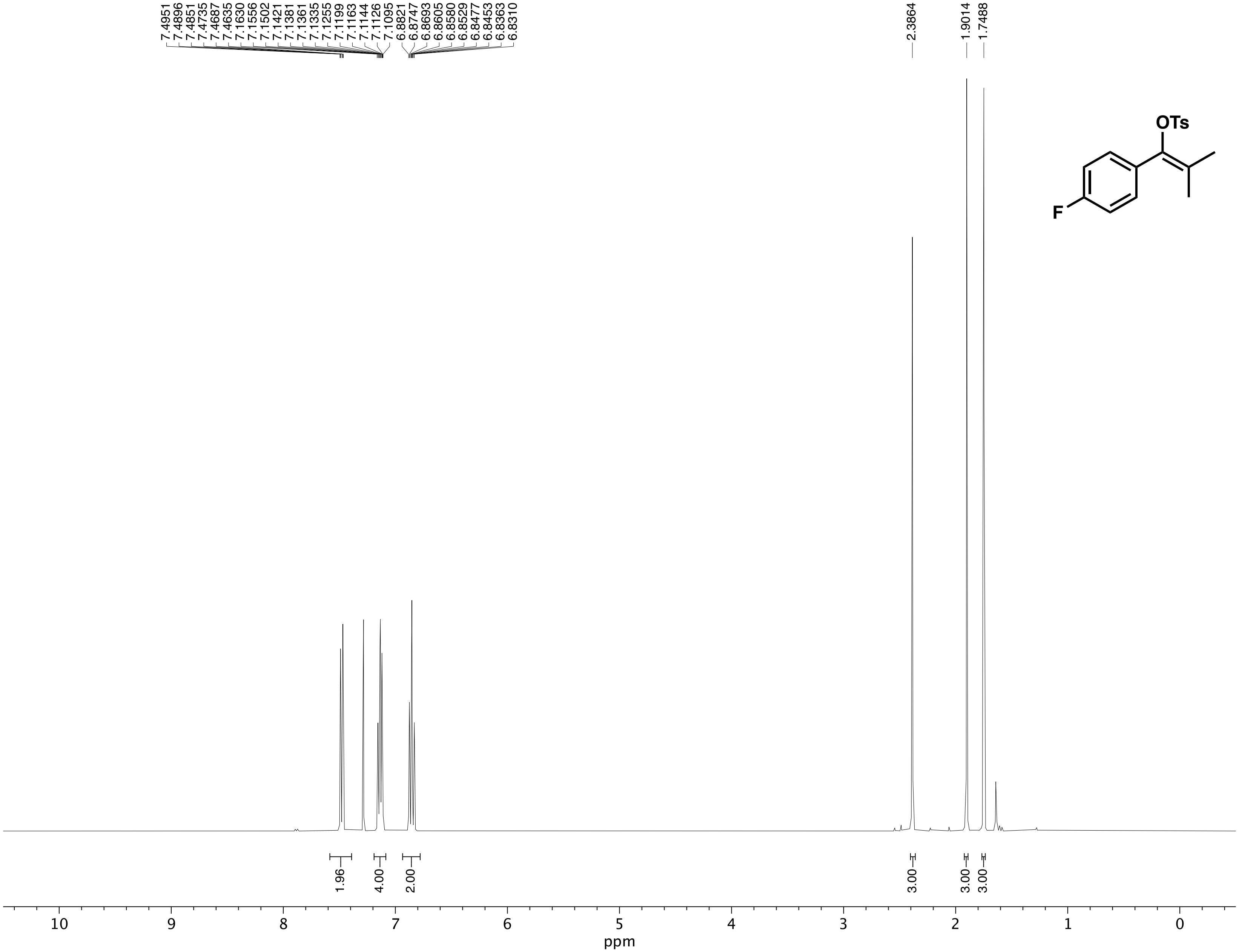

^{1}H NMR (400 MHz, $CDCl_3$) of compound **SI-4**.

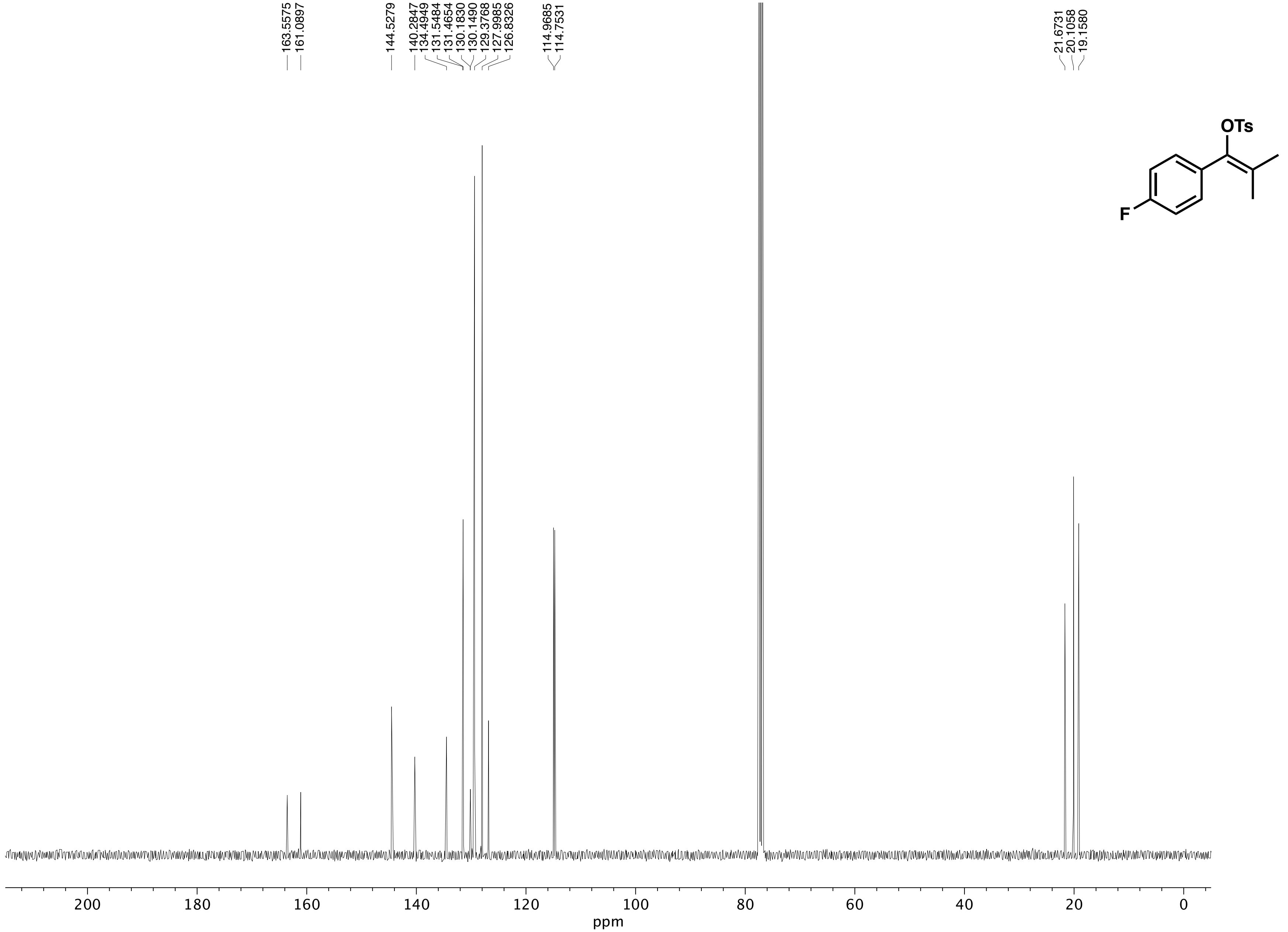

^{13}C NMR (101 MHz, $CDCl_3$) of compound **SI-4**.

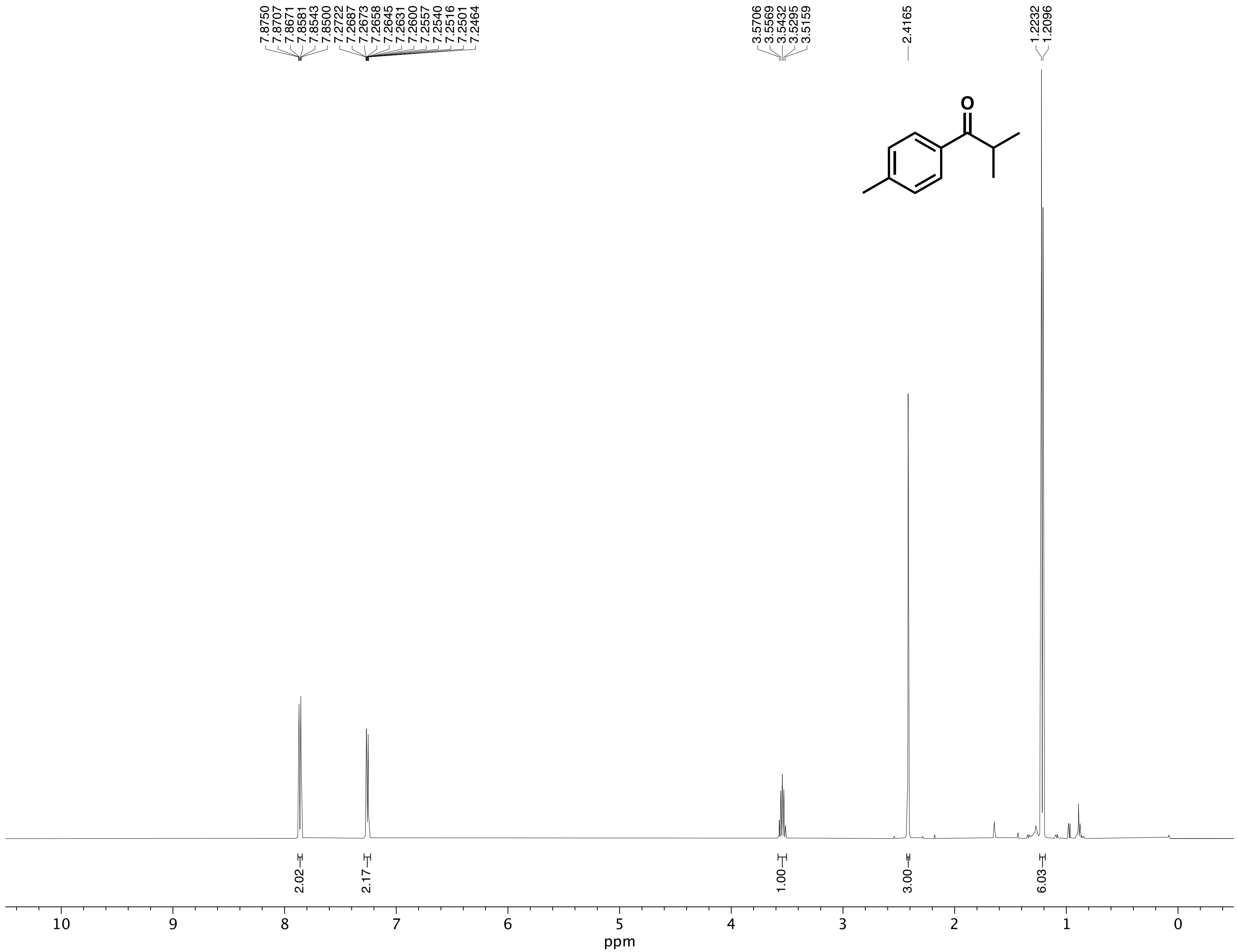

^{1}H NMR (500 MHz, $CDCl_3$) of compound **SI-5**.

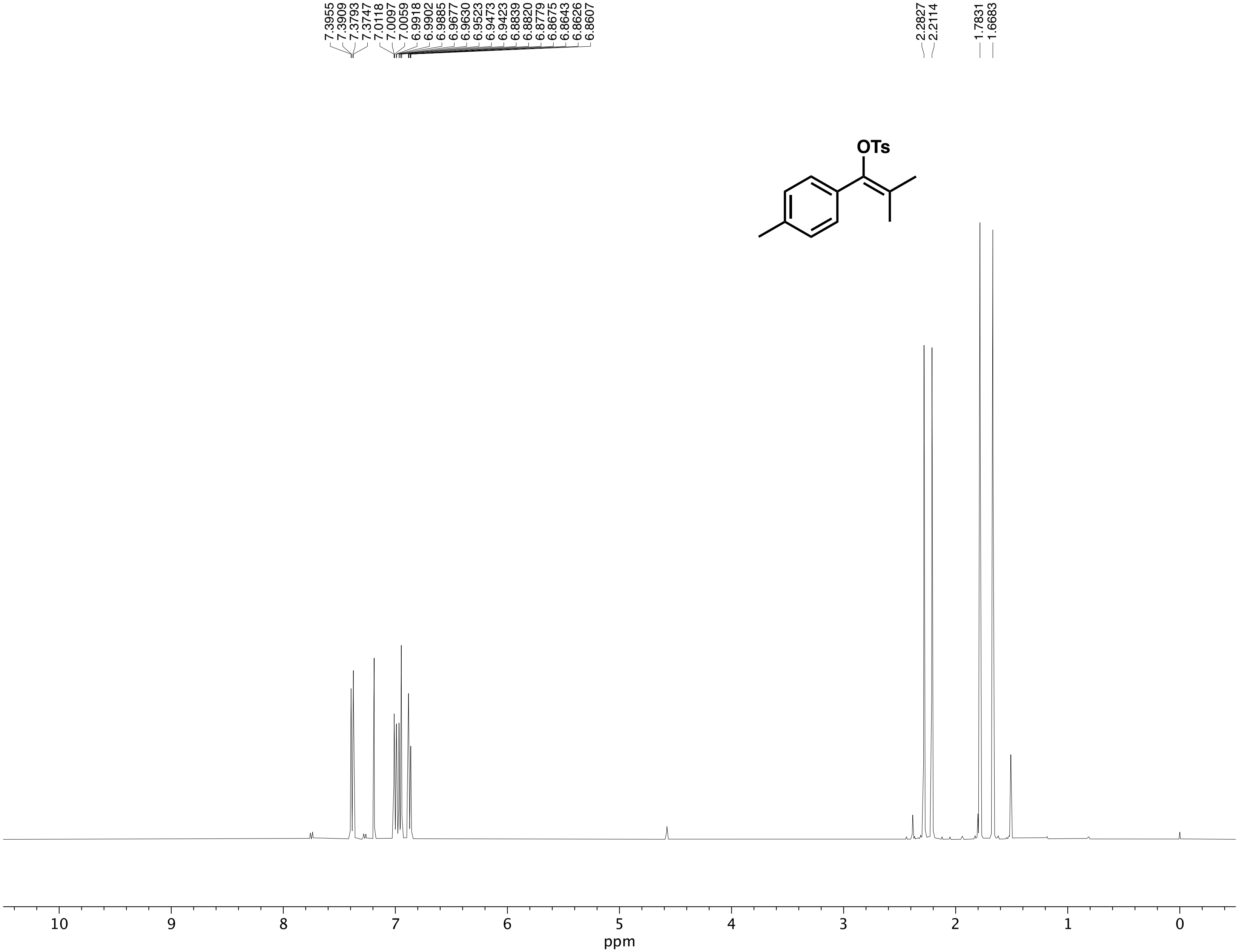

^{1}H NMR (400 MHz, $CDCl_3$) of compound **SI-6**.

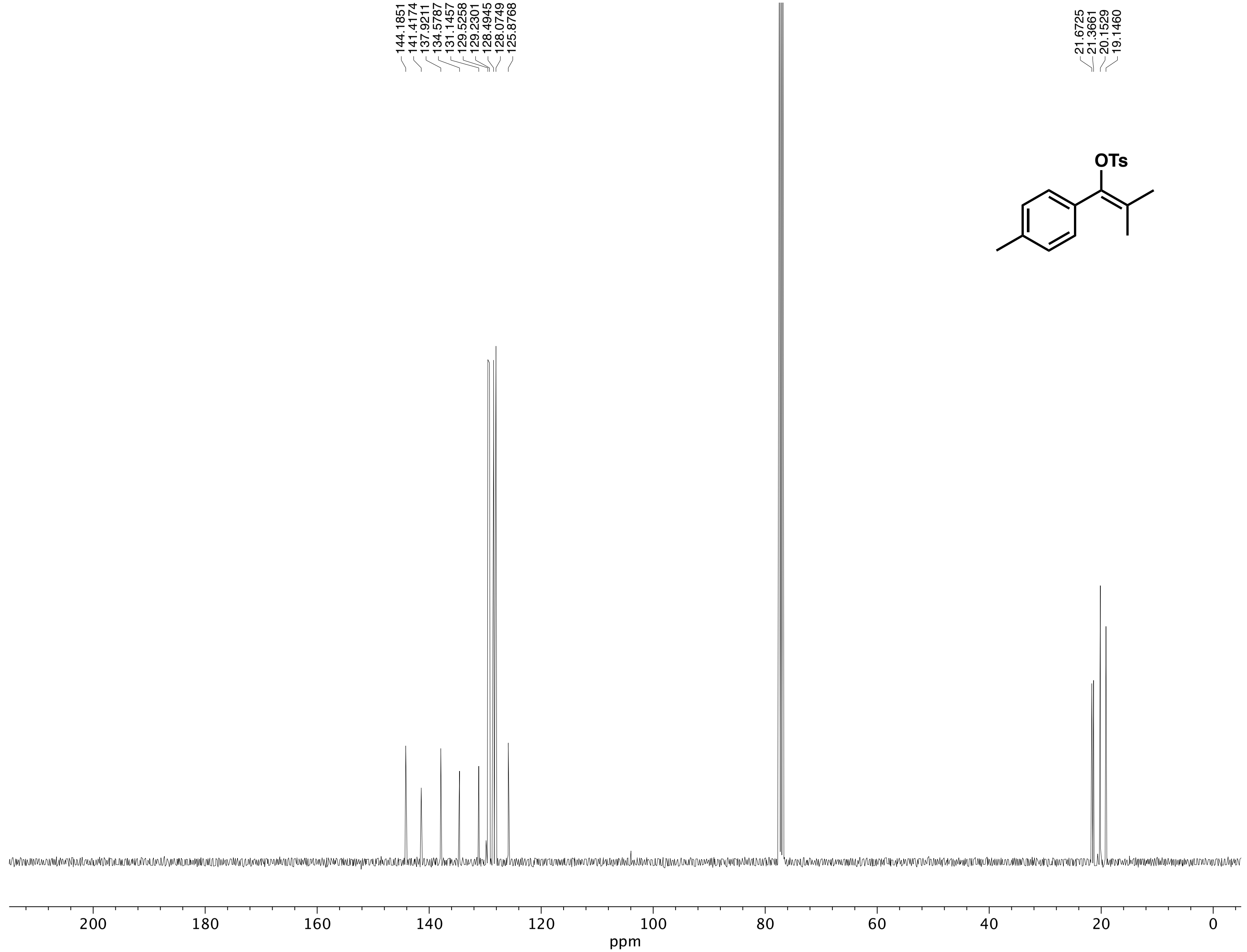

^{13}C NMR (101 MHz, $CDCl_3$) of compound **SI-6**.

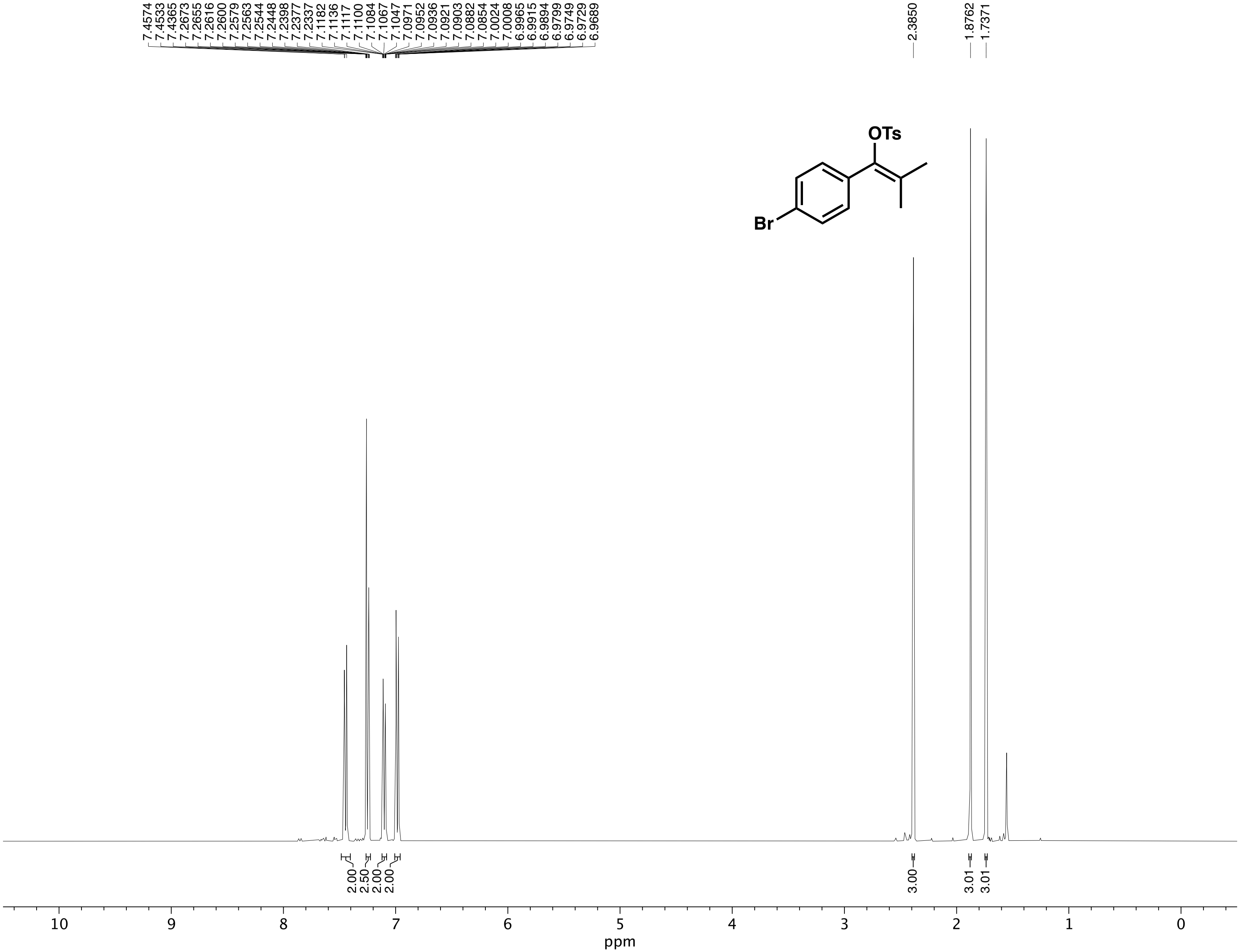

^1H NMR (400 MHz, $CDCl_3$) of compound **SI-7**.

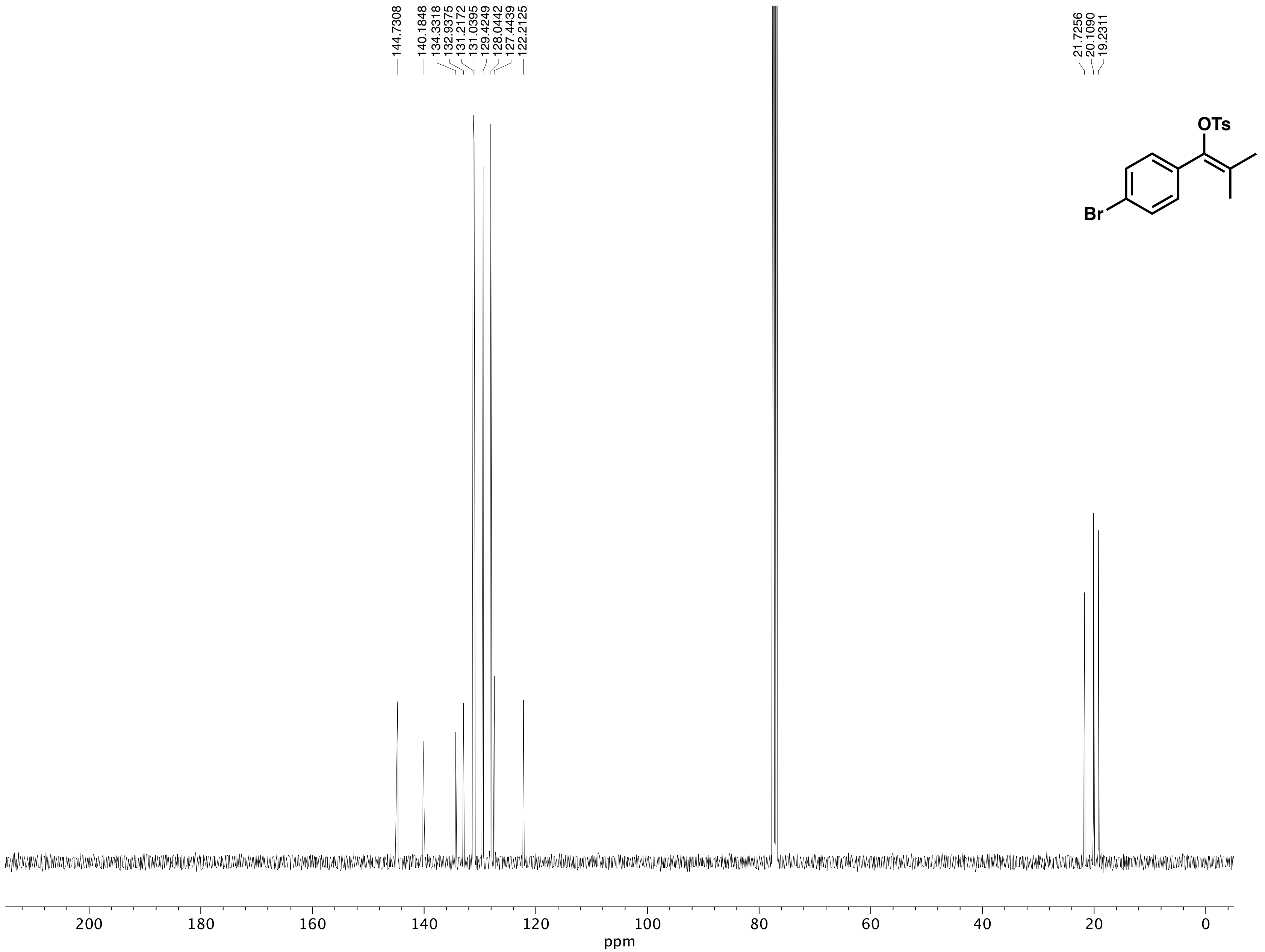

^{13}C NMR (101 MHz, $CDCl_3$) of compound **SI-7**.

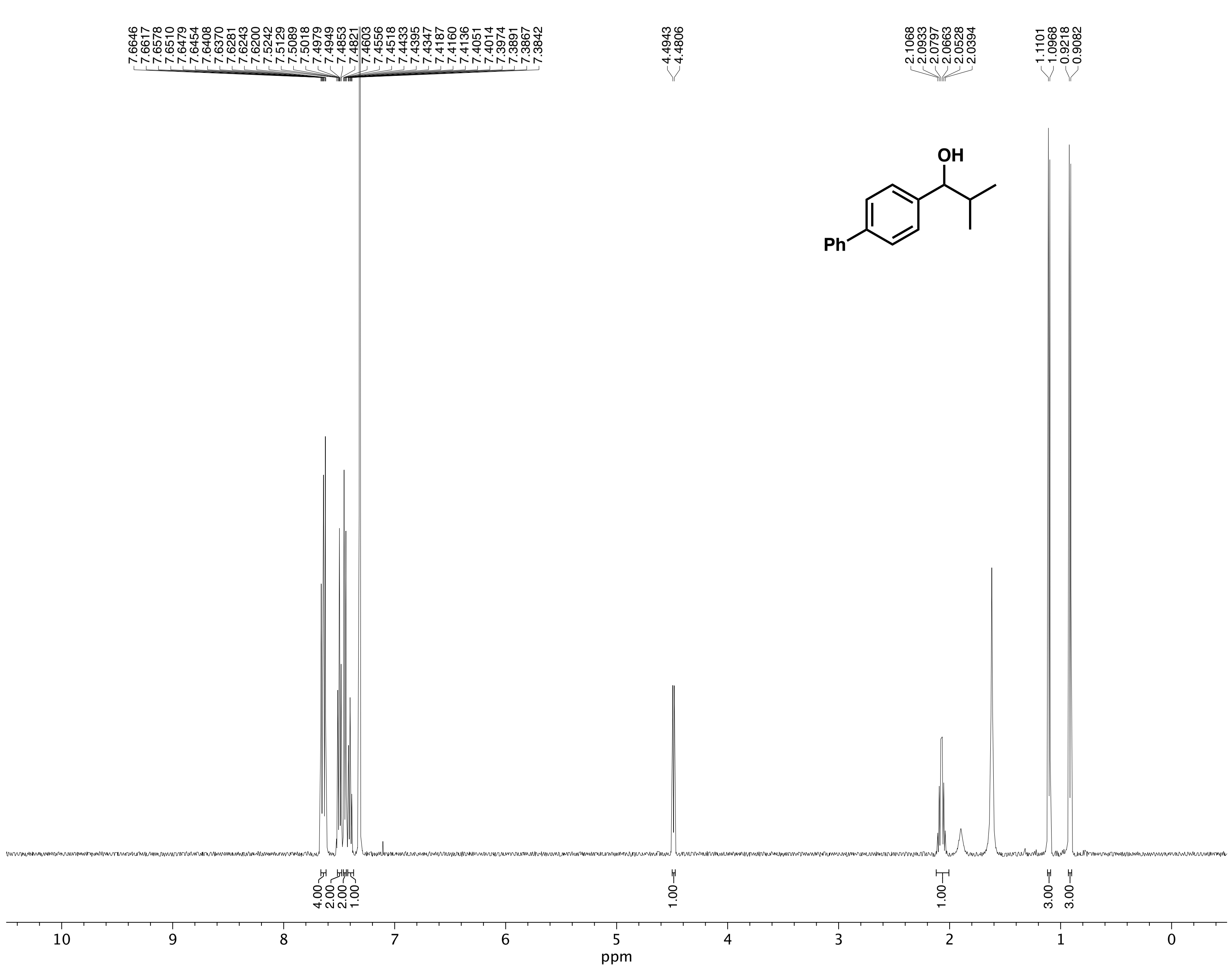

^{1}H NMR (500 MHz, $CDCl_3$) of compound **SI-8**.

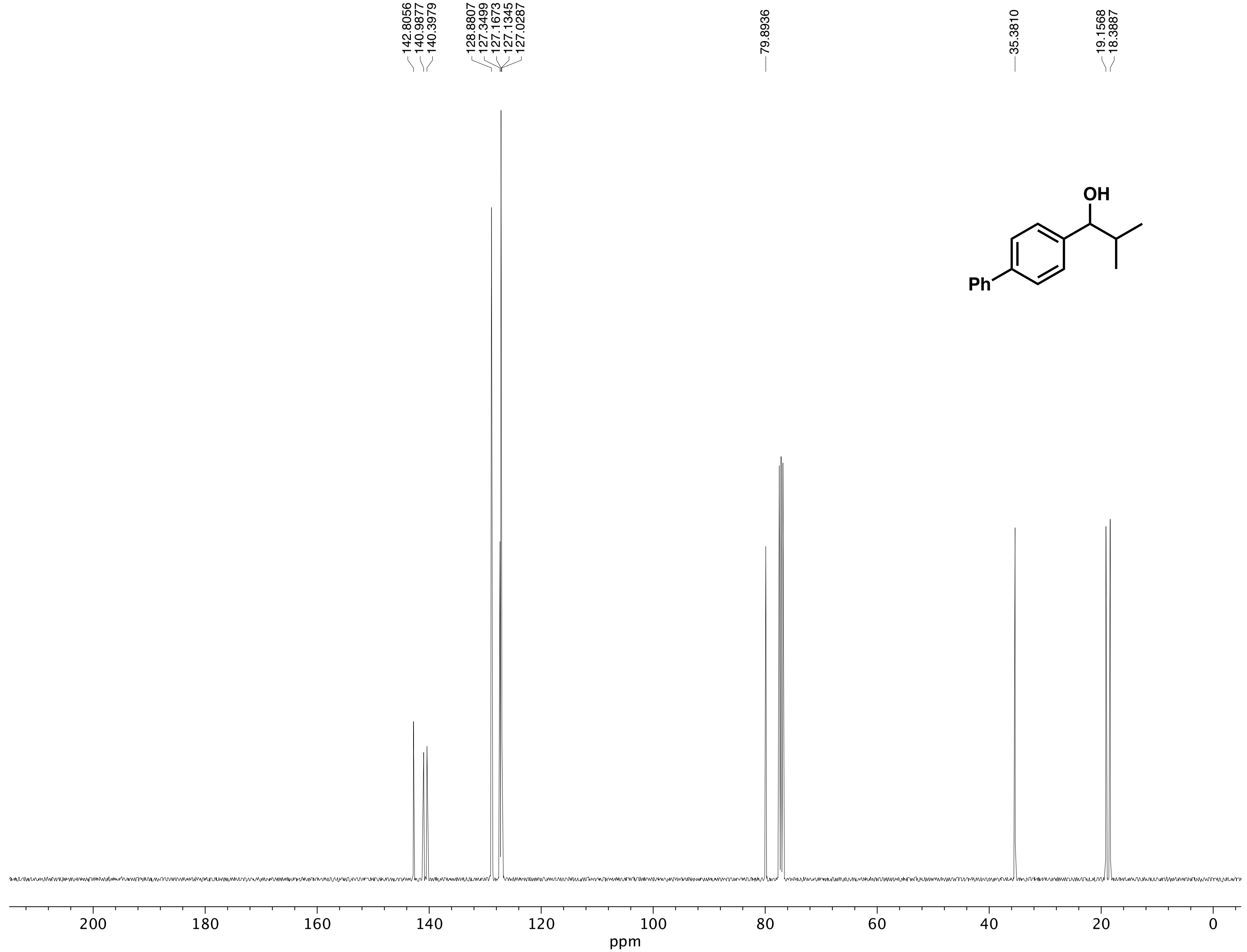

^{13}C NMR (101 MHz, $CDCl_3$) of compound **SI-8.**

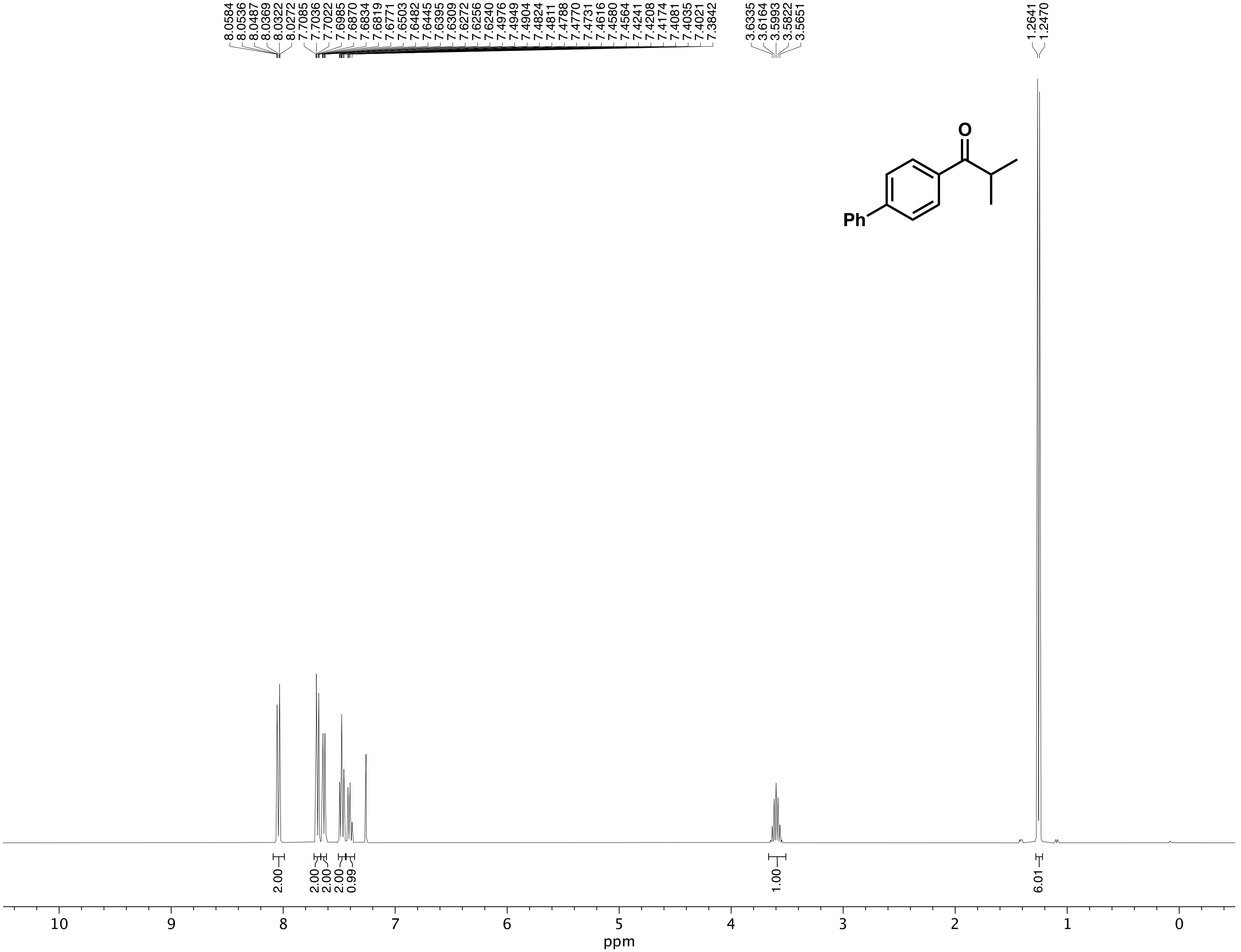

^{1}H NMR (400 MHz, $CDCl_3$) of compound **SI-9**.

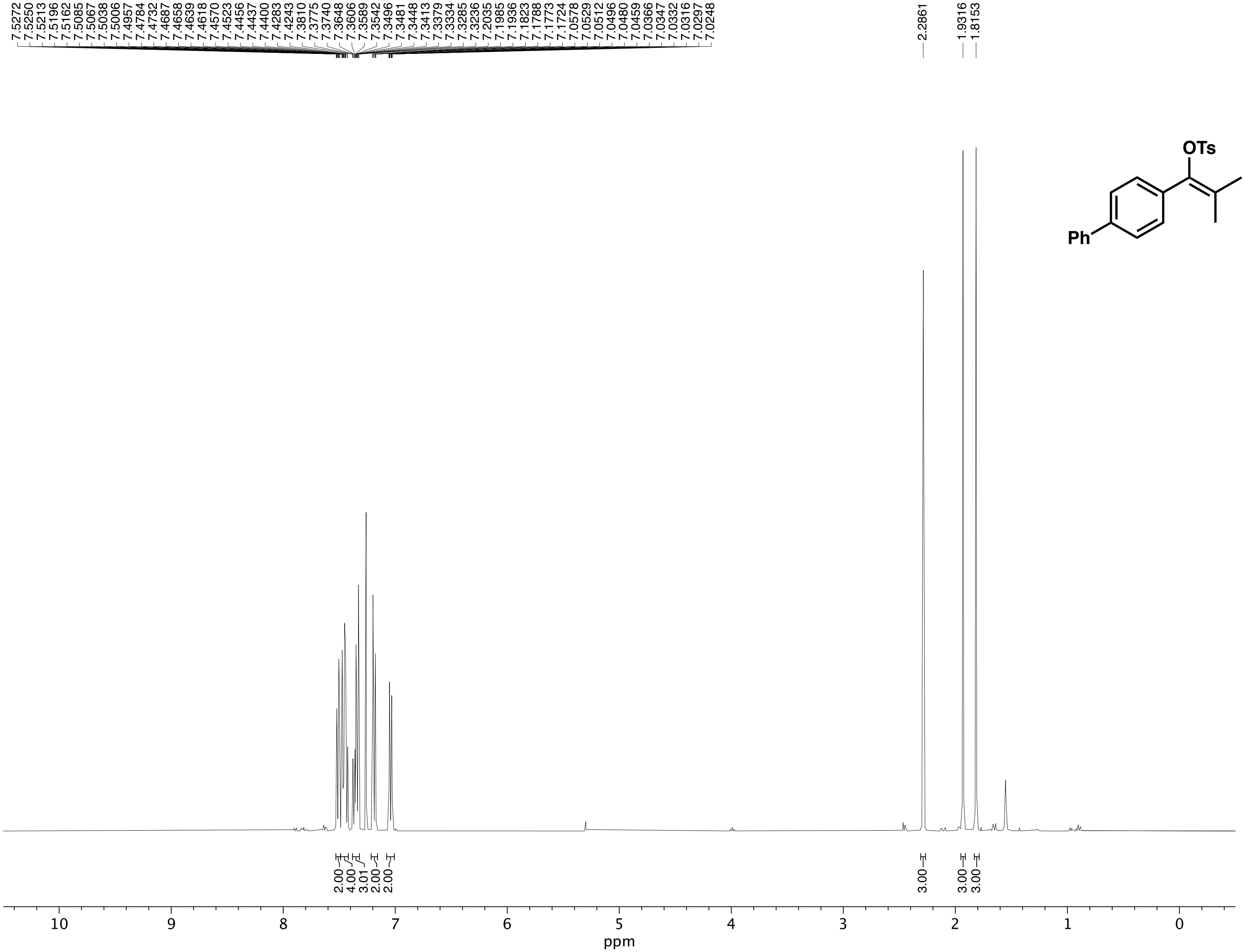

^{1}H NMR (400 MHz, $CDCl_3$) of compound **SI-10**.

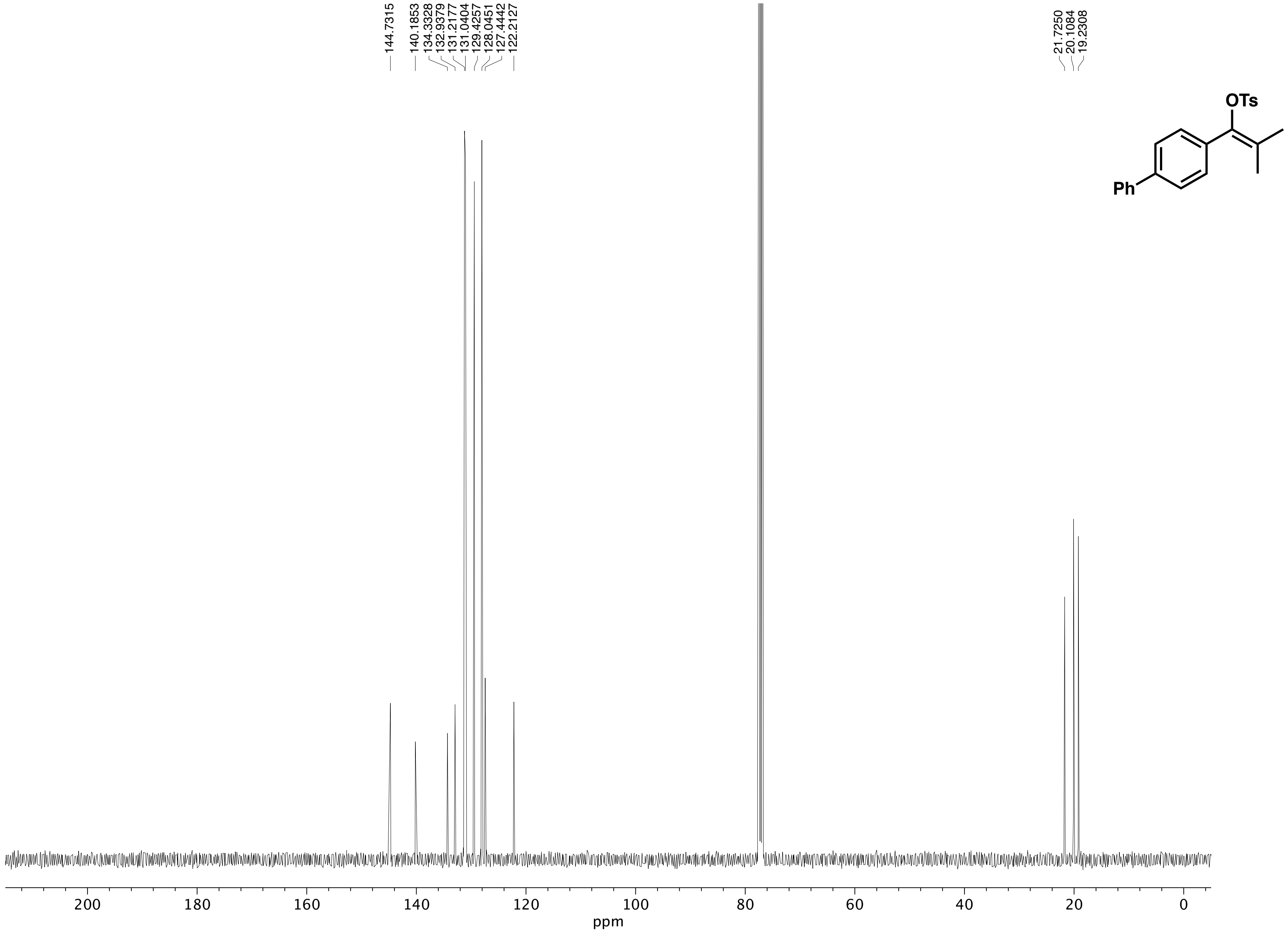

^{13}C NMR (101 MHz, $CDCl_3$) of compound **SI-10.**

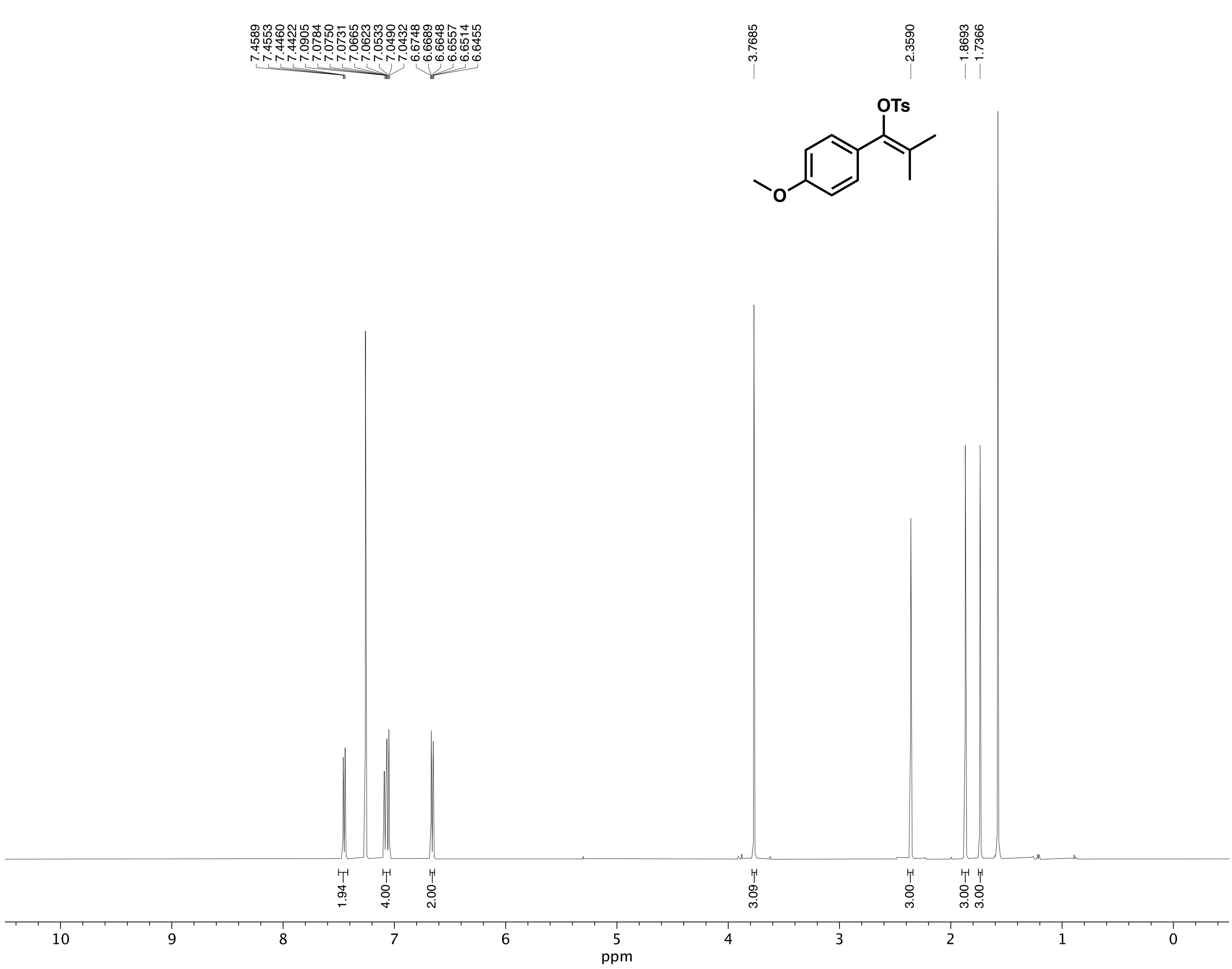

^{1}H NMR (500 MHz, $CDCl_3$) of compound **SI-11.**

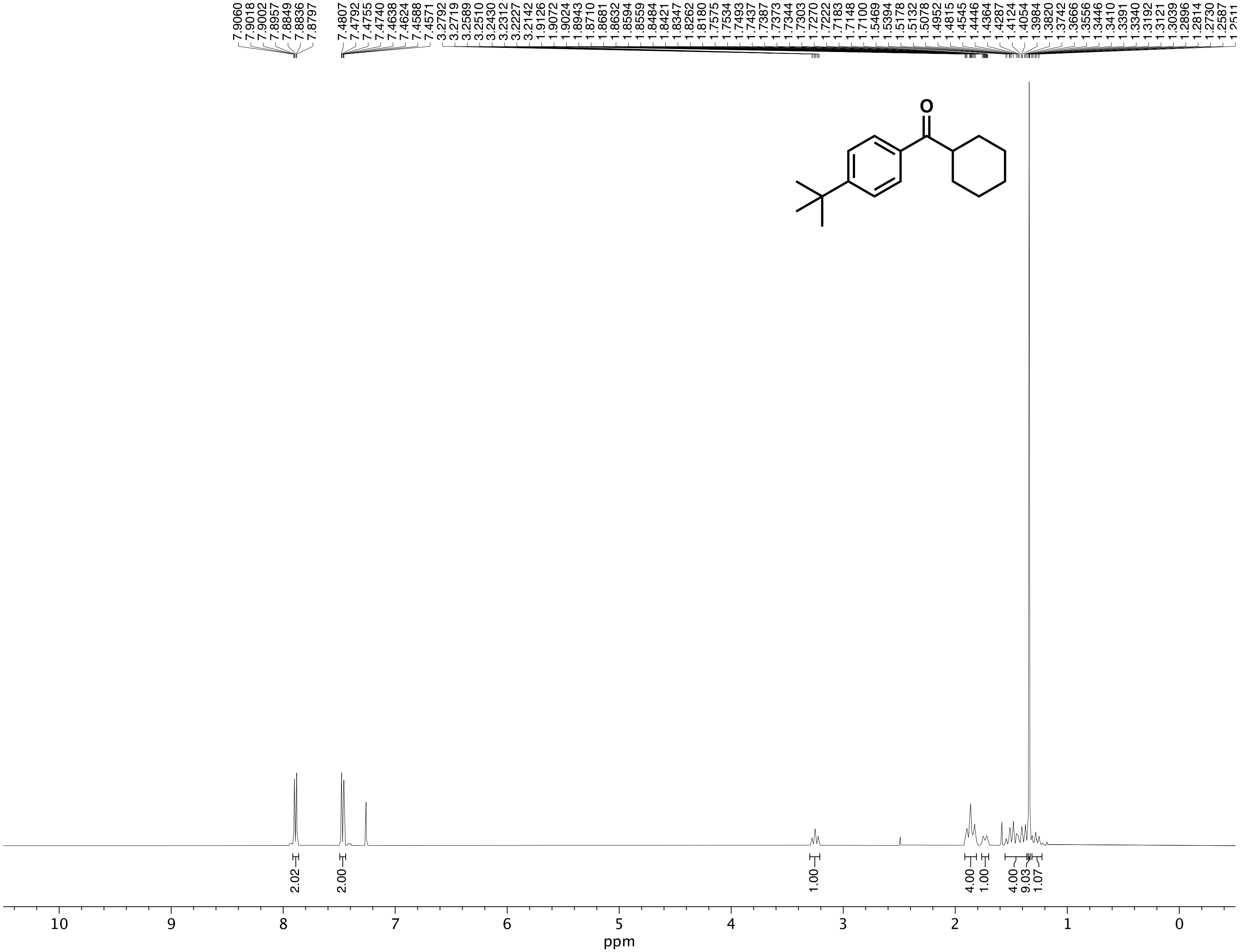

^1H NMR (400 MHz, $CDCl_3$) of compound **SI-12**.

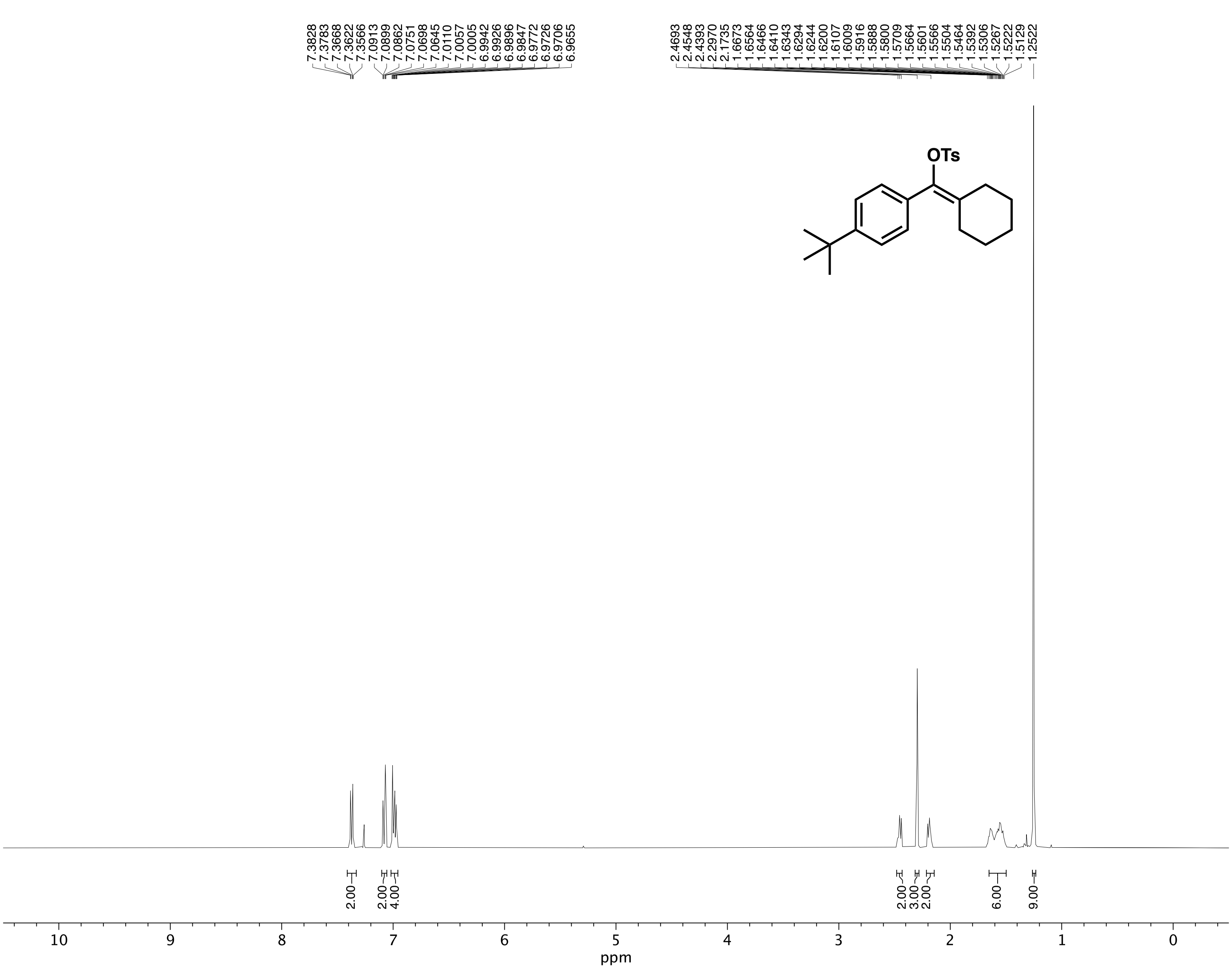

^{1}H NMR (400 MHz, $CDCl_3$) of compound **SI-13**.

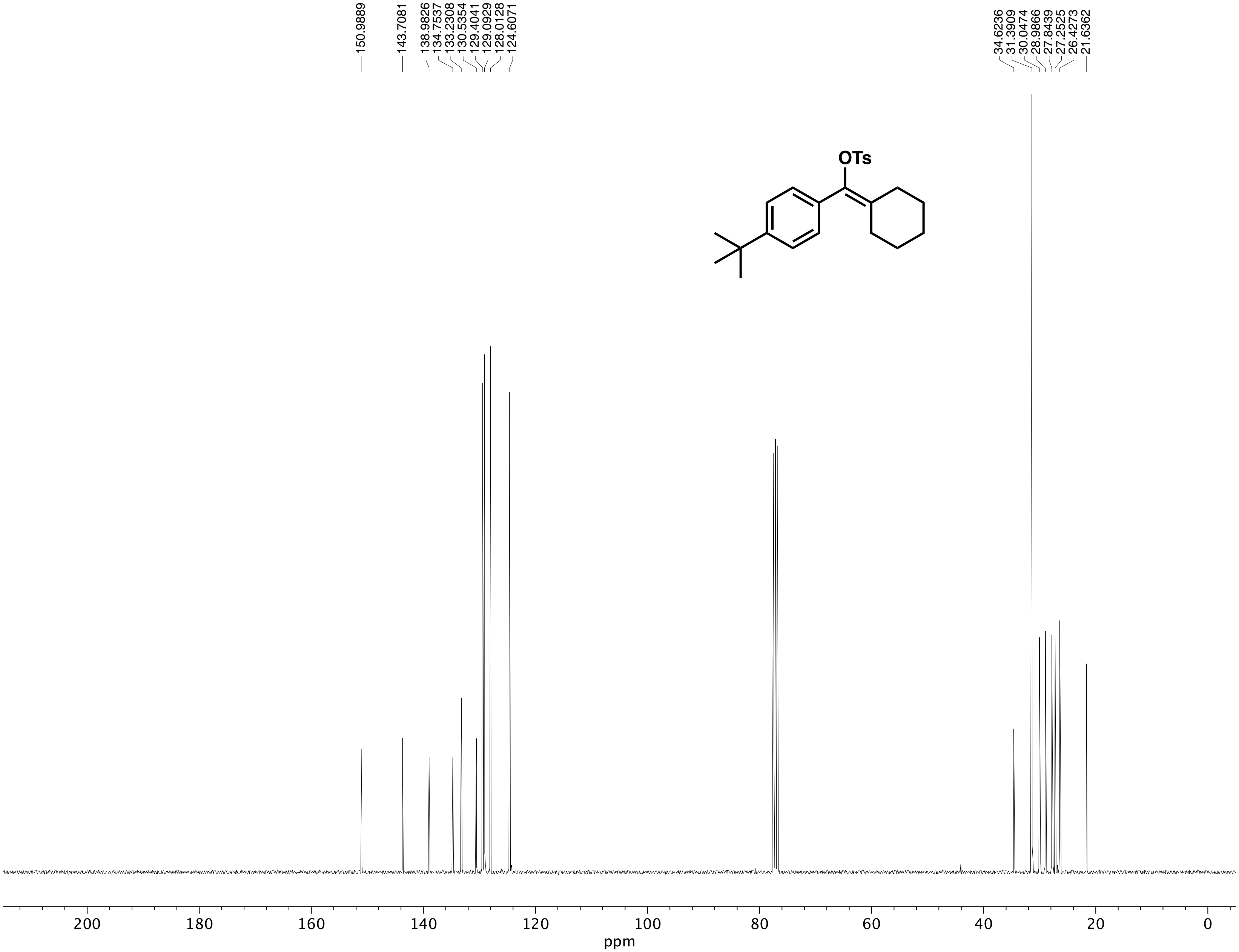

^{13}C NMR (101 MHz, $CDCl_3$) of compound **SI-13.**

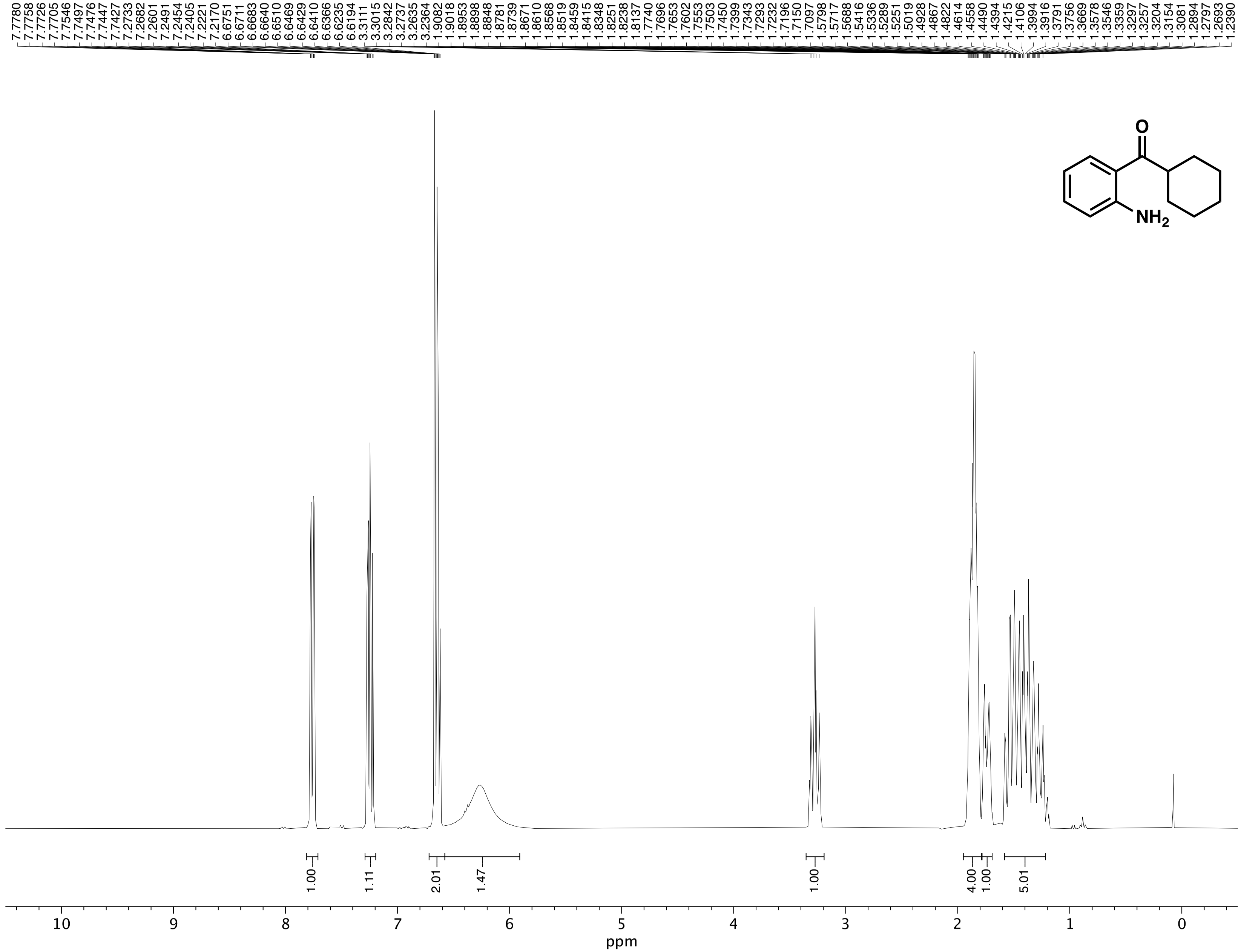

^{1}H NMR (400 MHz, $CDCl_3$) of compound **SI-14**.

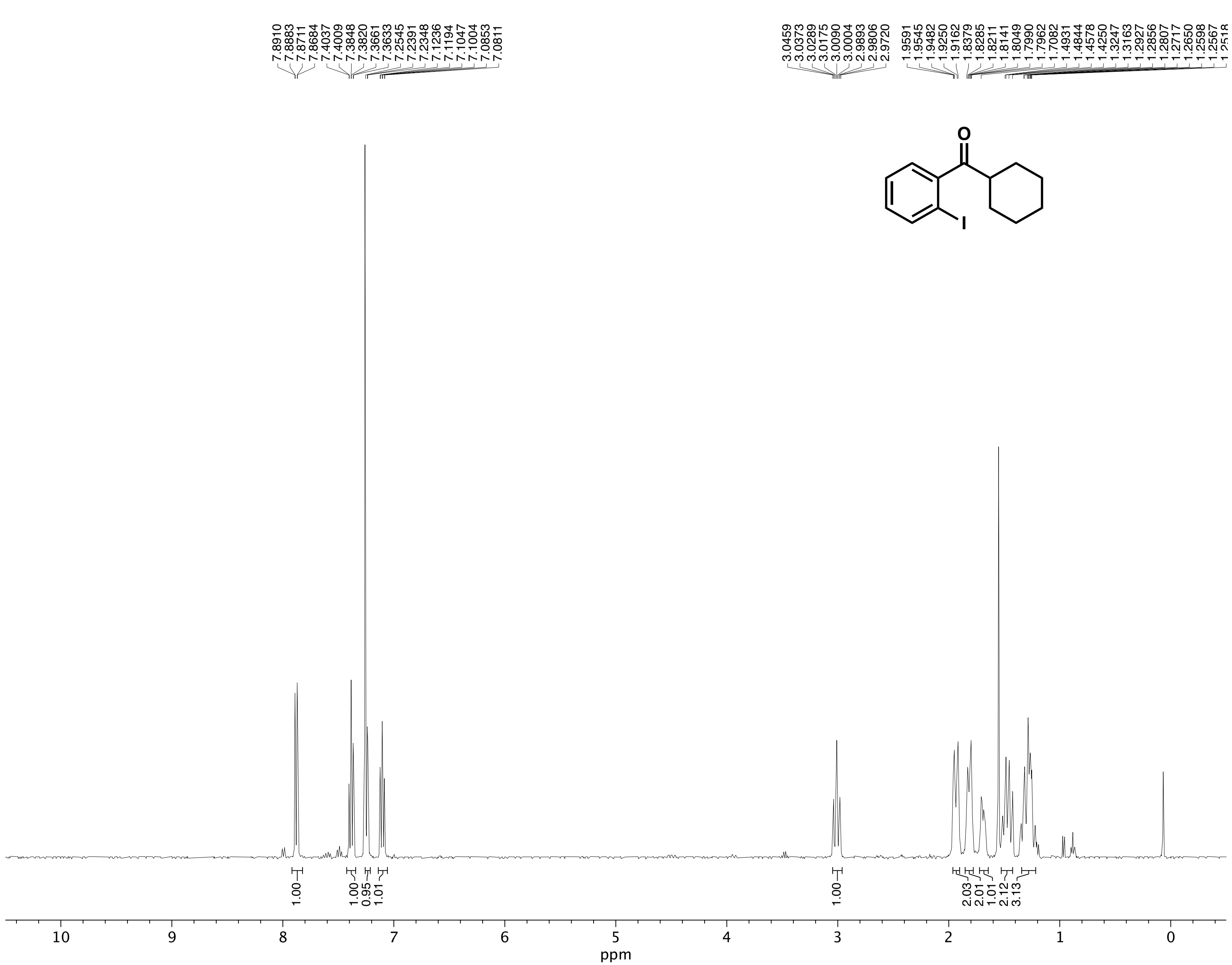

^{1}H NMR (400 MHz, $CDCl_3$) of compound **SI-15**.

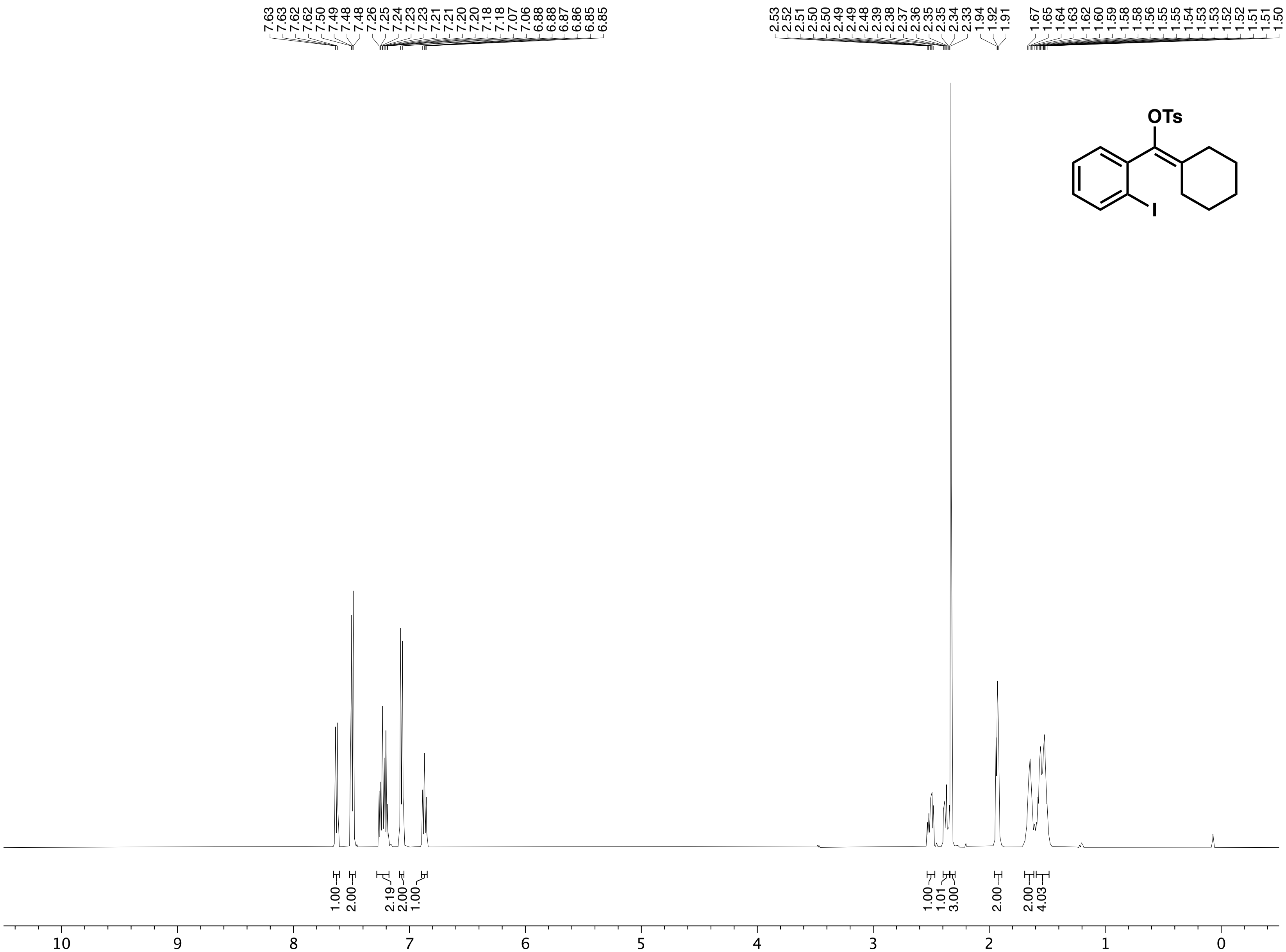

^{1}H NMR (500 MHz, $CDCl_3$) of compound **SI-16**.

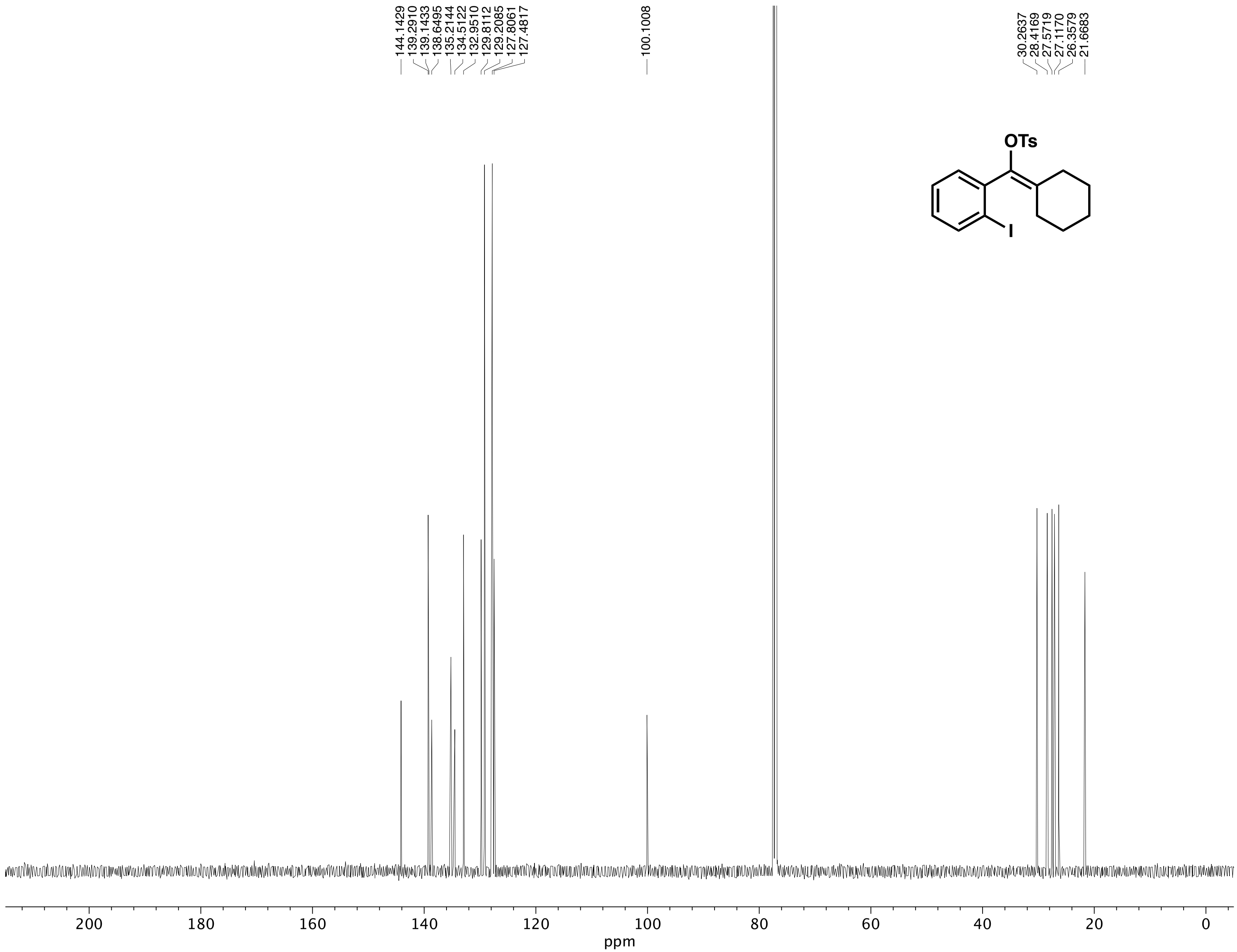

^{13}C NMR (126 MHz, $CDCl_3$) of compound **SI-16**.

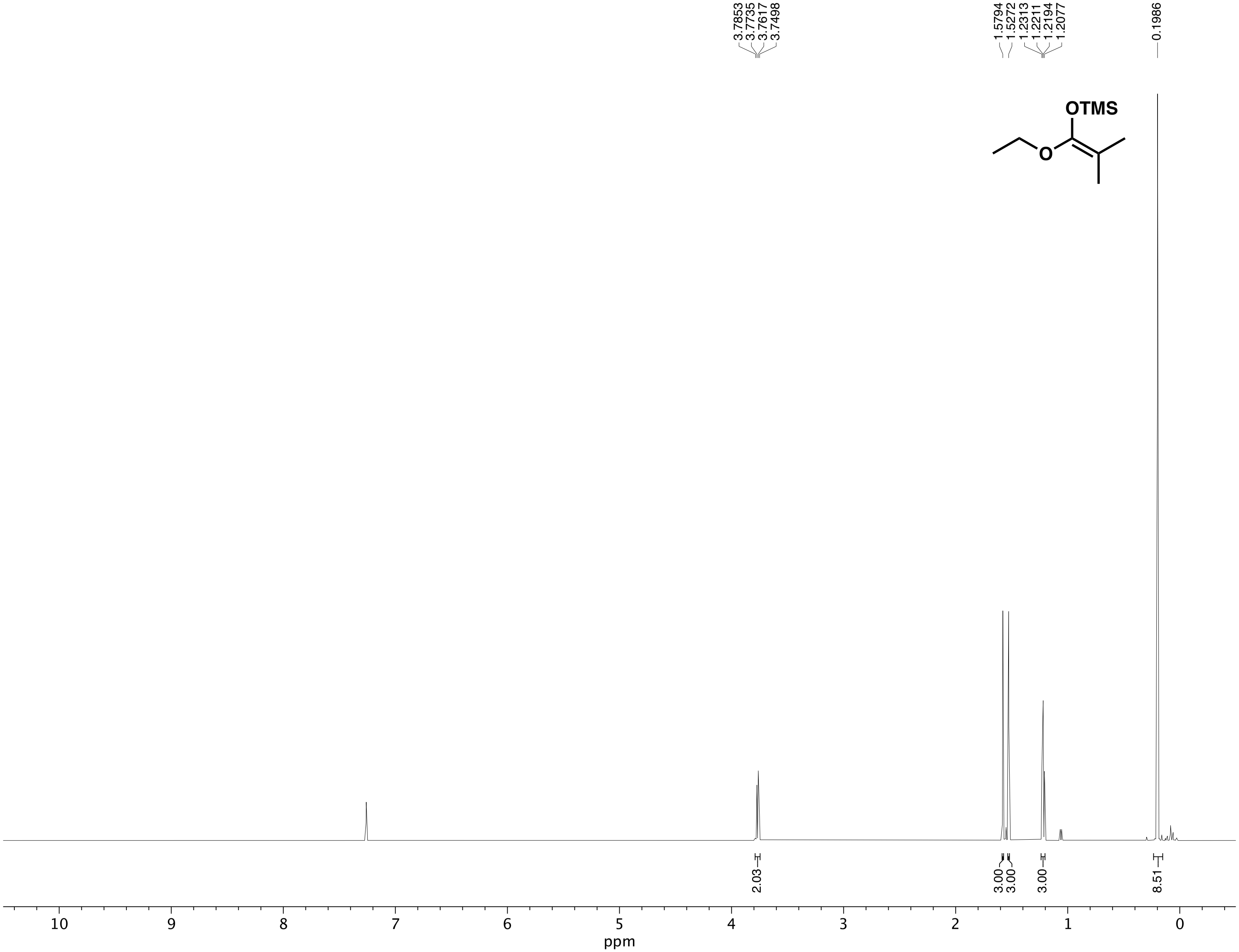

^{1}H NMR (600 MHz, $CDCl_3$) of compound **SI-17**.

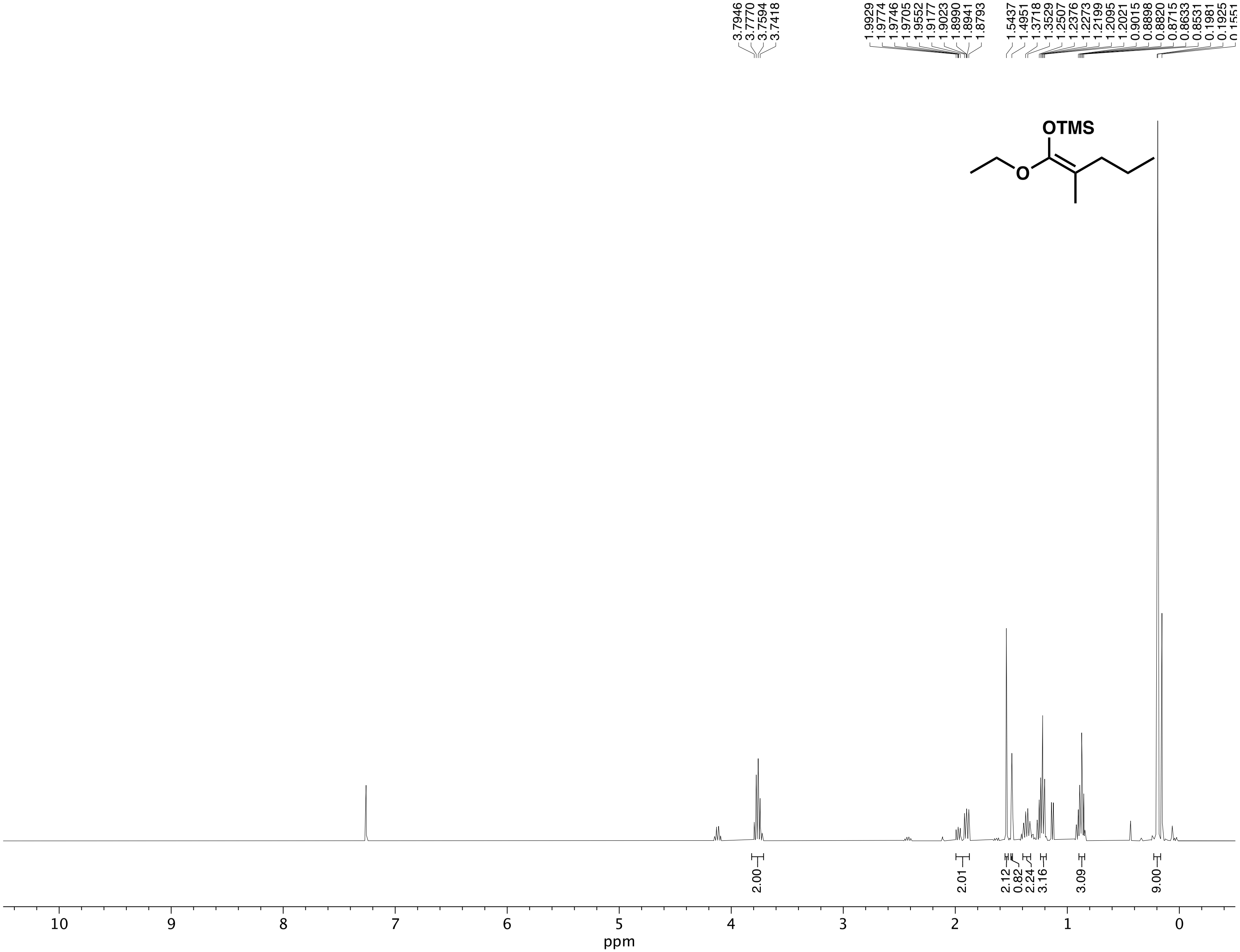

^{1}H NMR (400 MHz, $CDCl_3$) of compound **SI-18**.

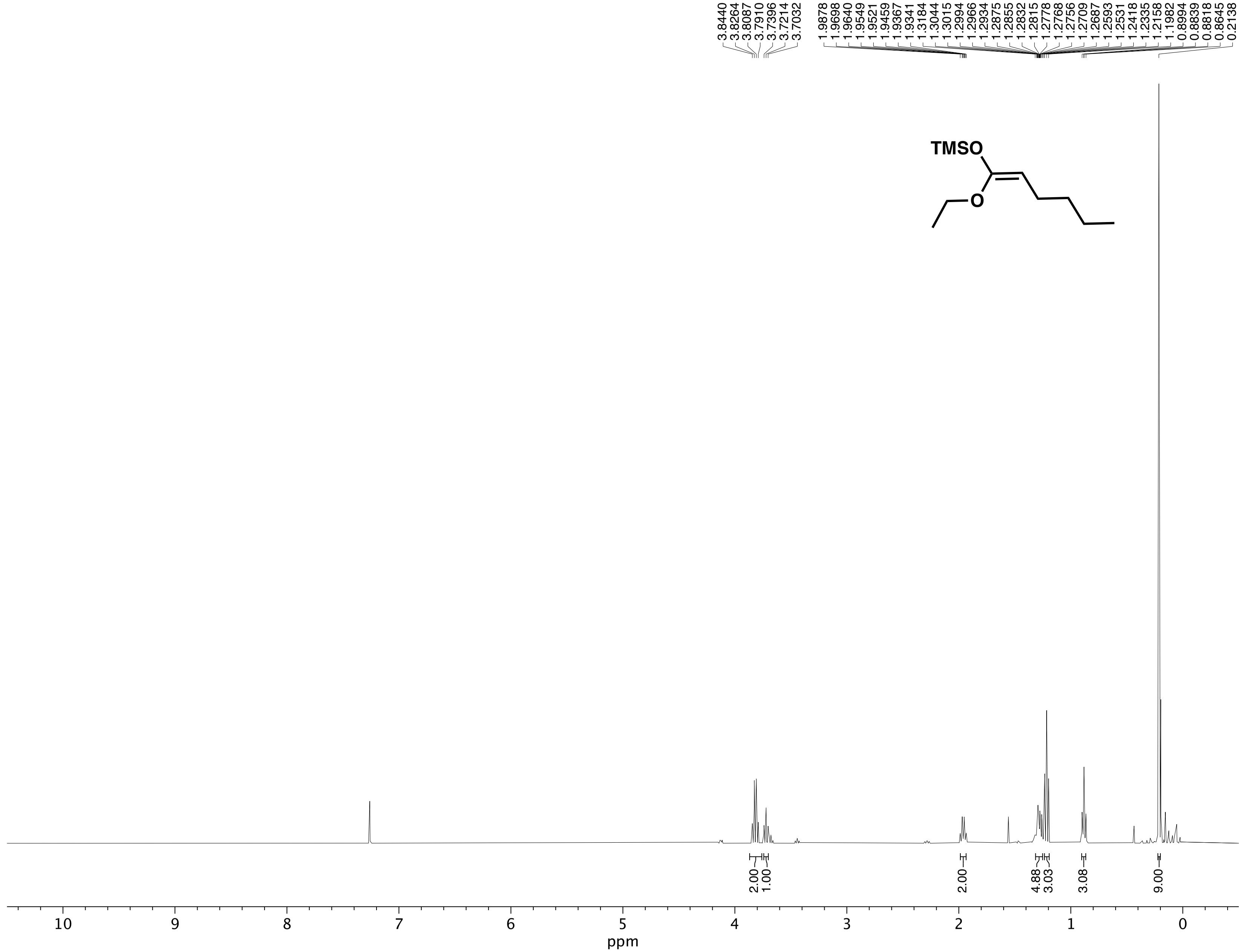

^{1}H NMR (400 MHz, $CDCl_3$) of compound **SI-19**.

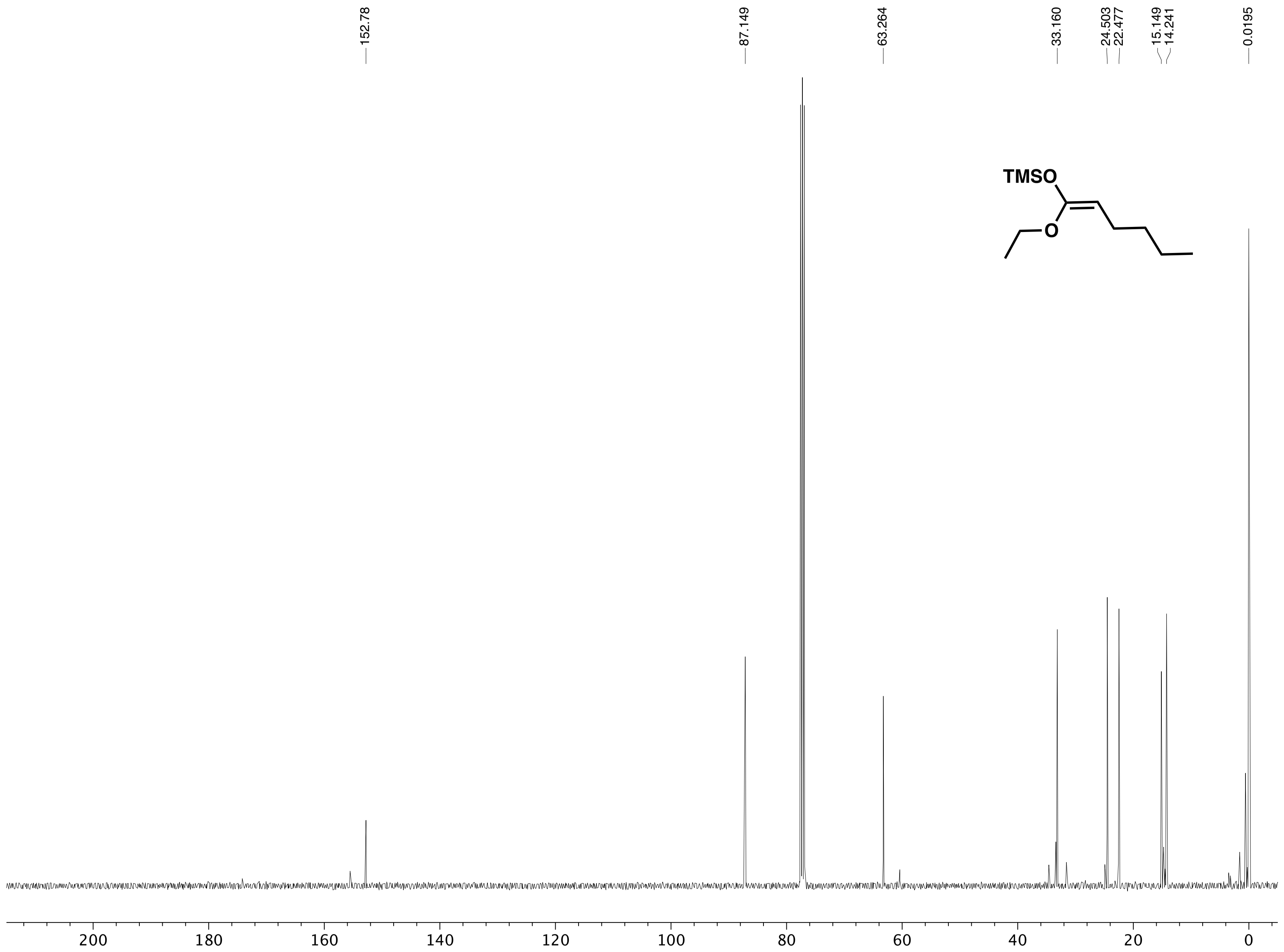

^{13}C NMR (101 MHz, $CDCl_3$) of compound **SI-19**.

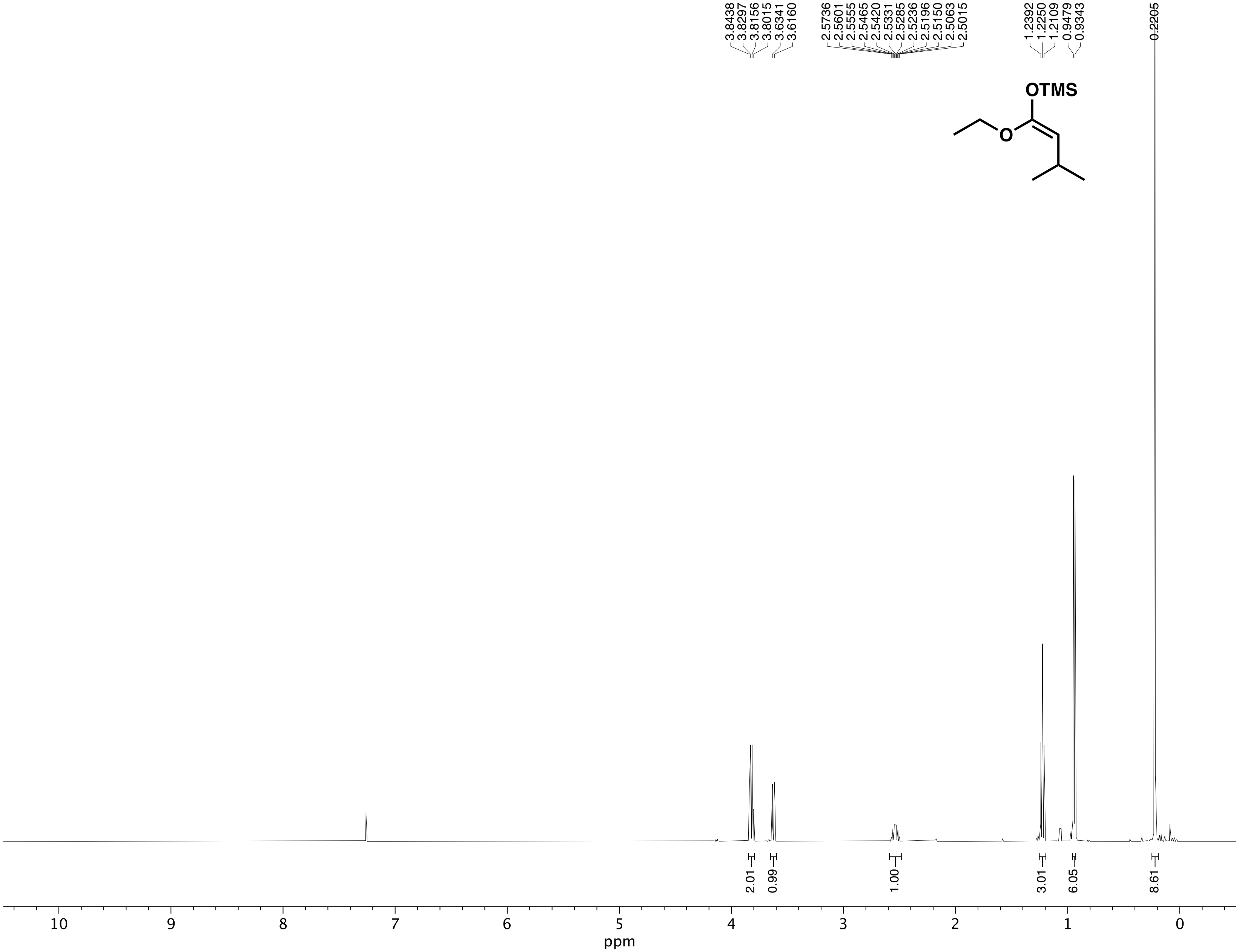

^{1}H NMR (400 MHz, $CDCl_3$) of compound **SI-20**.

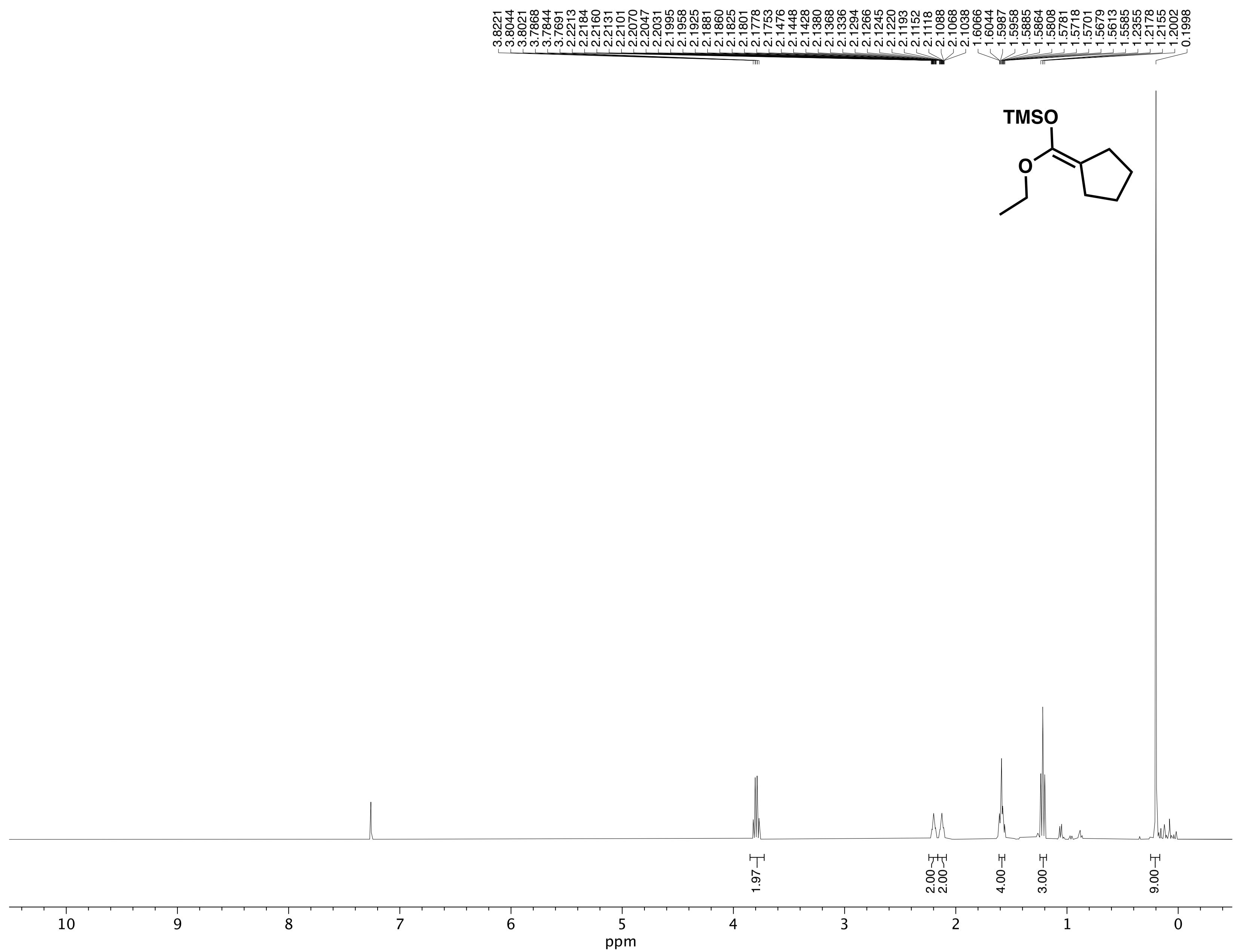

^{1}H NMR (400 MHz, $CDCl_3$) of compound **SI-21**.

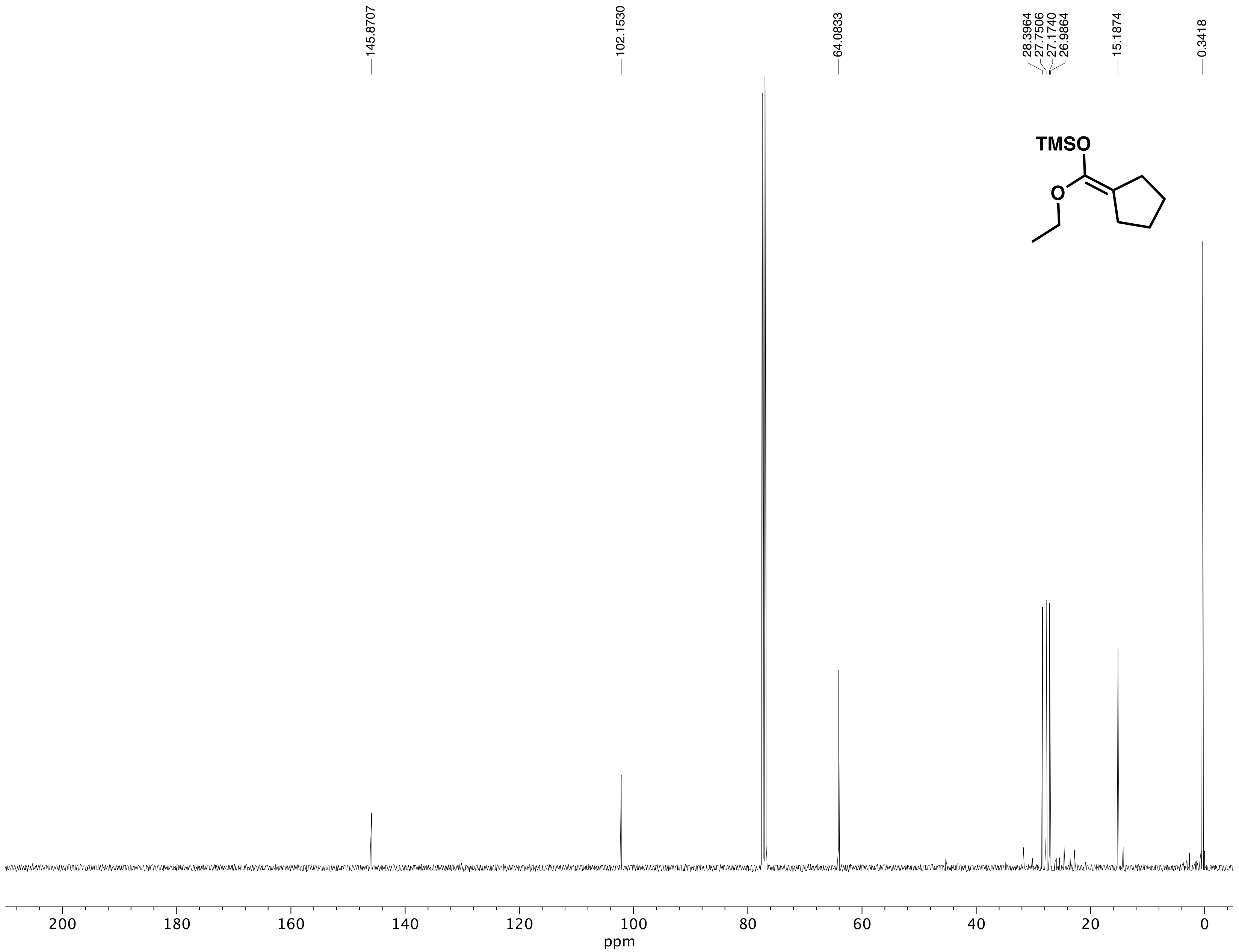

^{13}C NMR (101 MHz, $CDCl_3$) of compound **SI-21**.

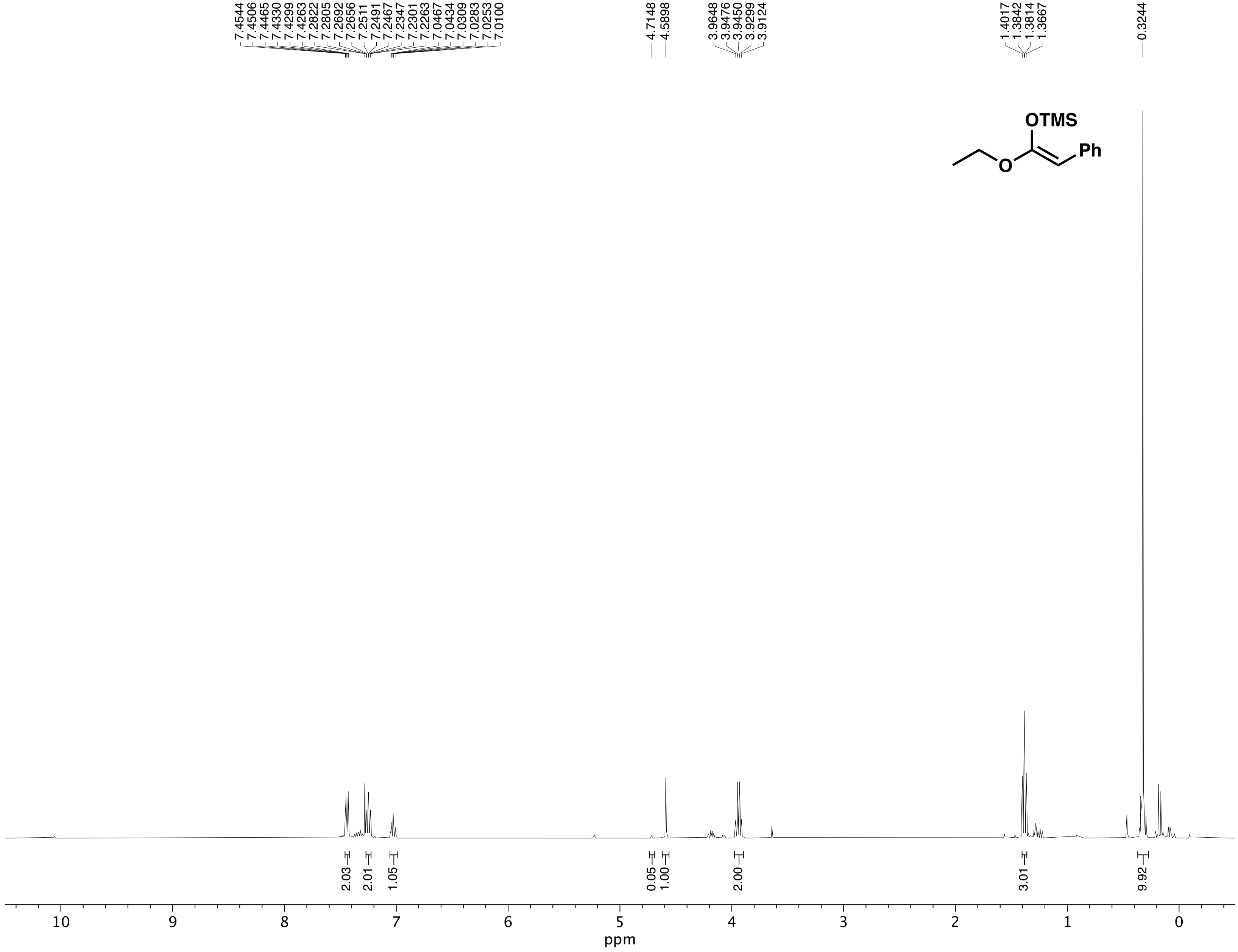

^{13}C NMR (101 MHz, $CDCl_3$) of compound **SI-22**.

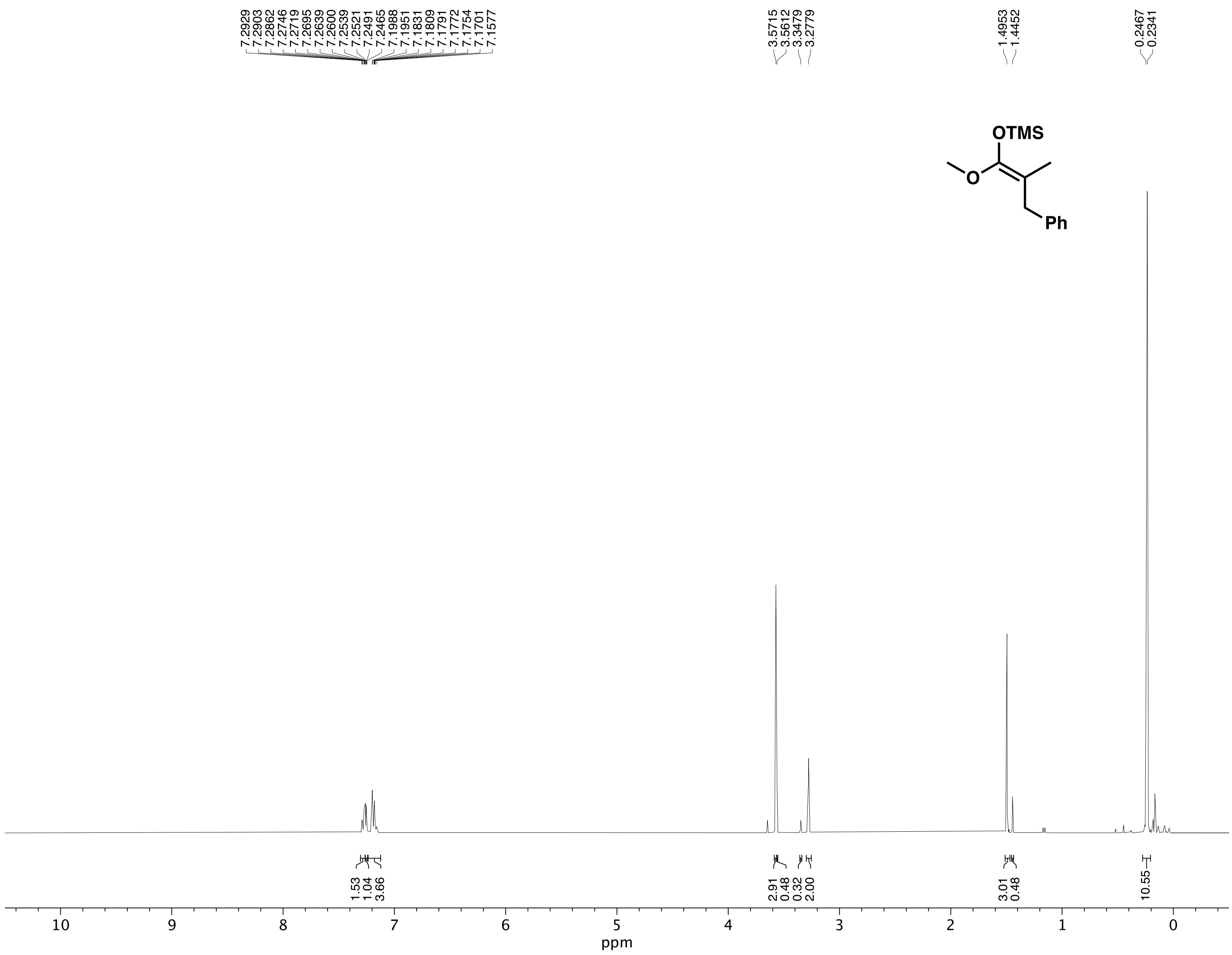

^{1}H NMR (400 MHz, $CDCl_3$) of compound **SI-23**.

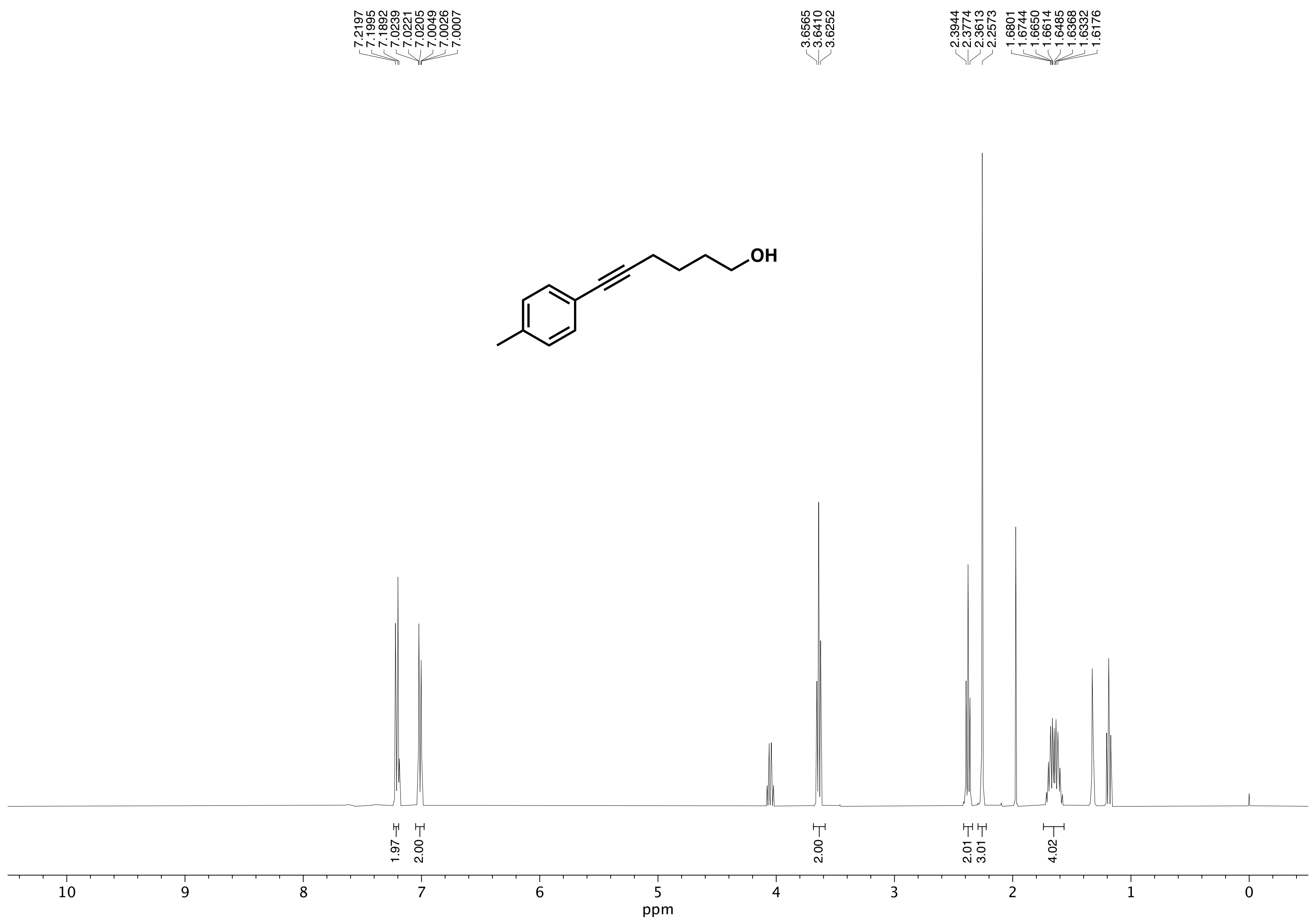

^{1}H NMR (400 MHz, $CDCl_3$) of compound **SI-24**.

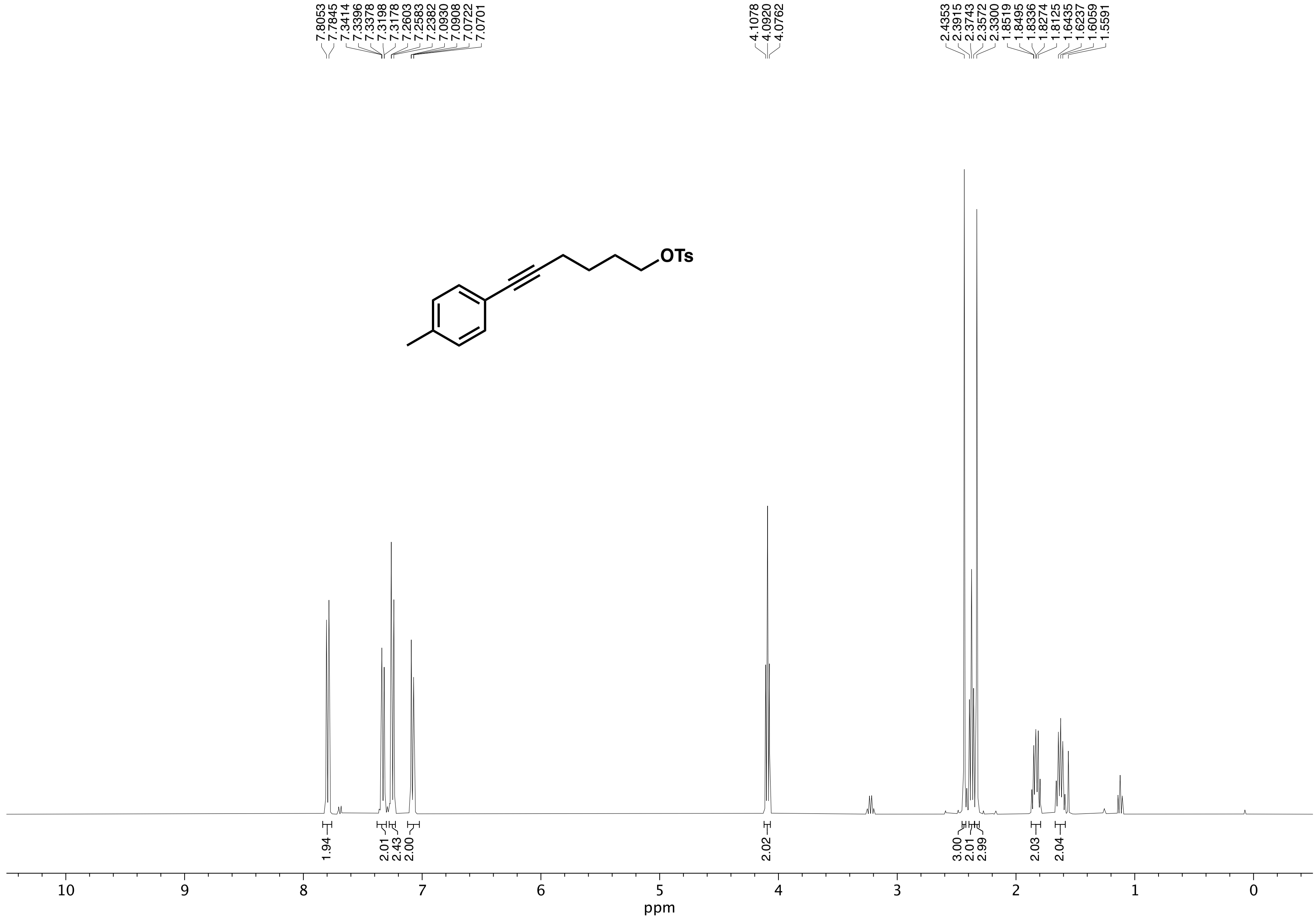

^{1}H NMR (400 MHz, $CDCl_3$) of compound **SI-25**.

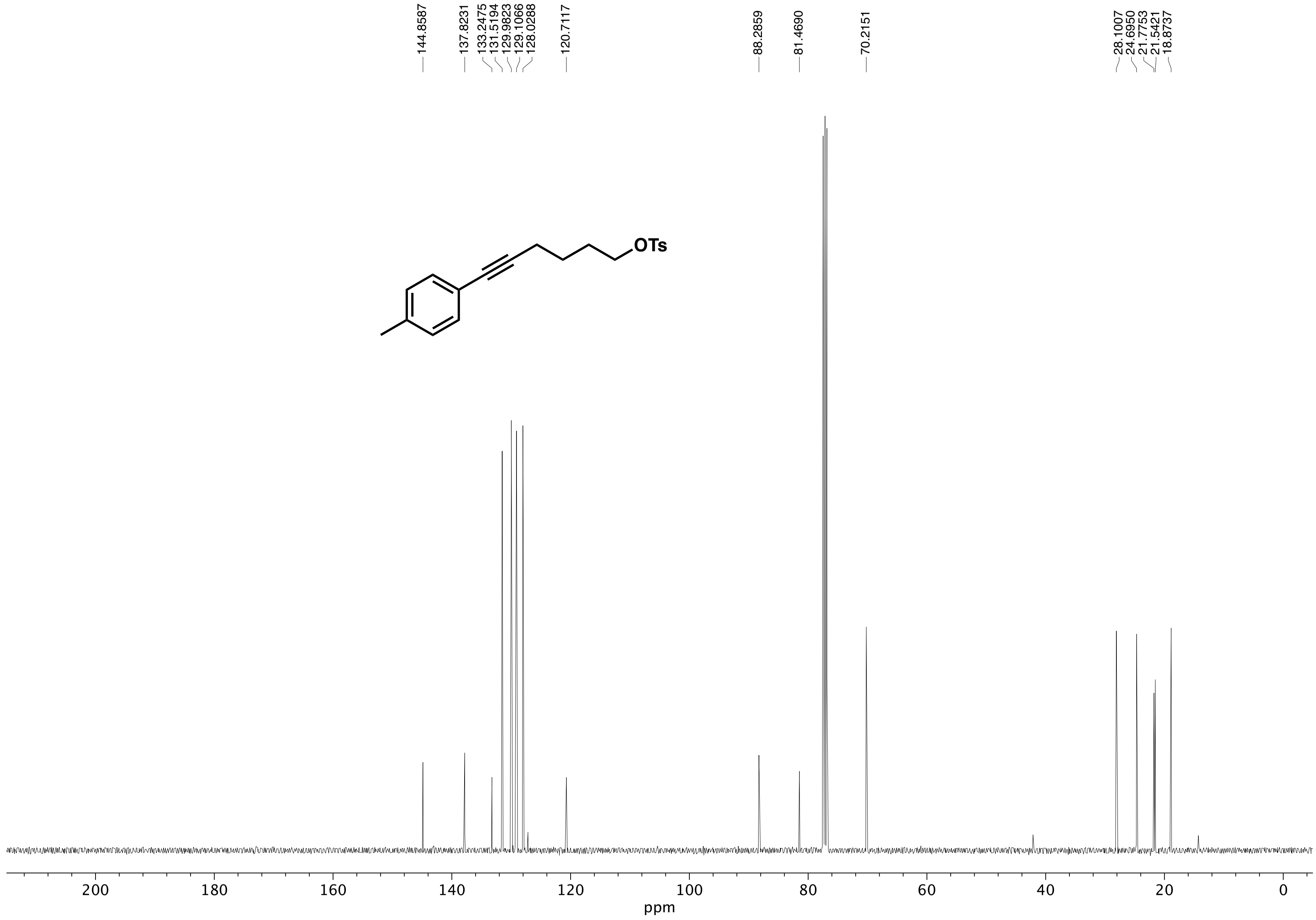

^{13}C NMR (101 MHz, $CDCl_3$) of compound **SI-25**.

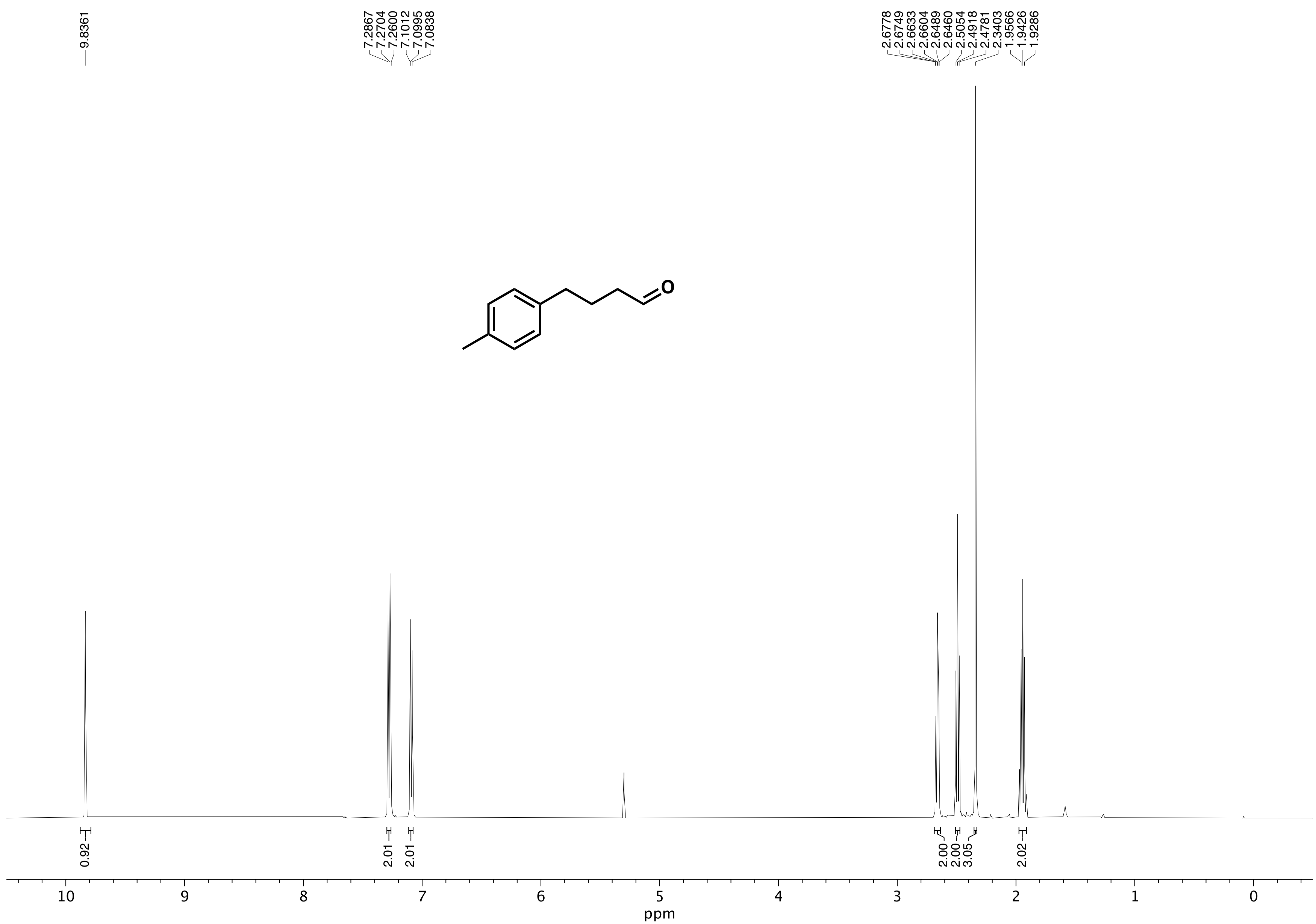

^{1}H NMR (400 MHz, $CDCl_3$) of compound **SI-26**.

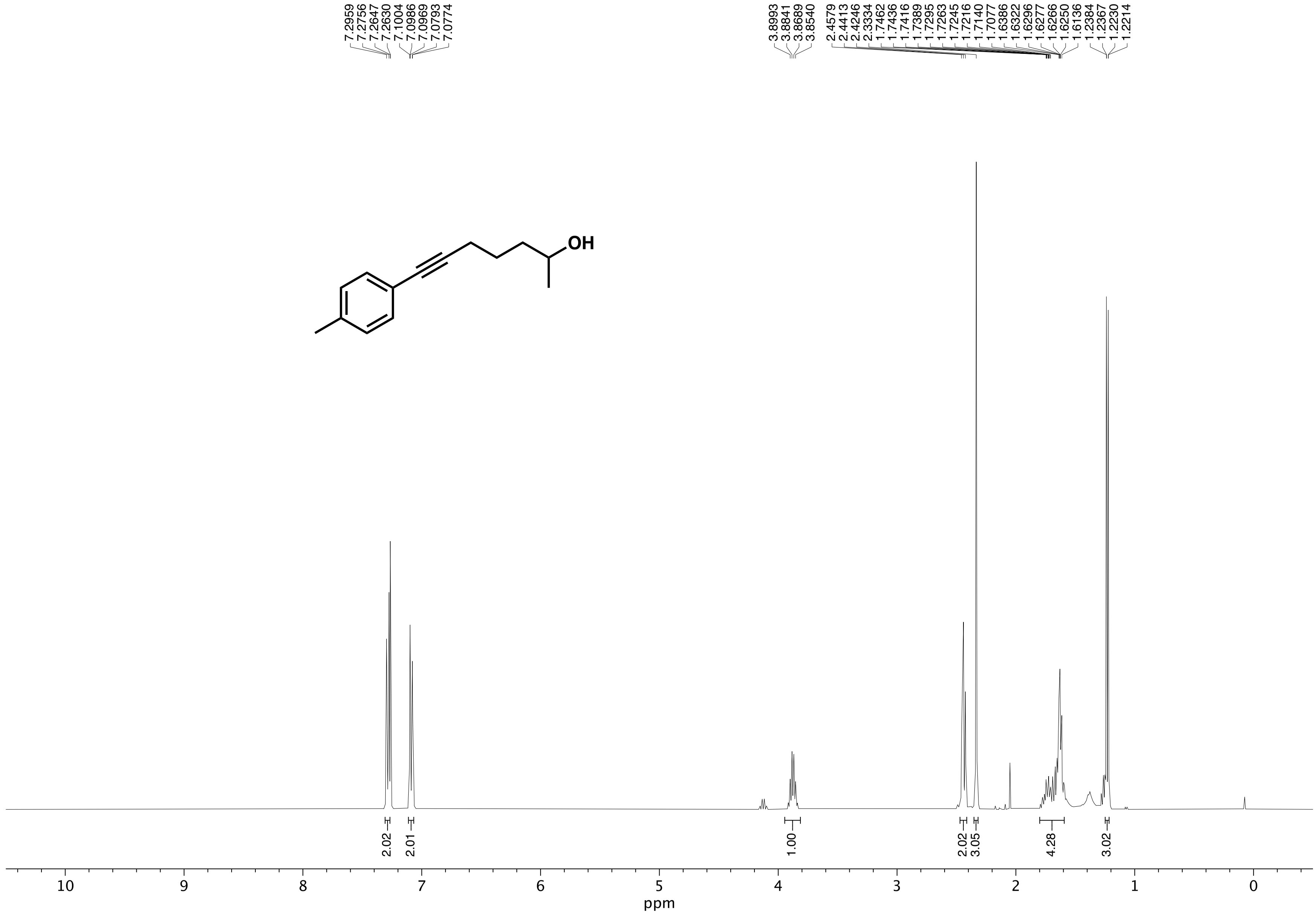

^{1}H NMR (400 MHz, $CDCl_3$) of compound **SI-27**.

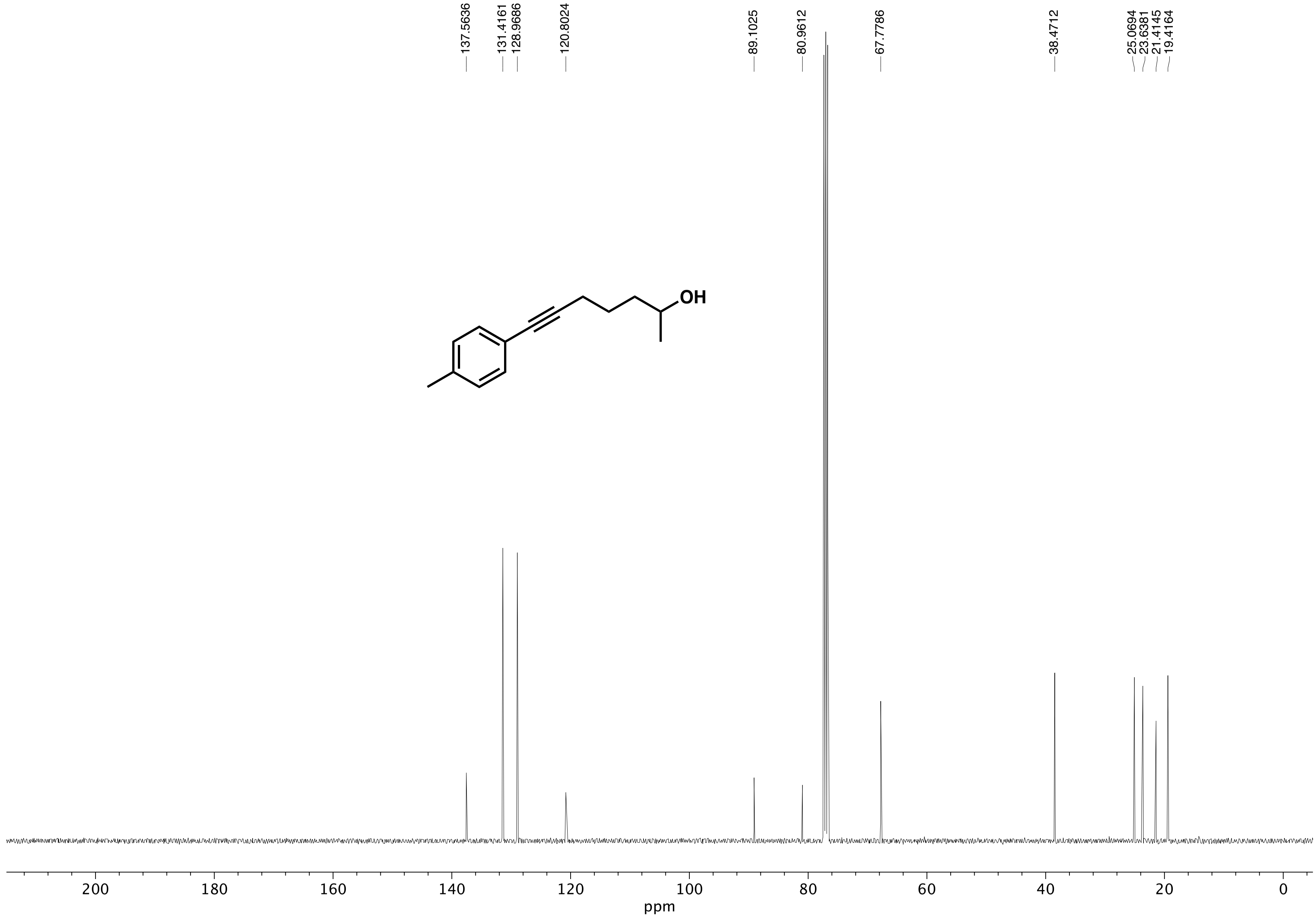

^{13}C NMR (101 MHz, $CDCl_3$) of compound **SI-27**.

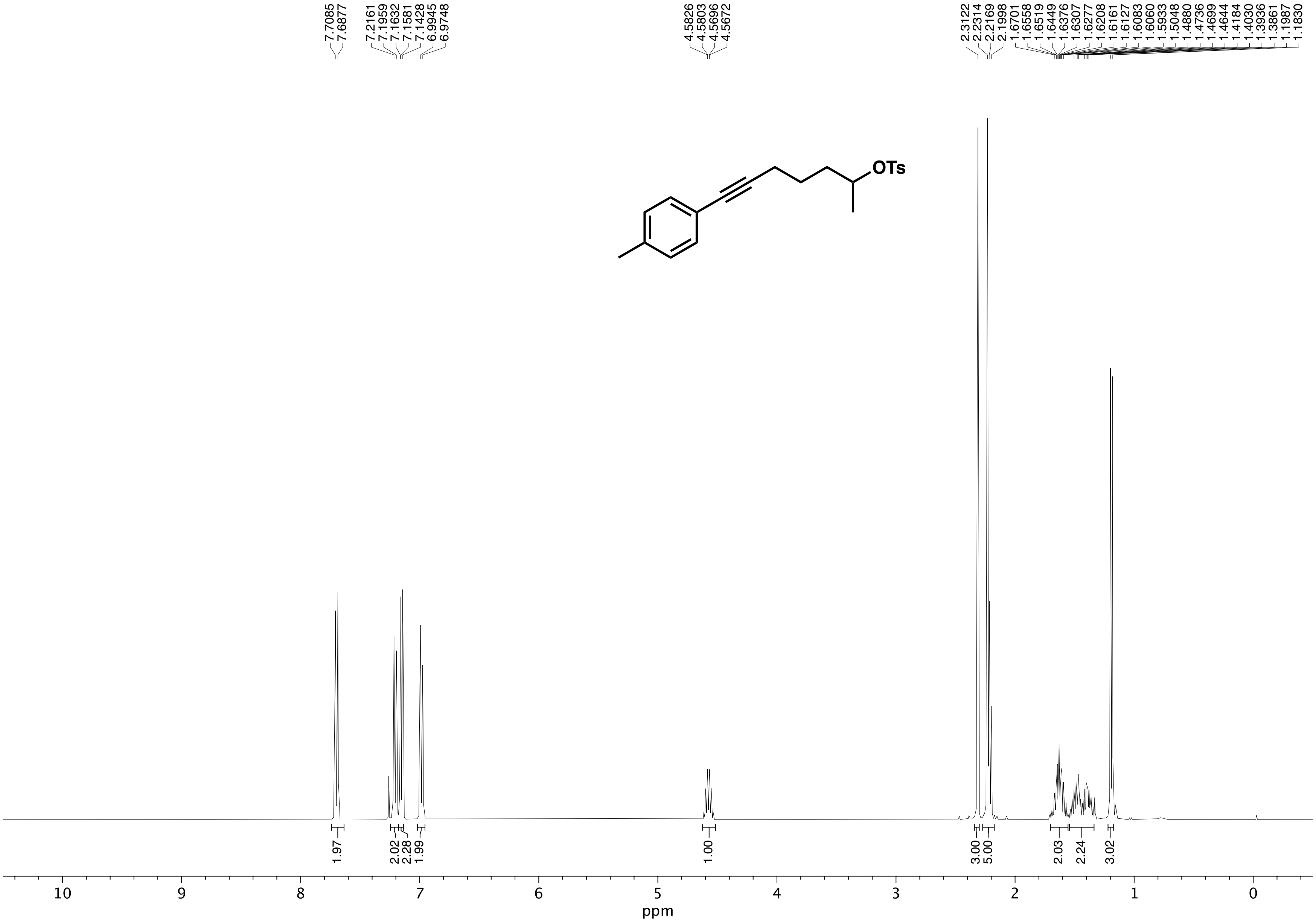

^{1}H NMR (400 MHz, $CDCl_3$) of compound **SI-28**.

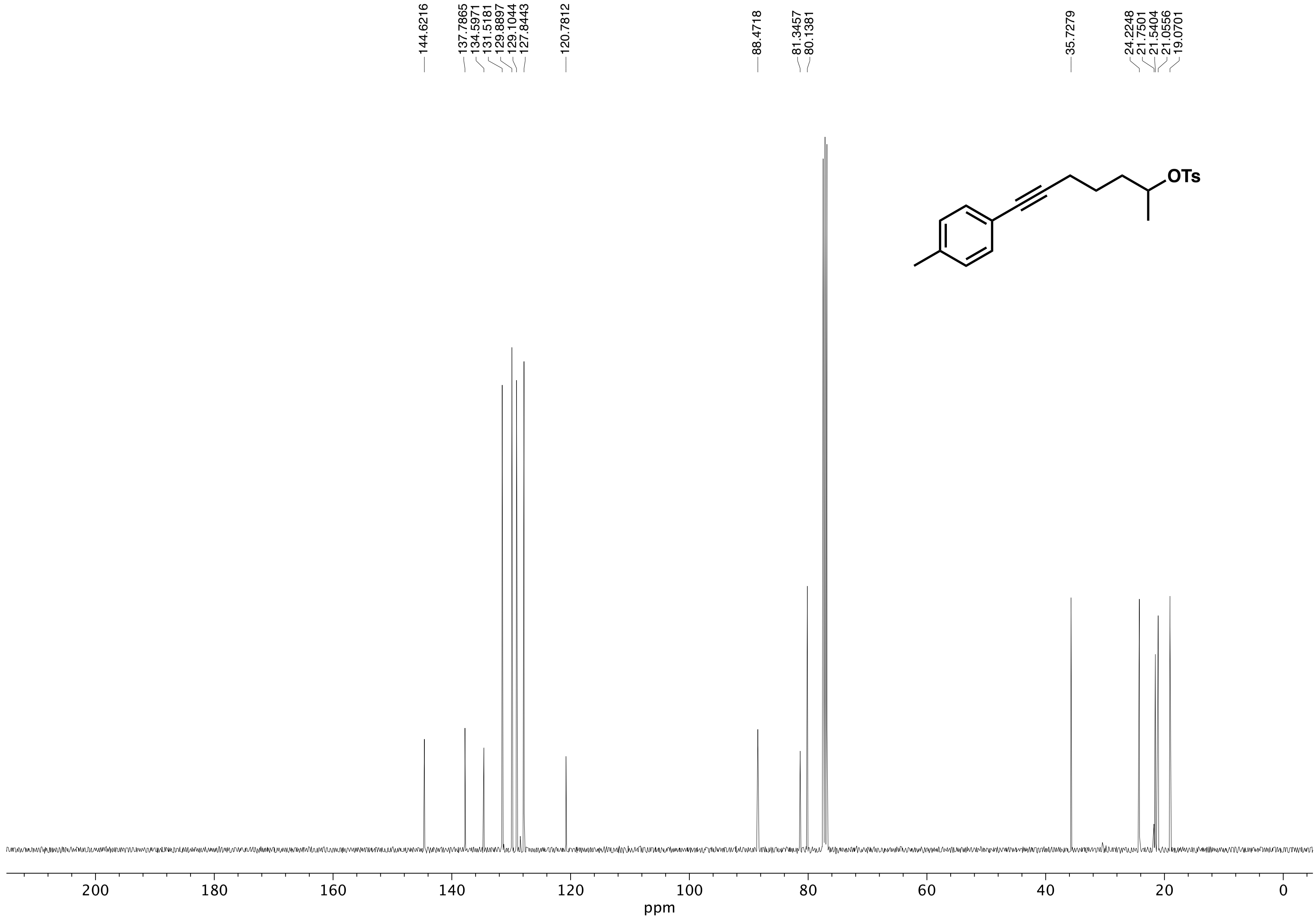

^{13}C NMR (101 MHz, $CDCl_3$) of compound **SI-28**.

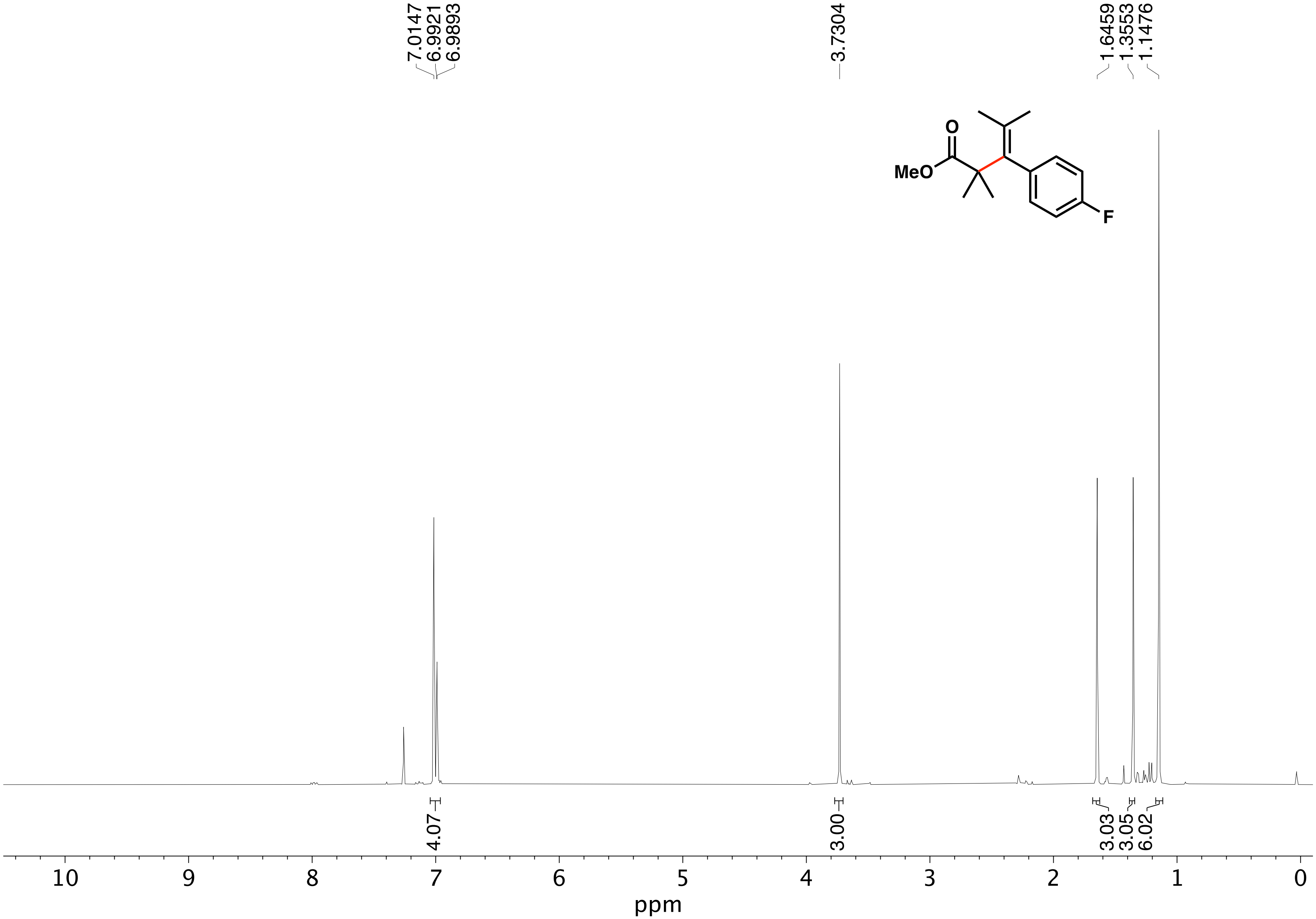

^{1}H NMR (300 MHz, $CDCl_3$) of compound **3a.**

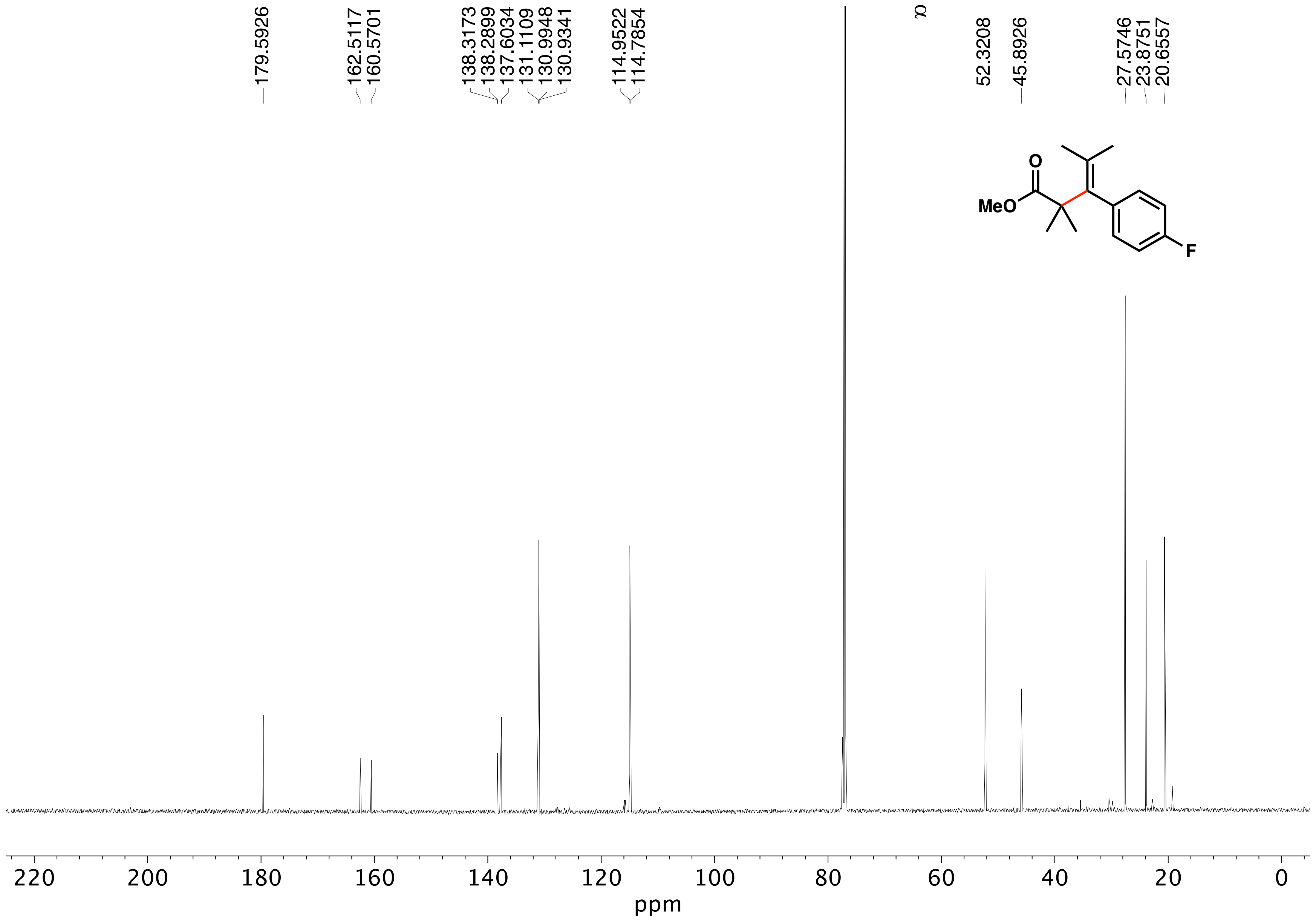

^{13}C NMR (126 MHz, $CDCl_3$) of compound **3a**.

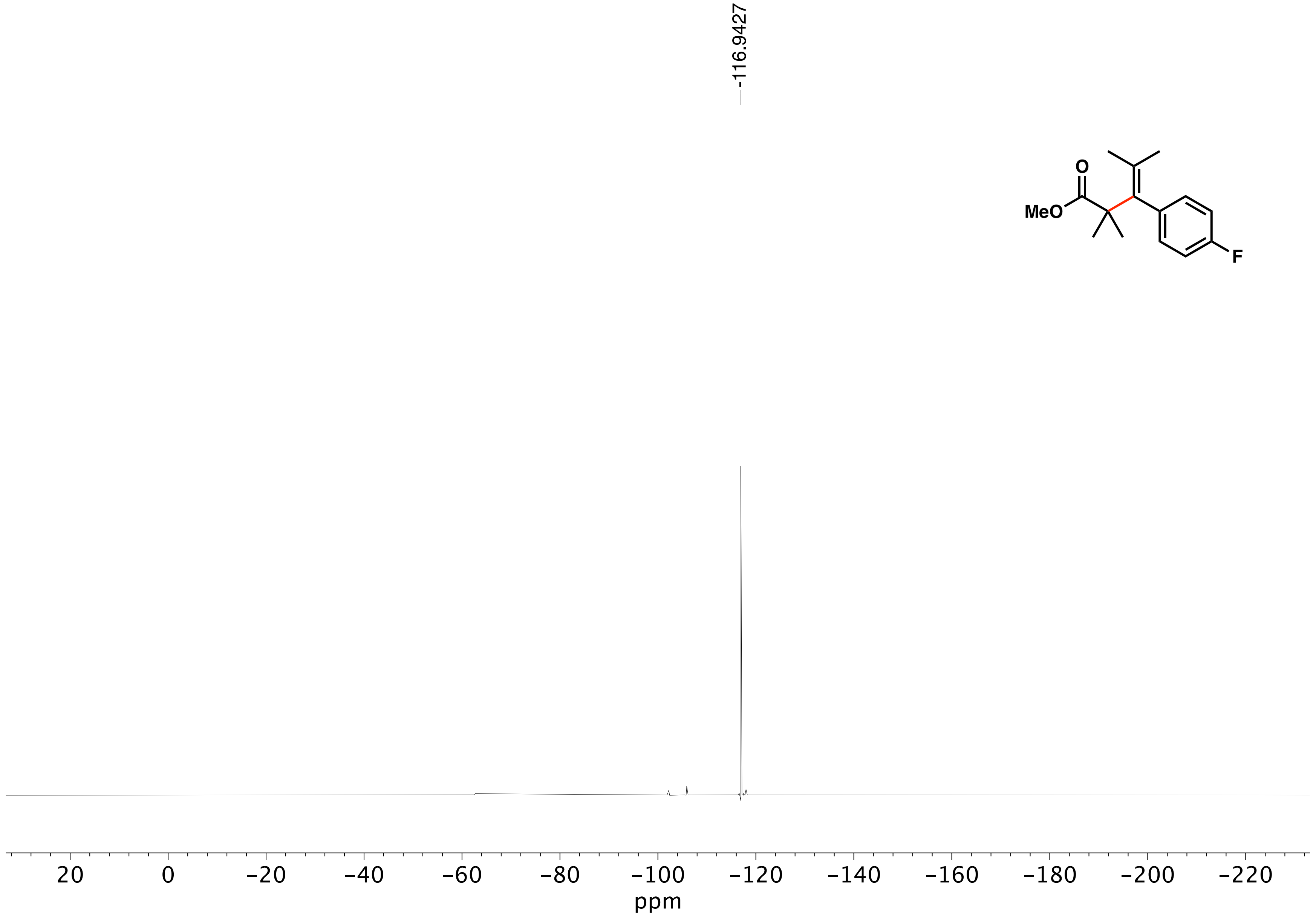

^{19}F NMR (282 MHz, $CDCl_3$) of compound **3a**.

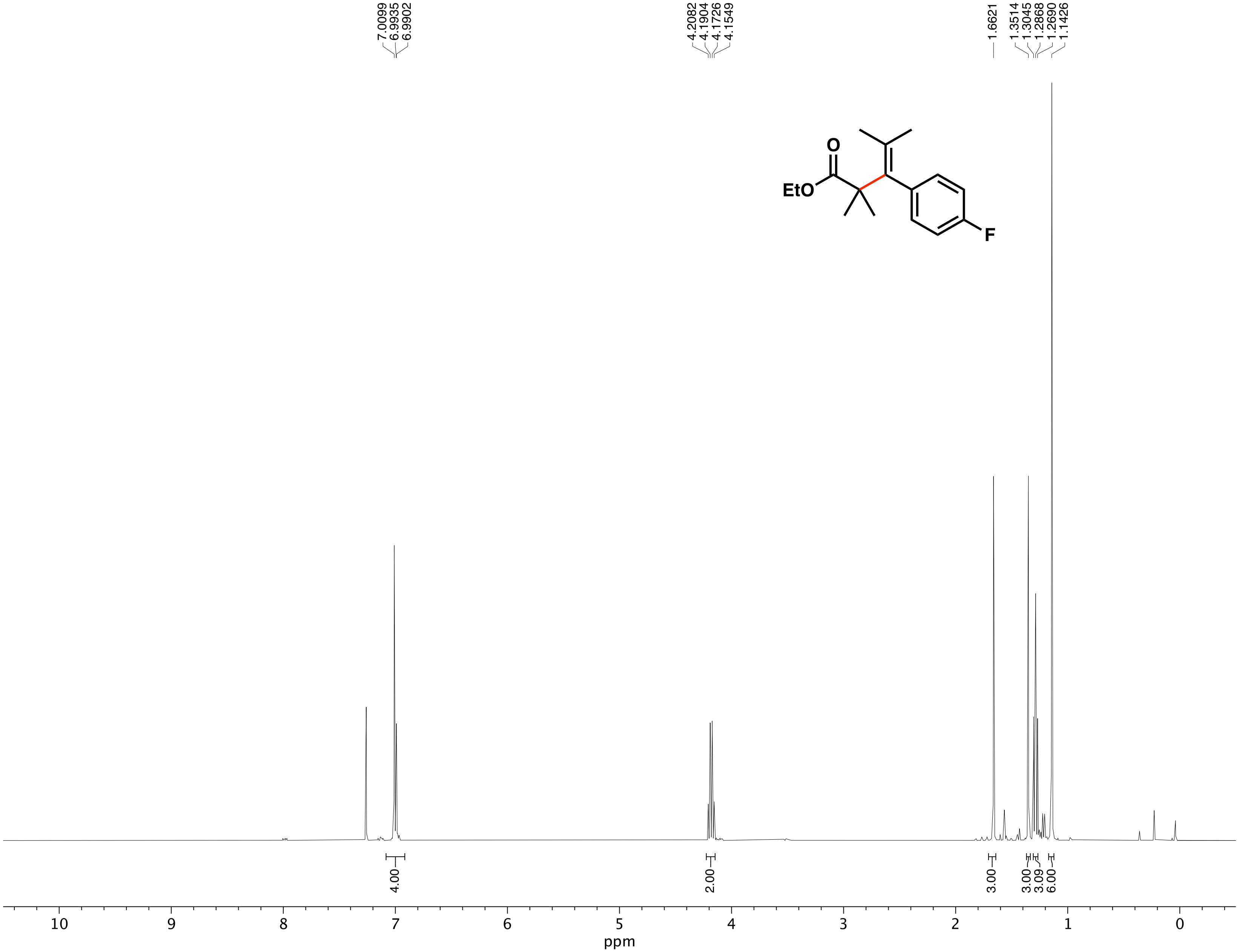

^1H NMR (400 MHz, $CDCl_3$) of compound **3b.**

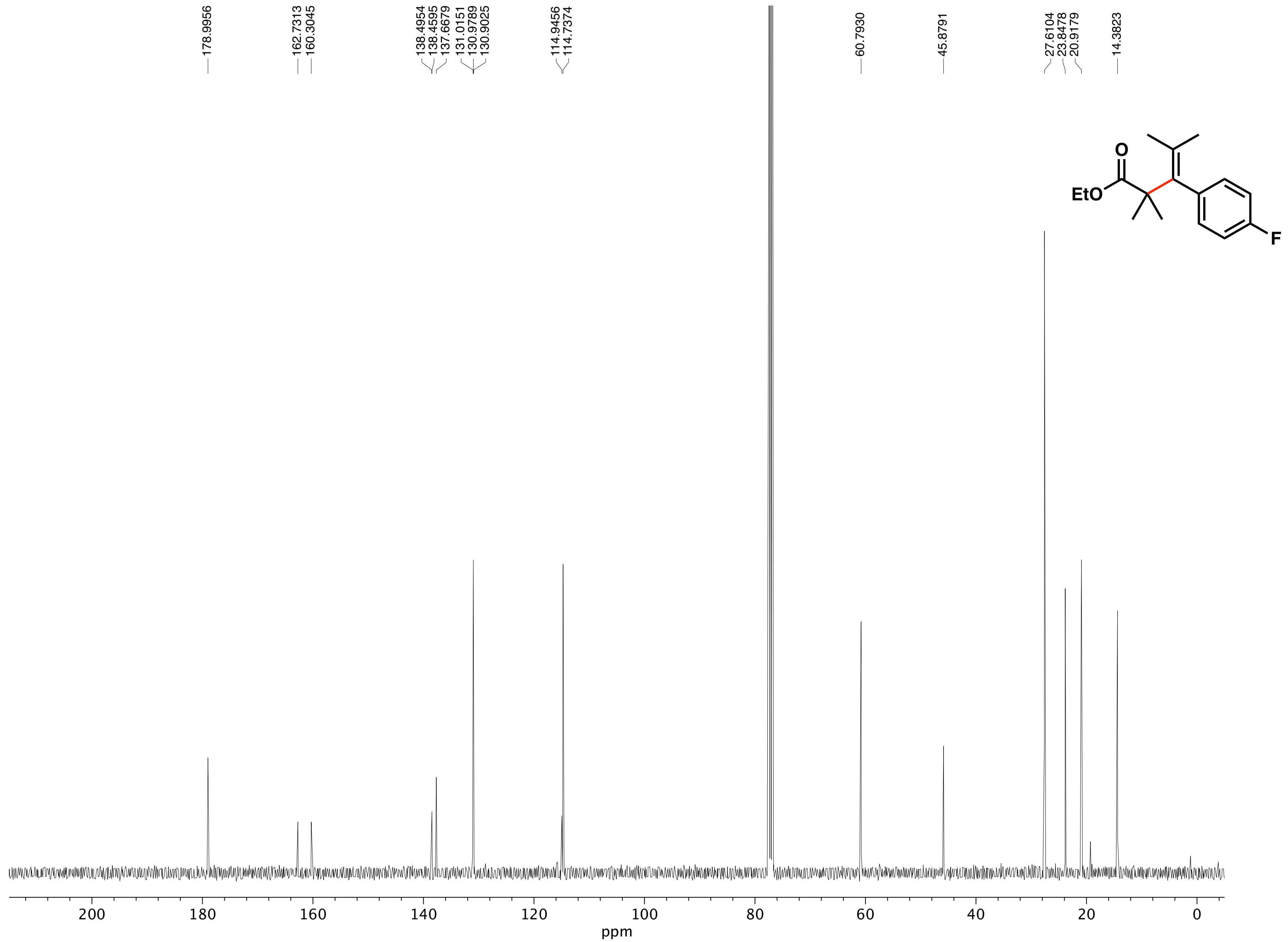

^{13}C NMR (101 MHz, $CDCl_3$) of compound **3b**.

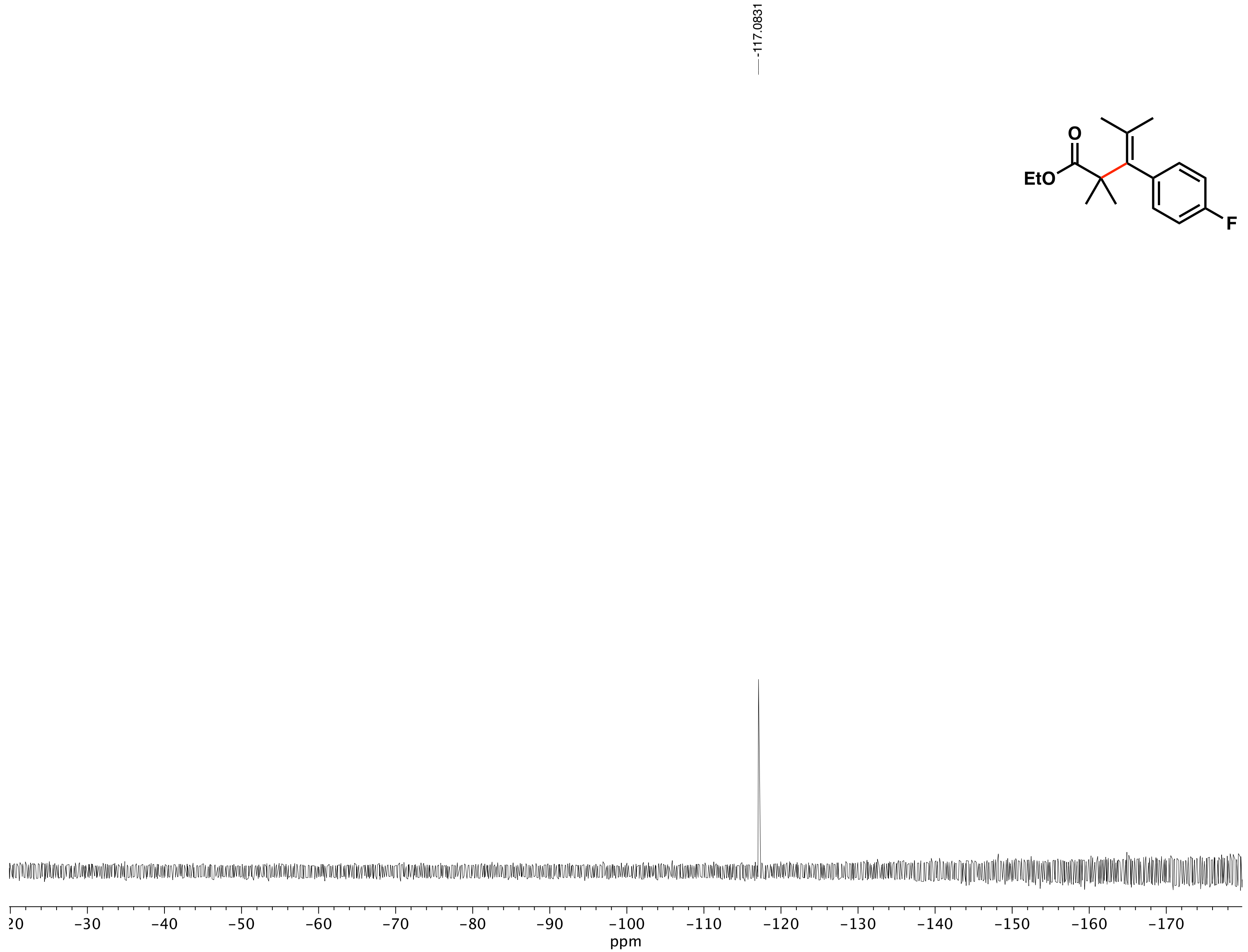

^{19}F NMR (282 MHz, $CDCl_3$) of compound **3b**.

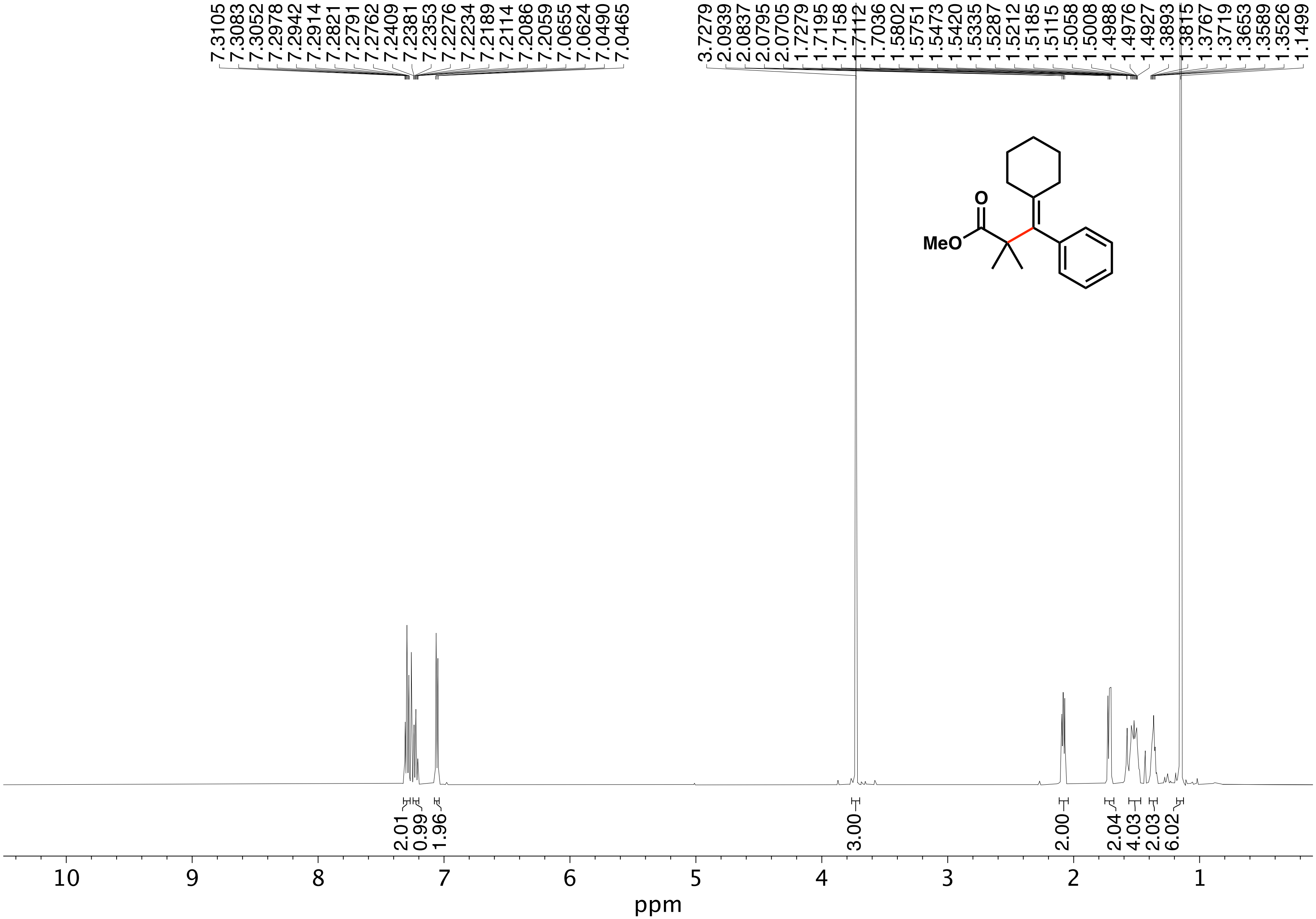

^{1}H NMR (300 MHz, $CDCl_3$) of compound **4.**

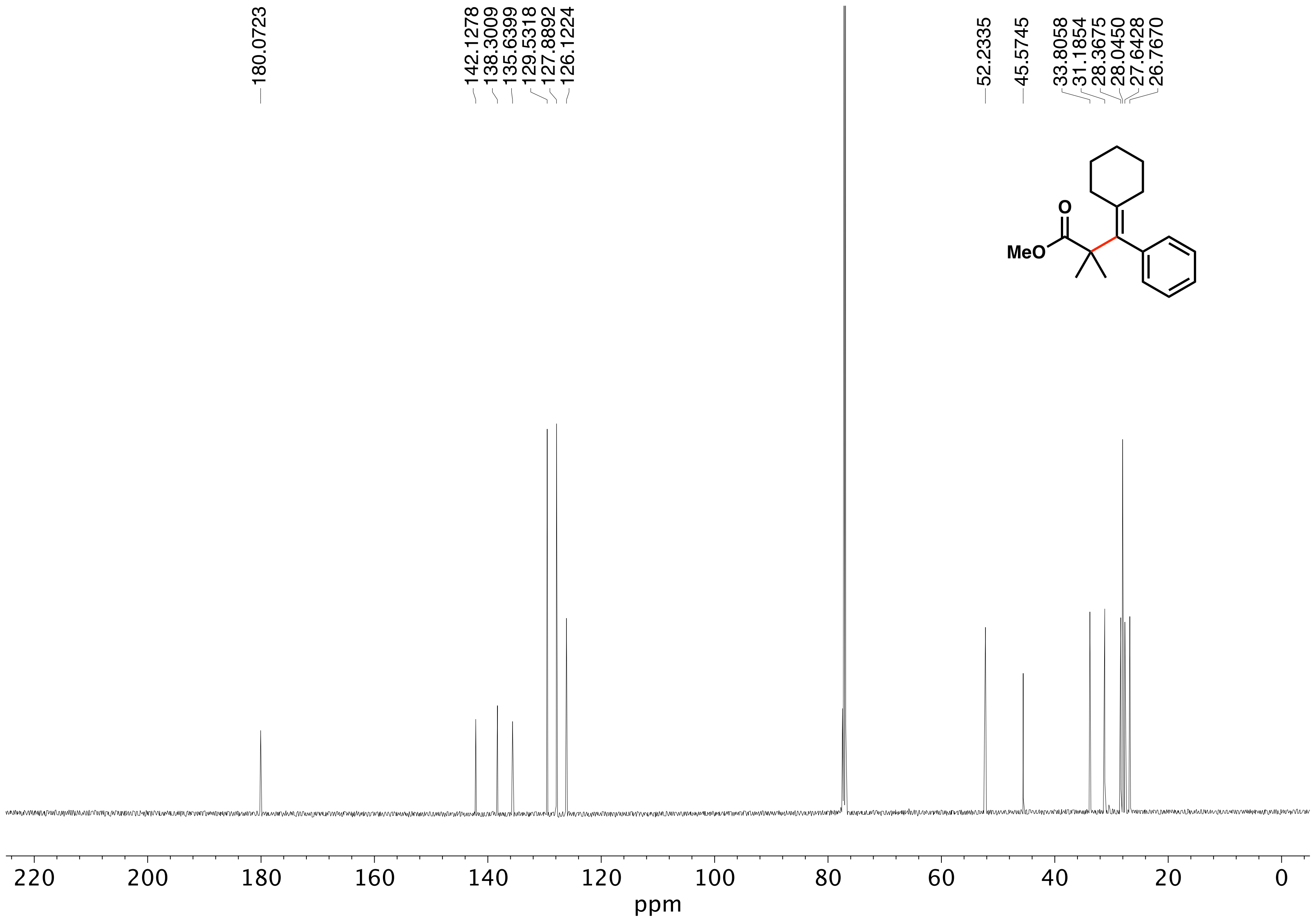

^{1}H NMR (500 MHz, $CDCl_3$) of compound **4.**

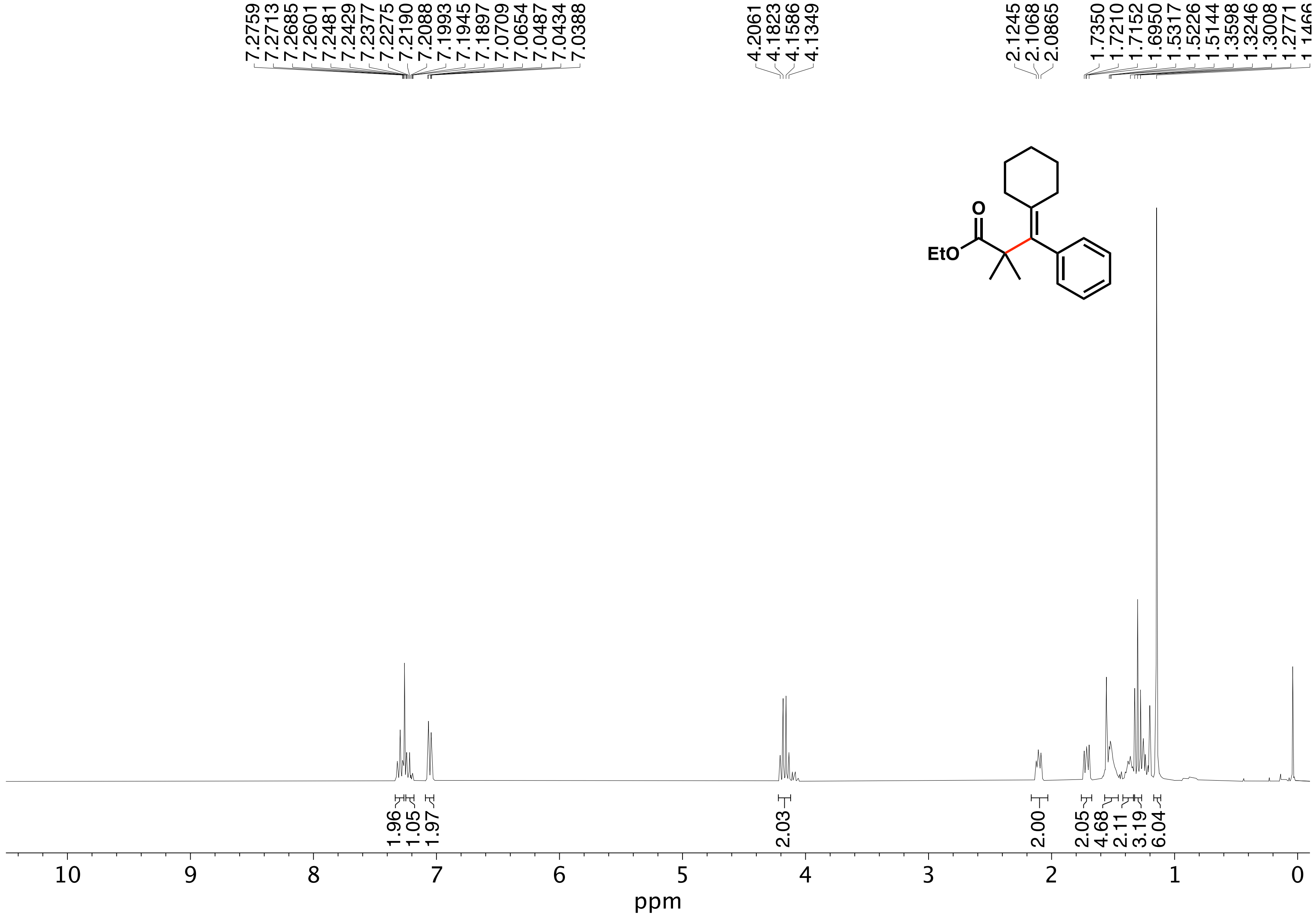

^{1}H NMR (300 MHz, $CDCl_3$) of compound **5**.

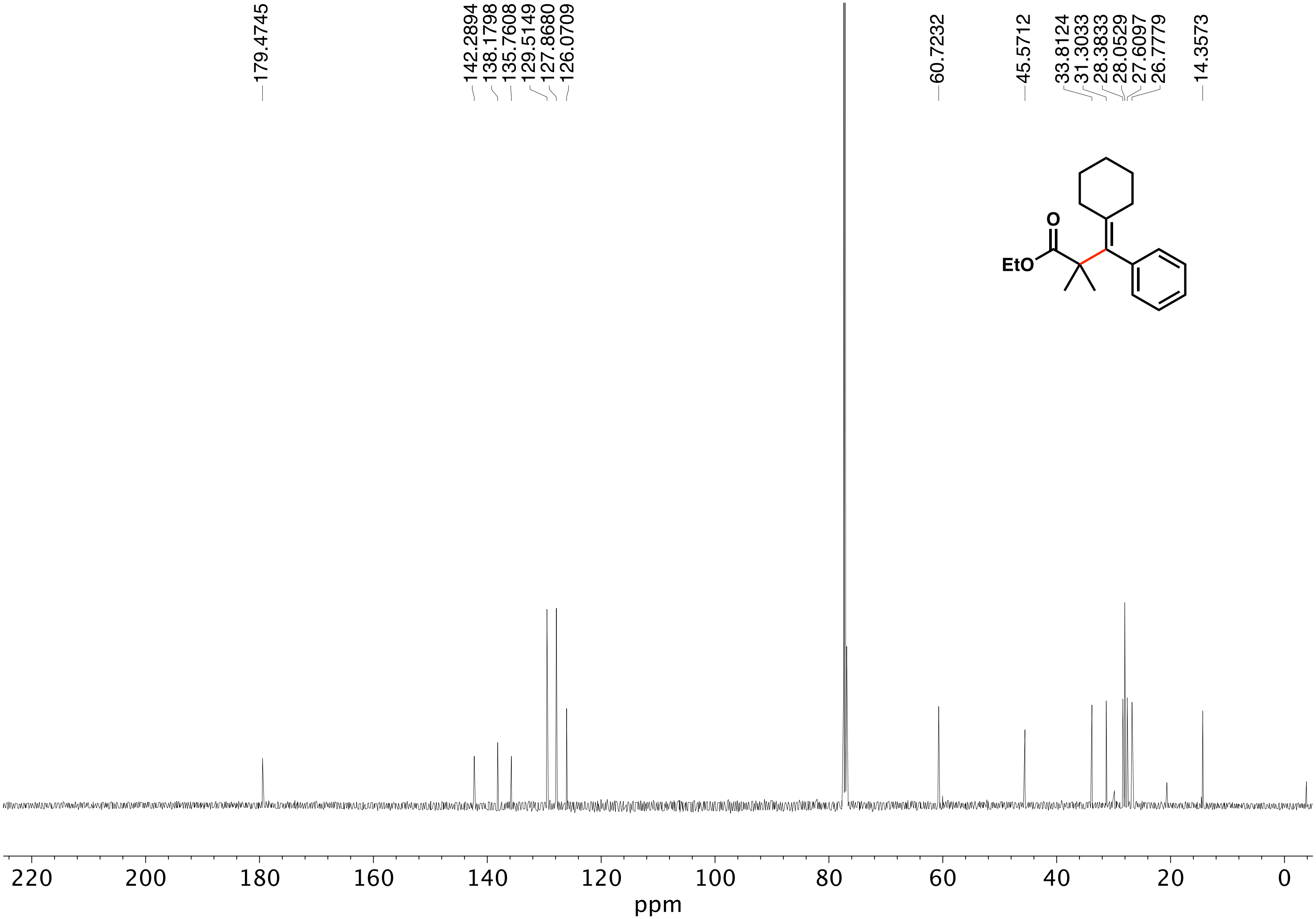

^{1}H NMR (500 MHz, $CDCl_3$) of compound **5**.

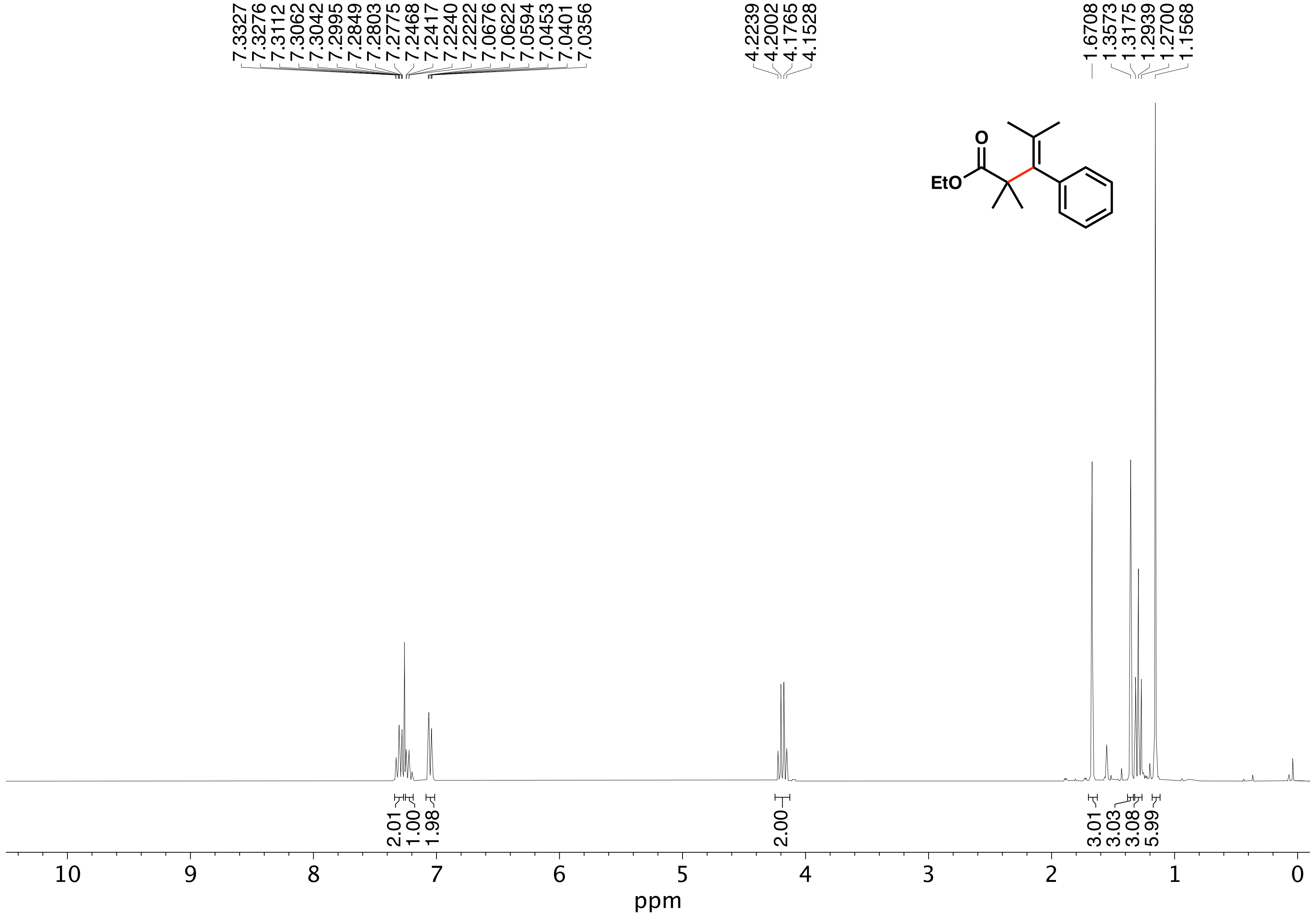

^{1}H NMR (300 MHz, $CDCl_3$) of compound **6**.

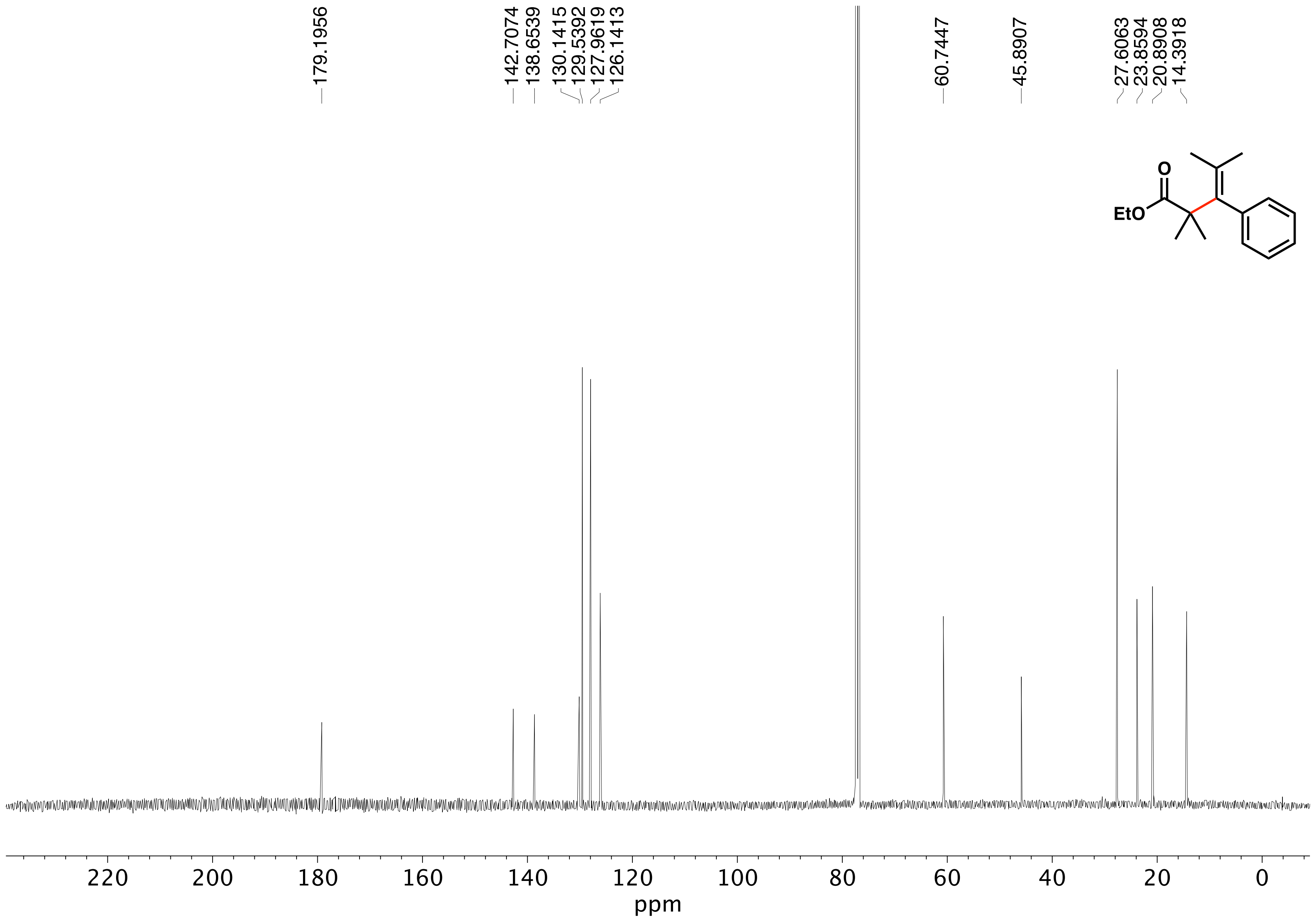

^{13}C NMR (500 MHz, $CDCl_3$) of compound **6**.

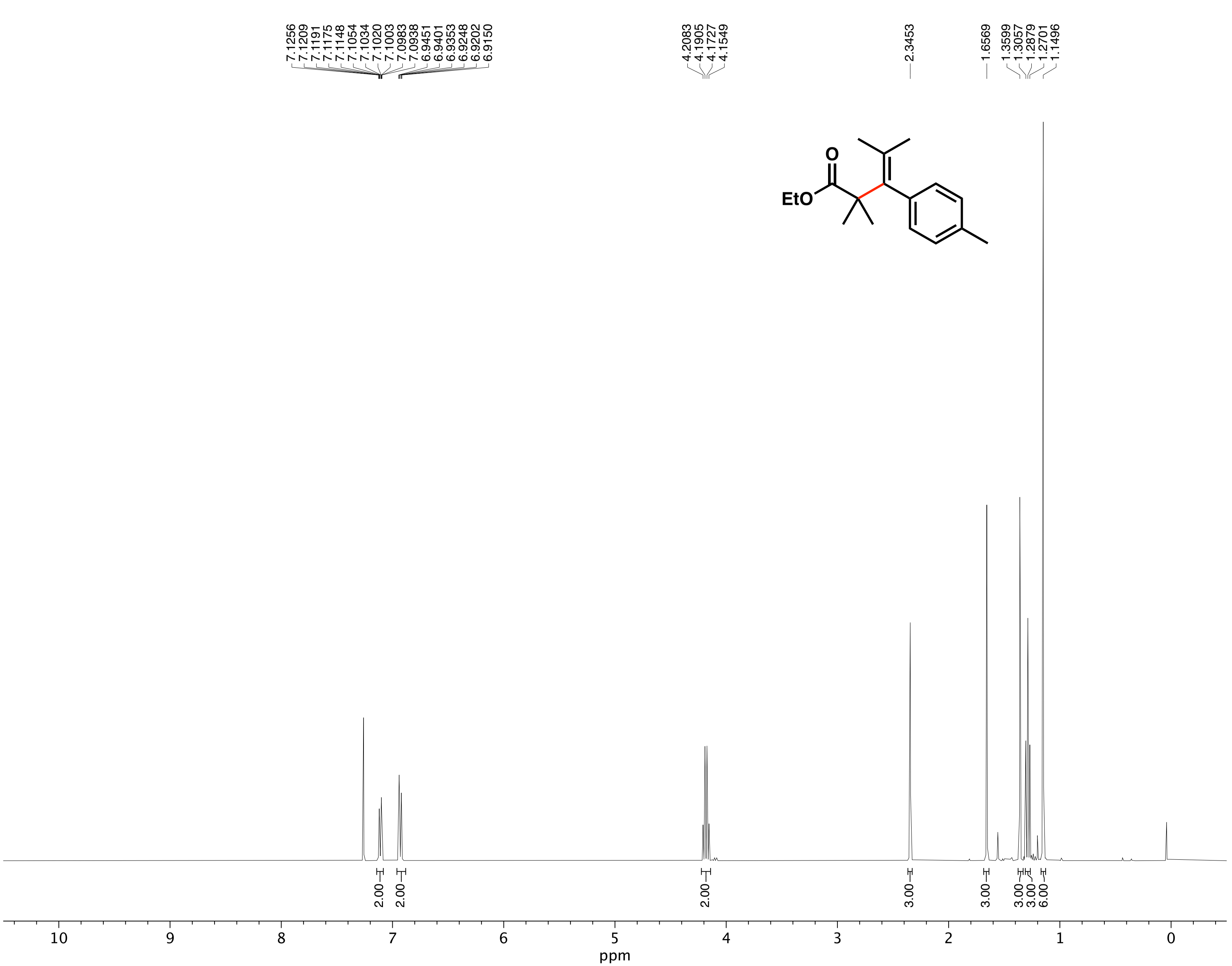

^{1}H NMR (400 MHz, $CDCl_3$) of compound **7**.

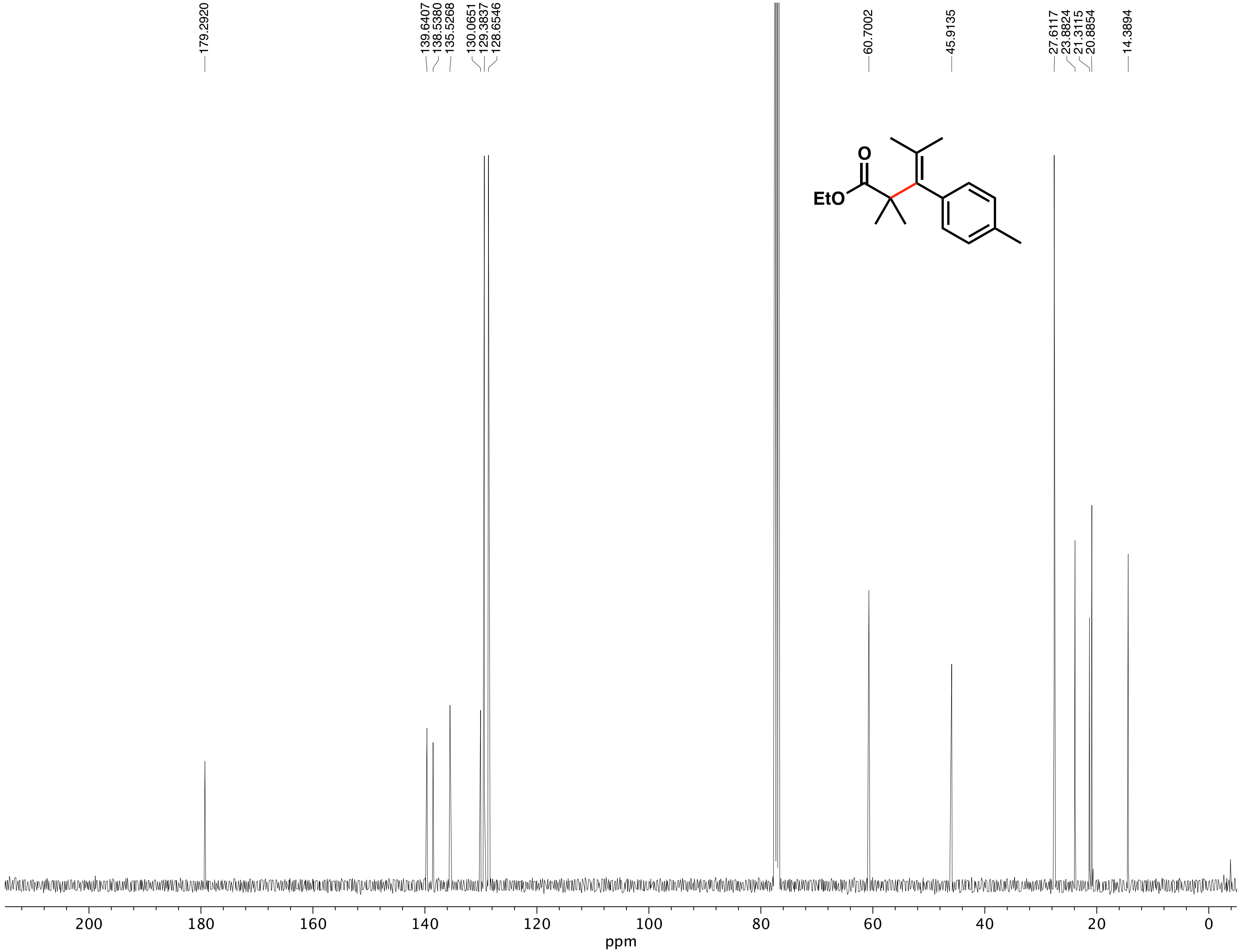

^{13}C NMR (400 MHz, $CDCl_3$) of compound **7**.

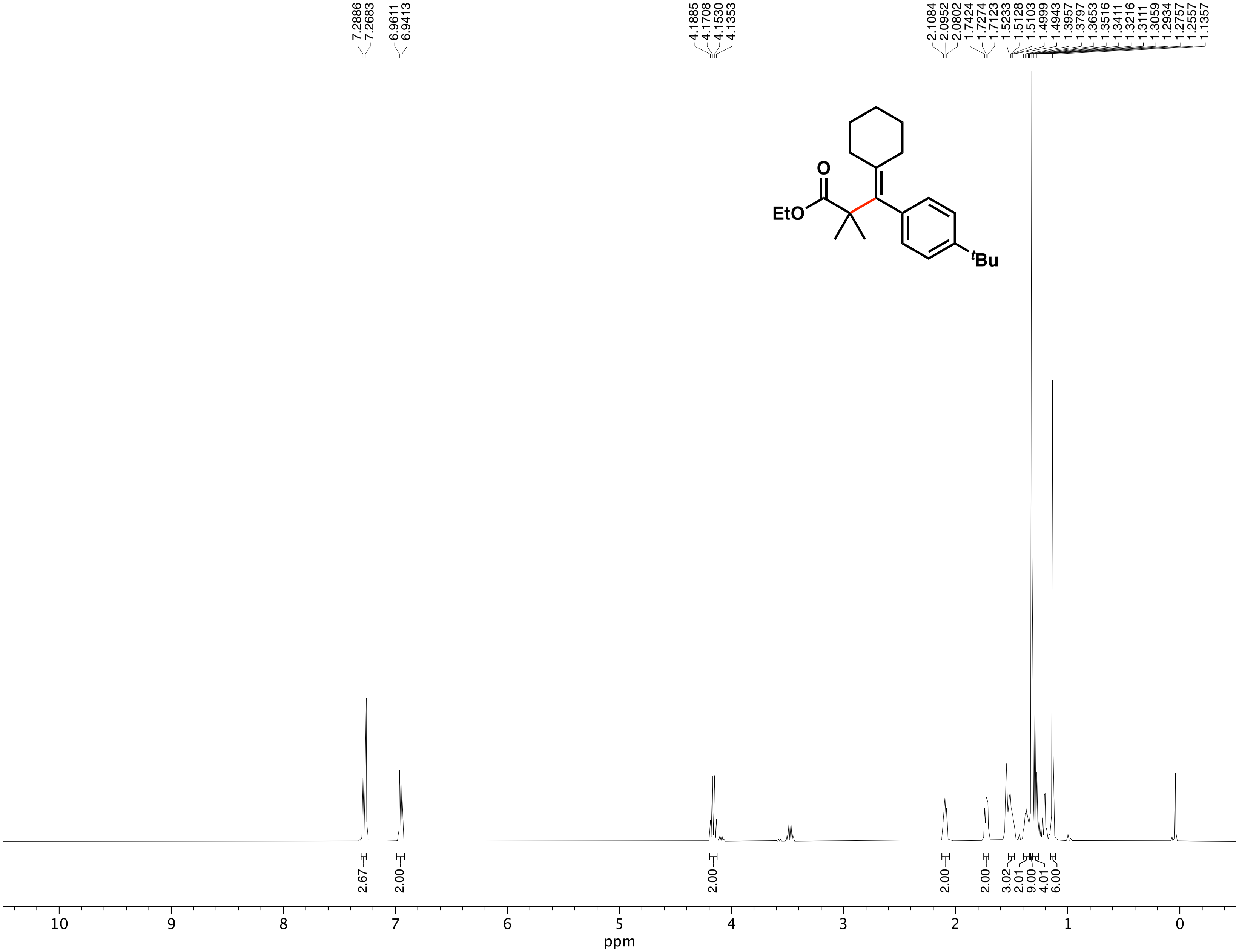

^{1}H NMR (400 MHz, $CDCl_3$) of compound **8**.

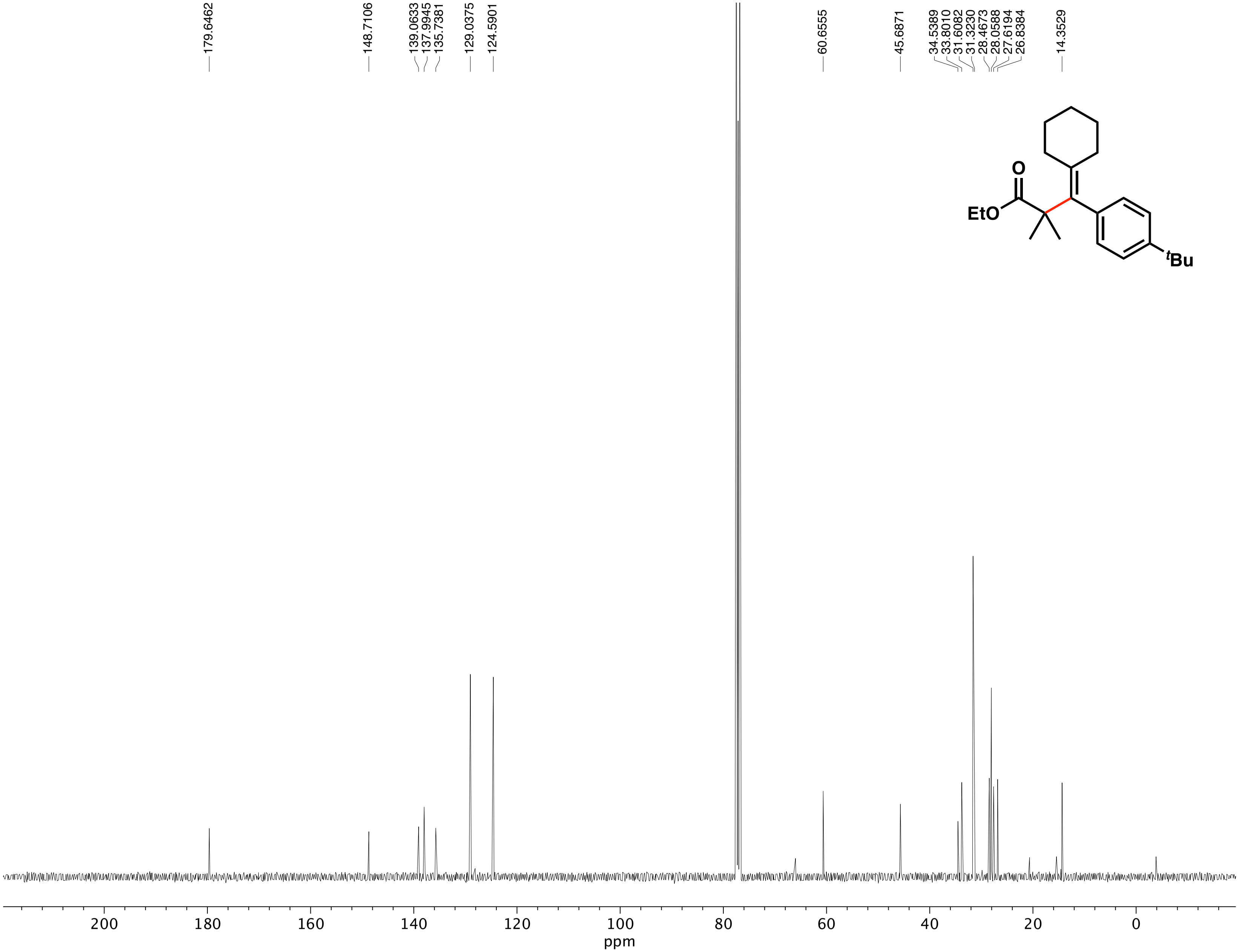

^{13}C NMR (400 MHz, $CDCl_3$) of compound **8**.

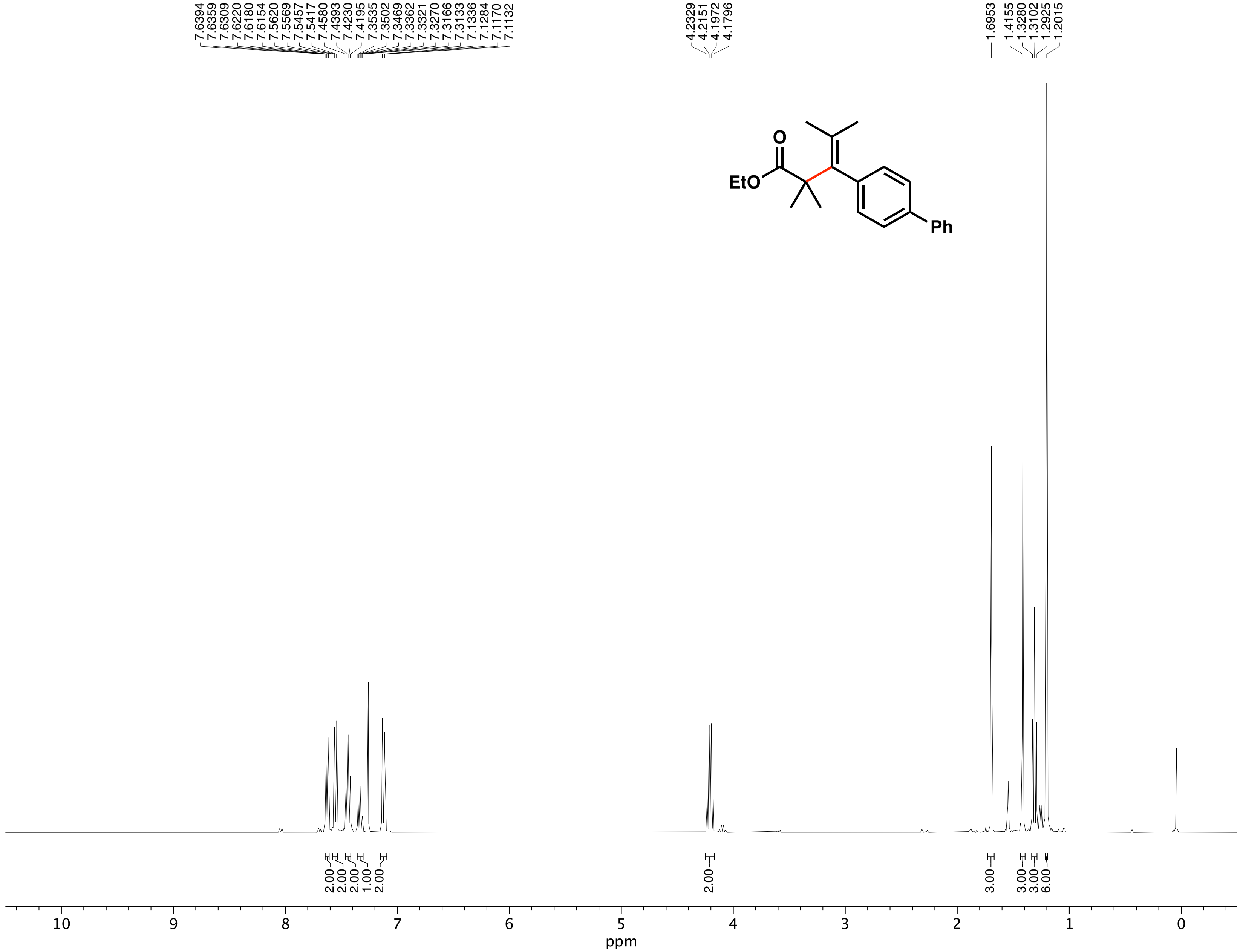

^{1}H NMR (400 MHz, $CDCl_3$) of compound **9**.

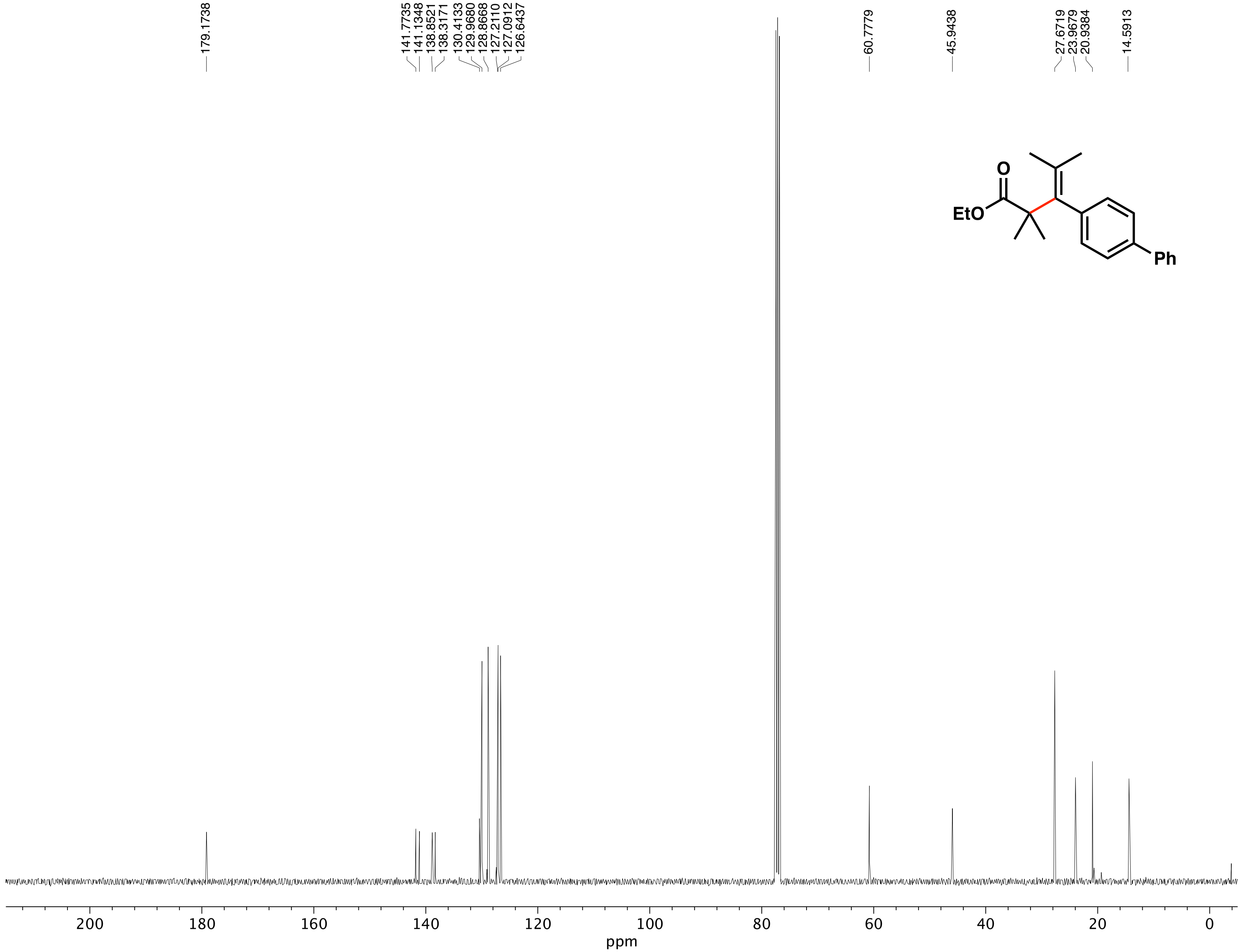

^{13}C NMR (400 MHz, $CDCl_3$) of compound **9**.

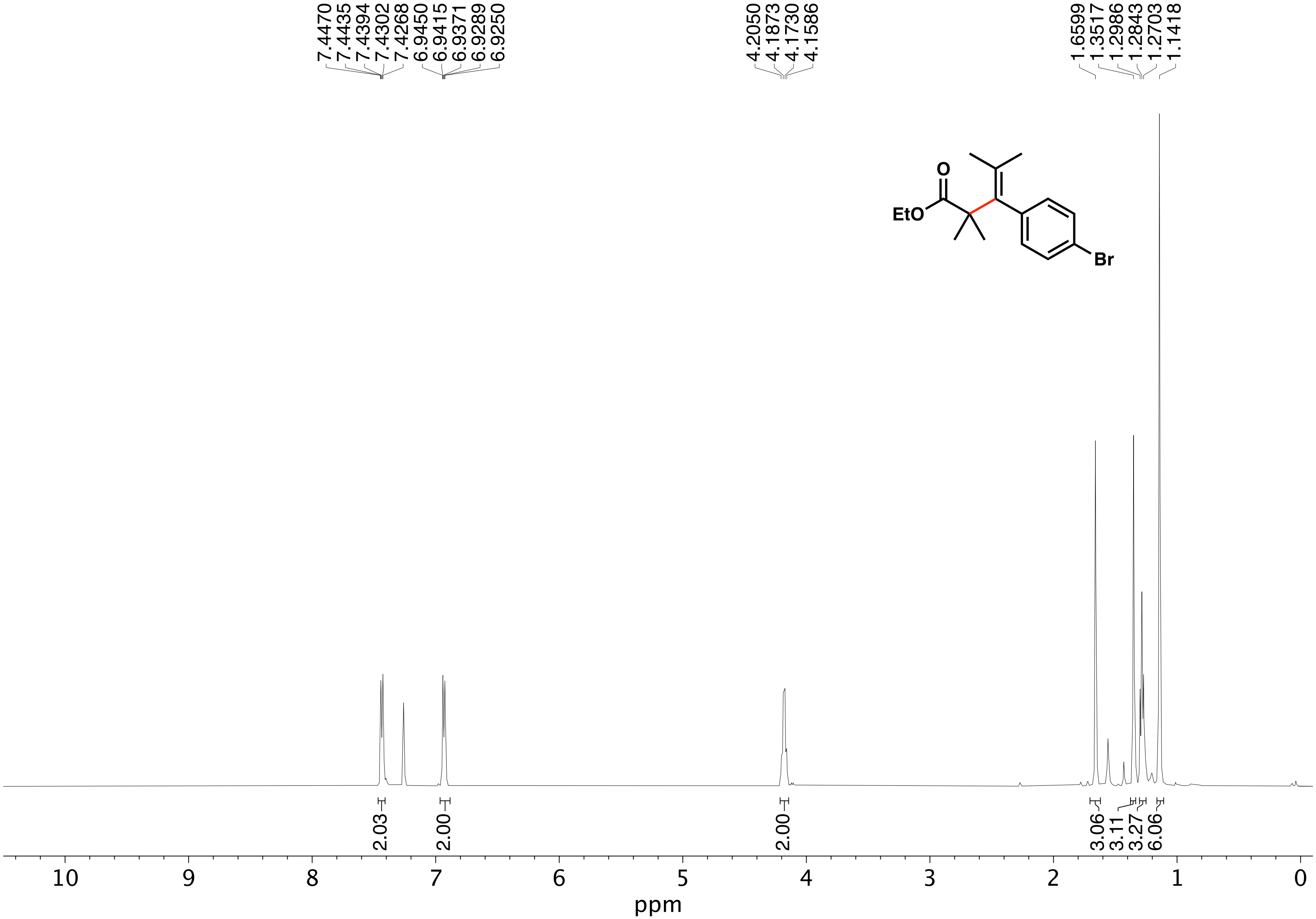

^{1}H NMR (500 MHz, $CDCl_3$) of compound **10**.

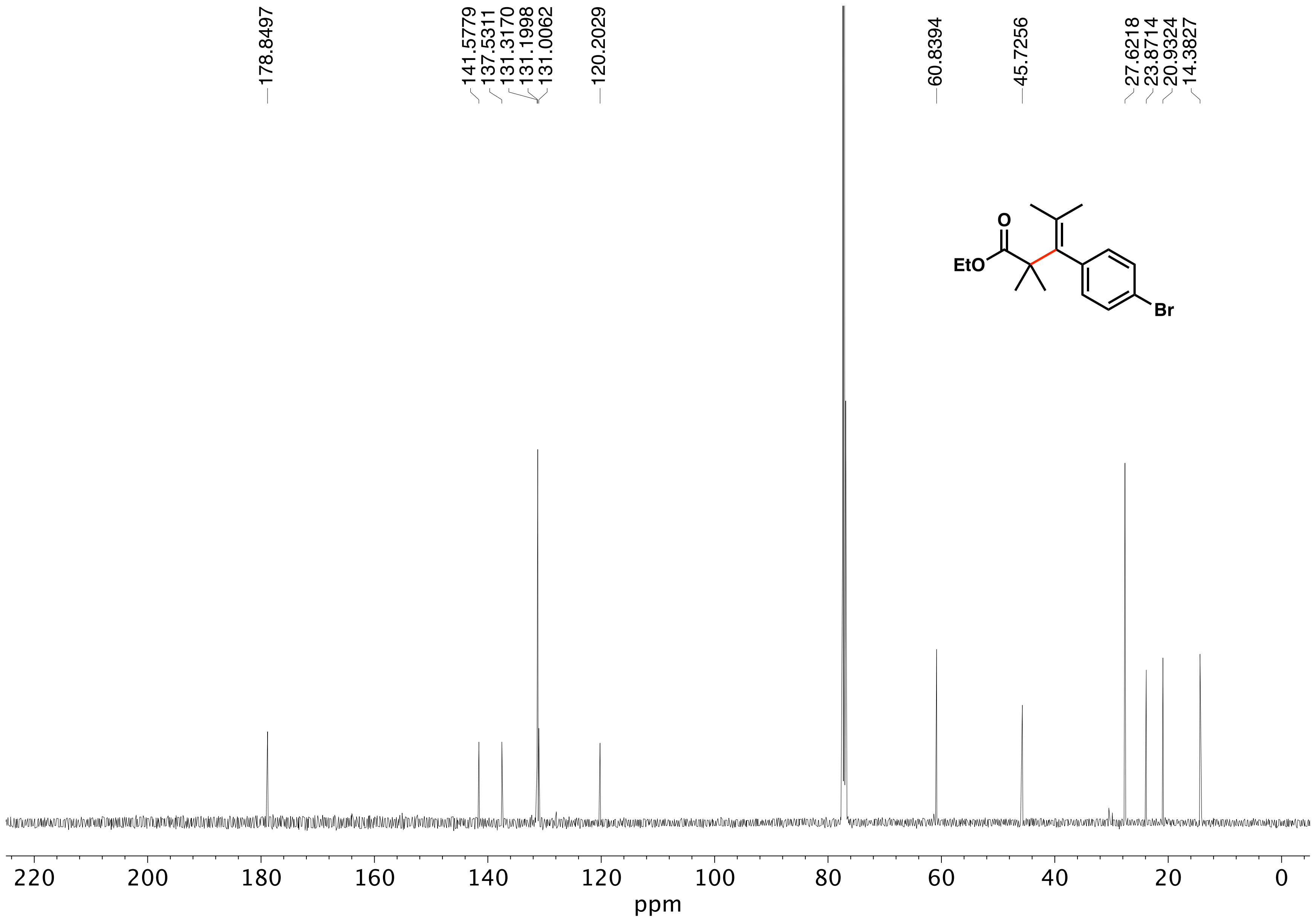

^{13}C NMR (126 MHz, $CDCl_3$) of compound **10.**

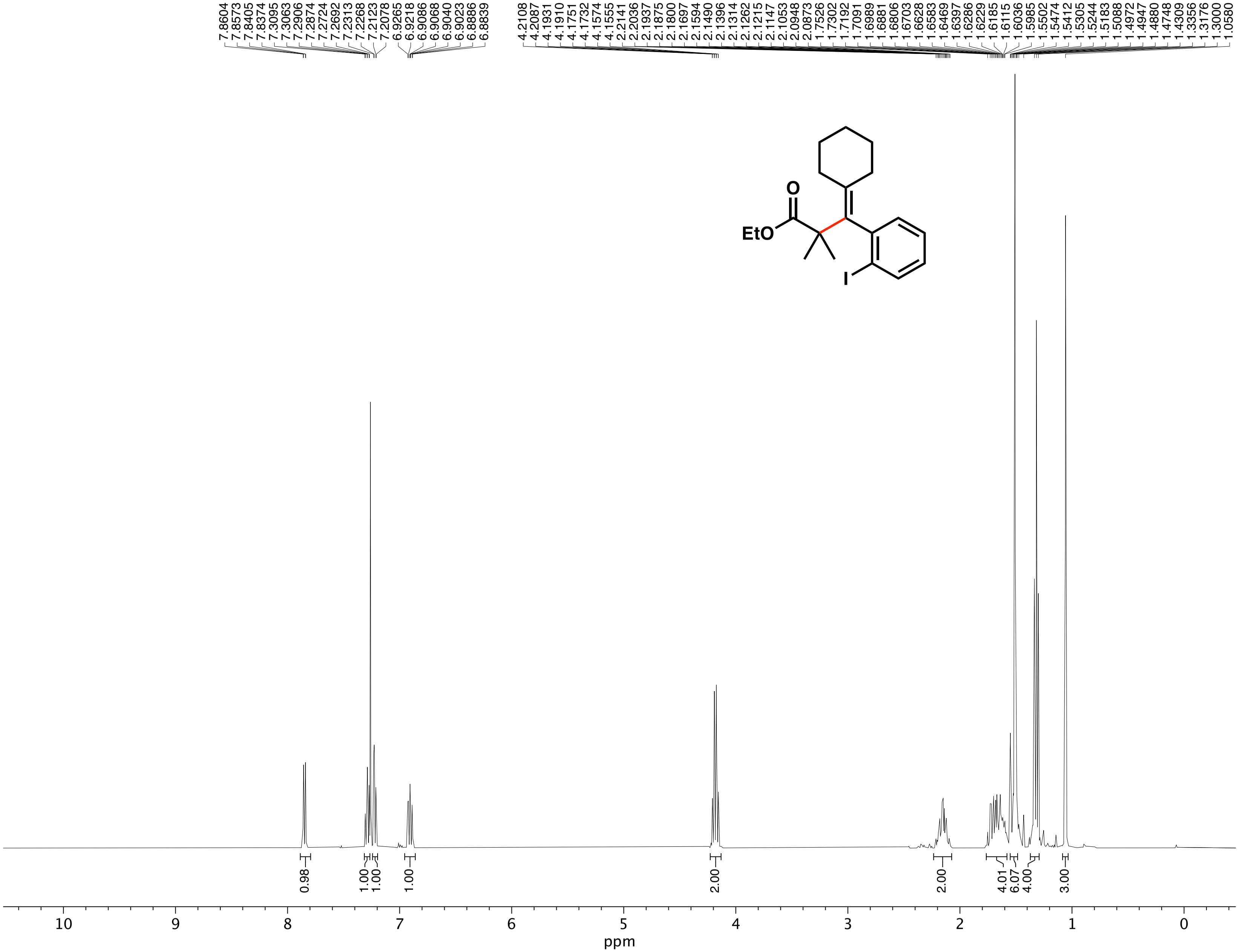

^{1}H NMR (400 MHz, $CDCl_3$) of compound **11**.

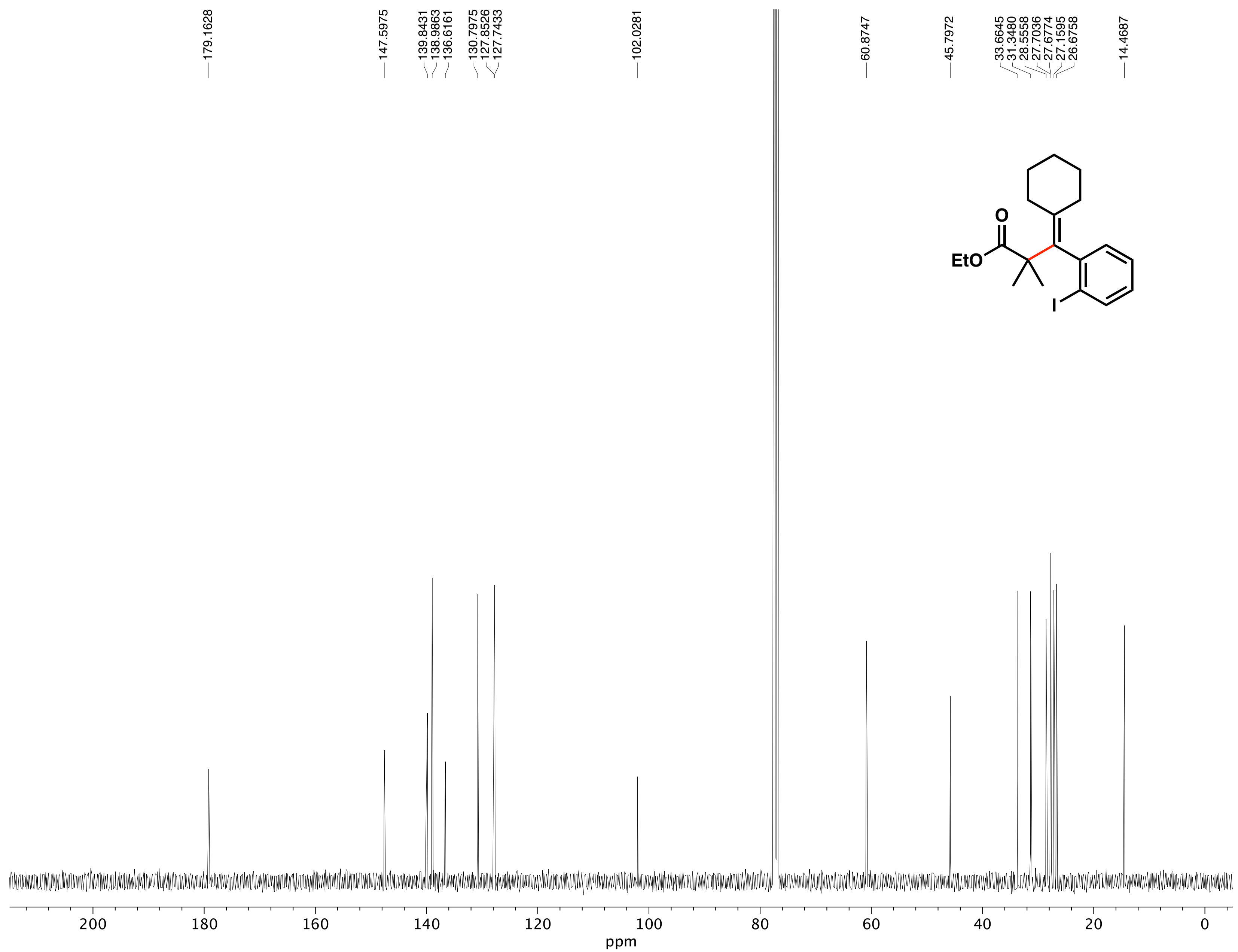

^{13}C NMR (101 MHz, $CDCl_3$) of compound **11**.

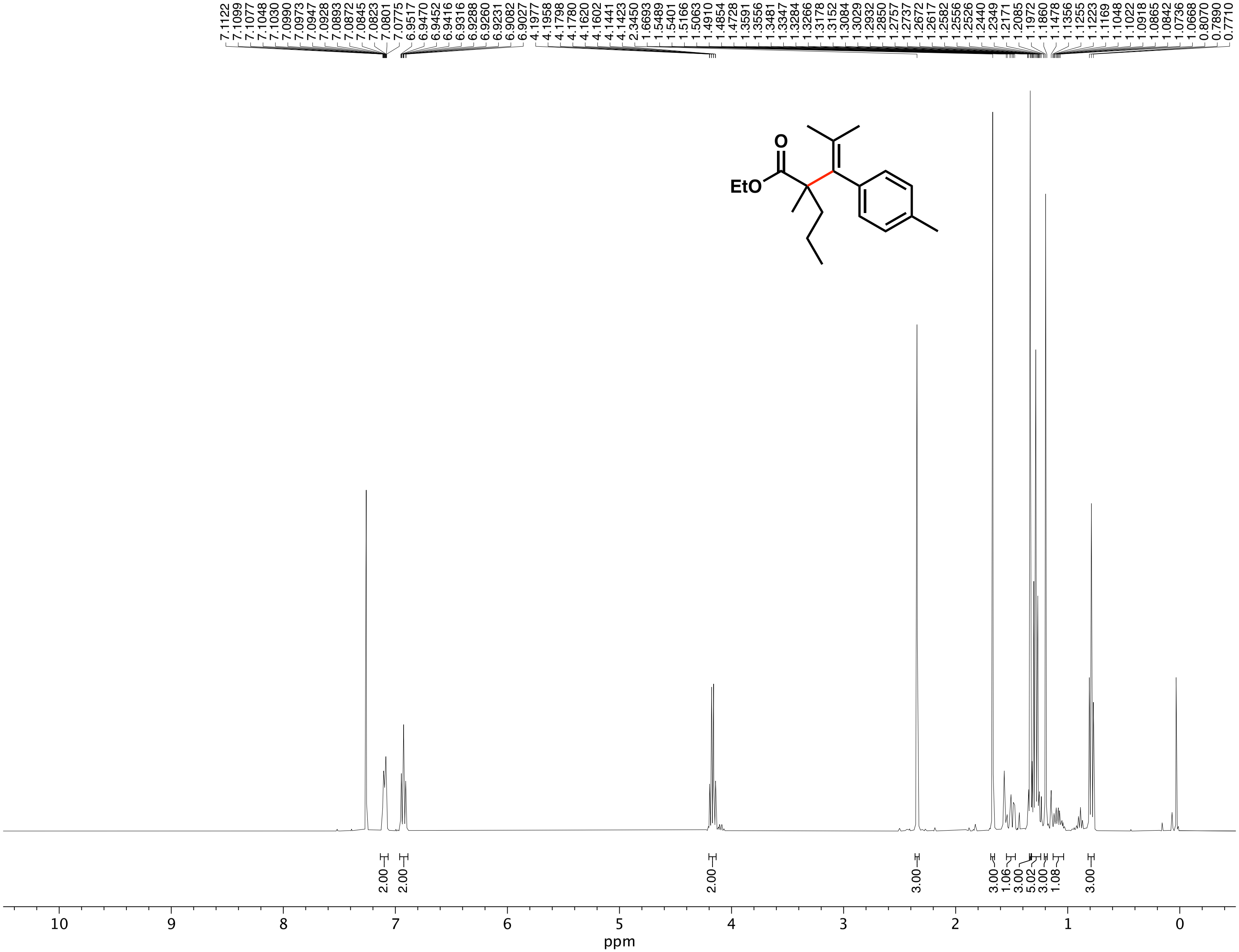

^{1}H NMR (400 MHz, $CDCl_3$) of compound **12**.

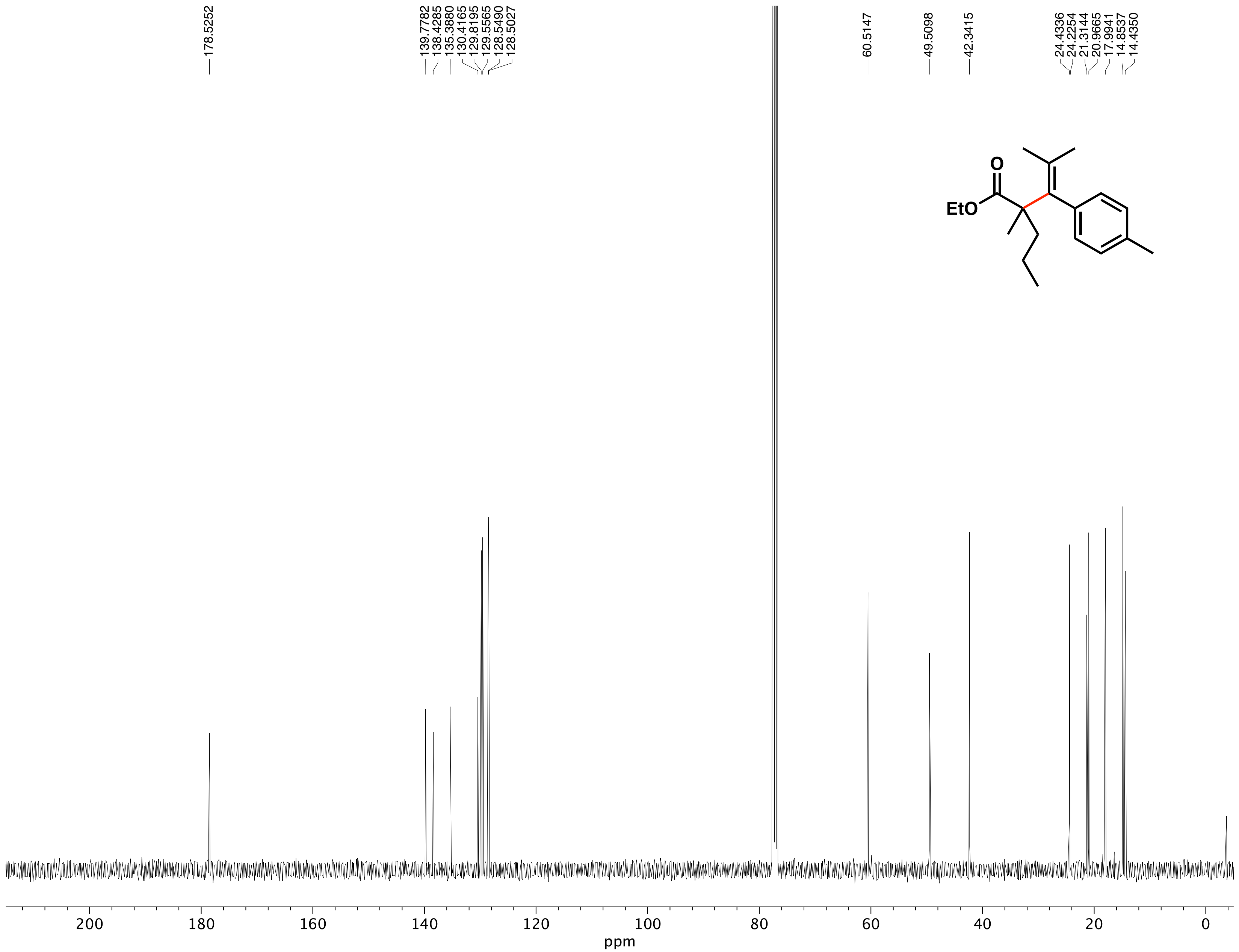

^{13}C NMR (101 MHz, $CDCl_3$) of compound **12.**

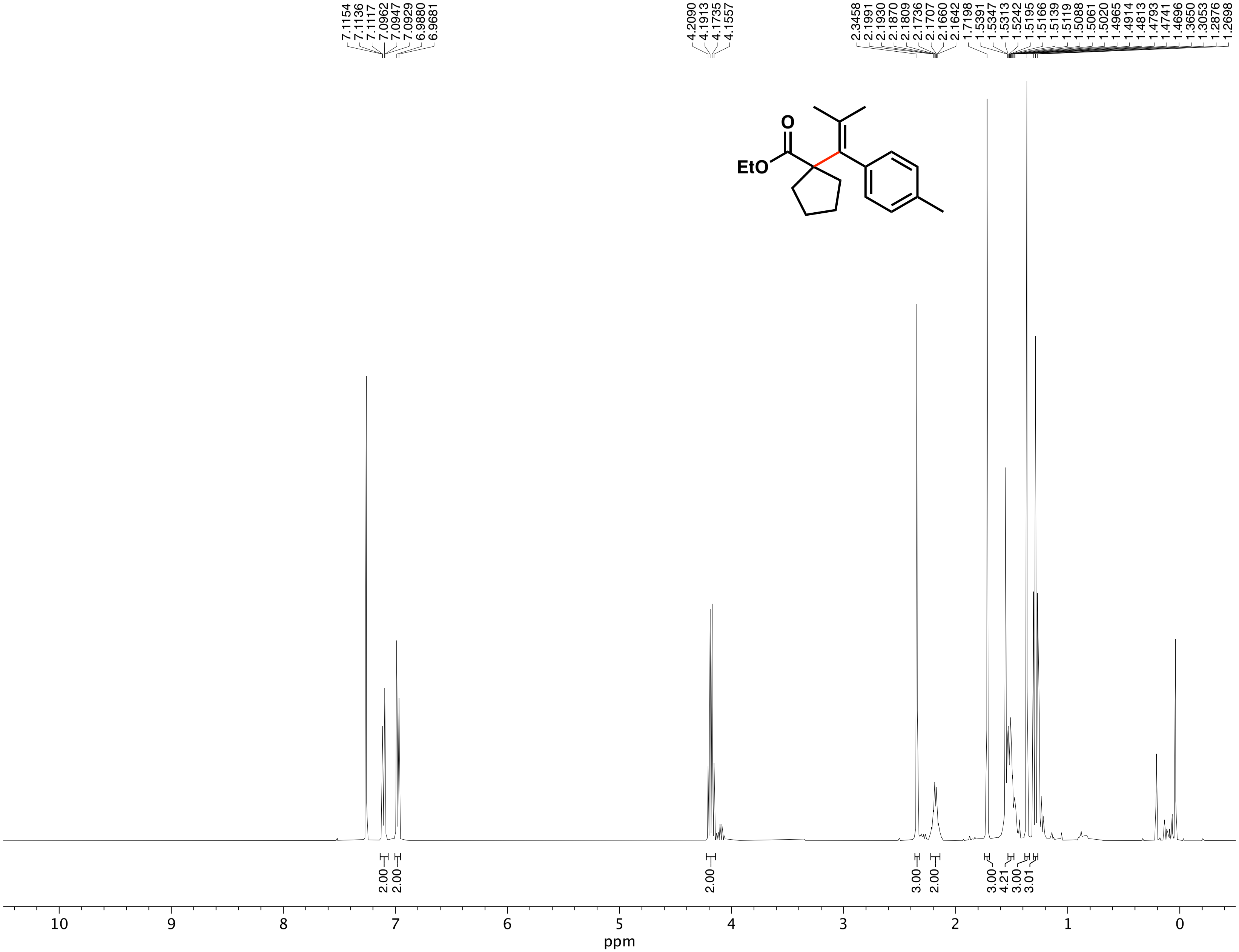

^{1}H NMR (400 MHz, $CDCl_3$) of compound **13**.

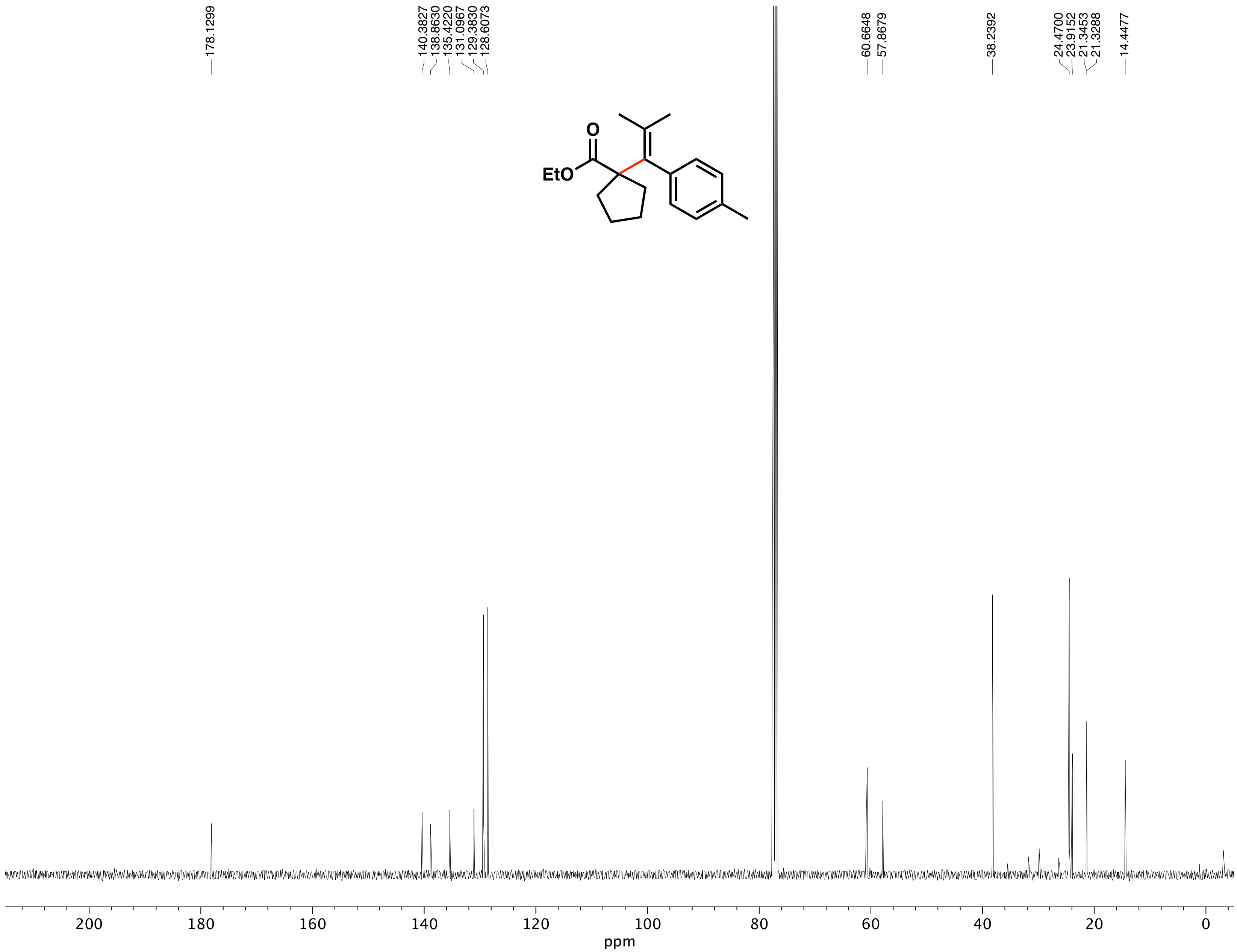

^{13}C NMR (101 MHz, $CDCl_3$) of compound **13**.

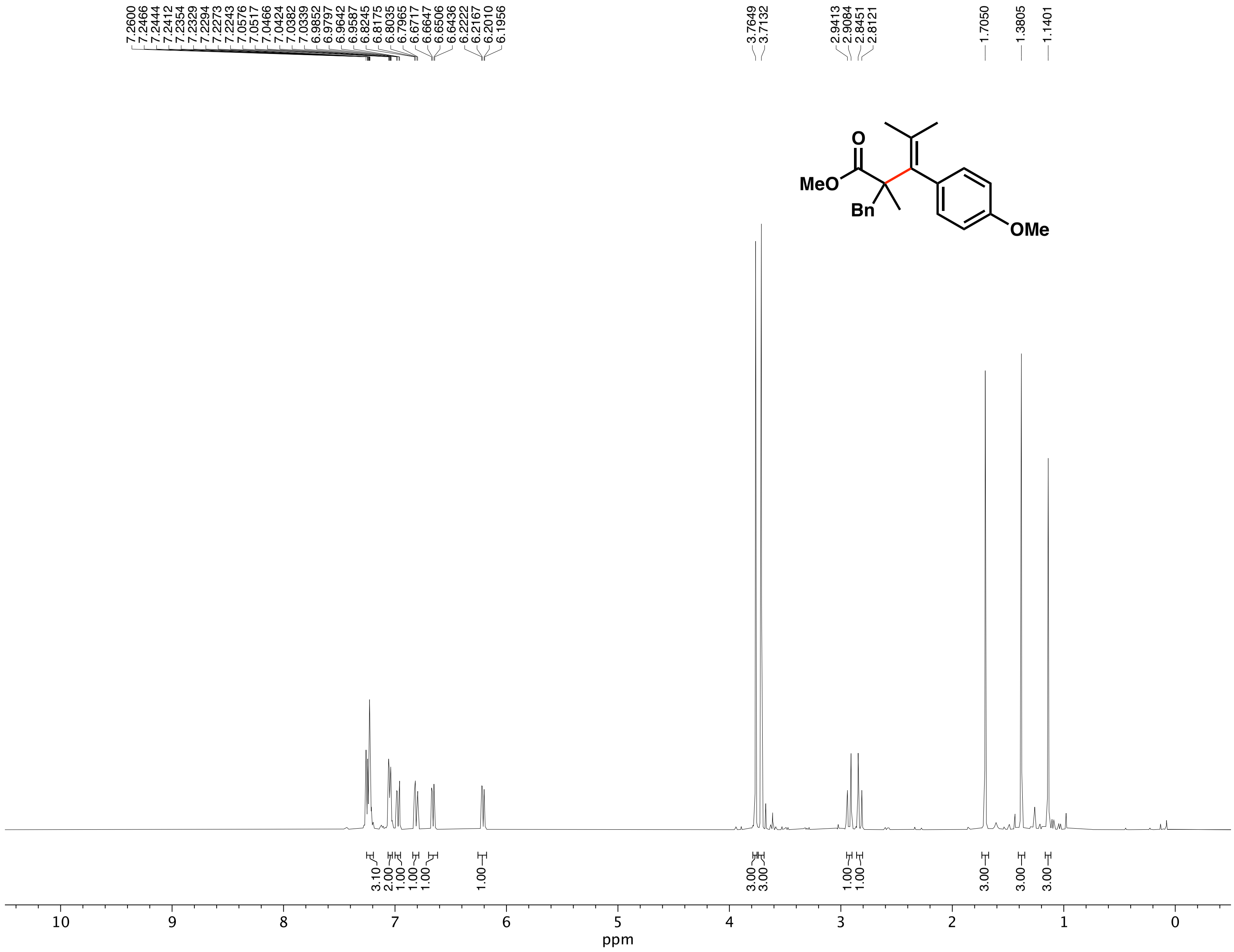

^{1}H NMR (400 MHz, $CDCl_3$) of compound **14**.

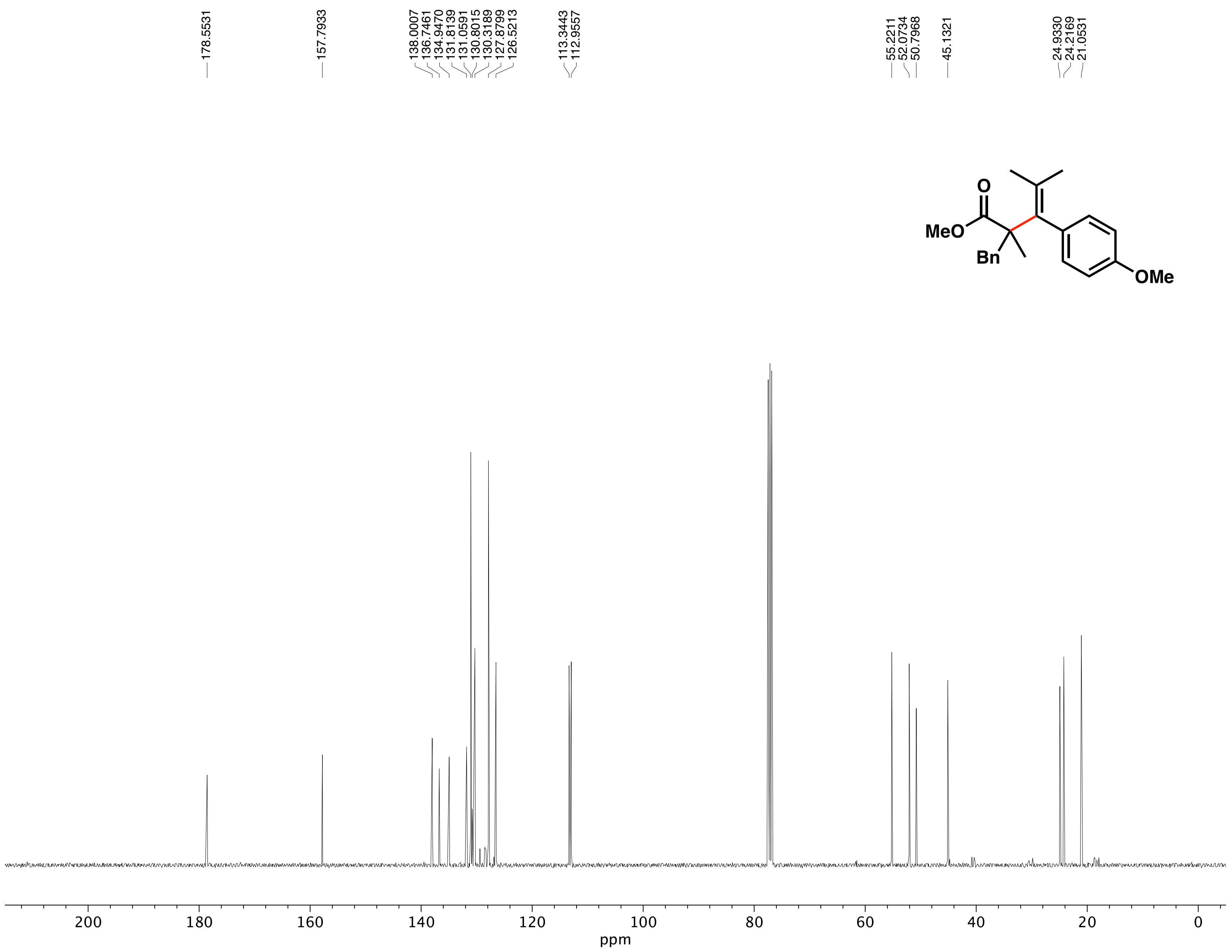

^{13}C NMR (400 MHz, $CDCl_3$) of compound **14**.

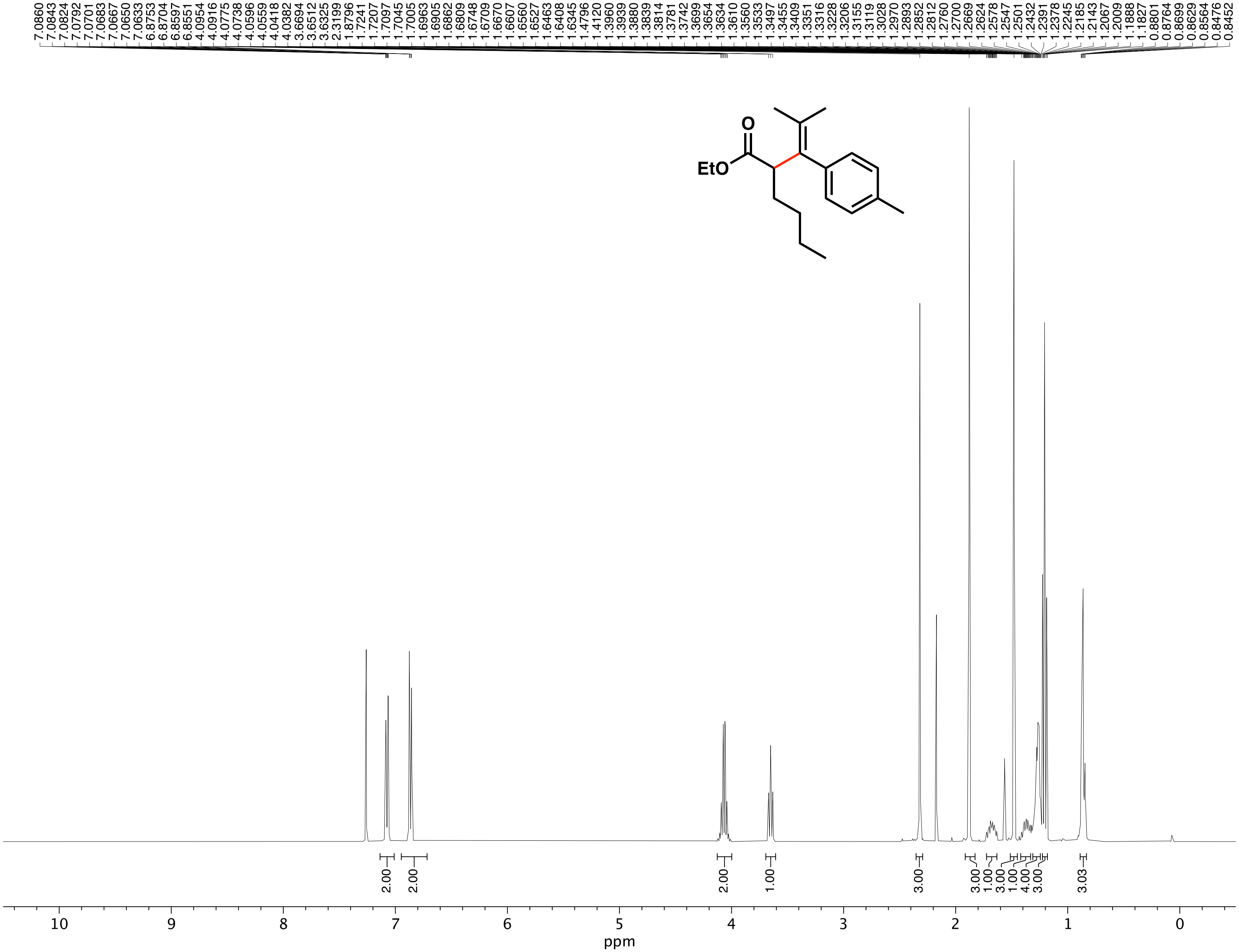

^{1}H NMR (400 MHz, $CDCl_3$) of compound **15.**

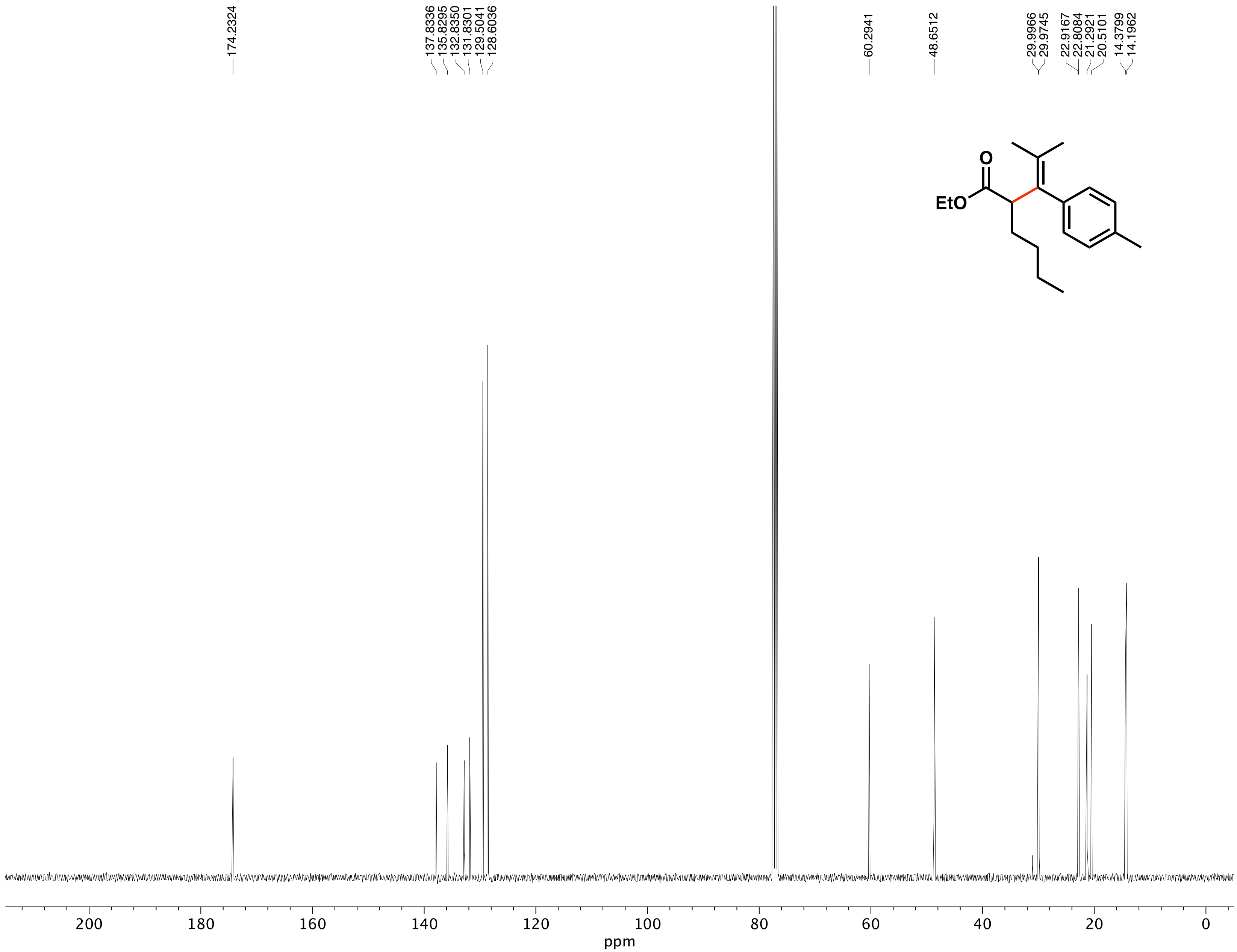

^{13}C NMR (101 MHz, $CDCl_3$) of compound **15**.

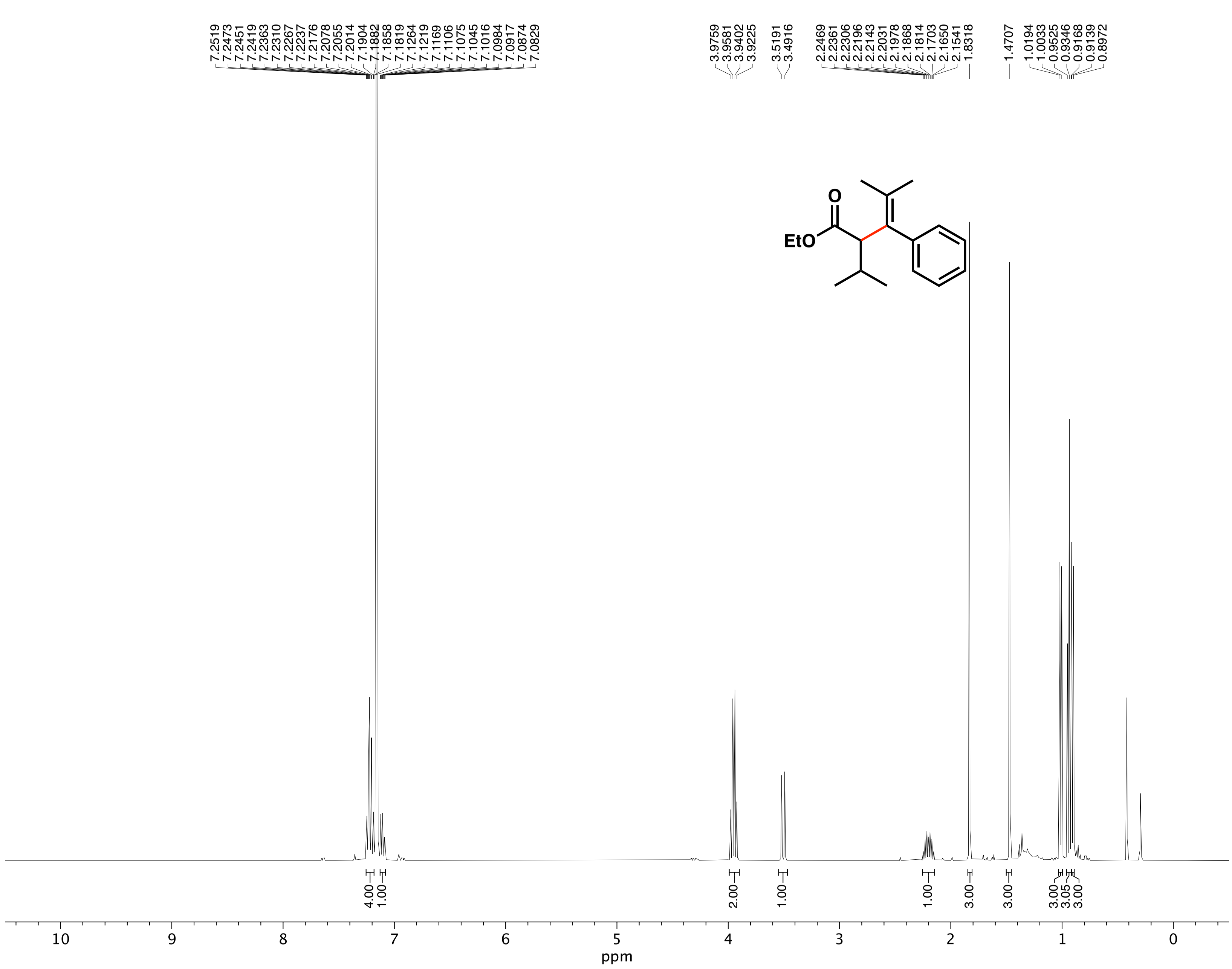

^{1}H NMR (400 MHz, C_6D_6) of compound **16.**

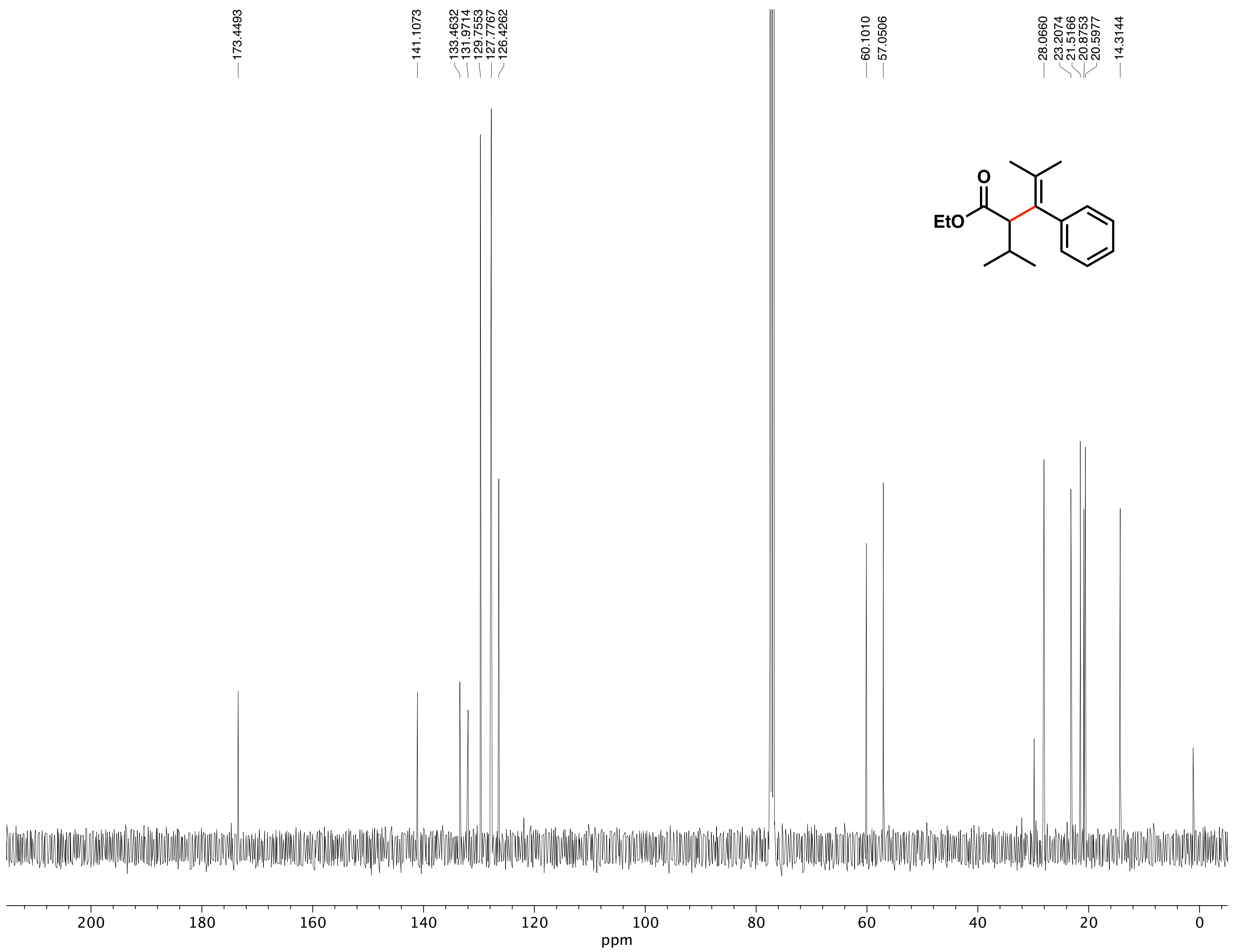

^{13}C NMR (101 MHz, $CDCl_3$) of compound **16**.

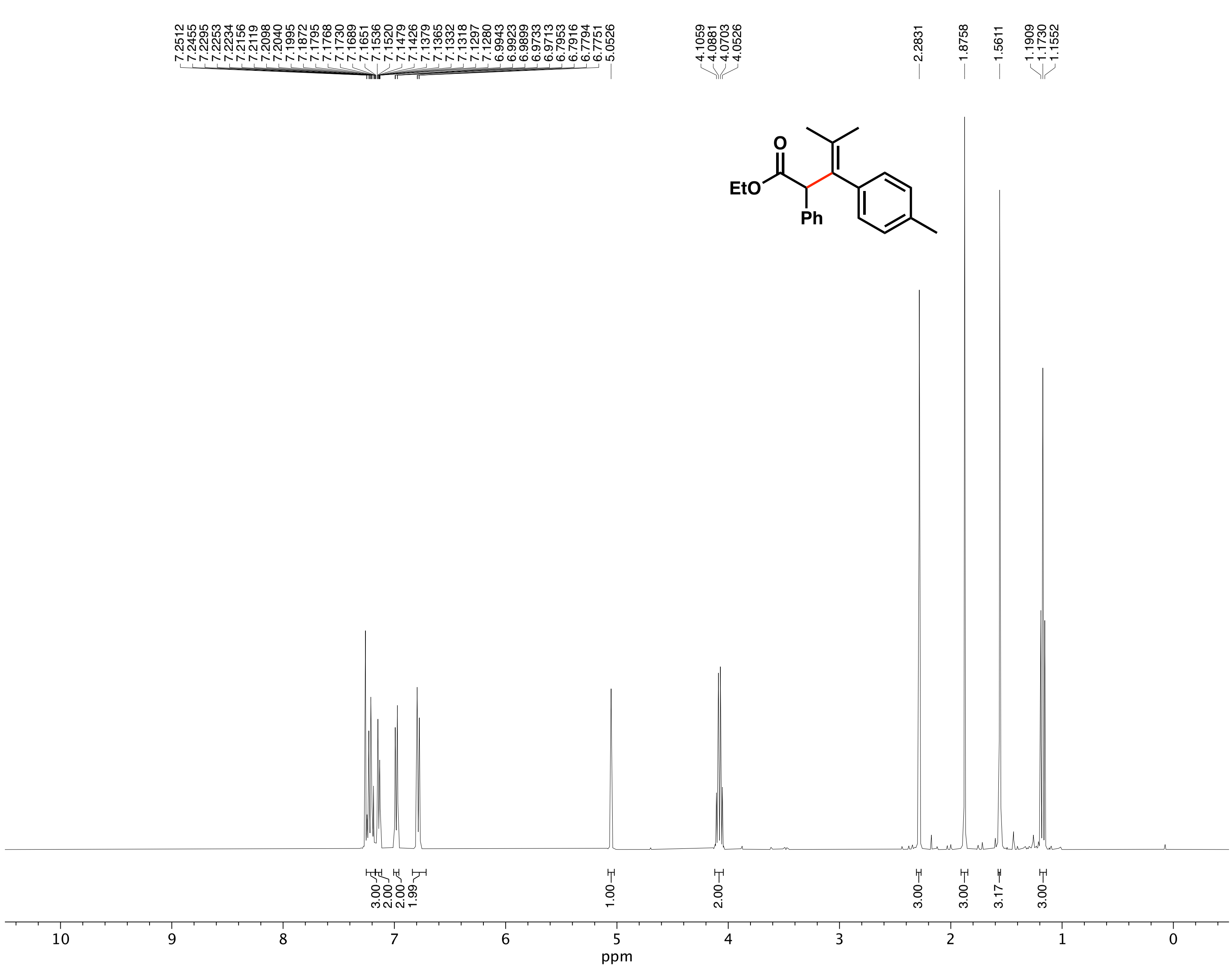

^{1}H NMR (400 MHz, $CDCl_3$) of compound **17**.

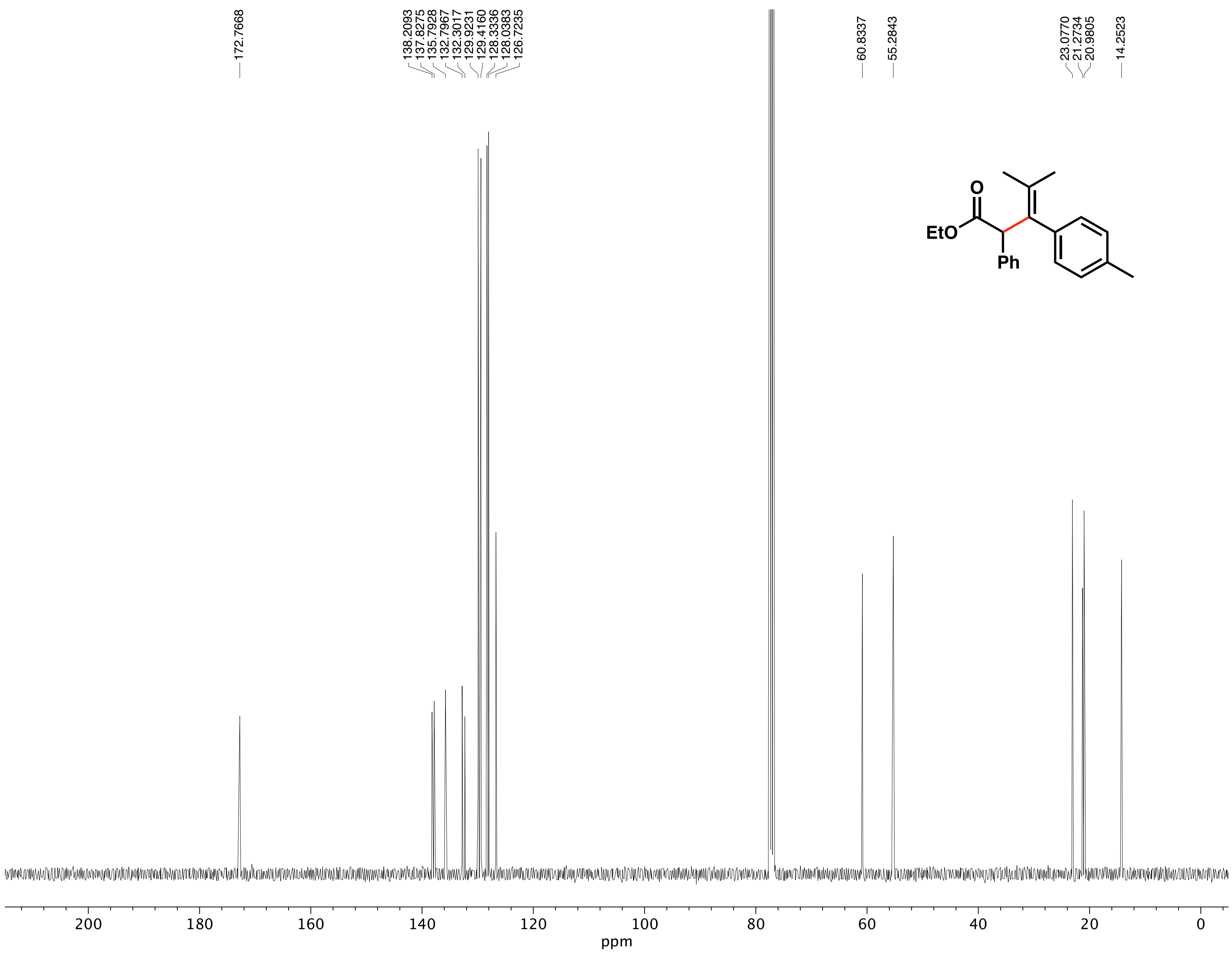

^{13}C NMR (400 MHz, $CDCl_3$) of compound **17**.

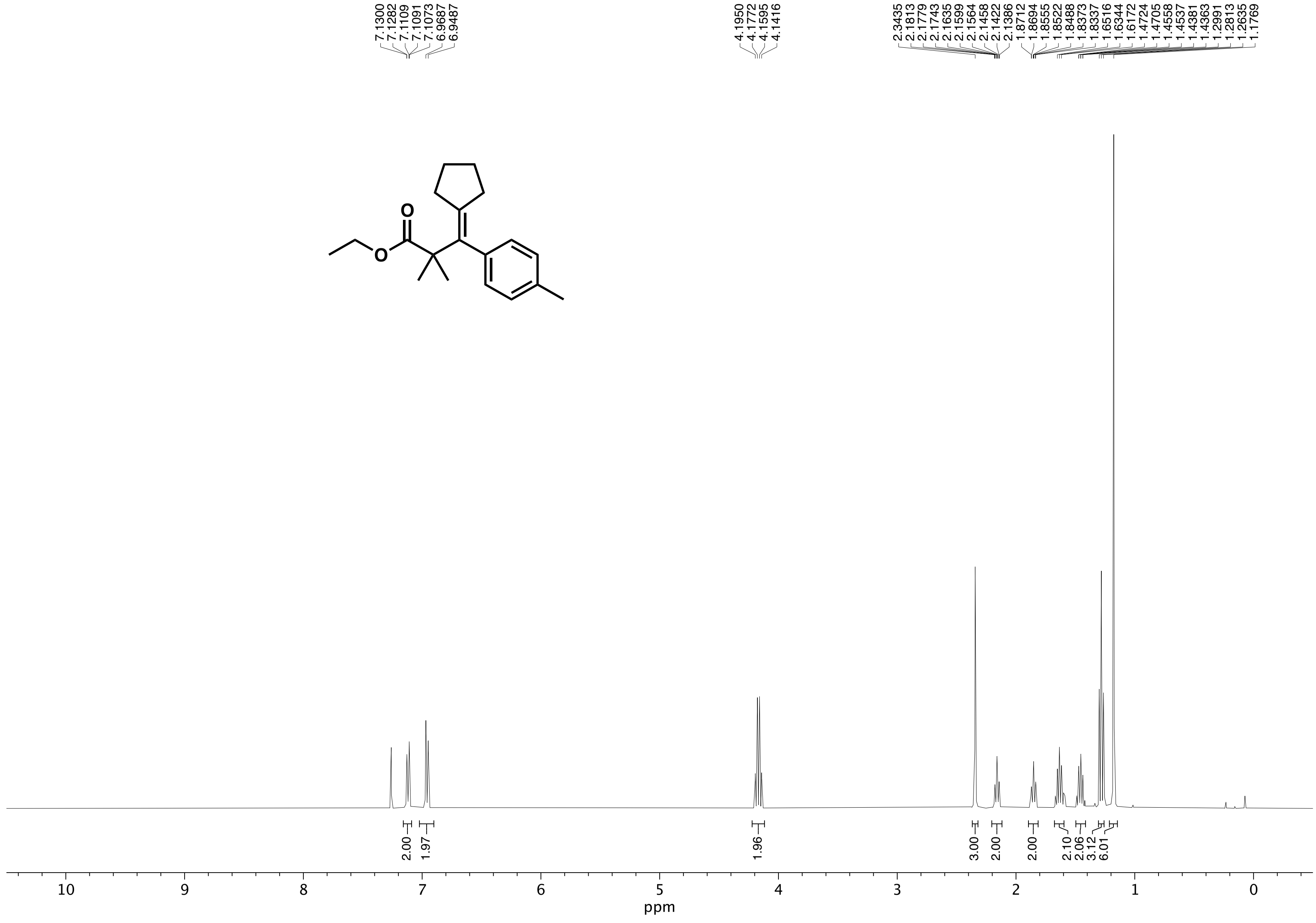

^{1}H NMR (400 MHz, $CDCl_3$) of compound **20.**

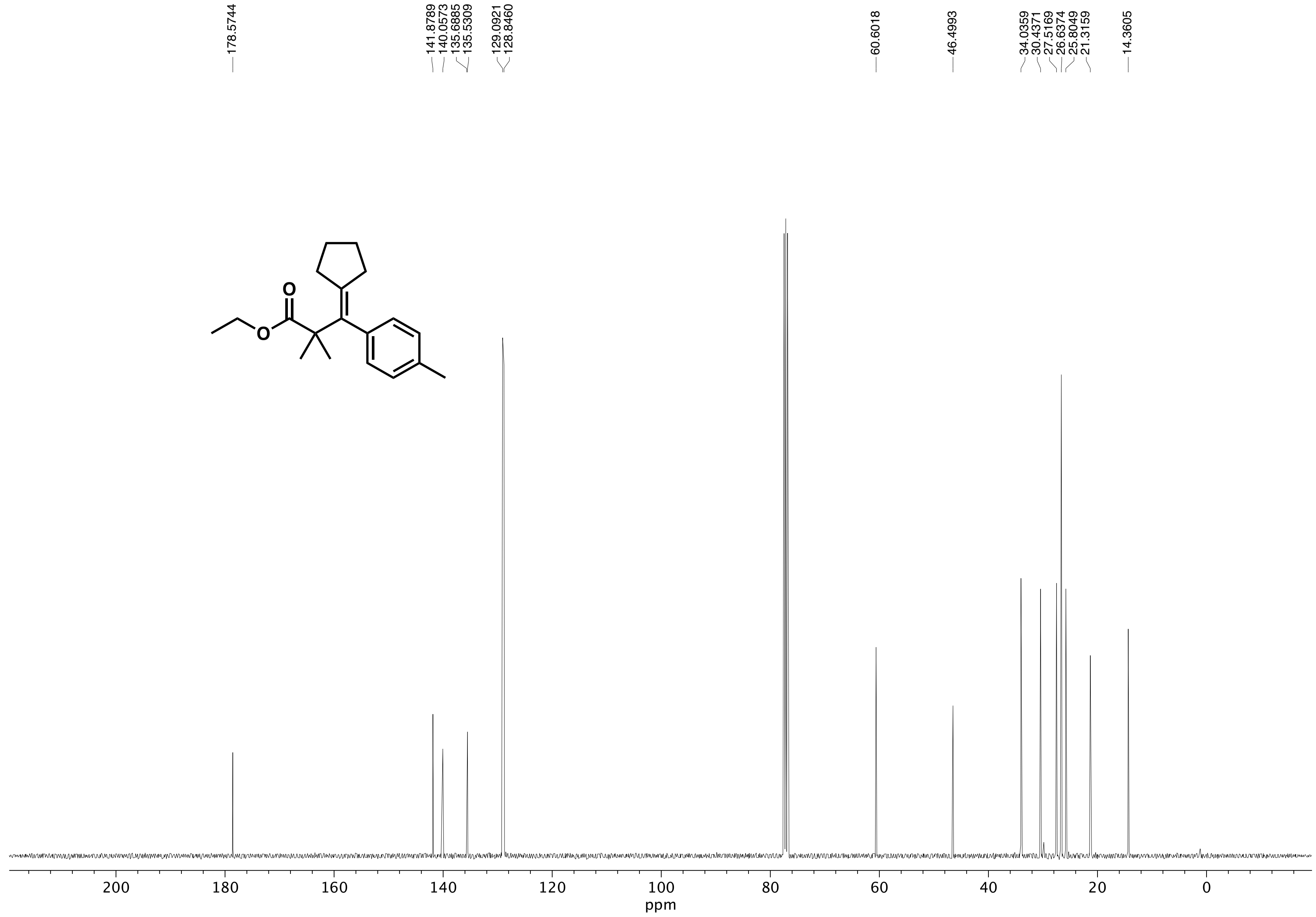

^{13}C NMR (101 MHz, $CDCl_3$) of compound **20.**

.

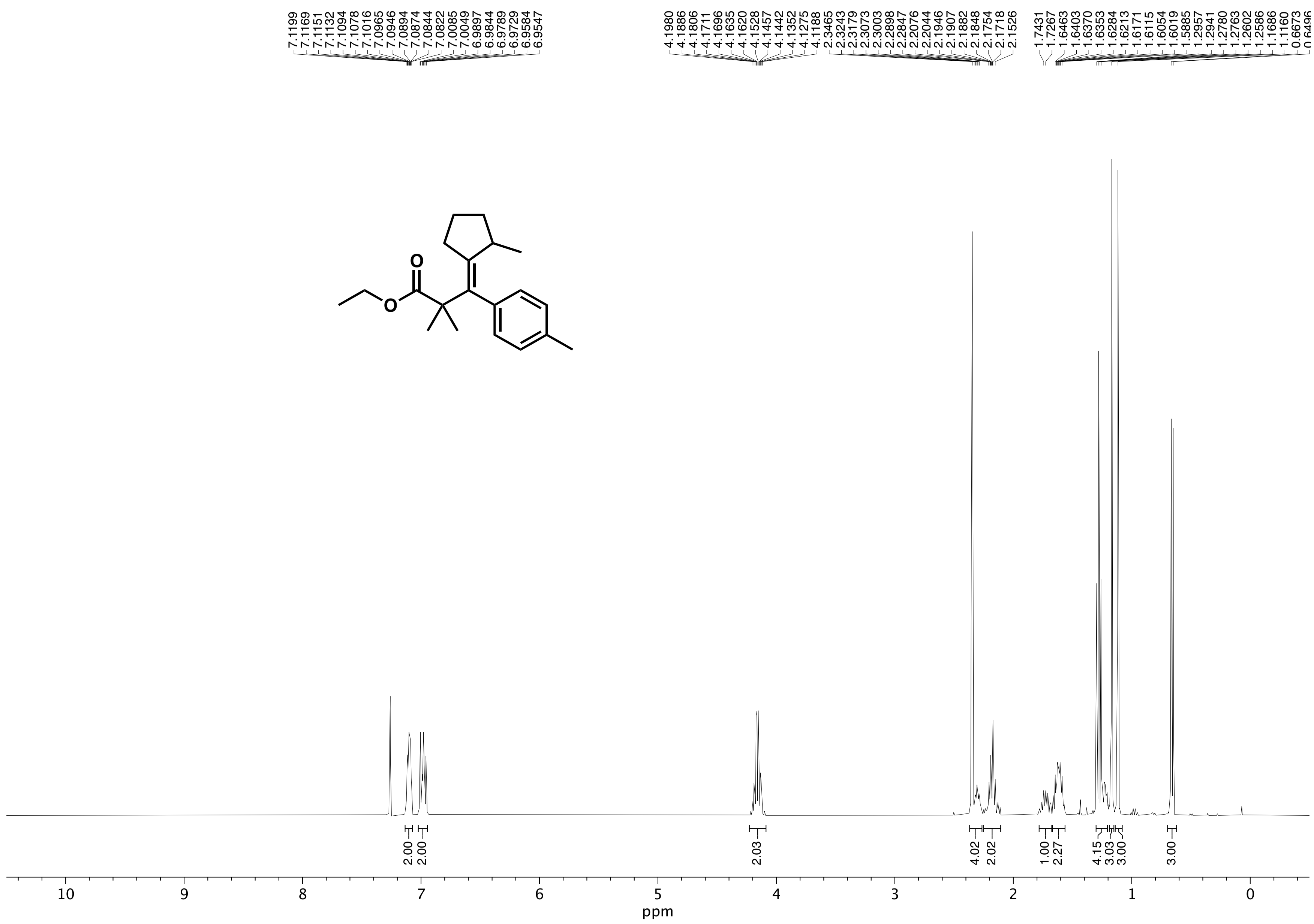

^{1}H NMR (400 MHz, $CDCl_3$) of compound **21**.

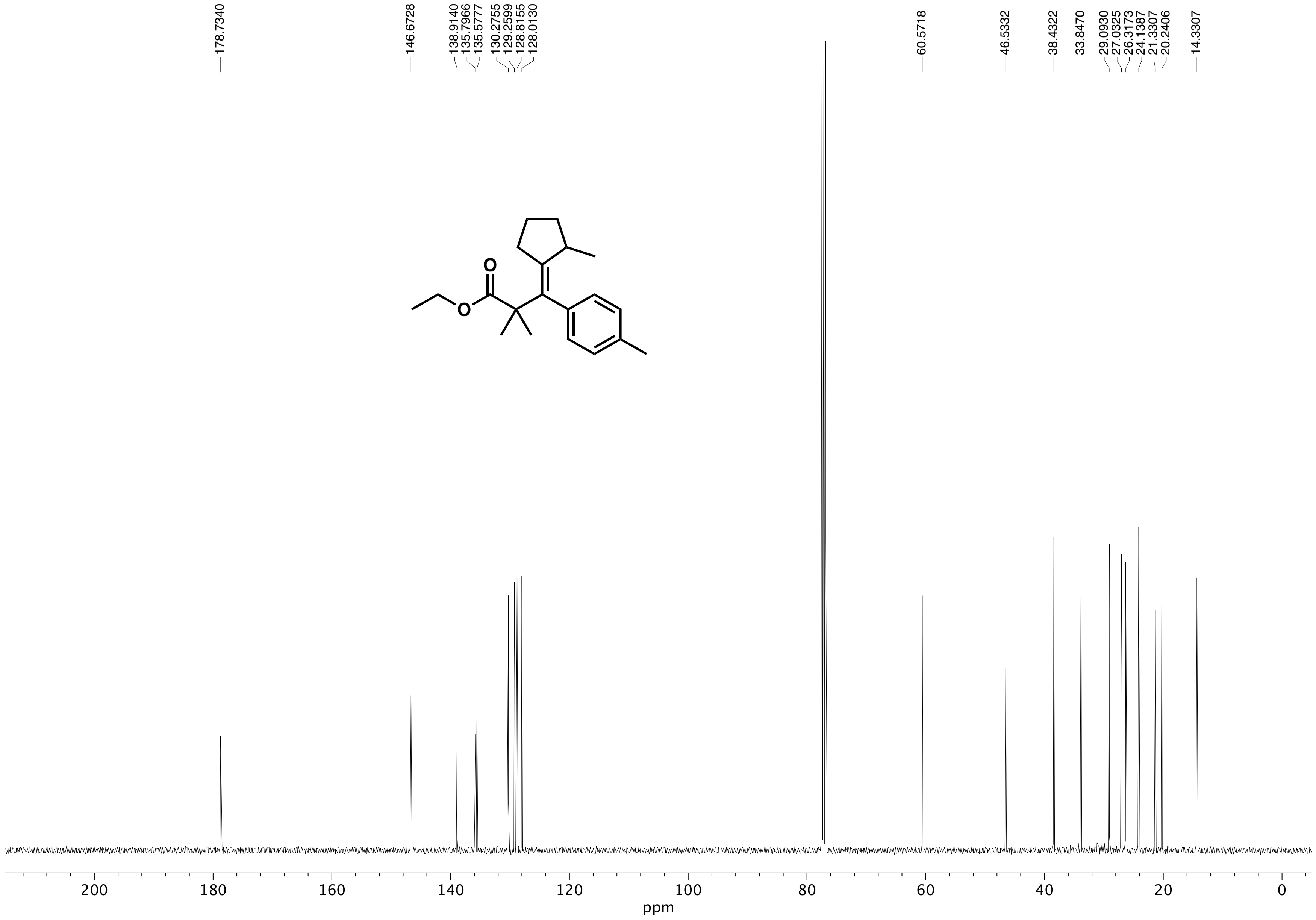

^{13}C NMR (101 MHz, $CDCl_3$) of compound **21**.

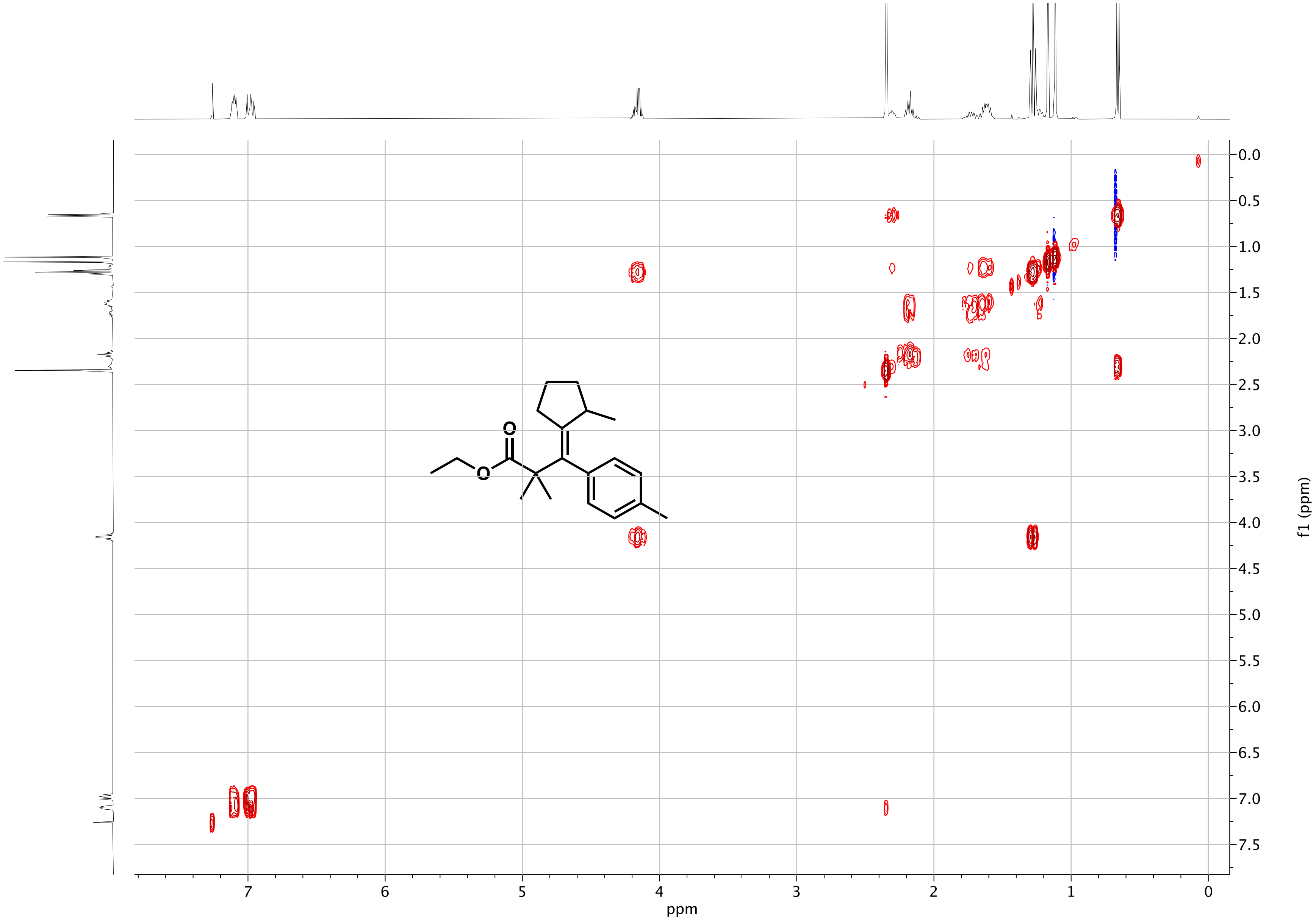

COSY NMR (400 MHz, $CDCl_3$) of **21**.

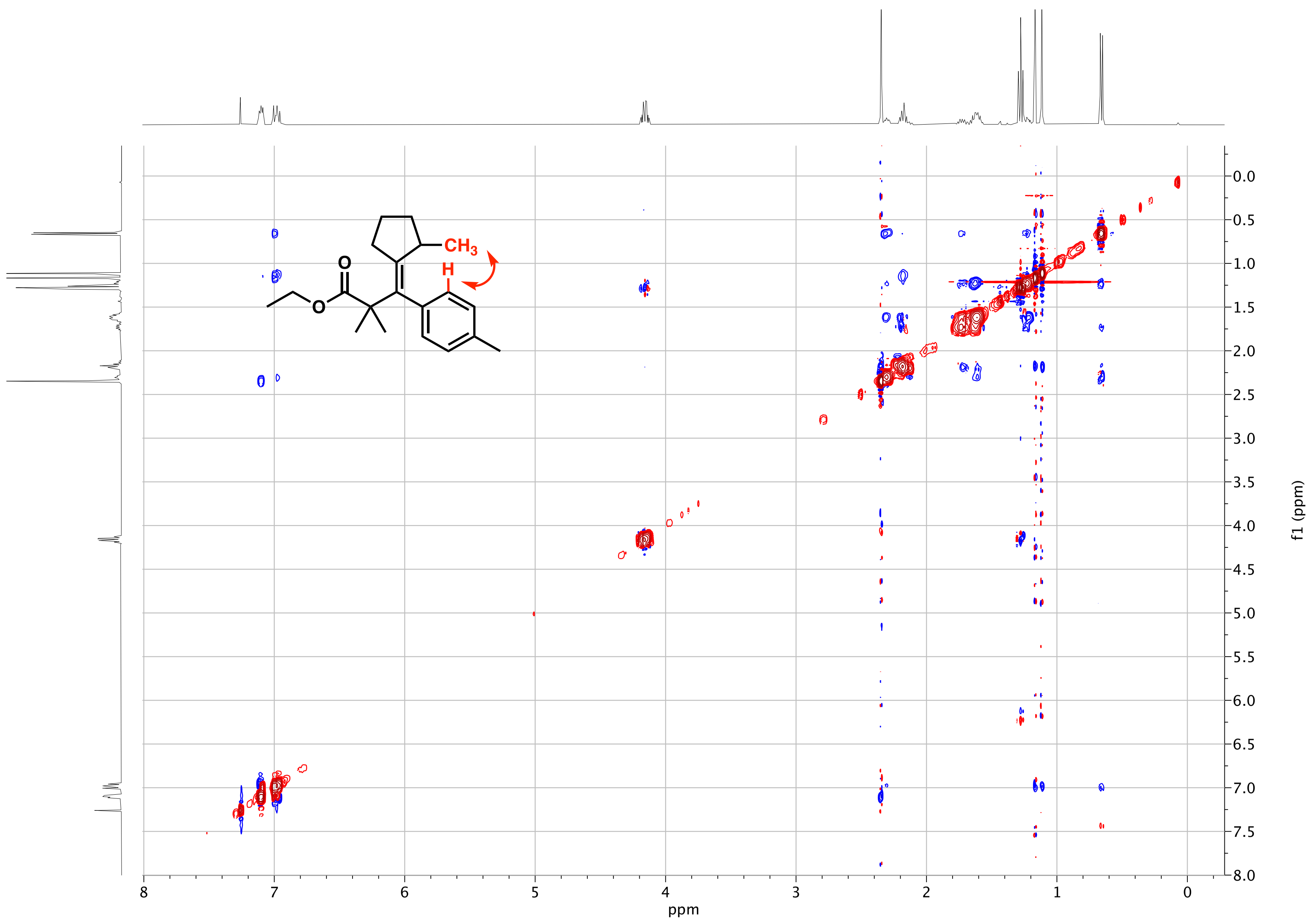

NOESY NMR (400 MHz, $CDCl_3$) of **21.**

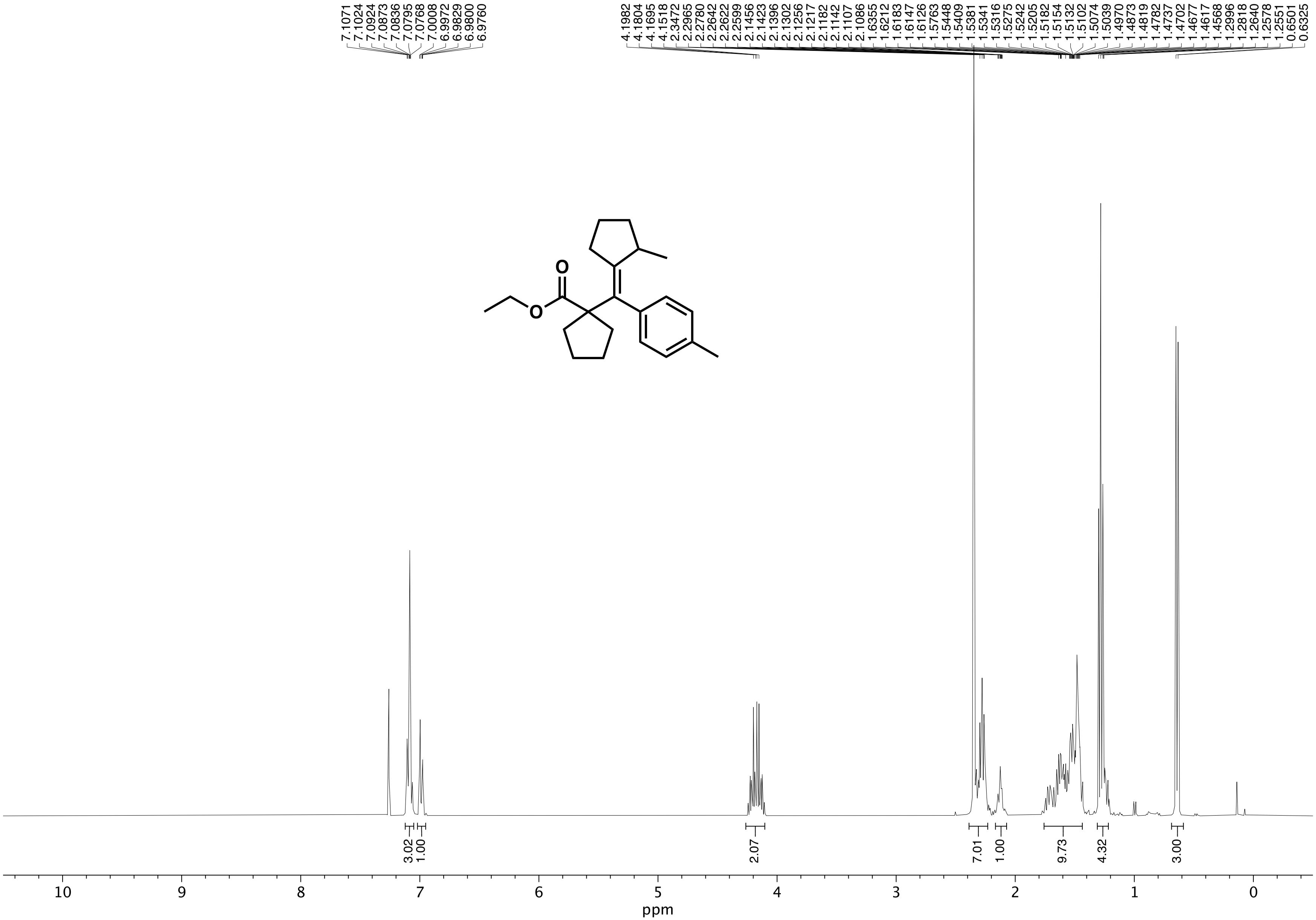

^{1}H NMR (400 MHz, $CDCl_3$) of compound **22.**

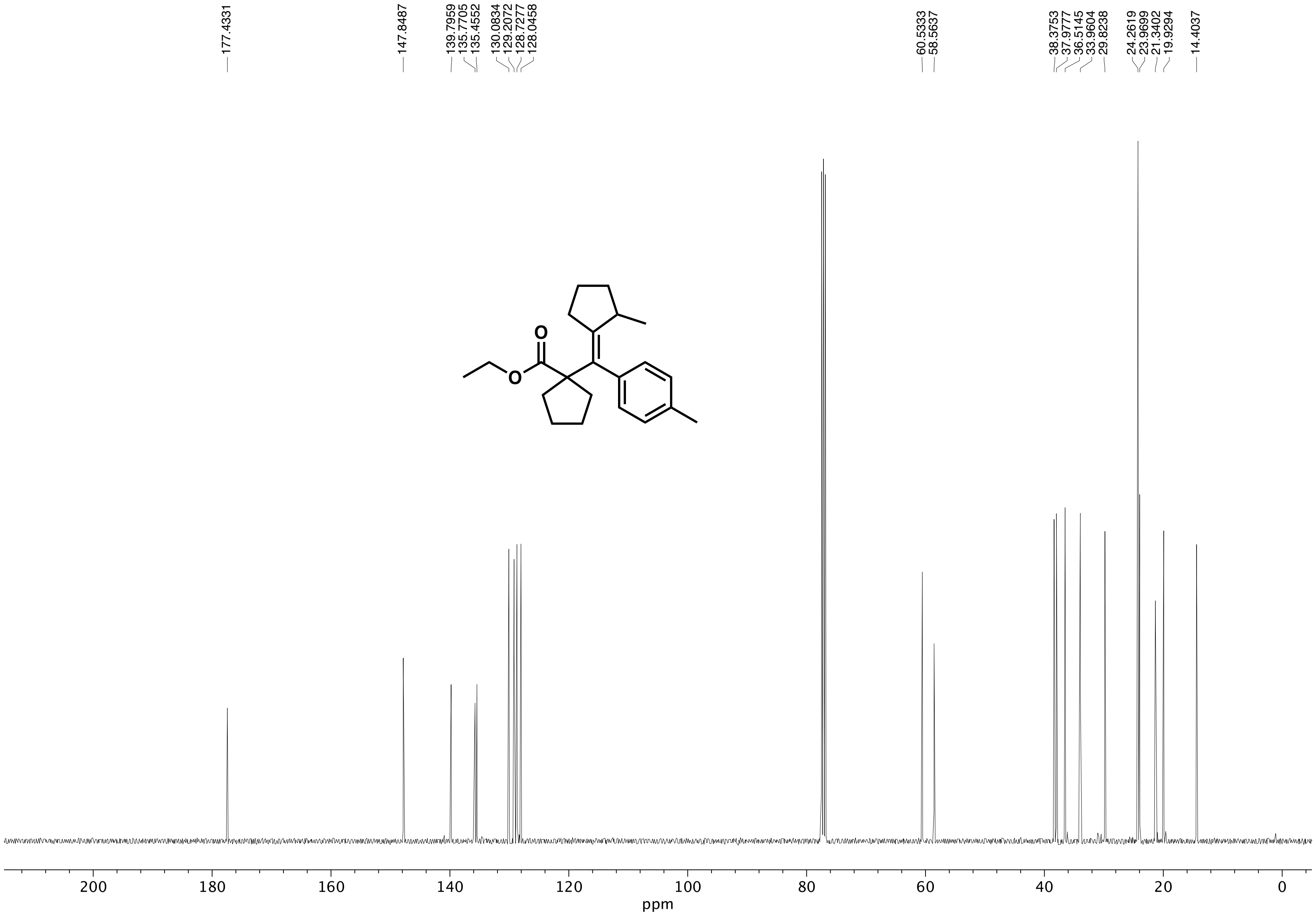

^{13}C NMR (101 MHz, $CDCl_3$) of compound **22.**

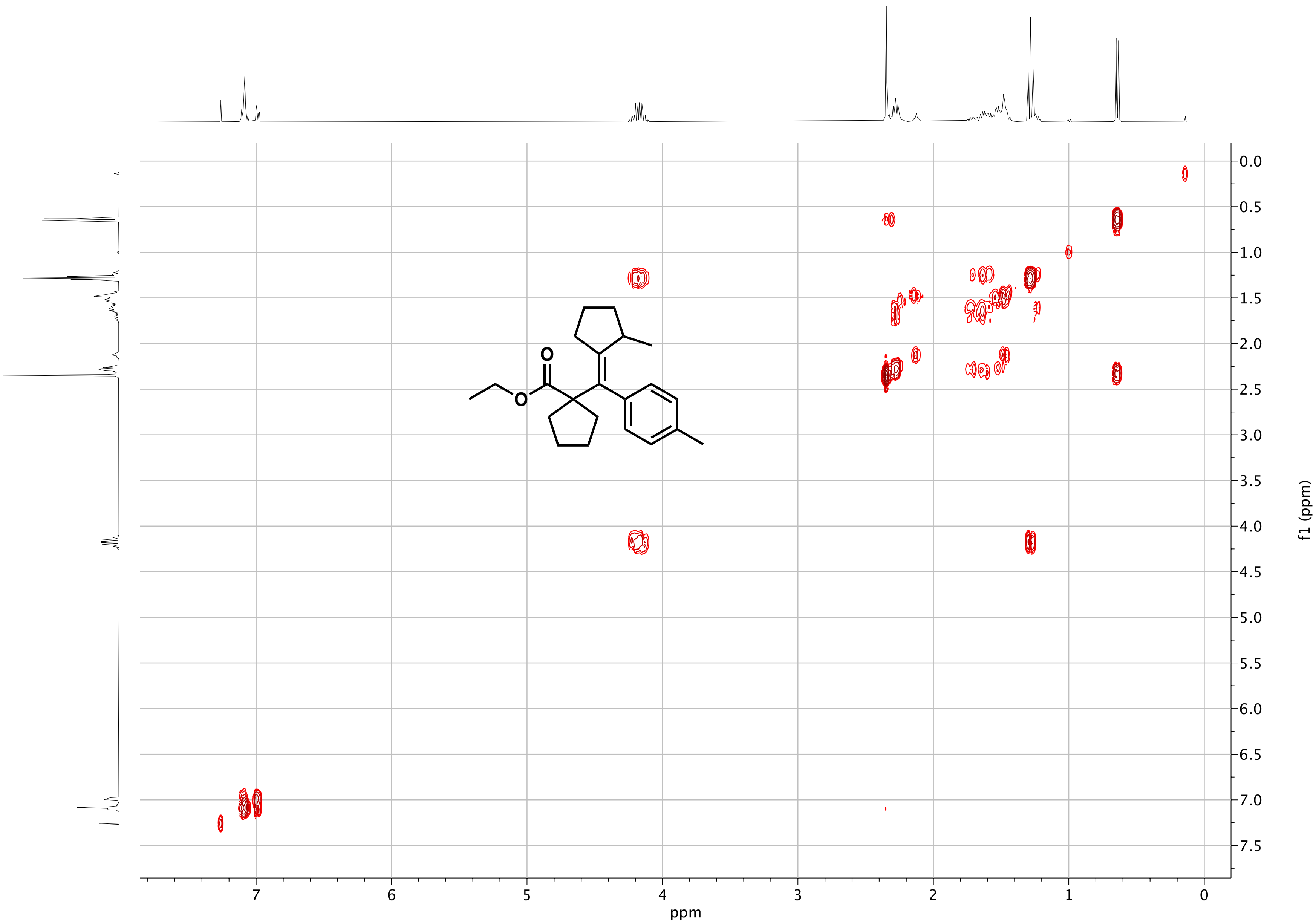

COSY NMR (400 MHz, $CDCl_3$) of compound **22.**

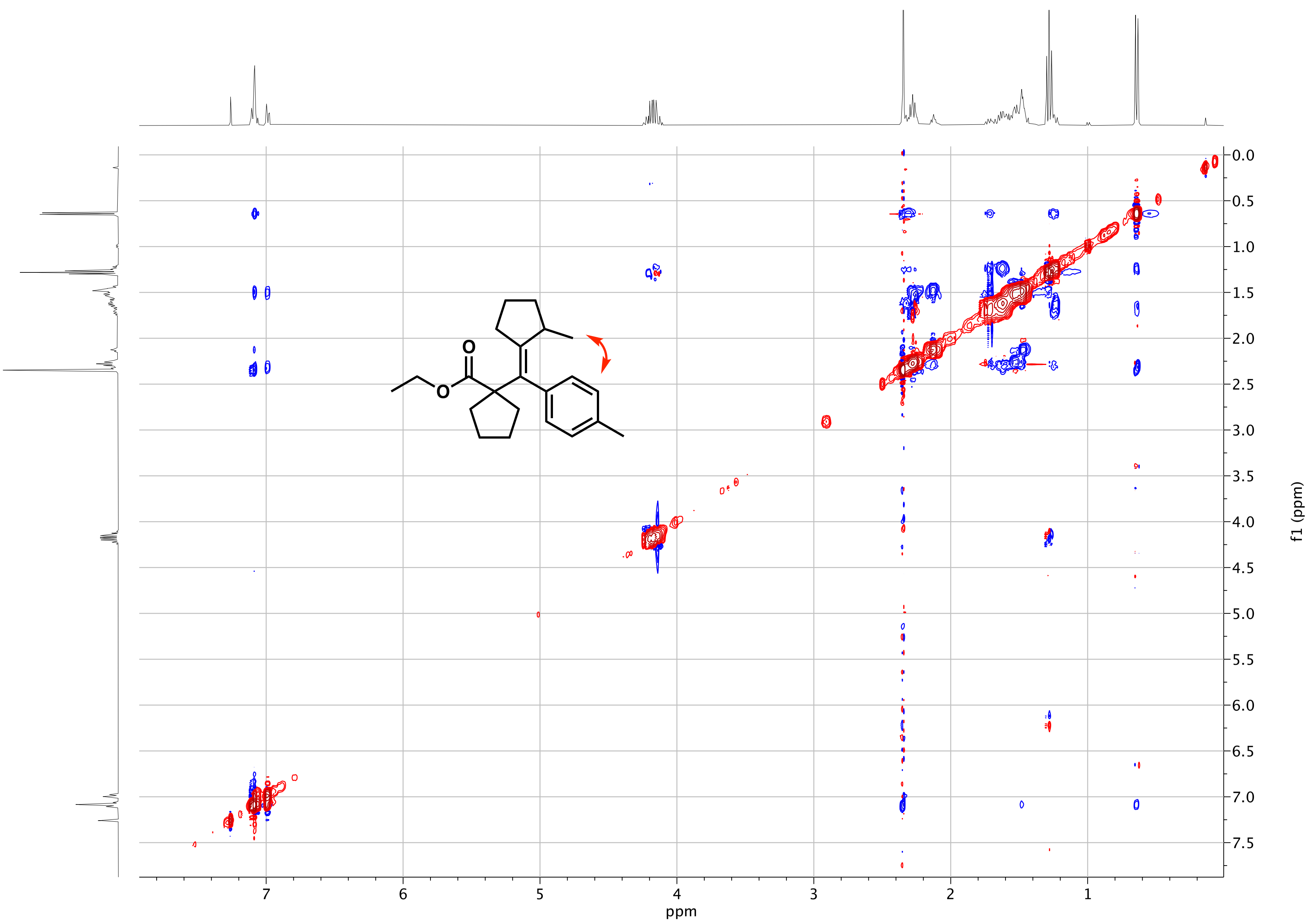

NOESY NMR (400 MHz, $CDCl_3$) of compound **22.**

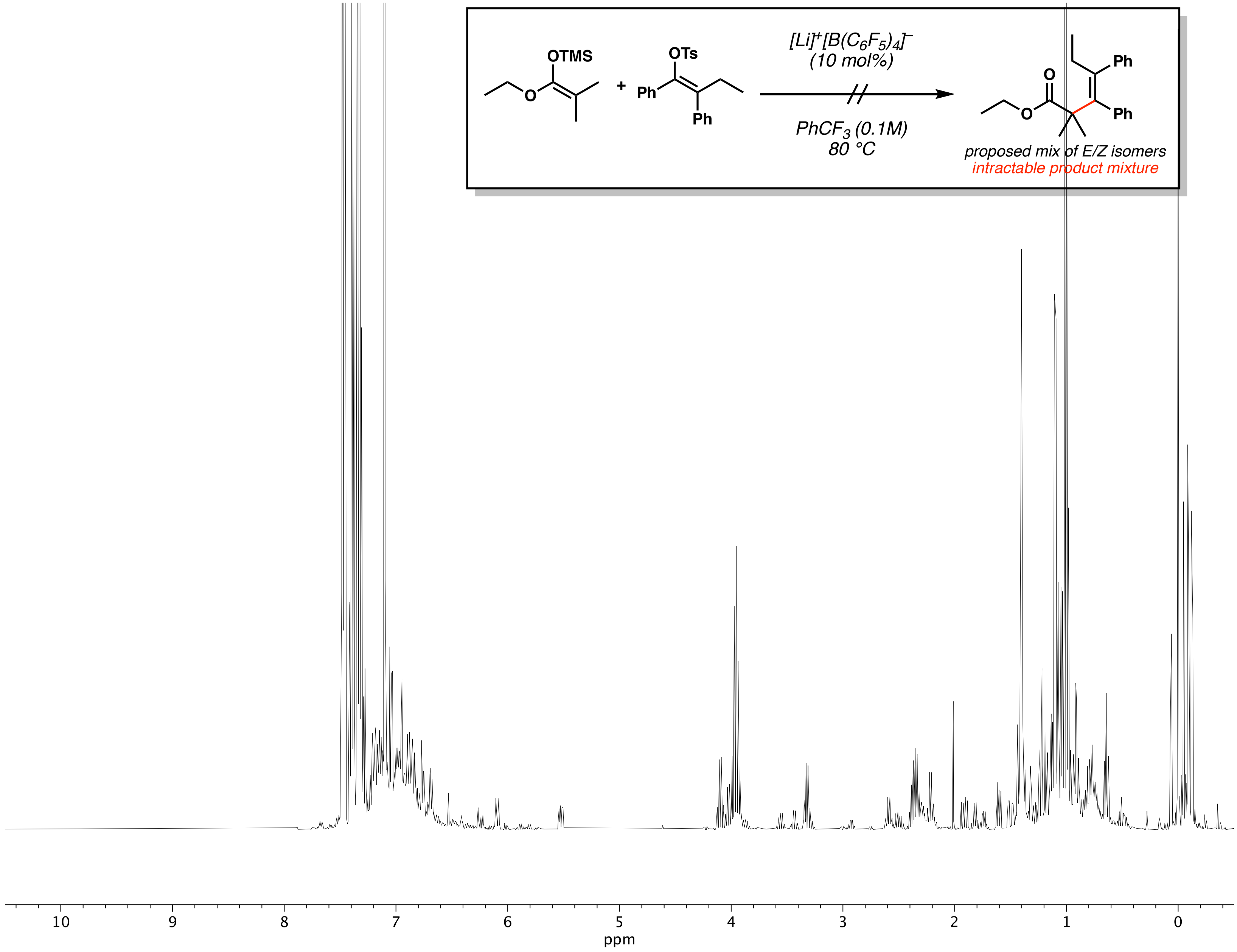

^{1}H NMR (400 MHz, $CDCl_3$) of crude reaction mixture.
